# A tiling microarray for global analysis of chloroplast genome expression in cucumber and other plants

**DOI:** 10.1186/1746-4811-7-29

**Published:** 2011-09-28

**Authors:** Agnieszka Żmieńko, Magdalena Guzowska-Nowowiejska, Radosław Urbaniak, Wojciech Pląder, Piotr Formanowicz, Marek Figlerowicz

**Affiliations:** 1 Institute of Bioorganic Chemistry, Polish Academy of Sciences, Noskowskiego 12/14, Poznan, Poland; 2 Department of Plant Genetics, Breeding and Biotechnology, Faculty of Horticulture and Landscape Architecture, Warsaw University of Life Sciences-SGGW, Nowoursynowska 166, Warsaw, Poland; 3 Institute of Computing Science, Poznan University of Technology, Piotrowo 2, 60-965 Poznan, Poland; 4 Current Address: Friedrich Miescher Institute for Biomedical Research, Maulbeerstrasse 66, P.O. Box 2543, 4002 Basel, Switzerland

## Abstract

Plastids are small organelles equipped with their own genomes (plastomes). Although these organelles are involved in numerous plant metabolic pathways, current knowledge about the transcriptional activity of plastomes is limited. To solve this problem, we constructed a plastid tiling microarray (PlasTi-microarray) consisting of 1629 oligonucleotide probes. The oligonucleotides were designed based on the cucumber chloroplast genomic sequence and targeted both strands of the plastome in a non-contiguous arrangement. Up to 4 specific probes were designed for each gene/exon, and the intergenic regions were covered regularly, with 70-nt intervals. We also developed a protocol for direct chemical labeling and hybridization of as little as 2 micrograms of chloroplast RNA. We used this protocol for profiling the expression of the cucumber chloroplast plastome on the PlasTi-microarray. Owing to the high sequence similarity of plant plastomes, the newly constructed microarray can be used to study plants other than cucumber. Comparative hybridization of chloroplast transcriptomes from cucumber, *Arabidopsis*, tomato and spinach showed that the PlasTi-microarray is highly versatile.

## Background

Plastids form a large family of cellular organelles that occur in plants and algae. The most prominent members of the plastid family are chloroplasts. Chloroplasts use light energy to convert carbon dioxide into organic compounds in a process called photosynthesis. Depending on tissue localization and environmental conditions, other types of plastids may develop. Plastids are also involved in various aspects of plant cell metabolism, e.g., they can store starch, lipids or proteins. Certain factors can induce mature plastids to transform from one type to another, as well as to revert back [1]. The process of plastid biogenesis and interconversion is coupled with large structural and biochemical changes. This huge transformation potential of plastids is partly a result of the presence of their own genetic material (plastome) and inherent transcriptional and translation machinery. The first complete sequences of plastid genomes (from *Nicotiana tabacum *and *Marchantia polymorpha*) were determined in 1986. Currently, more than 200 plastome sequences are available in GenBank. Most of them (more than 170) are derived from flowering plants. The majority of plastomes were sequenced after 2006, when high throughput sequencing methods became more widely available and less expensive [2,3]. The sequences of plastid genomes and their organization are highly conserved. Plastomes range in length from 120 to 200 Mbp. They usually contain two large inverted repeats (IR), namely IRA and IRB, separated by single copy regions. However, in some plants, such as *Medicago truncatula*, the plastomes lack one IR region. Genes encoded in the plastome can be divided into two categories: protein coding (about 70-100 genes, mostly coding for proteins related to the light-phase of photosynthesis or coding for ribosomal proteins), and RNA coding (about 30-50 rRNA and tRNA genes). There are also some conserved open reading frames (conserved ORFs), which have undefined or poorly defined functions. Some plastid genes overlap one another, and many genes are organized into operons, indicative of their prokaryotic origin. The latter are transcribed into polycistronic preRNAs, which are further processed into individual RNA species. The transcripts undergo extensive post-transcriptional modifications, including trans-splicing and RNA editing [4-7].

Plastids do not operate independently of nuclear genetic information. A large number of photosynthesis-related chloroplast proteins are encoded in the nucleus. Similarly, many proteins that are essential for post-transcriptional processing and stabilization of plastid transcripts are encoded in the nucleus and transported to plastids after their synthesis in the cytoplasm [8]. For example, sigma factors are proteins of nuclear origin that confer promoter specificity of plastid-encoded RNA polymerase (PEP) core subunits. This specificity is one of the regulation mechanisms that modulates gene expression under changing environmental conditions [7,9,10]. Apart from PEP, nucleus-encoded phage-type RNA polymerases (NEPs) are also engaged in transcription in plastids [11,12]. It has recently been shown that genes transcribed by PEP are down-regulated and genes transcribed by NEP are up-regulated in tobacco *ΔpsaA *and *ΔpsbA *deletion mutants, which lack genes that code for core components of photosystem I and photosystem II, respectively. These mutations, located in the chloroplast genome, also affect the expression of nuclear genes. Genes related to photosynthesis were down-regulated, and stress-responsive genes were up-regulated [13]. This and many other works demonstrate that plastid genes act in concert with nuclear genome products, allowing plants to adapt quickly and flexibly to changing environmental and developmental conditions. However, although the overall structure and function of plastids are quite well known already, and individual plastid genes have often been subjected to intensive studies, few plastome-scale expression studies have been published so far [6,9,10,13-22]. Moreover, most reported experiments focus on the gene-coding regions, but there is growing evidence that the so-called non-coding parts of genomes may play important regulatory roles in prokaryotes and in eukaryotic organelles [15,23-27]. Therefore, based on cucumber plastid genome sequence, we constructed an oligonucleotide tiling microarray (PlasTi-microarray). Although the probes on the PlasTi-microarray do not overlap nor they are contiguous, this array has the highest resolution of the plastid arrays reported so far and covers both coding and non-coding regions on both strands of the plastome. This array is an excellent versatile tool for global functional studies of plastid genomes. In this paper, we present the microarray design, as well as detailed protocols for chloroplast RNA (cpRNA) sample preparation and hybridization. We also propose general procedures that can be used for PlasTi-microarray data normalization and analysis. We demonstrate that the PlasTi-microarray can be used for analyzing the plastome transcriptome in cucumber and other flowering plants.

## Results

### Construction of the PlasTi-microarray

**Figure 1:**
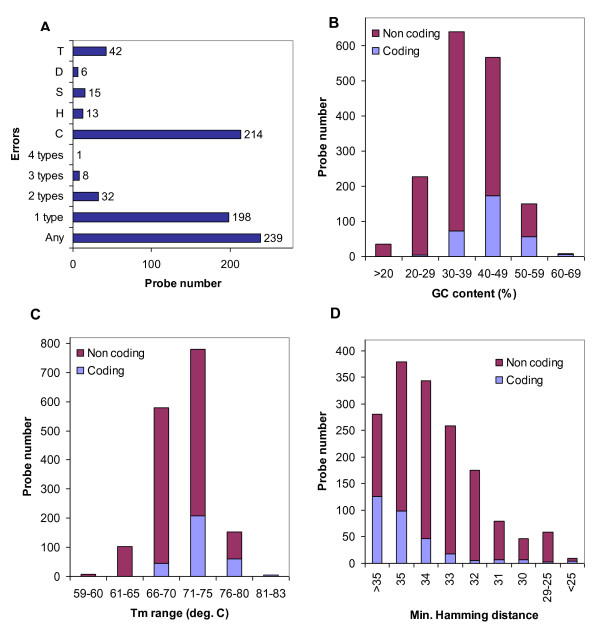
**Presentation of the selected parameters in the final microarray probe set**. **A**. Comparison of numbers of probes not meeting each design criterion (T - Melting Temperature, D - Hamming Distance, S - Substring, H - Hairpin, C - Nucleotide Composition, 1 type (2 types, 3 types, 4 types) - probes for which 1 criterion (2, 3 or 4 criteria, respectively) are not fulfilled, Any - probes that do not meet at least one design criterion). **B**. Frequency distribution of probe GC content (a component of the Nucleotide Composition criterion). **C**. Frequency distribution of probe melting temperatures. **D**. Frequency distribution of minimum Hamming distance of the probes, describing the probe specificity within the cucumber chloroplast genome.

The cucumber plastid genome is 155,293 bp long and contains a pair of large inverted repeats, IRA and IRB (25,191 bp each). These repeats are separated by two single-copy regions - small (SSC, 18,222 bp) and large (LSC, 86,688 bp) [28]. The genome comprises 89 protein-coding genes, 8 rRNA genes and 37 tRNA genes. Some of these genes contain introns [29]. Our aim was to obtain a microarray probe set allowing for both expression studies of known plastid genes or conserved ORFs, as well as discovery of new RNAs transcribed from non-protein coding regions of the cucumber chloroplast genome. To construct such a universal microarray, we employed a tiling strategy. This strategy assumes that probes uniformly cover the whole target sequence, in a regular manner. If the tiling array resolution (the average distance between the central positions of adjacent probes) is equal or lower than the probe length, it is possible to obtain full genome coverage. Here, ~50% coverage of the non-coding part of the genome was obtained by designing 70-nucleotide probes with 140-nucleotide resolution. Uniform tiling of both strands of the chloroplast genome with such a density required the synthesis of over 2200 probes. Considering the specific organization of plastid genomes, we were able to reduce this number. A set of common probes was designed to cover IRA and IRB regions, which are almost (99%) identical. Also, the number of probes in the coding regions was limited to a maximum of four per gene/exon. Gene introns and conserved ORFs of undefined functions were tiled with a density similar to other non-coding regions. To avoid ambiguity in the results, probes located at the borders of coding and intergenic regions were not used. To meet this requirement, 15 probes targeting very short regions (predominantly short exons of tRNA-coding genes) were extended to 70 nt by adding adaptors that were not complementary to the cucumber plastid genome. Other criteria applied to the PlasTi-microarray probe design considered oligonucleotide properties (rule C), their secondary structure (rule H) and probe specificity within the plastid genome (rules D and S) (see Table 1 for details). The limits imposed on specific parameters were based on the criteria used to design the Array-Ready Oligo Set™ for the *Arabidopsis thaliana *Genome (Version 3.0) but were slightly modified to better reflect the nature of the plastome [30]. For example, GC content limits were set to 20% - 60%, as the mean GC content in the plastid DNA sequence is small (about 37% in cucumber) [28]. Also, the specificity rule D was made more stringent. To this end, the minimum Hamming distance to non-target parts of the cucumber plastid genome was > 25. Application of this rule resulted in the exclusion of probes that could potentially cross-hybridize to non-target sequences displaying high (≥ 64.3%) identity to the target region (up to 70% identity was allowed in case of the Operon probes) [30]. Cross-hybridization analysis was made only for the plastid genome sequence because i) the cucumber nuclear genome sequence was unavailable, and ii) the procedure of RNA sample preparation involved chloroplast separation; thus, most transcripts of nuclear origin were removed [see Methods]. In rare cases, some design rules were relaxed when a probe that met all established criteria could not be found (Table 1). As a result, 1629 oligonucleotide probes (70-mers) were designed and synthesized. A total of 315 probes targeted coding regions of the plastome, and 1314 probes targeted the non-coding parts of the plastid genome (introns and intergenic regions) and conserved ORFs. The design rules were relaxed for 239 oligonucleotides; for more than 73% of those "imperfect probes", the rule did not meet the only "Nucleotide composition" criterion (Figure 1). Such a situation is an obvious result of a compromise between the need for a regular probe distribution and the capacity of the probes to hybridize. It should be emphasized that only nineteen probes did not comply with the rules S and/or D, (Substring and Hamming distance, respectively, see (Table 1) for the description of those parameters) which could slightly affect their specificity. The lower density of probe coverage in the coding regions permitted better optimization. Accordingly, the parameters for 91.4% of coding sequence-specific probes met all of the established criteria (Figure 1).

**Table 1:** Criteria for oligonucleotide probe selection

Oligo selection criterion	probes satisfying the rule [%]	Rule symbol
**Oligonucleotide properties**		

Melting temperature = 70°C (+/- 8°C)	97.18%	T

Nucleotide composition: oligonucleotide cannot have a contiguous single nucleotide base repeat longer than 8 bases AND probe GC content fits the 20-to-60% range AND each base cannot constitute more than 40% of the oligonucleotide sequence	85.33%	C

**Secondary structure**		

Stem of potential hairpin structure cannot be longer than 8 bases	99.20%	H

**Probe specificity**		

Minimum Hamming distance to non-target parts of genome > 25	99.45%	D

Substring: not more than 20 contiguous bases common to other (non-target) parts of genome	99.02%	S

A total of 306 probes were common to both the IRA and IRB regions. Sixteen of these probes were not perfectly complementary to one of the IRs because of minimal differences in their nucleotide sequences. However, these discrepancies are minor and would have little impact on the hybridization results (Table 2). Detailed information on probe sequences and target regions is presented as additional data [Additional Files 1 and 2]. Probe names have a uniform format P/MxxxxxxC/N, where the first symbol is a letter indicating the genome strand (P for plus, M for minus), followed by the six-digit number for the genome target coordinates, with the last letter indicating the region type (C for coding, N for non-coding). Probes common to the IR regions are named according to the IRB target coordinates.

**Table 2:** Discrepancies from the probe genome perfect complementarities in inverted repeat regions

Discrepancy	Number of probes affected
70/70 > Identity ≥ 65/65, 0 Mismatches, 0 Gaps	2

Identity = 69/70, 1 Mismatch, 0 Gaps	7

Identity = 69/70 or 70/71, 0 Mismatches, 1 Gap	4

Identity = 68/70 or 70/72, 0 Mismatches, 2 Gaps	2

Identity = 70/76, 0 Mismatches, 6 Gaps	1

Discrepancy occurs when the probe targets IRA and IRB in regions that are not perfectly identical. In this case, the probe is designed to match one IR and is not 100% complementary to the other one. Level of identity of the probe to the second IR region is presented as number of probe bases perfectly aligning to target region/total length of aligning probe sequence. Probe 5' or 3' ends that do not match the second IR, do not add up to the reported total alignment length. Number of internal mismatches and gaps in alignment is also shown

### Evaluation of experimental procedure

All microarray experiments described in this paper were performed using a two-color hybridization approach. Cucumber PlasTi-microarrays were produced with a SpotArray 24 instrument (PerkinElmer). All probes were printed in duplicate on the epoxide-coated glass slides (Corning) in sixteen 17 × 36 print-tip groups, together with Stratagene's SpotReport™ Alien™ cDNA Array Validation System oligonucleotide set and control buffer spots. The probes are complementary to the target sequences (they are also complementary to transcripts, not to cDNA). As a result, a method of direct chemical RNA labeling was chosen. For all experiments described in this paper, the Micromax ASAP RNA labeling kit (PerkinElmer) was used. In the original manufacturer's procedure, the total RNA is chemically modified with either Cy3 or Cy5 during a short incubation, and the labeled mRNA is further purified with Oligotex™ RNA kit (QIAGEN). Here, the total cpRNA was subjected to analysis, so the purification procedure was limited to the removal of non-incorporated dye, without fractionating the labeled RNA. Therefore, the miRNeasy Mini Kit (QIAGEN) was used for purification. This kit preserves shorter RNA molecules (tRNA transcripts, highly abundant in chloroplasts and presumptive regulatory RNAs) from washing out. Also, the amount of input RNA was lowered [see Methods for detailed labeling protocol]. Alternatively, the Micromax ASAP RNA labeling kit can be replaced by the Arcturus^® ^Turbo Labeling™ Kit with Cy3/Cy5 (Applied Biosystems) (data not shown).

### Data normalization and gene expression analysis

**Figure 2:**
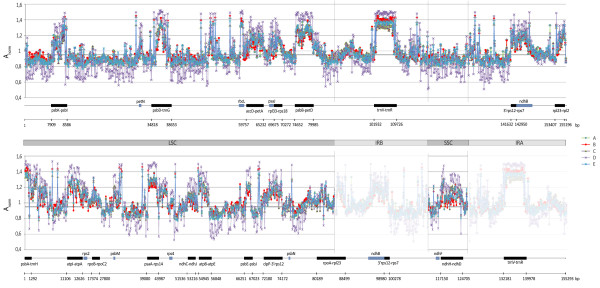
**Patterns of probe signal intensities from five independent microarray experiments**. Comparison of signal intensities of all 1629 microarray probes, obtained from five independent, separately normalized microarray experiments. Probes are ordered according to the genome coordinates of their target regions, on the plus strand (upper diagram) and minus strand (lower diagram). Probes covering IR regions have targets on both strands and are displayed twice; on the minus strand, they are masked by semitransparent blocks. Normalized probe intensity from each of five independent microarray experiments (A-E) is presented (A_norm_). For each probe in each experiment, A_norm _value was obtained by dividing probe intensity (average from two duplicates) by the corresponding A_mean _(defined in Table 2). This enabled signal intensity comparison across all five experiments. All microarrays were analyzed with no background correction, apart from experiment D, for which local background subtraction was applied, due to high background intensity on most microarrays in this set.

The PlasTi-microarray is a combination of two array types, a typical gene expression array and a tiling array, for which different normalization methods are usually applied [for review see [31]]. Accordingly, we needed to determine which approach would be more useful in the case of the PlasTi-microarray. Considering that our microarray contains relatively long probes and that none of them overlaps with the coding and intergenic regions (which have different GC content), we assumed that the effect of sequence-dependent hybridization can be omitted. As a result, the standard (median- or loess-based) normalization methods, commonly used for gene expression arrays, seemed appropriate. However, the application of those methods could be called into question, as they can be used if at least half of the spots on the array produce measured signals, which is usually not the case with the high-density genome tiling arrays. To verify whether such a standard normalization can be applied to the PlasTi-microarray, we calculated average numbers of probes that produced signals higher than the background intensity in microarrays from five separate experiments (A-E, Table 3). Each experiment addressed a specific biological problem and was designed and performed independently of the other ones (see Additional file 3 for more information). All microarrays hybridized within one experiment were normalized together. After data quantification, an average log-2 expression value across all channels and all microarrays from one experiment was calculated for each probe (A_probe_). In addition, a mean signal from all probes (A_mean_) was established. The signals produced by negative controls were used to calculate the average level of background intensity (Aneg_mean_) (Table 3). The controls included printing buffer, an alien cDNA oligonucleotide set and "empty" spots. They generated comparable signals, with those from the empty spots being slightly less intense than those from the buffer or the alien cDNA spots. The Aneg_mean _value was then used to calculate the threshold intensity value (Athr = Aneg_mean _+ 2 SD), above which the spot was acknowledged as "detected" (Table 3). At least two-thirds of the cucumber probes generate signals higher than the threshold value in all five experiments. From this fact, we concluded that the methods developed for standard expression arrays can be used for the normalization of results generated with the PlasTi-microarray. In subsequent analysis, the "print-tip loess" normalization method adopted in the Limma package of the R/Bioconductor project was applied. Microarray normalization performed independently for each experiment resulted in comparable patterns of probe signal intensities on both genome strands, regardless of the experiment type. The observed differences in the absolute values of the signal intensities thus resulted from the varying methods of background correction applied (Figure 2).

**Table 3:** Evaluation of hybridization signal intensities on PlasTi-microarrays

Experiment	A	B	C	D	E
**Overall spot intensity characteristics**					

**A**_**mean**_	9.509	10.164	10.819	10.122	10.269

**Norm. min (A**_**min**_**/A**_**mean**_)	0.508	0.797	0.784	0.814	0.770

**Norm. max (A**_**max**_**/A**_**mean**_)	1.535	1.462	1.362	1.444	1.401

**"Coding" probes with A**_**probe **_**> A**_**mean**_	287 (91.11%)	254 (80.63%)	286 (90.79%)	268 (85.08%)	277 (87.94%)

**"Non-coding" probes with A**_**probe **_**> A**_**mean**_	267 (20.32%)	226 (17.20%)	225 (17.12%)	203 (15.45%)	245 (18.65%)

**Non-specific signal characteristics**					

**Aneg**_**mean**_	5.540	8.276	8.571	8.317	7.989

**BUFFER (Abuff**_**mean**_**/Aneg**_**mean**_)	1.021 ± 0.160	0.999 ± 0.020	1.002 ± 0.027	1.002 ± 0.026	1.001 ± 0.034

**EMPTY (Aempty**_**mean**_**/Aneg**_**mean**_)	0.916 ± 0.043	0.993 ± 0.018	0.989 ± 0.013	0.990 ± 0.014	0.987 ± 0.010

**ALIEN (Aalien**_**mean**_**/Aneg**_**mean**_)	1.031 ± 0.155	1.000 ± 0.026	1.001 ± 0.027	1.001 ± 0.022	1.002 ± 0.029

**Athr**	**7.288**	**8.862**	**9.125**	**8.799**	**8.637**

**Probes with A**_**probe **_**> Athr**	**1094 (67.16%)**	**1298 (79.68%)**	**1244 (76.36%)**	**1250 (76.73%)**	**1225 (75.20%)**

A_mean _is the mean intensity of all probes on all microarrays within the experiment. Aneg_mean _is the mean intensity of all negative control spots on all microarrays within the experiment. Norm.min and Norm.max are normalized minimal and maximal intensity values. Athr is a threshold intensity value qualifying the probe as "detected" and was calculated from the formula Athr = Aneg_mean _+ 2 SD. Experiments A-E are briefly described in the Additional file 3.

**Figure 3:**
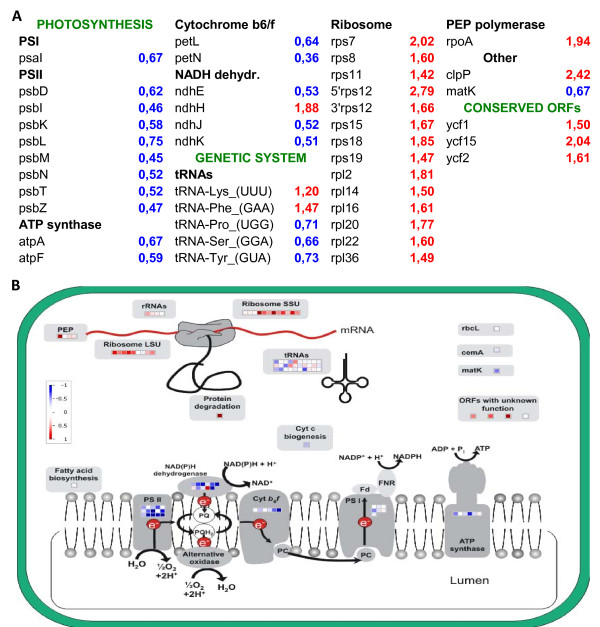
**Comparative analysis of plastid transcriptomes in cucumber**. Gene expression fold change in flowers is presented relative to expression level in leaves. For each gene, an average signal from all probes targeting this gene is reported. Genes are grouped according to their function. A - only results with statistical significance (average p-value of all the probes per gene < 0.05) are presented. B - all gene expression data are displayed on the chloroplast metabolic pathway diagram. Expression changes transformed to logarithmic values are displayed in colors, according to the red-blue scale.

As an example illustrating the application of the PlasTi-microarray to gene expression analysis, we present selected data from experiment E (other results will be presented elsewhere). Comparison of the plastid transcriptome isolated from female flowers to that isolated from the leaves revealed global up-regulation of genes engaged in transcription and translation in the flower plastids (Figure 3A). Thirteen ribosomal protein genes were significantly up-regulated (six with p < 0.01 and the remaining with p < 0.05). A significant increase in transcript accumulation was also observed for *rpoA *(which codes for the PEP polymerase subunit), *clpP *(which codes for ATP-dependent protease) and three conserved ORFs, *ycf1*, *ycf2 *and *ycf15*. The first two of them had previously been shown to be functional genes that are essential for cell survival, suggesting that they are involved in basic plastid metabolism [32]. By contrast, in flowers, photosynthesis-related genes were down-regulated overall. The most notable examples are the significantly lower transcript levels of eight *psb *genes (coding for elements of the photosystem II complex). Many genes coding for components of the ATP synthase, photosystem I, cytochrome b_6_/f and NADH dehydrogenase were also down-regulated. The *ndhH *gene was the only significantly up-regulated photosynthesis-related gene.

To permit better visualization of the expression patterns of functionally related genes, a graphic representation of the expression data was created with the MapMan software (Figure 3B). MapMan is a user-driven tool that superimposes large data sets, for example microarray data, on diagrams of metabolic pathways [33]. For our dataset, a ChloroPlast_CustomArray pathway diagram [20] was modified to display all genes represented on a PlasTi-microarray, and specific mapping files were prepared. Those files were added to MapMan Store. They can be downloaded from the project website and used to visualize any gene expression data produced with PlasTi-microarrays [34].

### Transcriptional activity of non-coding regions

**Figure 4:**
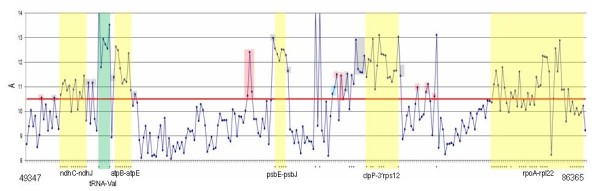
**Signal intensities of the probes targeting gene introns and intergenic regions**. This diagram is a magnified part of a signal intensity diagram for experiment E. The diagram covers a 37,018 -base region of the minus strand, covered by 264 probes. Signals from the probes targeting gene coding regions are marked with asterisks, the operons are marked by yellow blocks, and one gene containing introns is marked by a green block. Signals from the probes targeting non-coding regions that exceed the threshold value (Athr = 10.5) are colored: gray - probes in the proximity of gene coding regions; red - signals from probes in the antisense orientation to genes coded on the plus strand; blue - signals from probes positioned with a distance larger than 600 bases from known coding regions.

As was mentioned earlier, intergenic sequences were covered by PlasTi-microarray probes in a regular manner. This coverage made it possible to survey the transcriptional activity of the regions classified as non-coding. In the analysis of high density tiling arrays, sliding window methods are usually adopted to search for probe intensity peaks [35]. However, those methods may not perform well in this case, owing to the irregular distribution of probes in the coding and intergenic regions and the moderate array resolution (one 70 nt probe per ~140 bases). At such a resolution, even a single high intensity signal may be significant, but such a signal could likely be ignored in the automatic search. Examination of the signals generated by the "non-coding" probes in microarray experiment E showed that their substantial numbers had an intensity higher than A_mean _and were comparable to the intensities of the "coding" probes. Therefore, for "non-coding" probe analysis, we propose a simple approach, based on establishing a cut-off value that is higher than A_mean_, followed by subjecting all of the signals above this threshold to a more detailed investigation. As the total number of "non-coding" probes on the array is 1314 and the majority of them are expected to target regions transcribed at low levels, the number of candidate probes should be reasonably low. In the experiment E, setting the cut-off at 10.5 (A_mean _= 10.27) resulted in 336 candidate probes. Information on the array probe sequence and localization [see Additional Files 1 and 2] will then be helpful to distinguish high intensity signals occurring in the proximity of gene coding regions or in introns from signals generated by antisense transcripts or those that are apparently unrelated to known genes (Figure 4).

### PlasTi-microarray in cross-species hybridization experiments

DNA microarrays have been widely adopted for cross-species hybridization (CSH) in situations where species-specific microarrays were unavailable. Several earlier studies showed that the value of biological data obtained in this way depends on the sequence similarity between cross-hybridized species [36,37]. As the plastomes of higher plants display high levels of sequence similarity, we attempted to determine whether the microarray designed for the cucumber plastid genome can be used to study the chloroplast transcriptomes of other plants. First, each of the 1629 cucumber probe sequences was used as a query in a blastn search against the plastid genomes of nine flowering plants: *Arabidopsis thaliana*, tobacco, tomato, spinach, lettuce, *Medicago truncatula*, *Lotus japonicus*, poplar and barley. The highest similarity was observed for tobacco and poplar plastid genomes, with over 1100 probes (> 67%) matching with E ≤ 10^-10 ^and more than 1250 (> 76%) matching with E < 10^-3^, where E is the expected number of high-scoring segment pairs (Table 4). Barley and spinach plastomes showed the lowest similarity to cucumber probes of all of the species under investigation. Even in those two cases, however, more than 800 queries (~ 50% of all microarray probes) matched the genome with E ≤ 10^-10^. We also observed that probes designed for coding regions of the genome, which are more conserved than non-coding regions, turned out to be the most suitable for CSH studies. More than 98% of the probes designed for the IR coding regions and more than 90% of the probes designed for the SC coding regions have enough similarity to serve as microarray probes in seven of nine analyzed species. In the cases of spinach and barley, the number of matching probes was > 92% and > 81% for IR and SC coding regions, respectively. As whole IR regions are highly conserved in plants, IR "non coding" probes also match all analyzed genomes very well (73-78% of probes in the case of barley, spinach and *Medicago*, and more than 94% for the remaining plants). Non-coding parts of SC regions are less conserved, and so the number of SC "non-coding" probes matching the examined plastid genomes with E < 10^-3 ^was much smaller. Still, it was about 44% - 45% in spinach and barley and 59% - 70% in the remaining plants.

**Table 4:** Sequence similarity of cucumber PlasTi-microarray probes to plastid genomes of other plants

Organism	**PlasTi- microarray probes matching target plastid genome with E < 1 × 10**^**-3**^					**PlasTi- microarray probes matching target plastid genome with E ≤ 1 × 10**^**-10**^
		
	All	SC coding	SC non-coding	IR coding	IR non-coding	
**C.sa**	**1629**	**262**	**1062**	**53**	**252**	**1629**

A.tha	1188 (72.9%)	246 (93.9%)	638 (60.1%)	53 (100%)	251 (99.6%)	1035 (63.5%)

N.tab	1253 (76.9%)	250 (95.4%)	706 (66.5%)	53 (100%)	244 (96.8%)	1108 (68.0%)

S.lyc	1212 (74.4%)	250 (95.4%)	666 (62.7%)	53 (100%)	243 (96.4%)	1086 (66.7%)

S.ole	927 (56.9%)	214 (81.7%)	470 (44.3%)	49 (92.5%)	194 (77.0%)	807 (49.5%)

L.sat	1225 (75.2%)	252 (96.2%)	682 (64.2%)	52 (98.1%)	239 (94.8%)	1078 (66.2%)

M.tru	1101 (67.6%)	238 (90.8%)	615 (57.9%)	52 (98.1%)	196 (77.8%)	944 (57.9%)

L.jap	1164 (71.4%)	247 (94.3%)	625 (58.9%)	52 (98.1%)	240 (95.2%)	1032 (63.4%)

P.tri	1287 (79.0%)	245 (93.5%)	740 (69.7%)	53 (100%)	249 (98.8%)	1142 (70.1%)

H.vul	927 (56.9%)	219 (83.6%)	474 (44.6%)	49 (92.5%)	185 (73.4%)	802 (49.2.%)

Each probe was used as a query in a blastn search against each plastid genome, with the E value threshold set at 1 × 10^-3^. C.sa - *Cucumis sativus *[GenBank: NC_007144], A. tha - *Arabidopsis thaliana *[GenBank: NC_000932], N.tab - *Nicotiana tabacum *[GenBank: NC_001879], S.lyc - *Solanum lycopersicon *[GenBank: NC_007898], S.ole - *Spinacia oleracea *[GenBank: NC_002202], L.sat - *Lactuca sativa *[GenBank: NC_007578], M.tru - *Medicago truncatula *[GenBank: NC_003119], L.jap - *Lotus japonicus *[GenBank: NC_002694], P.tri - *Populus trichocarpa *[GenBank: NC_009143], H.vul - *Hordeum vulgare *[GenBank: NC_008590]. For each genome, number of probes matching with E value lower than the threshold as well as number of probes matching with E ≤ 1 × 10-^10 ^is reported.

**Figure 5:**
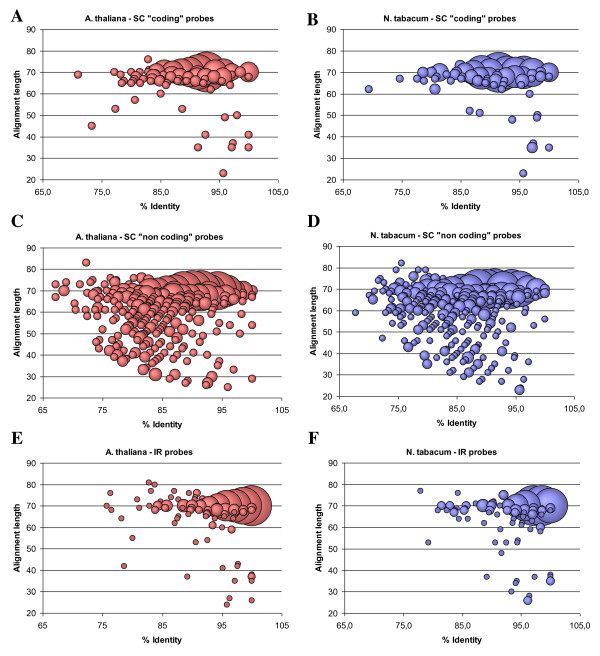
**Similarity of cucumber probes to *A. thaliana *and *N. tabacum *plastid genomes**. Only results with E < 10^-3 ^are presented. For perfectly matching probes, without gaps or mismatches, the alignment length is exactly 70 bases and the identity is 100%. The bubble size reflects the number of probes with the same alignment length and % identity. A, C, E - similarity to *A. thaliana *plastid genome; B, D, F - similarity to *N. tabacum *plastid genome.

**Figure 6:**
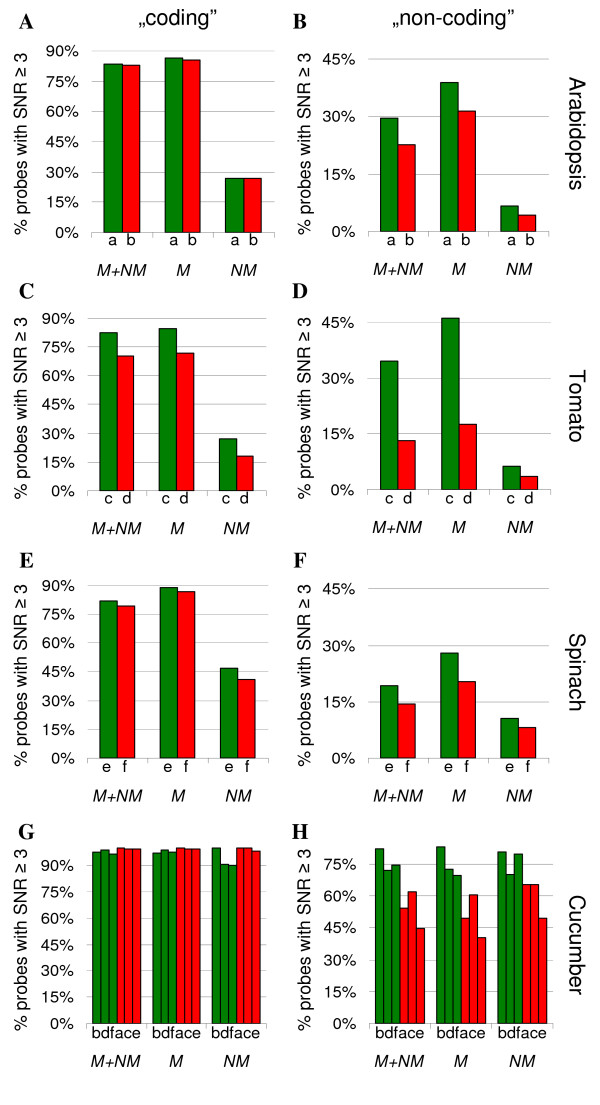
**Proportion of PlasTi-microarray probes with SNR ≥ 3 in CSH-based microarray experiments**. Graphs present percentages of probes with SNR ≥ 3 in CSH-based microarray experiments, separately for "coding" (panels A, C, E) and "non-coding" probes (panels B, D, F). Each bar represents the value obtained from one channel of one microarray. Only probes with both replicate spots flagged as good were considered, and the SNR values were averaged between the replicates. a-f - arrays, as indicated in (Table 5). M -probes matching analyzed genome sequence with E ≤ 10^-3^, NM - probes not matching the genome sequence with E ≤ 10^-3^. Colors represent array channels: green - Cy3 channel, red - Cy5 channel.

More detailed examination of aligning sequences (selected by blastn search) made for *Arabidopsis *and tobacco further confirms the high applicability of the designed probes in CSH experiments (Figure 5). More than 90% of blastn-selected probes targeting SC coding and whole IR regions of the *Arabidopsis *and tobacco plastid genomes, align across at least 60 bases with ≥ 80% identity, which indicates that there are not many gaps or mismatches within the alignment. This level of similarity is enough to allow successful hybridization of the cucumber microarray probes to plastomes of other species. To verify in practice whether the PlasTi-microarray is truly well suited for CSH studies, a trial set of hybridization experiments was performed. Chloroplast RNA from *Arabidopsis*, tomato and spinach leaves was extracted, labeled and hybridized to the PlasTi-microarray with the cucumber samples labeled with the second dye as a control. For each species, one biological sample and one technical replicate (labeled in a dye-swap orientation) were analyzed, resulting in two microarray hybridizations per species and six microarrays (a-f) in total (Table 5). After image acquisition, signal quantification and quality control, data from each channel on each microarray were analyzed separately. All control and negative probes were excluded from further calculations, as were probes with at least one spot replicate flagged as "bad". The signal-to-noise ratios (SNR) of the remaining probes were then estimated by averaging SNR values from two spot replicates. Subsequently, the number of "coding" probes with average SNR ≥ 3 was estimated as a percentage of all "coding" probes. Additionally, the fraction of probes with average SNR ≥ 3 was calculated separately for "coding" probes matching and "coding" probes not matching the analyzed genome (Figure 6A, C, E). Similar calculations were performed for the "non-coding" probes (Figure 6B, D, F). Matching probes were selected on the basis of blastn similarity search results, independently for each species (see Table 4 and the text above). To allow results comparisons, the "matching" probe set for cucumber was always identical to the "matching" set of co-hybridized species; therefore, it varied among arrays. As one might expect, in cases of species-specific hybridization (cucumber), the number of probes (both "coding" and "non-coding") with SNR ≥ 3 was always higher than in cross-species hybridization. In all examined species, the SNR values of "non-coding" probes were lower compared with the SNR values of the "coding" probes. Those "non-coding" probes by definition target regions that are unlikely to be intensively transcribed. Additionally, all those regions are less conserved compared to coding regions. Consequently, for *Arabidopsis*, spinach and tomato complementarity between probes and target sequences is reduced. In the case of "coding" probes, more than 96% of them had a SNR above the set threshold in cucumber. In the case of cross-species hybridization, the calculated SNR ratios were above the threshold for 70-83% of the "coding" probes. In each case, probe filtering (based on the results of blastn searches) increased the percentage of probes with SNR ≥ 3. This relationship was observed for both "coding" and "non-coding" probes. It was also true for both green and red channels, regardless of the dye effect. In our hands, the Cy3 signals gave overall higher absolute SNR values compared to Cy5, even when the total intensity of the Cy5 channel was twice as high as the Cy3 channel (for details compare Figure 6 and Table 5). Therefore, the initial filtering out of probes with low sequence similarity to the target plastid genome is highly recommended.

**Table 5:** Summary of CSH microarray experiments

Array	Green Channel (Cy3)	Red Channel (Cy5)	No. of probes with both spot replicates flagged as "good"		Cy5/Cy3 total intensity ratio of probes
				
			"coding"	"non-coding"	
a	Arabidopsis	cucumber	306	1251	1.96

b	cucumber	Arabidopsis	306	969	2.13

c	tomato	cucumber	308	1165	2.24

d	cucumber	tomato	307	1114	1.76

e	spinach	cucumber	307	1129	2.11

f	cucumber	spinach	308	1147	1.18

## Discussion

The past twenty years were remarkable for the development of high throughput methods in biology. This development resulted in researchers' focusing their interests on complex systems (e.g., genomes, transcriptomes and proteomes) instead of on individual genes or pathways. For more than a decade, DNA microarrays have been the leading tools for analyzing global gene expression in plants and animals. These tools are still attractive and offer a relatively cheap alternative to next generation sequencing. However, so far microarrays have been rarely used for the global analysis of plastome expression. Plastid gene expression has been assessed with whole-genome expression arrays, such as GeneChip *Arabidopsis *ATH1 Genome Arrays (Affymetrix). These arrays contain probe sets for almost 24,000 genes, both nuclear and organellar. The use of such arrays is beneficial for providing information about possible co-regulation of nuclear and organelle genes [13,22,38]. However, in cases in which such data are unnecessary, the genome arrays are too expensive and too complex for studying the plastome. This issue has raised a need for constructing plastome-specific microarrays. So far, only about ten plastid genome-specific macro- or microarrays have been developed (Table 6). Almost all of them cover only gene coding regions, and all but one are based on PCR-generated cDNA probes. The sole oligonucleotide-based plastome microarray reported so far has been designed as a tool for gene expression analysis in the *Solanaceae *species (tobacco, potato and tomato). This microarray contains 128 probes, printed in duplicate, each representing one gene, or conserved ORF, plus five additional probes to compensate for sequence differences between tobacco and two other *Solanaceae *species [20]. The *Solanaceae *plastid microarray was used in gene expression studies, including the analysis of tomato fruit development and chloroplast-to-chromoplast conversion or potato tuber amyloplasts vs leaf chloroplasts comparisons [6,20]. For now, this microarray can be considered the tool of choice for the analysis of plastome protein coding gene expression in the *Solanaceae *family. However, the *Solanaceae *microarray does not enable analysis of the non-coding regions. Only two arrays presented in Table 6 include probes for non-coding regions. The first one, constructed by Schmitz-Linneweber et al., was employed for searching maize chloroplast RNAs that bind to pentatricopeptide proteins (PPRs), CRP1, PPR4 and PPR5 using the RIP-chip (RNA immunoprecipitation and chip hybridization) method. For this purpose, cDNA microarrays were generated, consisting of 248 probes that covered the whole plastome of maize [39]. The probes varied in length, from 73 to 1653 bp and overlapped each other. Similarly, Nakamura et al. used 220 PCR amplicons (71 - 2373 bp), each corresponding to a single known gene or an intergenic region, to build a genome microarray for the tobacco plastome [15]. Resolution of both maize and tobacco cDNA microarrays is far too low to enable precise analysis of transcriptional activity of non-coding regions. Moreover, the PCR-generated DNA probes do not distinguish between the plus and minus strands, which is why the transcriptional activity of the antisense regions is masked by signals from the sense transcripts. All of those pitfalls are omitted in the PlasTi-microarray. The two major factors that ensure the high specificity of the PlasTi-microarray are the algorithm applied for the oligonucleotide probe design and the method used for cpRNA separation (this method prevents cross-hybridization with nuclear transcripts). In addition, the application of antisense probes, which are untypical for microarrays, allows for direct labeling of cpRNA, using chemical dye coupling methods. It is easier and faster than performing reverse transcription with random primers. Moreover, direct labeling eliminates numerous problems that can arise during reverse transcription, e.g., false-positive results caused by RNA self-priming [40]. The PlasTi-microarray also has proved to be highly sensitive. This microarray allowed us to detect as low as 2 μg cucumber cpRNA, without the need for material amplification.

**Table 6:** Published plant plastome macro- and microarrays

Organism	Array description	Experiment	Ref
tobacco	nylon macroarray	leaves of wild-type vs transplastomic tobacco lacking PEP	[14]

tobacco	cDNA microarray; 220 PCR probes (71 - 2373 bp), each corresponding to a single known gene or an intergenic region	light- or dark-grown seedlings, RIP-chip analysis of MatK-bound RNAs	[15,47]

*Physcomitrella patens*	cDNA microarray; 108 DNA fragments to detect all annotated plastid genes	analysis of knockout transformant for the arginine tRNA gene, trnR-CCG	[16]

*Arabidopsis*	cDNA microarray; 79 PCR probes (88 - 1646 bp) representing protein-coding genes	effects of the *sig2 *lesion on the global plastid gene expression	[9]

Maize (used also for barley in CSH studies)	cDNA microarray; 248 overlapping PCR products (73 - 1653 bp) covering the whole plastid genome	identification of RNAs associated with PPR proteins in maize (CRP1, PPR4, PPR5) or whirly1 in barley, by RIP-chip	[39,48,49]

*Chlamydomonas reinhardtii*	cDNA microarray; PCR products (150 - 1500 bp) for 47 chloroplast, 9 mitochondrial, and 15 nuclear genes	analysis of nonphotosynthetic mutants carrying mutations in the *Mcd1 *nuclear gene	[17]

*Cyanidioschyzon merolae *(red algae)	cDNA microarray; 193 PCR probes for protein coding genes and orfs	role of nuclear-encoded sigma factors in plastid transcriptome changes during the shift from dark to light	[10]

wheat (used also for barley and rice in CSH studies)	nylon macroarray; 67 PCR products (200 bp - 1259 bp) representing 60 wheat plastid genes (excluding tRNAs) and 7 nuclear genes related to plastid metabolism	germinating seeds and seedlings at three different stages of development	[18]

Maize	cDNA microarray; PCR probes for 887 nuclear, 62 chloroplast, and 27 mitochondrial transcripts	comparison of chloroplasts and etioplasts in stage 2 semi-emerged leaf blades of one month-old plant	[19]

tobacco, potato, tomato	oligonucleotide microarray; 128 probes (68-71 bases) representing tobacco genes, ycfs and orfs (+ 5 probes designed for potato and tomato due to insufficient homology of tobacco probes)	tomato fruit development and chloroplast-to-chromoplast conversion; potato tuber amyloplasts vs leaf chloroplasts comparison	[6,20]

*Euglena gracilis*	nylon macroarray; 96 PCR probes (75 - 400 bp), representing all *Euglena *genes, pseudogenes and orfs	12 different developmental stages and stress treatments	[21]

*Arabidopsis*	nylon macroarray; 94 PCR probes for genes encoding plastid proteins, tRNAs and rRNAs; data were complemented with analysis of published data from Affymetrix 22 K ATH1 array experiments	numerous nuclear *Arabidopsis *mutants affected in diverse chloroplast functions and wild-type plants subjected to various stresses and conditions	[22]

The PlasTi-microarray tiles the whole plastid genome with a resolution that is much higher than any plastid array reported so far. Still, this resolution is not comparable to that of regular high-density tiling arrays, where probes often overlap each other. We show that this moderate resolution can be beneficial for the simplicity of the data analysis pipeline. The small size of the plastid genomes and the perfect separation of the probes that target coding and intergenic regions allowed us to successfully adopt normalization methods dedicated to expression arrays. The use of these methods is further justified by recent findings that standard (sequence-independent) normalization methods perform equally to sequence-dependent methods even for high-density tiling arrays with short oligonucleotide probes [41]. Also, the analysis of the non-coding regions can simply be based on the probe intensity threshold and can be performed with Excel or similar software. The interactive map that shows the localization of the probes on the cucumber genome sequence is presented as supplementary material [Additional File 2]. This map provides the possibility of distinguishing between the signals located near or opposite to gene coding regions and the signals that apparently are not linked to those parts of the genome. This kind of information can provide clues to the significance of transcripts detected in the intergenic regions.

Using the PlasTi-microarray, we compared the patterns of gene expression in the cucumber plastids isolated from female flowers and from mature leaves. We observed that profiles of functionally related gene expression are congruent, e.g., in flowers; the expression of plastid genetic machinery-related genes is enhanced and the expression of photosynthesis-related genes is weakened. Also, the co-transcribed genes, for example genes of the *rpl23*-*rpoA *operon, often shared a similar expression pattern. These results are consistent with observations made by Cho and coworkers during their DNA macroarray-based studies of plastid gene expression in *Arabidopsis *flowers and leaves [22]. They reported significant down-regulation of many photosynthesis-related genes and up-regulation of the plastid genetic system-related genes. The relative fold changes were higher in Cho's *Arabidopsis *studies than in our study. However, the PlasTi-microarrays are sensitive enough to detect even minor changes in gene expression. The expression profiles of functionally related genes generated with our microarray were more congruent than those obtained by Cho et al. (except for tRNA genes, see Figure 3). In Cho's experiments, the expression profiles of genes coding for particular components of protein complexes were often contradictory. For example, of the genes encoding the ATP-synthase protein complex, two (*atpA *and *atpF*) were up-regulated, and the other two (*atpH *and *atpI*) were down-regulated. Similarly, among the Photosystem I complex coding genes, *psaA *and *psaB *were up-regulated, whereas *psaC*, *psaI *and *psaJ *were down-regulated [22].

The high sequence similarity of plant plastomes allows the application of the PlasTi-microarray in cross-species hybridization studies. The probe length (70 nt) ensures high specificity and low sensitivity to single nucleotide mismatches, which frequently occur during inter-species hybridization. Accordingly, for *Arabidopsis*, spinach and tomato, we were able to obtain a high number of signals with SNR ≥ 3, the value widely used as a threshold for flagging spots of bad quality. Moreover, we showed that filtering the microarray probe set based on the homology of the probes to the analyzed genome can further increase the ratio of probes with high SNR values. Homology-based probe filtering is a common practice in the inter-species microarray studies [cited in [36]]. As the number of published plastid genome sequences is large and still growing, initial *in silico *evaluation of the PlasTi-microarray probe specificity can easily be performed before cross-species hybridization experiments.

## Conclusions

We have presented in detail the PlasTi-microarray design and hybridization procedure. We show that this procedure can provide high-resolution data without the need for sophisticated analysis. The PlasTi-microarray allows the expression of both coding and non-coding plastome regions to be surveyed and generates data with at a level of resolution that has not been reported for previous plastid arrays. The microarrays are available from the authors on a cost-reimbursement basis, to academic and non-profit institutions.

## Methods

### Plant growing and material collection

All cucumber plants were in var. Borszczagowski background. Seeds were imbibed in water for several hours and sterilized in a regular bleaching reagent (Domestos, 25% v/v sol., 10 min.), followed by extensive washing with the sterile water. Seeds were grown for 4 days on plates with damp blotting-paper. After that seedlings were transferred to watered peat Jiffy pellets (Agrowit, Poland) and grown as follows: (i) experiments A, B and C - wt plants were grown in versatile chamber MLR-350H, Sanyo at with 16 h light at 25°C/8 h dark at 23°C cycles (120-160 μmol*m^2^*s^-1 ^light), with Jiffy pellets 2/3 immersed in water. 1st and 2nd leaves of 3-4 week old plants were collected; (ii) experiment D - plants (wt, *msc16 and tch03 *lines) were grown in phytotron at 25°C/8 h dark at 23°C cycles (160-200 μmol*m^2^*s^-1 ^light). Plants were transferred to 8-cm pots after 2 weeks. Five fully developed leaves were collected from each of 6-week old plants; (iii) experiment E - wt plants were grown in greenhouse (natural light, supported by sodium lamps (16 h light/8 h dark cycles, 100-300 μmol*m^2^*s^-1^). Plants were transferred to 30-cm pots after ten days. Material (young and mature leaves, female flowers, growing points, young fruits) was collected from 9-week old plants. For etiolated seedlings (experiment E), seeds were grown for 4 days at dark, at 28°C, before seedling collection.

Whenever necessary (experiments B and C), plants were stressed before collection (see Additional file 3 for description, details will be described elsewhere). All material was roughly grounded in liquid nitrogen and stored at -80°C.

For CSH studies, *Arabidopsis*, tomato and spinach seedlings were grown as described above. *Arabidopsis *and spinach plants were further grown in peat Jiffy pellets, in a phytotron at 20°C with 8 h light/16 h dark cycles (160-200 μmol×m^2^×s^-1 ^light). Mature leaves were collected from 6-week-old plants. Tomato plants were cultured in a greenhouse in mineral wool. Fully developed leaves were collected from 12-week-old fruiting plants. The material was roughly ground in liquid nitrogen and stored at -78°C.

### cpRNA isolation and labeling

Chloroplasts were isolated according to [42] with the following modifications: the sorbitol concentration was increased to 0.4 M in buffers SGB (Sorbitol Grinding Buffer) and SDB (Sorbitol Dilution Buffer). Chloroplast isolation was conducted on ice, and samples were centrifuged at 4°C. Briefly, plant tissue was soaked and ground using a mortar and pestle in homogenization buffer (SGB; 0.4 M sorbitol, 50 mM HEPES, 2 mM EDTA, 1 mM MgCl_2_, 0.1% bovine serum albumin, 1% PVP; pH 7.4). The homogenate was filtered through Miracloth and centrifuged at 3,000 × g for 5 min. The pellet was resuspended and washed twice in sorbitol dilution buffer (SDB; 0.4 M sorbitol, 20 mM HEPES, 2 mM EDTA, 1 mM MgCl_2_, 0.1% bovine serum albumin, pH 7.4). The chloroplasts were loaded onto step gradients (30%:70% Percoll in SDB) and centrifuged for 30 min at 4°C at 1,500 × g. The chloroplasts were recovered from the Percoll interface and washed twice in SDB buffer. Only intact chloroplasts were used for cpRNA isolation.

The cpRNA was extracted from the isolated chloroplasts with the use of an miRNeasy Mini Kit (Qiagen) following the manufacturer's instructions. Potential DNA contamination was removed with TURBO DNA-free™ (Ambion) according to the manufacturer's instructions with the following modifications: i) 0.5 U of enzyme was used per 100 μl reaction, and ii) the time of incubation was shortened to 10 minutes. The cpRNA was precipitated with ethanol and redissolved in water to achieve a final concentration of ~ 500 ng/μl. Then, 2 μg of cpRNA was used per 20 μl labeling reaction with MICROMAX ASAP RNA Labeling Kit (PerkinElmer). The reaction mixture included 2 μl of Cy3 or Cy5 dye and 10 μl of labeling buffer. After a 15 min incubation at 85°C in a TC-3000 thermocycler (Techne), the mixture was rapidly cooled to 4°C. A 5 μl amount of Stop Solution was then added, and the samples were cleaned up, as described below.

For removing the unbound dye, Cy3 and Cy5 corresponding reactions were combined and cleaned up with an miRNeasy Mini Kit (Qiagen), following the isolation protocol (starting with the ethanol addition step). RNA was eluted twice with 35 μl water that had been preheated to 55°C. Labeling efficiency was controlled on Nanodrop 1000. Samples were then concentrated in a SpeedVac, to < 10 μl volume, denatured at 68°C for 5 min and mixed with 115 μl of SlideHyb #3 Buffer (Ambion) that had been preheated to 68°C. All samples were incubated at 43°C (for 5 - 30 min) prior to hybridization.

### Oligonucleotide probe design and selection

The oligonucleotide probe design and selection algorithm was written in C++ Builder. The cucumber plastid genome sequence, deposited in GenBank under accession number AJ970307, was used as a template. At the beginning of the design procedure, all possible 70-mer nucleotides (with a 1-nucleotide shift) were generated for each strand. We analyzed their nucleotide composition, melting temperature (Tm, calculated from the equation 64.9+(41 × (GC-16.4))/N, where N is probe length and GC is the sum of G and C occurrences in the probe) and secondary structure (determined by the hairpin detection algorithm, followed by mFold analysis of best candidates). To evaluate oligonucleotide specificity, the Hamming distance and substring length in comparison to all other possible 70-mers within the genome were calculated for each candidate sequence. Oligonucleotides not satisfying the threshold criteria, described in Table 1 of the Results section, were penalized: S - 20 points, H - 8 points, T - 5 points and C - 2 points. The best set of probes was chosen within the desired locations (contiguous coding/non coding regions), with an assumed distance between neighboring probes of 140 +/- 10 bases (and up to 4 probes per coding region) by selection of probes with the highest D parameters and 0 penalty points. If there were no oligonucleotides satisfying those criteria, probes with i) the lowest number of penalty points and ii) the highest D parameter were chosen.

### Microarray preparation and hybridization

Probes (20 μM) (Amino-C6 70-mers, Operon) in Epoxide Spotting Buffer (IDT) were spotted in duplicate onto epoxide-coated slides (Corning) using SpotArray24 arrayer (PerkinElmer), with 16 pins. As controls, SpotReport Alien Oligo Array Validation System oligonucleotides (Stratagene) were spotted, each in 24 or 32 replicates. Also, 274 buffer spots were printed. The array design has been deposited in the Array Express database under accession ID A-MEXP-2057. Prior to use, microarrays were cross-linked in a UVIlink crosslinker (UVITEC Cambridge) with 150 mJ energy. Pre-hybridization and hybridization were performed in an automatic HybArray12 station (PerkinElmer). Prehybridization (in 5 × SSC, 0.1% SSC, 0.1 mg/ml BSA) was performed at 42°C for 45 min, followed by washing at 25°C with 0.1 × SSC (30 s flow, 1 min hold, 3 cycles) and water (15 s flow, 15 s hold, 2 cycles). Slides were drained by centrifugation in a High-Speed Centrifuge (ArrayIt) and loaded into clean hybridization chambers. Hybridization was conducted at 43°C for 17 hours, followed by washing with: LS Buffer (2 × SSC, 0.1% SDS) at 43°C, MS buffer (0.5 × SSC) at 30°C and HS buffer (0.005 × SSC) at 25°C, each for 5 cycles (20 s flow, 40 s hold). Slides were then removed, immersed in 0.05 × SSC, drained and scanned on a ScanArray Express scanner (PerkinElmer).

### Microarray data analysis

For quantitative analysis filtering, GenePix Pro v. 6.1 (Axon Instruments) was used. Spots with more than 10% pixels with signal saturation in both channels, as well as visually bad or contaminated spots, were flagged out. Further data analysis was performed with R/Bioconductor packages. Hybridization quality was assessed with ArrayQuality [43] and ArrayQualityMetrix [44]. Limma was used for normalization and assessing gene differential expression [45]. Data filtering and other calculations were made in Microsoft Office Excel 2003 and 2007. Microarray data have been deposited to ArrayExpress Database and assigned accessions E-MEXP-3227 (analysis of organ-specific gene expression in cucumber) and E-MEXP-3220 (CSH validation experiment).

### Homology searches

The PlasTi-microarray probes in fasta format were used as queries for homology searches *via *the NCBI web page [46], using the blastn algorithm, with the following parameters: word size = 11; Expect threshold = 0.001; Match/Mismatch scores = 1,-1; Gap costs: Existence 2; Extension 1. Search results were further analyzed in Excel 2003.

## Competing interests

The authors declare that they have no competing interests.

## Authors' contributions

AŻ designed and produced microarrays, performed the microarray experiments and analyzed CSH data. MG-N collected plant material, performed RNA isolation and labeling, and analyzed gene expression data. RU and PF wrote the oligonucleotide design and selection algorithm. WP and MF conceived and designed the study. AŻ and MF wrote the manuscript. All authors contributed to the preparation and approval of the final manuscript.

## Supplementary Material

Additional file 1 **1.6 k plastid microarray probe set**. This file provides detailed information about probe names and sequences, as well as information about the type and coordinates of the target regions in the cucumber plastome. Click here for file

Additional file 2 **Map of the microarray probes on the cucumber genome**. This mini web site provides a graphical view of probe positioning in the cucumber plastome sequence. The site allows quick investigation of the sequence context for each probe of interest by browsing to the desired region (as specified by the probe name). After pointing the cursor to the yellow field, which represents the probe target region, information on the probe and target will be displayed. E - exon, I - intron, - - non-coding region. Click here for file

Additional file 3 **1.6 k plastid microarray probe set**. This file provides a brief description of microarray experiments A-E and their design. Click here for file
