# Supplementary material for: A tiling microarray for global analysis of chloroplast genome expression in cucumber and other plants
Source: Plant Methods. 2011 Sep 28;7:29. doi: 10.1186/1746-4811-7-29 (PMC3195753; doi:10.1186/1746-4811-7-29)
Supplement: Additional file 2 — Map of the microarray probes on the cucumber genome. This mini web site provides a graphical view of probe positioning in the cucumber plastome sequence. The site allows quick investigation of the sequence context for each probe of interest by browsing to the desired region (as specified by the probe name). After pointing the cursor to the yellow field, which represents the probe target region, information on the probe and target will be displayed. E - exon, I - intron, - - non-coding region. [file 1746-4811-7-29-S2.HTML]

**Map of the microarray probes on the cucumber genome**
  

---

  
Legend:  
   Gene coding region  
   Intron  
   Intergenic region  
   "Target region for the microarray probe"  
  

---

  
000001 "TTATGGGCGA ACGACGGGAA TTGAACCCGC GCATGGTGGA TTCACAATCC ACTGCCTTGA TCCACTTGGC"   
       AATACCCGCT "TGCTGCCCTT AACTTGGGCG CGTACCACCT AAGTGTTAGG TGACGGAACT AGGTGAACCG "  
  
000071 TACATCCGCC CCCTACCCTA TATATAAGTA GAAATTATAA AAAAAAAGTT ATGTAAATTT TATTAATTAA   
       "ATGTAGGCGG" GGGATGGGAT ATATATTCAT CTTTAATATT TTTTTTTCAA TACATTTAAA ATAATTAATT   
  
000141 AAAGGAGCAA "TACCAATCCT CTTGAGAAAA CAAGAAATTG GTTATTGCTC CTTTACTTTC AAGAACTCGT "  
       TTTCCTCGTT AT"GGTTAGGA GAACTCTTTT GTTCTTTAAC CAATAACGAG GAAATGAAAG TTCTTGAGCA "  
  
000211 "ATACACTAAG" ACAGAAGTCT TTATCCATTG ATAGATGGAA CTTCAACAGC AGCTAGGTCT AGA"GGGAAGT "  
       "TATGTGATTC TG"TCTTCAGA AATAGGTAAC TATCTACCTT GAAGTTGTCG TCGATCCAGA TCTCCCTTCA   
  
000281 "TGTGAGCATT ACGTTCATGC ATAACTTCCA TACCAAGATT AGCACGGTTA ATAATATCAG CCC"AGGTATT   
       ACACTCGTAA TGCAAGTACG TATTGAAGGT ATGGTTCTAA TCGTGCCAAT TATTATAGTC GGGTCCATAA   
  
000351 AATTACACGA CCTTGACTAT CAACTACAGA TTGGTTGAAA TTGAAACCAT TTAAGTTGAA "AGCCATAGTG "  
       TTAATGTGCT GGAACTGATA GTTGATGTCT AACCAACTTT AACTTTGGTA AATTC"AACTT TCGGTATCAC "  
  
000421 "CTAATACCTA AAGCGGTGGA CCAGATACCT ACTACAGGCC AAGCAGCTAG GAAGAAATGT" AAAGAACGAG   
       "GATTATGGAT TTCGCCACCT GGTCTATGGA TGATGTCCGG TTCGTCGATC CTTCT"TTACA TTTCTTGCTC   
  
000491 AGTTGTTGAA ACTAGCATAT TGGAAGATCA ATCGGCCAAA ATAACCATGA GCAGCTACGA TATTATAAGT   
       TCAACAACTT TGATCGTATA ACCTTCTAGT TAGCCGGTTT TATTGGTACT CGTCGATGCT ATAATATTCA   
  
000561 TTCTT"CCTCT TGACCGAATC TGTAACCTTC ATTAGCAGAT TCATTTTCTG TGGTTTCCCT GATCAACTTA "  
       AAGAA"GGAGA ACTGGCTTAG ACATTGGAAG TAATCGTCTA AGTAAAAGAC ACCAAAGGGA CTAGTTGAAT "  
  
000631 "GAAGT"TACCA AGGAACCATG CATAGCACTG AATAGGGAGC CGCCGAATAC ACCAGCTACA CCTAACAT"GT "  
       "CTTCA"ATGGT TCCTTGGTAC GTATCGTGAC TTATCCCTCG GCGGCTTATG TGGTCGATGT GGATTGTACA   
  
000701 "GGAATGGGTG CATAAGGATG TTGTGCTCAG CCTGGAATAC AATCATGAAG TTGAAAGTAC CAGAGATT"CC   
       CCTTACCCAC GTATTCCTAC AACACGAGTC GGACCTTATG TTAGTACTTC AACTTTCATG GTCTCTAAGG   
  
000771 TAGAGGCATA CCGTCAGAAA AGCTTCCTTG ACCAATTGGG TAAATCAAGA AAACAGCAGT AGCTGCTGCA   
       ATCTCCGTAT GGCAGTCTTT TCGAAGGAAC TGGTTAACCC ATTTAGTTCT TTTGTCGTCA TCGACGACGT   
  
000841 A"CAGGAGCTG AATATGCAAC AGCAATCCAA GGACGCATAC CCAGACGGAA ACTAAGTTCC CACTCTCGAC "  
       TGTCCTCGAC TTATACGTTG TCGTTAGGTT "CCTGCGTATG GGTCTGCCTT TGATTCAAGG GTGAGAGCTG "  
  
000911 "C"CATGTAACA TGCTACACCA AGTAAGAAGT GTAGAACAAT TAGCTCATAA GGACCACCGT TGTATAA"CCA "  
       "GGTACATTGT ACGATGTGGT TCATTCTTCA" CATCTTGTTA ATCGAGTATT CCTGGTGGCA ACATATTGGT   
  
000981 "TTCATCAACG GAAGCAGCTT CCCAAATTGG GTAAAAGTGC AAACCGATAG CTGCAGAGGT AGGGATA"ATA   
       AAGTAGTTGC CTTCGTCGAA GGGTTTAACC CATTTTCACG TTTGGCTATC GACGTCTCCA TCCCTATTAT   
  
001051 GCACCAGAAA TAATATTGTT TCCGTAAAGT AGAGATCCAG AAACAGGTTC ACGAATACCA TCAAT"ATCCA "  
       CGTGGTCTTT ATTATAACAA AGGCATTTCA TCTCTAGGTC TTTGTCCAAG TGCTTATGGT AGTTATAGGT   
  
001121 "CTGGAGGAGC AGCAATGAAG GCAATAATAA ATACAGAAGT TGCGGTCAAT AAGGTAGGGA TCATC"AAAAC   
       GACCTCCTCG TCGTTACTTC CGTTATTATT TATGTCTTCA ACGCCAGTTA TTCCATCCCT AGTAGTTTTG   
  
001191 ACCAAACCAT CCAATGTAAA GACGGTTTTC AGTGCTGGTT ATCCAGTTAC AGAAACGACC CCATAGGCTT   
       TGGTTTGGTA GGTTACATTT "CTGCCAAAAG TCACGACCAA TAGGTCAATG TCTTTGCTGG GGTATCCGAA "  
  
001261 TCGCTTT"CGC GTCTCTCTAA AATTGCAGTC ATGGTAAAAT CTTGGTTTAT TTAATAATTA ATCATCAGGG "  
       "AGCGAAAGCG CAGAGAGATT" TTAACGTCAG TACCATTTTA GAACCAAATA AATTATTAAT TAGTAGTCCC   
  
001331 "ACTCCCA"AGC ACATGAATTC TCTAGAAATA GAAAATGAAG GGCTTGTTAT TCAACAGTAT AA"CATGACTT "  
       TGAGGGTTCG TGTACTT"AAG AGATCTTTAT CTTTTACTTC CCGAACAATA AGTTGTCATA TTGTACTGAA "  
  
001401 "ATATACCCAT GTCAACCAAT ATCAACATCC ATATGCTTTT ATTAGTTATC TAGATTAATC TT"TCTTTTTT   
       "TATATGGGTA CAGTTGG"TTA TAGTTGTAGG TATACGAAAA TAATCAATAG ATCTAATTAG AAAGAAAAAA   
  
001471 TTATTTAATT CTTTTCATTC ATTAAAAAAT CGAATTAGCC TTGATAGATT ATTTTGATAT GATAAATTAG   
       AATAAATTAA GAAAAGTAA"G TAATTTTTTA GCTTAATCGG AACTATCTAA TAAAACTATA CTATTTAATC "  
  
001541 TTCC"CTATCA TCATTAATAT GGGTTGCCCG GGACTCGAAC CCGGAACTAG TCGGATGGAG TCGATAATTT "  
       "AAGGGATAGT AGTAATTAT""A CCCAACGGGC CCTGAGCTTG GGCCTTGATC AGCC"TACCTC AGCTATTAAA   
  
001611 "ACTT"AAAAAA AAAATAAGTA AAAAAAATCC CTCCCCAAAC CGTGCTTGCA TTTTTCATTG CA"CACGGCTT "  
       TGAAT"TTTTT TTTTATTCAT TTTTTTTAGG GAGGGGTTTG GCACGAACGT AAAAAGTAAC GTGTGCCGAA "  
  
001681 "TACCTATGTA TACATCTAAA ACGCAGTTAC TTCCCTAGAC AGGATTCTAA CTAAGAAAGT GG"AATATTCT   
       "ATGGA"TACAT ATGTAGATTT TGCGTCAATG AAGGGATCTG TCCTAAGATT GATTCTTTCA CCTTATAAGA   
  
001751 GTTGATCAAC TCTTACCAAA TGAACATTTC ATAATAGAAA TTAATGAATT TTTTTAGTAT "CTCGTGAGGA "  
       CAAC"TAGTTG AGAATGGTTT ACTTGTAAAG TATTATCTTT AATTACTTAA AAAAATCATA GAGCACTCCT "  
  
001821 "TTTATTAAGT AAAATTCTCA TATACATGGT TTCATAACCA ATCGTTCATG ATTGGCCAAA" TCATTCATGC   
       "AAAT"AATTCA TTTTAAGAGT ATATGTACCA AAGTATTGGT TAGCAAGTAC TAACCGGTTT AGTAAGTACG   
  
001891 AAATAATATC CAAATACCAA ATTTTCCCCC TATATACCCT CCGTAAAGTA AAAGAAGTTC T"TGGGAAGAT "  
       TTTATTATAG GTTTATGGTT TAAAAGGGGG ATATATGGGA GGCATTTCAT TTTCTTCAAG AACCCTTCTA   
  
001961 "CAAAGAAAGA ACCTGTTCTT GCTCCGTAAA GAATTCTTCC AATAATTCCG AATTTAACCT T"TTAAAAAGA   
       GTTTCTTTCT TGGACAAGAA CGAGGCATTT CTTAAGAAGG TTATTAAGGC TTAAATTGGA AAATTTTTCT   
  
002031 GCACGGTACA GTACTTTTGT GTTTACGAGC CAAAGTTTTA ACACAAGAAA GGCGAAGTAT ATATT"GTATT "  
       CGTGCCATGT CATGAAAACA CAAATGCTCG GTTTCAAAAT TGTGTTCTTT CCGCTTCATA TATAACATAA   
  
002101 "CGATACAAAT TCTTTTTTTT TGAGGATCCT CTGTAATAAT GAGAAAGATT TCTGGATATA CGCAA"AAATC   
       GCTATGTTTA AGAAAAAAAA ACTCCTAGGA GACATTATTA CTCTT"TCTAA AGACCTATAT GCGTTTTTAG "  
  
002171 GATCAATAAT ATCAAAATCC GACGAATCGA TCCAGGTCGG CTTACTAATG GGATGTCTAA GACGTTAC"AA "  
       "CTAGTTATTA TAGTTTTAGG CTGCTTAGCT AGGTCCAGCC GAATG"ATTAC CCTACAGATT CTGCAATGTT   
  
002241 "AATTTCGCTT TAGCCAATGA TGCAAGCAGA GTAATAATAG GAACTAGTGT ATCGAGTTTC TGCGTAGC"AT   
       TTAAAGCGAA ATCGGTTACT ACGTTCGTCT CATTATTATC CTTGATCACA TAGCTCAAAG ACGCATCGTA   
  
002311 TATCTATTAG AAATGAATTT TCTAACATCT GAGTCCATAC CACTGAAGGA TTTAATCGCA CACTTGAAAT   
       ATAGATAATC TTTACTTAAA AGATT"GTAGA CTCAGGTATG GTGACTTCCT AAATTAGCGT GTGAACTTTA "  
  
002381 ATA"GCCCAAA AAGTCAAGAG AACGCTTGGA TAATTGGTTT ATATAGATCC TTTCTGGTTG TGACCACACA "  
       "TATCGGGTTT TTCAGTTCTC TTGCG"AACCT ATTAACCAAA TATATCTAGG AAAGACCAAC ACTGGTGTGT   
  
002451 "TAA"AAATGAC ATTGCCATAA ATTGACAAGG TAATATTTCC ACTTATTAAT CAGAAGTGGC GTATCCTTTG   
       ATTTTTACTG TAACGGTATT TAACTGTTCC ATTATAAAGG TGAATAATTA GTCTTCACCG CATAGGAAAC   
  
002521 AA"ACCAGAAT AGATTTTGCT TGATATCTAA CATAATGTAT TAAAGGATCC TTGAAGACCC GTAAGATAGC "  
       TTTGGTCTTA TCTAAAACGA ACTATAGATT GTATTACATA ATTTCCTAGG AACTTCTGGG CATTCTATCG   
  
002591 "CG"AAAAATAA TTAGCAAACA CTTTGACAAG ATGTTCGATT TTTCCATAGA AATATATTCG CTCAAAAA"GG "  
       GCTTTTTATT AATCGTTTGT GAAACTGTTC TACAAGCTAA AAAGGTATCT TTATATAAGC GAGTTTTTCC   
  
002661 "CCCTTATAAG AAGTTAATCG TATATGAGAA GATTGGTTAC GTAGAAAAAG GAAAATGGAT TCGTATCC"AC   
       GGGAATATTC TTCAATTAGC ATATACTCTT CTAACCAATG CATCTTTTTC CTTTTACCTA AGCATAGGTG   
  
002731 ATACATAAGA ATTGTATAGC AACAAGACTA ATCTTCGATT TCTTTTTGAA AAAAAAGAAA TCAATTTTTT   
       TATGTATTCT TAACATATCG TTGTTCTGAT TAGAAGCTAA AGAAAAACTT TTTTTTCTTT AGTTAAAAAA   
  
002801 TGAAGTACTA "AGACTATTCA AATTACAATA CTCGTGAAAA AAGAACCGTA ATAAATGAAA AGAAGAGGCA "  
       ACTTCATGAT TCTGATAAGT TTAATGTTAT GAGCACTTTT TTCTTGGCAT TATTTACTTT TCTTCTCCGT   
  
002871 "TCTTTCACCC" AGGAGCGAAG GATTTGAACT AAAATTTCCA GATGGAGCGG ATAGGGTATT AATACATCTG   
       AGAAAGTGGG TCCTCGCTTC CTAAACTTGA TTTTAAA"GGT CTACCTCGCC TATCCCATAA TTATGTAGAC "  
  
002941 ACACATAATT "TAAATGTGGG AATTTATCCT CTAAAAAAGG AAATATTGAA TGACTTGATC GTAAATTATA "  
       "TGTGTATTAA ATTTACACCC TTAAATAGGA GATTTTT"TCC TTTATAACTT ACTGAACTAG CATTTAATAT   
  
003011 "AGATTTTGCG" ATTTCTTCTT CTTCTAAGGA AGATACTAAT CGTAGGGAAA GTGGAATTTC "CGCAATGACT "  
       TCTAAAACGC TAAAGAAGAA GAAGATTCCT TCTATGATTA GCATCCCTTT CACCTTA"AAG GCGTTACTGA "  
  
003081 "GCAAACCCTT CTGATAACAT TTGAGAATAC AAATTGTTGT TGTACCCCCA AAAAGGGTTT" TTGTTATAAT   
       "CGTTTGGGAA GACTATTGTA AACTCTTATG TTTAACAACA ACATGGGGGT TTTTCCC"AAA AACAATATTA   
  
003151 AATTAGTGAA AAAAAAAAAA TGATTCTGGT GATACATTCG AGTAATTAAA TGTTTTACCA "TTAGTAAACT "  
       TTAATCACTT TTTTTTTTTT ACTAAGACCA CTATGTAAGC TCATTAATTT ACAAAATGGT AATCATTTGA   
  
003221 "GGATTTTTTG TCATAACCAG AATTTTCCAA CAAAATGGAT CCATTTAAAA AATGATCATG" AGAAAATGCG   
       CCTAAAAAAC AGTATTGGTC TTAAAAGGTT GTTTTACCTA GGTAAATTTT TTACTAGTAC TCTTTTACGC   
  
003291 TAAATATACT CCCGAAAGAT AAGTGGGTAT AGGAAGTCAC GTTGCCGAGA TTTCTCAAGT TCTAAATAT"C "  
       ATTTATATGA GGGCTTTCTA TTCACCCATA TCCTTCAGTG CAACGGCTCT AAAGAGTTCA AGATTTATAG   
  
003361 "CTTGAAATTC CTCCATTTGA AATTTGATTT GAACTGGGGA TACTATAAAT GGATTTATTG GGTTATCAA"A   
       GAACTTTAAG GAGGTAAACT TTAAACTAAA CTTGACCCCT ATGATATTTA CCTAAATAAC CCAATAGTTT   
  
003431 TGATACATAG TGCGATACAG TCAAAACAAG GTATTAGAGT AAAATAAAGA ATAATAATAG "ATACCTCGTA "  
       ACTATGTATC ACGCTATGTC AGTTTTGTTC CATAATCTCA T"TTTATTTCT TATTATTATC TATGGAGCAT "  
  
003501 "AATAGGTAAG ACTTATCAAC GGACTCTCTA TCCTCTCTTT TCTTTTGTCA TCTAATTGGT" TTCTATTTTA   
       "TTATCCATTC TGAATAGTTG CCTGAGAGAT AGGAGAGAAA A"GAAAACAGT AGATTAACCA AAGATAAAAT   
  
003571 GAATAAAAAA GATGGTTAGA AATCCTTTAT TTTTTCAACC CAATCGCTCT TTTGATTTTG GAAAAAAACT   
       CTTATTTTTT CTACCAATCT TTAGGAAATA AAAAAGTTGG G"TTAGCGAGA AAACTAAAAC CTTTTTTTGA "  
  
003641 CTTTA"TCAAT ATACTGCTTC TTCTACACAT TCCCCTCTAC CTCATAACGT AGAGTAACTA ATAGTTAGGA "  
       "GAAATAGTTA TATGACGAAG AAGATGTGTA AGGGGAGATG G"AGTATTGCA TCTCATTGAT TATCAATCCT   
  
003711 "CTTAG"AAAAA AATGGATAAC TCACTCATAA GAAAAGTCCT TCCCGCATCA AGCACTAATA T"AGTTTTAAC "  
       GAATCTTTTT TTACCTATTG AGTGAGTATT CT"TTTCAGGA AGGGCGTAGT TCGTGATTAT ATCAAAATTG "  
  
003781 "GTCTAATTAG ATCGGGGAAT GATTCAAATT AAGAACATAA GCCCGTTACT TTTTATTTCT C"TATAATTGG   
       "CAGATTAATC TAGCCCCTTA CTAAGTTTAA TT"CTTGTATT CGGGCAATGA AAAATAAAGA GATATTAACC   
  
003851 AGCATAGTGC CCTATCCATT TATTGATTCG ACCCGACTTT ACTTTTTTTT TCCGCATCAC GAATTAAAAC   
       TCGTATCACG GGATAGGTAA ATAAC"TAAGC TGGGCTGAAA TGAAAAAAAA AGGCGTAGTG CTTAATTTTG "  
  
003921 AAGGTT"TGGC CTAATCTAGT AAGGTAAAAA TATTACTAGA ATTTTCCATT GATACGACAT GCTGCTTTTT "  
       "TTCCAAACCG GATTAGATCA TTCCA"TTTTT ATAATGATCT TAAAAGGTAA CTATGCTGTA CGACGAAAAA   
  
003991 "CCATTC"ATTC CTTTCAGGAT CAGTCGCGGT CTTACAAACT CTACCGATAG TATGGACGAA TCTGTTAC"TT "  
       GGTAAGTAAG GAAAGTCCTA GTCAGCG"CCA GAATGTTTGA GATGGCTATC ATACCTGCTT AGACAATGAA "  
  
004061 "CATAGAAATG TGTAAAAGAT GCTAGCCGCA CTTAAAAGCC GAGTACTCTA CCATTGAGTT AGCAACCC"AA   
       "GTATCTTTAC ACATTTTCTA CGATCGG"CGT G"AATTTTCGG CTCATGAGAT GGTAACTCAA TCGTTGGG"TT   
  
004131 AAATATGCAA AATTTTTCTG TGTAGATACA ATCGGAATCA AAATAACAAA AGAAATTAAA TT"ACATGACG "  
       TTTATACGTT TTAAAAAGAC "ACATCTATGT TAGCCTTAGT TTTATTGTTT TCTTTAATTT AATGTACTGC "  
  
004201 "TAATCAAAAT ATTCCAATAA AAAAAAAATC ATACAAATAT TACACAAAAG TAATTTTTTG TA"TCACAAAA   
       "ATTAGTTTTA TAAGGTTATT" TTTTTTTTAG TATGTTTATA ATGTGTTTTC ATTAAAAAAC ATAGTGTTTT   
  
004271 AATACAATGA GACCATCATG TGCGTGAAAA GCAAAGGTCA TGAAAGGGAG ATAACTAGCT TCATTTATAC   
       TTATGTTACT CTGGTAGTAC ACGCACTT"TT CGTTTCCAGT ACTTTCCCTC TATTGATCGA AGTAAATATG "  
  
004341 A"CGATCGGAT TATATTTATT TGATACACTG TTGTCAATAT ATGAATGTGA ATAATGAAAA ACGCCTATAA "  
       "TGCTAGCCTA ATATAAATAA ACTATGTG"AC AACAGTTATA TACTTACACT TATTACTTTT TGCGGATATT   
  
004411 "T"GGACAAAAC AAAAGAATTA GAAAAAAATT ATATAACGAA TAAAAAACAA ATGGAAATAA CAATTTGAGT   
       ACCTGTTTTG TTTTCTTAAT "CTTTTTTTAA TATATTGCTT ATTTTTTGTT TACCTTTATT GTTAAACTCA "  
  
004481 TGAATAACTA "TATTTATTTT ATATATATAA AATAAATATA GTTATATTTA ACTAGATAAA GCTAACAAAA "  
       "ACTTATTGAT ATAAATAAAA" TATATATATT TTATTTATAT CAATATAAAT TGATCTATTT CGATTGTTTT   
  
004551 "TAGAATTAAG" AGAAAATATT CTAATAATAA TCAATTATAT TAAAATAATA GAAAAGAATT AAAAA"AAACT "  
       ATCTTAATTC TCTTTTATAA GATTATTATT "AGTTAATATA ATTTTATTAT CTTTTCTTAA TTTTTTTTGA "  
  
004621 "ACGGACTTGG ATTGGATTGG CACTCTAGAG ATAATCAACA TAGATAGACA TAAAAGATGT AGGCG"AAAGA   
       "TGCCTGAACC TAACCTAACC GTGAGATCTC" TATTAGTTGT ATCTATCTGT ATTTTCTACA TCCGCTTTCT   
  
004691 AGAAATTAAG GAAGAAGTAG AAAAAAATAT ATCGGGTTGA TTCAATCCAT AAATAATAAG TAAAA"AAACT "  
       TCTTTAATTC CTTCTTCATC TTTTTTT"ATA TAGCCCAACT AAGTTAGGTA TTTATTATTC ATTTTTTTGA "  
  
004761 "TAACCCCCCC TTTTTATTTA TTCATAAAAT TGCAACAAAA TCAACCGAGG TAATGTACAA ATTTG"ATATA   
       "ATTGGGGGGG AAAAATAAAT AAGTATT"TTA ACGTTGTTTT AGTTGGCTCC ATTACATGTT TAAACTATAT   
  
004831 AAATATATCG TCTAGAACCA ATTAGTTCAT TTAGTCACTT GATTTATTTT TTTTTCTATT "CATCTTAGAT "  
       TTTATATAGC AGATCTTGGT TAA"TCAAGTA AATCAGTGAA CTAAATAAAA AAAAAGATAA GTAGAATCTA "  
  
004901 "CAATTTATAT CAATAAAATC AAAATAGATT TTTTTCATTA TCGAAGACGG AATTTTAAGG" TAATAAGTGT   
       "GTTAAATATA GTTATTTTAG TTT"TATCTAA AAAAAGTAAT AGCTTCTGCC TTAAAATTCC ATTATTCACA   
  
004971 ATAGGATGAA TAGAACAAGA GAGGGGGGAA ATACTAAAGA AAAGGTTAGT CAAAGCTATA TACAAGTTAA   
       TATCCTACTT ATCTTGTTCT CTCCCCCCTT TATGATTTCT "TTTCCAATCA GTTTCGATAT ATGTTCAATT "  
  
005041 GTTATCCA"CA CCCTCGTTTA TTAATATTAA TGGAATTTCG GTTATTGATT AAGGTGAAGT TCCGTAAAAA "  
       "CAATAGGTGT GGGAGCAAAT AATTATAATT ACCTTAAAGC" CAATAACTAA TTCCACTT"CA AGGCATTTTT "  
  
005111 "CACCTGCC"TT CTTTAAAATA TCATGAACAG TTCCTGTAGG TTGAGCACCC TTTTCAAGGA AATAT"AGAAT "  
       "GTGGACGGAA GAAATTTTAT AGTACTTGTC AAGGACATCC AACTCGTGGG AAAAGTTC"CT TTATATCTTA   
  
005181 "CGCAGGAACA TTTAAATAGG TTTGATTCTT TATCGGATCA TAAAAACCCA CTTTACGAAG ATCTC"TTCCT   
       GCGTCCTTGT AAATTTATCC AAACTAAGAA ATAGCCTAGT ATTT"TTGGGT GAAATGCTTC TAGAGAAGGA "  
  
005251 TCTCTTCGGG ATCGAACATC AATTGCAATG ATTCGATAAA CAGCTCATTG GGATAGATGT A"AATTAACAA "  
       "AGAGAAGCCC TAGCTTGTAG TTAACGTTAC TAAGCTATTT GTCG"AGTAAC CCTATCTACA TTTAATTGTT   
  
005321 "TACCCCCCCC AGAACCGTAT AAGAAGTTTT CTCCTCGTAC GGCTCGAGGA AATTCAAAGT T"ATGTATAGA   
       ATGGGGGGGG TCTTGGCATA TTCTTCAAAA GAGGA"GCATG CCGAGCTCCT TTAAGTTTCA ATACATATCT "  
  
005391 ATTCTAATTA ATGTCAAATA GATCCATAGA TTATTAAATC AATTAGACTA TGATTTAAAT AATTTTTTCT   
       "TAAGATTAAT TACAGTTTAT CTAGGTATCT AATAA"TTTAG TTAATCTGAT ACTAAATTTA TTAAAAAAGA   
  
005461 TATTCTTT"TG TTTTTCTTTT GAAAAAAAAA CTCATTCTTA TCCCCATAAC TCAAGTTGGA TAACTCTCAC "  
       ATAAGAAAAC AAAAAGAAAA CTTTTTTTTT GAGTAAGAAT AGGGGTATT"G AGTTCAACCT ATTGAGAGTG "  
  
005531 "GCAAAGGA"AA AAAACCTTAA GCTTAAGCAT TTCATTTATT GAGCGGTCTC TAACCTCTTT AT"TTTTGCCT "  
       "CGTTTCCTTT TTTTGGAATT CGAATTCGTA AAGTAAATAA CTCGCCAGA"G ATTGGAGAAA TAAAAACGGA   
  
005601 "GTCTCCGTTA GAATCTATTT CGATTCTTCA TTCTGATCTA GTTTAGTTAT TGAGACAATT GA"AAAAGATT   
       CAGAGGCAAT CTTAGATAAA GCTAAGAAGT AAGACTAGAT CAAATCAATA A"CTCTGTTAA CTTTTTCTAA "  
  
005671 TTTACTTGTT CCGGGATCCT TTATCCTTGC CTTGAATCAT TGGGTTTAGA CATTACTTCG GTGATCTTTA   
       "AAATGAACAA GGCCCTAGGA AATAGGAACG GAACTTAGTA ACCCAAATCT G"TAATGAAGC CACTAGAAAT   
  
005741 ATCGTTTCA"A AATGGTAGCA ACATACCATT TTTTTGTGAT TTCTTTTTAT CAAAGAATCA TATAAATAAT "  
       TAGCAAAGTT TTACCATCGT TGTATGGTAA AAAAACACTA AAGAAAAATA G"TTTCTTAGT ATATTTATTA "  
  
005811 "GGATTCTCG"T GTGATACACT TTTGATCAAA TTTTATGTTT GAATTTGAAA CTTGTTCGAA TTGGATCCTT   
       "CCTAAGAGCA CACTATGTGA AAACTAGTTT AAAATACAAA CTTAAACTTT G"AACAAGCTT AACCTAGGAA   
  
005881 TCGATT"TGTA TACCGAACCT ATACTTACGA AGTAGTTTTA ACTTATTGAT TGACACTAAC CCTAGATCTC "  
       AGCTAAACAT ATGGCTTGGA TATGAATGCT TCATCAAAAT TGAATAA"CTA ACTGTGATTG GGATCTAGAG "  
  
005951 "TGCCCT"TGAT AAATGAATCA ATACTTTCTA CTCGAGCTCC ATCATGTACT ATGTTACATC AAAACCCTAA   
       "ACGGGAACTA TTTACTTAGT TATGAAAGAT GAGCTCGAGG TAGTACA"TGA TACAATGTAG TTTTGGGATT   
  
006021 AAAAAA"CAGA GCTCAGTGCA ACCGAACAAA CTATGTCGAG TCAAGAGCAT CTTCATTCCT AAAAATGGTG "  
       TTTTTTGTCT CGAGTCACGT TGGCTTGTTT GATACAGCTC AGTTCTCGTA G"AAGTAAGGA TTTTTACCAC "  
  
006091 "GGTGTA"AGAA TCCACAGTGG ATCATGTCCT TCAAGTCGCA GCGTATAGTA CGCGAGGTCG AGTCTGCAG"T "  
       "CCACATTCTT AGGTGTCACC TAGTACAGGA AGTTCAGCGT CGCATATCAT G"CGCTCCAGC TCAGACGTCA   
  
006161 "CCTCCGTAGC ATGAGCTCTT CAGAGTGCGC ACGTTGCTTT CTACCACATC GTTTTAAACG AAGTTTTAC"C   
       GGAGGCATCG TACTCGAGAA GTCTCACGCG TG"CAACGAAA GATGGTGTAG CAAAATTTGC TTCAAAATGG "  
  
006231 ATAACCTCTA ATTTCTTGGA ACTGGGATGT AATTGATTCA TTTATGGAAT CATGTATAGT CATTGG"GTTA "  
       "TA"TTGGAGAT TAAAGAA"CCT TGACCCTACA TTAACTAAGT AAATACCTTA GTACATATCA GTAACCCAAT "  
  
006301 "GTTGGTCCAT AGTAATCTAT ACTCTTATGA CATGGGTAGT TTTTGAAAAA GTCTTAGCAA TACTTC"AATT   
       "CAACCAGGTA TCATTAG"ATA TGAGAATACT GTACCCATCA AAAACTTTTT CAGAATCGTT ATGAAGTTAA   
  
006371 TATTTAAACC CATATTTTAT TGTATATATT GGATCAATAA CATTTTTTTT GTATGAGTGC AATATTTAT"T "  
       ATAAATTT"GG GTATAAAATA ACATATATAA CCTAGTTATT GTAAAAAAAA CATACTCACG TTATAAATAA "  
  
006441 "TCTCTATATA TAGATATTTT CTATATAATA GAAATATGGA GAGATTTTTG GGAATTTCTA TAAAATAAA"T   
       "AGAGATAT"AT ATCTATAAAA GATATATTAT CTTTATACCT CTCTAAAAAC CCTTAAAGAT ATTTTATTTA   
  
006511 TAAGAAATAA TTAATTACGA ATAATTAAGA ATCTCCATTT TTCAATTTCT TCATAGAATG GATTC"ACCGA "  
       ATTCTTTAT"T AATTAATGCT TATTAATTCT TAGAGGTAAA AAGTTAAAGA AGTATCTTAC CTAAGTGGCT "  
  
006581 "GTTTCACACT TCTTCGAGTC CAAGGACTCA TAAGAAATCA TTAAAAAGAT TTACTTGATT GAAAT"TTACT   
       "CAAAGTGTG"A AGAAGCTCAG GTTCCTGAGT ATTCTTTAGT AATTTTTCTA AATGAACTAA CTTTAAATGA   
  
006651 ACCTCAATAT TATTATTTGA CTAAATTTTT GTAAAAAAAA AAAAAAGAAA TAAAACGATA AAAATAGATA   
       TGGAGTTATA ATAATA"AACT GATTTAAAAA CATTTTTTTT TTTTTTCTTT ATTTTGCTAT TTTTATCTAT "  
  
006721 ATAACAAT"AC ATACATATCA GCATTTTCTG CCCAGTTTAT TTAACTTAAG AGACTCAAGA ATCTTTCCCT "  
       "TATTGTTATG TATGTA"TAGT CGTAAAAGAC GGGTCAAATA AATTGAATTC TCTGAGTTCT TAGAAAGGGA   
  
006791 "AAAATCAA"AA ATAAAGTGAC AAAGATGTAT GAATTGTTAC TTGAATTTAC AATTATTCAT TACAGACAAA   
       TTTTAGTTTT TATTTCACTG T"TTCTACATA CTTAACAATG AACTTAAATG TTAATAAGTA ATGTCTGTTT "  
  
006861 CATACGAAAA "AATGAGGTGA AAAGAAATAC ATAATATGTT ACATATGTAA CTTGATTCTT TGTTAATAAG "  
       "GTATGCTTTT TTACTCCACT T"TTCTTTATG TATTATACAA TGTATACATT GAACTAAGAA ACAATTATTC   
  
006931 "ATTCGGGCAG" ATATTCAAAT TGATATCTTC CGTTATGGAT TACCTCCTAT CTACCCCCCC AA"TTAGATCG "  
       TAAGCCCGTC TATAAGTTT"A ACTATAGAAG GCAATACCTA ATGGAGGATA GATGGGGGGG TTAATCTAGC "  
  
007001 "AGTAGATGCA TCATAAATCA AAAAAGGATC CTACGGGGGA CTGGACTAGT TTCGGAGGTG CT"AGACTATC   
       "TCATCTACGT AGTATTTAG"T TTTTTCCTAG GATGCCCCCT GACCTGATCA AAGCCTCCAC GATCTGATAG   
  
007071 TTGTATAAGG CGAAAGTCAA ACAGATCCAG AGTATGTTCC TACCTATTTT TTTTATTTTA CTCTAAATTT   
       AACATATT"CC GCTTTCAGTT TGTCTAGGTC TCATACAAGG ATGGATAAAA AAAATAAAAT GAGATTTAAA "  
  
007141 GAGTAC"GCTA AATAGGTCTT AAGTTTCGTA AGACCATTGA TTGAATTCCA TTAGTGCCAA AAACACAAGA "  
       "CTCATGCG"AT TTATCCAGAA TTCAAAGCAT TCTGGTAACT AACTTAAGGT AATCACGGTT TTTGTGTTCT   
  
007211 "CCTTCG"TGTT TAAGAAATCC ACAGAAAAAA AATACTCGGG TTTCACACAC CATTTAAGAG ATAGAGA"ATA "  
       GGAAGCACAA ATTCTTTAGG T"GTCTTTTTT TTATGAGCCC AAAGTGTGTG GTAAATTCTC TATCTCTTAT "  
  
007281 "AGAGAGGCTT TCGCATTTCT TAAATGCATC ATTATAAGAA AATAATAAAG AACAATAACA TTCGATC"TAT   
       "TCTCTCCGAA AGCGTAAAGA A"TTTACGTAG TAATATTCTT TTATTATTTC TTGTTATTGT AAGCTAGATA   
  
007351 ATCTAGACTC TTAAGCATAA GGTTGGCAAT CTATAGTCTG ATATGATATC GAATATTTTT CCATATATCT   
       TAGATCTGAG AA"TTCGTATT CCAACCGTTA GATATCAGAC TATACTATAG CTTATAAAAA GGTATATAGA "  
  
007421 TA"TAATATAC TATTATTATA TATAAGACGC ATACTCTGGG ACGGAAGGAT TCGAACCTCC GAATAGCGGG "  
       "ATATTATATG AT"AATAATAT ATATTCTGCG TATGAGAC"CC TGCCTTCCTA AGCTTGGAGG CTTATCGCCC "  
  
007491 "AC"CAAAACCC GTTGCCTTAC CACTTGGCCA CGCCCCATTT ATATTCAACA CTAATAAACA CTAATATTGG   
       "TGGTTTTGGG CAACGGAATG GTGAACCGGT GCGGGGTA"AA TATAAGTTGT GATTATTTGT GATTATAACC   
  
007561 TA"GTGGTTAG TCGTCAATTC CAGTACAAAT ATTTATAGAA AAAATTAGAT TGTTGCTCGG ATTTTGATAC "  
       ATCACCAATC AGCAGTTAAG GTCATGTTTA TAAATATCTT TTTTAATCTA ACAACGAGCC TAAAACTATG   
  
007631 "GT"TTATAGAT CCAATTAAAC TAAATTTATT GATCATTACA TATAATTCCA TTAAGATATT GTATGAAAGT   
       CAAATATCTA GGTT"AATTTG ATTTAAATAA CTAGTAATGT ATATTAAGGT AATTCTATAA CATACTTTCA "  
  
007701 AGGATT"TCTT CGATTCTCGT TTTATTTGAG AATTGAAGGA TTTTTGATTG GCTGAGTTCA AATCAAAGAA "  
       "TCCTAAAGAA GCTA"AGAGCA AAATAAACTC TTAACTTCCT AAAAACTAAC CGACTCAAGT TTAGTT"TCTT "  
  
007771 "AGGTTT"TTTG ACCTACTTTA TGTTATTAAT TTTTCCCTTA TCACTTATAT CATAGGAATA ATAGGGGATT   
       "TCCAAAAAAC TGGATGAAAT ACAATAATTA AAAAGGGAAT AGTGAATATA GTATCCTTAT TATCCC"CTAA   
  
007841 AATAACTCAA TCAAATCAAA AGAAATACAA TTATCTTCAA GAACAAAGAA AAAAAAAAAA AAATTGTTAT   
       TTATTGAGTT AGTTTAGTTT TCTTTATGTT AATAGAAGTT CTTGTTTCTT TTTTTTTTTT TTTAACAATA   
  
007911 GCTTAATATC TTAAGTT"TCA TCGGTATCTG TCTTAATTCT TTCCTTTATT CAAGTAGTTT TTTCGTCGCC "  
       CGAATTATA"G AATTCAAAGT AGCCATAGAC AGAATTAAGA AAGGAAATAA GTTCATCAAA AAAGCAGCGG "  
  
007981 "AAATTGCCCG AGGCCTA"CGC TTTTTTGAAT CCAATAG"TAG ATGTTATGCC AGTAATACCT CTATTCTTTT "  
       "TTTAACGGG"C TCCGGATGCG AAAAAACTTA GGTTATCATC TACAATACGG TCATTATGGA GATAAGAAAA   
  
008051 "TTCTATTAGC TTTTGTTTGG CAAGCTGCTG TAAGCTT"TCG ATAATATTTT TAATACTGTC CGAGACAAAT   
       AAGATAATCG AAAA"CAAACC GTTCGACGAC ATTCGAAAGC TATTATAAAA ATTATGACAG GCTCTGTTTA "  
  
008121 TCATGATTTA CTAGGAAAAA AAAAAAAAAA GATTCTAA"CT AGTTATAAGA TCAGATAAGT TGTACATACA "  
       "AGTACTAAAT GATC"CTTTTT TTTTTTTTTT CTAAGATTGA TCAATATTCT AGTCTATTCA ACATGTATGT   
  
008191 "GTCTGAAGCT TTAAGTTGAA TCCAATATAG AAATCCTT"CT ATAGCTTTGA TAAATCTGGA TCACTCTCAT   
       "CAGACTTCGA AATTCAACTT AGGTTATATC TTTAGGAAGA TATCGAAACT ATTTAGACCT AGTGAGAGTA"   
  
008261 TTTATTATTC TTACTTTTTT C"CTTTGAACG ACCCTGTTAG AGTCCCCCAC AATATAGAAT TGTGGGTATG "  
       AAATAATAAG AATGAAAAAA GGAAACTTGC TGGGACAATC TCAGGGGGTG TTATATCTTA ACACCCATAC   
  
008331 "AAATCCAATT TTGAGTAATC A"AGGACTCAA CTCTTTAAAT TTTTTCCTAT TTCTAGAAAT AACTTCACTG   
       T"TTAGGTTAA AACTCATTAG TTCCTGAGTT GAGAAATTTA AAAAAGGATA AAGATCTTTA TTGAAGTGAC "  
  
008401 CATTTCTTGG TGTCAAAACA GATTAAAATA GAGAATCTAT TCTCTTAAAA AAAAAATGAT CTTGGAGATT   
       "G"TAAAGAACC ACAGTTTTGT CTAATTTTAT CTCTTAGATA AGAGAATTTT TTTTTTACTA GAACCTCTAA   
  
008471 GTGTAATGCT TACTCTCAAA CTATTTGTTT ATA"CAGTAGT AATATTCTTT GTTTCTCTCT TCATTTTCGG "  
       CACATTA"CGA ATGAGAGTTT GATAAACAAA TATGTCATCA TTATAAGAAA CAAAGAGAGA AGTAAAAGCC "  
  
008541 "ATTCCTATCT AATGACCCGG GACGTAATCC GGG"GCGTGAA GAATAAAAAA ATCAGATTTT TTTTCCTT"GC "  
       "TAAGGAT"AGA TTACTGGGCC CTGCATTAGG CCCCGCACTT CTTATTTTTT TAGTCTAAAA AAAAGGAA"CG "  
  
008611 "TTGATTTTGA AATGTTCTTA ACATTTTATC TATTCCACAC CTTTCCTCAA CTATAAAAAA ATACCAAG"TA   
       "AACTAAAACT TTACAAGAAT TGTAAAATAG ATAAGGTGTG GAAAGGAGTT GATATTTTTT TATGGTTC"AT   
  
008681 ATTGACAGAA ACGGAAAGA"G AGGGATTCGA ACCCTCGGTA CAAAAAAATC GTACAACGGA TTAGCAATCC "  
       TAACTGTCTT TGCCTTTCTC TCCCTAAGCT TGGGAGCCAT GTTTTTTTAG CATGTTGCCT AATCGT"TAGG "  
  
008751 "GACGCTTTAG TCCACTCAG"C CATCTCTCCC CAAATGGAAA ACGGATAATT ACTATGATAC ATTGTATAAA   
       "CTGCGAAATC AGGTGAGTCG GTAGAGAGGG GTTTACCTTT TGCCTATTAA TGATACTATG TAACAT"ATTT   
  
008821 AAATAGAAAA T"AAAGGGCTT GAAAAAAGCC CTTCTTTATC TCCCCCCTTT ATTATATAGA TATATAATTA "  
       TTTATCTTTT ATTTCCCGAA CTTTTTTCGG GAAGAAATAG AGGGGGGAAA TAATATATCT ATATATTAAT   
  
008891 "TATTGATATA G"ATAATTATT TATATTGATA TAGATAATTA TCTAGATATA TCGATGGATA TCTATAAAAT   
       ATAACTATAT CTAT"TAATAA ATATAACTAT ATCTATTAAT AGATCTATAT AGCTACCTAT AGATATTTTA "  
  
008961 CGATAAAAAC TTTT"TTTATT AGGAATTCCA TTTAGATAAA TTAGATTTGA TTAGATAAGG GCTCAGCTCA "  
       "GCTATTTTTG AAAA"AAATAA TCCTTAAGGT AAATCTATTT AATCTAAACT AATCTATTCC CGAG"TCGAGT "  
  
009031 "AAAGAAGAGA TATC"TTCAAT CCTAATATAT AAATAATACC AATATATTAA AGATTCATAA AAATATTTAT   
       "TTTCTTCTCT ATAGAAGTTA GGATTATATA TTTATTATGG TTATATAATT TCTAAGTATT TTTA"TAAATA   
  
009101 AAATAAAAAG AAAGTACCTT TTTTATTT"TA TCCGAAAAGT CCTTTTCTTT TTATTTTTAG TTCCACGGCC "  
       TTTATTTTTC TTTCATGGAA AAAATAAAAT AGGCTTTTCA GGAAAAGAAA AATAAAAATC AAGGT"GCCGG "  
  
009171 "TGGCCTGGTC AGTACCTAGC CGGGCTTT"TT TTGTTCCAAT GAATCGTAGA TCAAATTATT TATTTGATTT   
       "ACCGGACCAG TCATGGATCG GCCCGAAAAA AACAAGGTTA CTTAGCATCT AGTTTAATAA ATAAA"CTAAA   
  
009241 GAAAATGTTT TTGAAAAAAA AAAAAAAAA"T AGAAACAACA AGAATCCTAA GAAGATTTAT TATTTCAATT "  
       CTTTTACAAA AACTTTTTTT TTTTTTTTTA TCTTTGTTGT TCTTAGGATT CTTCTAAATA ATAAAGTTAA   
  
009311 "CCTATGATAC AGAAAAAGAT GATATAGAA"T CAAGGTTCGA TTCCTTATTA TTATTCTTTA GTACTTTACT   
       "GGATACTATG TCTTTTTCTA CTATATCTTA GTTCCAAGCT AAGGAATAAT AATAAGAAAT CATGAAATGA"   
  
009381 AGAATAAAAA ACTTGTTAAT TAGTTA"ATTA AAATGAGTCC TCTTTTTAAT ACGACTTATT TTCGTCTTCG "  
       TCTTATTTTT TGAACAATTA ATCAATTAAT TTTACTCAGG AGAAAAATTA TGCTGAATAA AAGCAGAAG"C "  
  
009451 "ACAAAAGGTC CATTTATATG CAATAA"TTGC ATTGTA"GCGG ATATAGTTTA GTGGTAAAA"G TGCGATTCGT   
       "TGTTTTCCAG GTAAATATAC GTTATTAACG TAACATCGCC TATATCAAAT CACCATTTTC ACGCTAAGC"A   
  
009521 TCTATTAATC CCTTAATAGT TAAGGGATCC TTCGGGTTGA TTAATAT"TCC GATCAAAAAC TTTATTTCTT "  
       AGATAATTAG GGAATTATCA ATTCCCTAGG AAGCCCAACT AATTATAAGG CTAGTTTTTG AAATAAAGAA   
  
009591 "AAAAGGATTT AATCCTTTAC CTCTCGATGA AAGATTCGAG GAAAAAT"AAA AATTCTCGTG ATTTGTATCC   
       TTTTCCTAAA TT"AGGAAATG GAGAGCTACT TTCTAAGCTC CTTTTTATTT TTAAGAGCAC TAAACATAGG "  
  
009661 AAAAATCTGT TCTAAATTGA CAAAATTGGA TCATGAAATT ACGAAACATA A"TTTTATTGA ATTGGATCAA "  
       "TTTTTAGACA AG"ATTTAACT GTTTTAACCT AGTACTTTAA TGCTTTGTAT TAAAATAACT TAACCTA"GTT "  
  
009731 "TACTTCCAAT TGAAGGAATA AGAGATCCAT GGATAAAGGT GGAATTTTTT C"TAATCGTAA CTAAATCTTC   
       "ATGAAGGTTA ACTTCCTTAT TCTCTAGGTA CCTATTTCCA CCTTAAAAAA GATTAGCATT GATTTAG"AAG   
  
009801 AATTTTTTCT TTGTATAAAG GAGATTGAAG CAAAACAAAA TAGCTATTA"A ACAATTTCTT TGGTTTACTA "  
       TTAAAAAAGA AACATATTTC CTCTAACTTC GTTTTGTTTT ATCGATAATT TGTTAAAGAA ACCA"AATGAT "  
  
009871 "GAGACGTCGA CATCGTTTTT TAGCTCGGCG GAAACAAACT ACTTTTCCT"A AGGATTATAT TAAATAGAAA   
       "CTCTGCAGCT GTAGCAAAAA ATCGAGCCGC CTTTGTTTGA TGAAAAGGAT TCCTAATATA ATTT"ATCTTT   
  
009941 TAGGGAACGA AAATAATACT GGAAAGATTG TTATAATCTC CTCTTGTATA G"GGATCATCT ATAAAGCGGG "  
       ATCCCTTGCT TTTATTATGA CCTTTCTAAC AATATTAGAG GAGAACATAT CCCTAGTAGA TATTT"CGCCC "  
  
010011 "TTCTTTTGAA TCATTCAGAC GGAAAGGCTG ACATAGATGT TATGGATGGC A"TTTTTTTTG TTTACATCTT   
       "AAGAAAACTT AGTAAGTCTG CCTTTCCGAC TGTATCTACA ATACCTACCG TAAAAAAAAC AAATG"TAGAA   
  
010081 CAATTTTTGA ATTTATCCAT CTTCCATAAA GGAGCCGAAT GA"AACCAAAG TTTCACGTTC GGTTTTGAAT "  
       GTTAAAAACT TAAATAGGTA GAAGGTATTT CCTCGGCTTA CTTTGGTTTC AAAGTGCAAG CCAAAACTTA   
  
010151 "TAGAGACGTT CAGTTCAAAA TGATGAATCA ACGTCGACTA TA"AC"CCCTAG CCTTCCAAGC TAACGATGCG "  
       ATCTC"TGCAA GTCAAGTTTT ACTACTTAGT TGCAGCTGAT ATTGGGGATC GGAAGGTTCG ATTGCTACGC "  
  
010221 "GGTTCGATTC CCGCTATCCG CT"TATCTTAT TCTTATCTTA TAATATATA"T ATTCTAAATA TAAATTAATT "  
       "CCAAG"CTAAG GGCGATAGGC GAATAGAATA AGAATAGAAT ATTATATATA TAAGATTTAT ATTTAATTAA   
  
010291 "AATTTATAAA TAGAAATTAA TTAATAATGG AATTACTCAA AAAAAAATT"T CAATTTACTT AAATTTCACT   
       TTAAATATTT AT"CTTTAATT AATTATTACC TTAATGAGTT TTTTTTTAAA GTTAAATGAA TTTAAAGTGA "  
  
010361 AAATTTTCAC TAATGTATTA GTTAATGTAA TGCAT"CATTG AATTGAATAT GCAATTTCCG GAATTCTCAC "  
       "TTTAAAAGTG AT"TACATAAT CAATTACATT ACGTAGTAAC TTAACTTATA CGTTAAAGGC CTTAAGAGTG   
  
010431 "ACATTTTTTT TACGAACAAG AGAGGTGAAA ATACG"AAAAA AAAATTGGAA TCAAAAGC"GT CCATTGTCTA "  
       TGTAA"AAAAA ATGCTTGTTC TCTCCACTTT TATGCTTTTT TTTTAACCTT AGTTTTCGCA GGTAACAGAT "  
  
010501 "ATGGATAGGA CAGAGGTCTT CTAAACCTTT GGTATAGGTT CAAATCCTAT TGGACGCA"AT TTTTTTCCAT   
       "TACCT"ATCCT GTCTCCAGAA GATTTGGAAA CCATATCCAA GTTTAGGATA ACCTGCGTTA AAAAAAGGTA   
  
010571 AGATTTTTTT GAGTTTGA"AA TTTTCTATAT CATGTCAAGA AAGGAAAGGT ATTTTGAATG ATTTGAATAC "  
       TCTAAAAAAA CT"CAAACTTT AAAAGATATA GTACAGTTCT TTCCTTTCCA TAAAACTTAC TAAACTTATG "  
  
010641 "GAGACGCTTA TTTATTAA"TA GTAATTTAGA AATTTGTTTT AATATTAATT GTAACTCTAG TTTTCGATTT   
       "CTCTGCGAAT AA"ATAATTAT CATTAAATCT TTAAACAAAA TTATAATTAA CATTGAGATC AAAAGCTAAA   
  
010711 ATTTATTATA TAAATAAACT AATAATTCAA ATA"TAAATAA ACTAATAATT CAATTCTGTT TTATTTGAAG "  
       TAAATAATAT AT"TTATTTGA TTATTAAGTT TATATTTATT TGATTATTAA GTTAAGACAA AATAAACTTC "  
  
010781 "ATAAGATATT AGTAGGAAGG ATATTCAAAT TCC"AAATATT AAGATATTTA TGATATTCTA AATATATTAT   
       "TATTCTATAA TC"ATCCTTCC TATAAGTTTA AGGTTTATAA TTCTATAAAT ACTATAAGAT TTATATAATA   
  
010851 TAGAAATATA ATCTAA"TTTT CACTTTATTT TTTTTTTATT AAAAAATTTA TTAATTGTAT TTACATTTCA "  
       ATCTTTATAT T"AGATTAAAA GTGAAATAAA AAAAAAATAA TTTTTTAAAT AATTAACATA AATGTAAAGT "  
  
010921 "TTTTCATATT GATATA"TATT TCTAGAAATA TTATCTACTT TCTTTAATAT ATTTATATAT ATTTAGTATT   
       "AAAAGTATAA C"TATATATAA AGATCTTTAT AATAGATGAA AGAAATTATA TAAATATATA TAAA"TCATAA "  
  
010991 TTATATATAT ATTCT"ACCTA CTAACACCTA ATAAATGAAA AAAATGAAAT TATTTCATGT TTCAATTTTA "  
       "AATATATATA TAAGATGGAT GATTGTGGAT TATTTACTTT TTTTACTTTA ATAAAGTACA AAGT"TAAAAT   
  
011061 "ACATTTTAAA GTTCT"AAAAT TTATTTATAC CTTACTTGTT TTCTTTTATA CTTGATCTTG AAGTAGAAAA   
       TGTAAAATTT CAAGATTTTA AATAAATATG GAATGAACAA AAGAAAATAT GAACTAGAAC TTCATCTTTT   
  
011131 CGTTCCATCT GTTC"CTGAAT AGCTTCTTTC AAAAGGACTT CCGCTTCAGG GGTGAATGTC TTCGTGGAAG "  
       GCAAGGTAGA CAAGGACTTA TCGAAGAAAG TTTTCCTGAA GGCGAAGTCC CCACTTACAG AAGCACCTTC   
  
011201 "AGATTATTTC TTGA"AACTGA GGTTTATTCG TTTTTACGTA AGTACGTAAC TCAACAAGAA ATTTCCTTAC   
       TCTAATAAAG AACTTTGACT CCAAATAAGC AAAAATGCAT TCATGCATTG AGTTGTTCTT TAAAGGAATG   
  
011271 TTGTGCAATT TCTAATGAA"T CAAGATAACC ATTAGTTCCG GTATAAACAG TCATTATCTG TTCATCTACC "  
       AACACGTTAA AGATTACTTA GTTCTATTGG TAATCAAGGC CATATTTGTC AGTAATAGAC AA"GTAGATGG "  
  
011341 "GTGAGAGGTG CTGATTGGG"A TTGTTTGAGC AACTCACGTA ATCGTTGACC TCTTGCCAAT TGATTCTGAG   
       "CACTCTCCAC GACTAACCCT AACAAACTCG TTGAGTGCAT TAGCAACTGG AGAACGGTTA AC"TAAGACTC   
  
011411 TAGCTTTATC GAGATCAGAA GCGAA"TTGCG CAAAGGCTTC TAATTCGGCA AATTGAGCCA ATTCCAATTT "  
       ATCGAAATAG CTCTAGTCTT CGCTTAACGC GTTTCCGAAG ATTAAGCCGT TTAACTCGGT TAAGGTTAAA   
  
011481 "TAATTTTCCG GCTACTTGTT TCATG"GCTTT AATTTGAGCT GCAGATCCTA CTCTGGAGAC GGAAATACCC   
       ATTAAAAGGC CGATGAACAA AGTACCGAAA TTAAACTCGA CGTCTAGGAT GAGACCTCTG CCTTTATGGG   
  
011551 ACATTAATAG CAGGTCTGAT TCCAGCATTG AATA"GATCGG CAGATAAGAA TATCTGTCCA TCGGTAATGG "  
       TGTAATTATC GTCCAGACTA AGGTCGTAAC TTATCTAGCC GTCTATTCTT ATAGACAGGT AGCCATTACC   
  
011621 "AAATTACATT AGTAGGAATA TAAGCTGAAA CATC"TCCCGA TTGGGTCTCA ACTATTGGTA AAGCAGTCAT   
       TTTAATGTAA TCATCCTTAT ATTCGACTTT GTAGAGGGCT AACCCAGAGT TGATAACCAT TTCGTCAGTA   
  
011691 ACTTCCTTCA CCTAAAGCAG AACTTAATTT A"GCGGCTCTT TCCAAAAGGC GTGAATGCAA ATAAAAAACA "  
       TGAAGGAAGT GGATTTCGTC TTGAATTAAA TCGCCGAGAA AGGTTTTCCG CACTTACGTT TATTTTTTGT   
  
011761 "TCTCCCGGAT AAGCTTCGCG GCCCGGCGGT C"TTCGTAAAA GAAGAGACAT TTGTCGATAA GCTTGTGCTT   
       AGAGGGCCTA TTCGAAGCG"C CGGGCCGCCA GAAGCATTTT CTTCTCTGTA AACAGCTATT CGAACACGAA "  
  
011831 GCTTGGAGGG ATCATCATAA ATATGAAGTG T"GCTGTTTTC TGTACATAAA ATATTCAGCC AAGCTGCTCC "  
       "CGAACCTCCC TAGTAGTAT"T TATACTTCAC ACGACAAAAG ACATGTATTT TATAAGTCGG TTCGACGAGG   
  
011901 "TGTATAAGGG GCAAGGTATT GTAATGTAGC C"GGAGAATCT GCCGTTTCGG CTACTATAAT AGTGTATTCC   
       ACATATTCCC CGTTCCATAA CATTACATCG GCCTCTTAGA CGGCAAAGCC GATGATATTA TCACATAAGG   
  
011971 ATTGCGCCCC TTTCCTGTAA AGTACTCACT ACTT"GAGCCA CAGAAGATGC TTTTTGACCA ATAGCTACAT "  
       TAACGCGGGG AAAGGACATT TCATGAGTGA TGAACTCGGT GTCTTCTACG AAAAACTGGT TATCGATGTA   
  
012041 "AAACACATAT TACATTTTGC CCTTGTTGAT TGAG"AATCGT ATCCGTGGCT ACTGCAGTTT TCCCGGTCTG   
       TTTGTGTATA A"TGTAAAACG GGAACAACTA ACTCTTAGCA TAGGCACCGA TGACGTCAAA AGGGCCAGAC "  
  
012111 CCTGTCCCCA ATAATTAATT CTCGTTGACC A"CGTCCGATA GGAATCATCG AATCAATAGC AATAAGTCCT "  
       "GGACAGGGGT T"ATTAATTAA GAGCAACTGG TGCAGGCTAT CCTTAGTAGC TTAGTTATCG TTATTCAGGA   
  
012181 "GTTTGAAGAG GCTCGTATAC GGAACGTCTC A"AAATAATAC CAGGAGCGGG GGATTCGATT AACCGAGAGT   
       CAAACTTCTC CGAGCATATG CCTTGCAGAG TTTTATTATG GTCCTCGCCC CCTAAGCTAA TTGGCTCTCA   
  
012251 CAGAAGATGA AATTTCACCT CGGCCATCAA T"AGGTTTAGC TAGGGCATTT ATAACACGAC CTAAATAAGC "  
       GTCTTCTACT TTAAAGTGGA GCCGGTAGTT ATCCAAATCG ATCCCGTAAA TATTGTGCTG GATTTATTCG   
  
012321 "CTCGCTTACT GGTATCTGAA CAATTCTTCC C"GTTGCTTTT ACAGAACTTC CCTCCTGTAT CAGCAAACCA   
       GAGCGAA"TGA CCATAGACTT GTTAAGAAGG GCAACGAAAA TGTCTTGAAG GGAGGACATA GTCGTTTGGT "  
  
012391 TCACCCATTA ATACAACACC AACATTA"TTT GATTCTAAAT TGAGAGCTAT GCCTATAGTA CCCTCTTCAA "  
       "AGTGGGT"AAT TATGTTGTGG TTGTAATAAA CTAAGATTTA ACTCTCGATA CGGATATCAT GGGAGAAGTT   
  
012461 "ATTCTACTAA TTCACCTGCC ATTACTT"CAT CAAGACCATA AATACGAGCA ATGCCGTCGC CTACTTGAAG   
       TAAGATGATT AAGTGGACGG TAATGAAGTA GTTCTGGTAT TTATGCTCGT TACGGCAGCG GATGAACTTC   
  
012531 TACAGTACCG GTATTTACAA TTTT"GACTTC TCTAGTATAT TGCTCAATAC GTTCACGGAT AATATTACTA "  
       ATGTCATGGC CATAAATGTT AAAACTGAAG AGATCATATA ACGAGTTATG CAAGTGCCTA TTATAATGAT   
  
012601 "ATTTCATCTG CTCGAATGGT TTCC"ATGAGT ATTTCGTAAT TCTTTTTTTT GAAGAAAAAA AATAATGCCT   
       TAAAGTAGAC GAGCTTACCA AAGGTACTCA TAAAGCATTA AGAAAAAAAA CTTCTTTTTT TTATTACGGA   
  
012671 ACAGTAGAAG GACTAATCAG TTAGTTATTT "CTTTCATCGT CCCAAACATG CCAATATTAG CACTGATGGT "  
       TGTCATCTTC CTGATTAGTC AATCAATAAA "GAAAGTAGCA GGGTTTGTAC GGTTATAATC GTGACTACCA "  
  
012741 "ACGTAAATGT AACTCGTTGT CCAAACAACT" ATTCAGAGTT CCTAGAGCTC CTTGTAAGGC TTGTTGGAAA   
       "TGCATTTACA TTGAGCAACA GGTTTGTTGA" TAAGTCTCAA GGATCTCGAG GAACA"TTCCG AACAACCTTT "  
  
012811 ACCTGTTGTC GTACTTGATT AAT"CGCTTTT TGTTGTTCAA AACGAATAGT TTCATTTTTA TAATTTTCTA "  
       "TGGACAACAG CATGAACTAA TTAGCGAAAA ACAACAAGTT TTGCTTATCA AAGTA"AAAAT ATTAAAAGAT   
  
012881 "ATTGTTCCAA ACTTTTAGAA GTT"GAATTAA TCAAATTCAA TTTTTCTCGT TCTATCTCAG AATATCCATT   
       TAACAAGGTT TGAAAATCTT CAACTTAATT AGTTTA"AGTT AAAAAGAGCA AGATAGAGTC TTATAGGTAA "  
  
012951 CACTCGAAAC TGATCCGCCT CC"ATTTCGAC TTTTCGTAAG CGAGCCCGGG CTTTTTCCAG CTGTTCAATG "  
       "GTGAGCTTTG ACTAGGCGGA GGTAAAGCTG AAAAGC"ATTC GCTCGGGCCC GAAAAAGG"TC GACAAGTTAC "  
  
013021 "GCCCCCCCAC GGAGTTCTTC TG"AATTTTGA ATTGTTTTCA AGATCCTCTG TTTTCGATTA TCTAATAAAT   
       "CGGGGGGGTG CCTCAAGAAG ACTTAAAACT TAACAAAAGT TCTAGGAGAC AAAAGCTA"AT AGATTATTTA   
  
013091 CACTTAATGA AAGTAGAT"TG TCTTTCCATT CATTTCAAAA CTTCGACGAT CCCTTCCCGA ACCAAACATG "  
       GTGAATTACT TTCATCTAAC AGAAAGGTAA GTAAAGTTTT GAAGCTGCTA GGGAAGGGCT TGGTTTGTAC   
  
013161 "AATCTTTCGA TTCATTTG"GC TCTCACGCTC AATTATTGCA ATTATTTCTG GGAAATTCCC ATATATCTTT   
       TTAGAAAGCT AAGTAAACCG AGA"GTGCGAG TTAATAACGT TAATAAAGAC CCTTTAAGGG TATATAGAAA "  
  
013231 TTGAATGTAA TGAGCCTA"TC CTCTTTTCTC TATTCATATT CCACAAAGAA AAATAAAAAA AAATATGGAT "  
       "AACTTACATT ACTCGGATAG GAG"AAAAGAG ATAAGTATAA GGTGTTTCTT TTTATTTTTT TTTATACCTA   
  
013301 "CATTAATCCA AGACCCGA"AT ATTCGGAGGA CTCTTCTGAC CAAACAAACA ATTGTCAGCA AAGTTGTTTC   
       GTAAT"TAGGT TCTGGGCTTA TAAGCCTCCT GAGAAGACTG GTTTGTTTGT TAACAGTCGT TTCAACAAAG "  
  
013371 TTTTTTTTTC TTGAA"ATCCA AAGAATTTTT CTTACTTTAT ACATAACATA GGTCATCGAT TCAGCATTCG "  
       "AAAAA"AAAAG AACTTTAGGT TTCTTAAAAA GAATGAAATA TGTATTGTAT CCAGTAGCTA AGTCGTAAGC   
  
013441 "ATAAAAAAGA AAAAA"ATTCT AATATTCTAT TAAAATAGAA AATAATAAAA AAAGGGGGGT TCAAATCATT   
       TATTTTTTCT TTTTTTAAGA TTATA"AGATA ATTTTATCTT TTATTATTTT TTTCCCCCCA AGTTTAGTAA "  
  
013511 TTATCGACAT GAGTGTTCTA TATCGAAAAA AAAT"CCCAAC TATTTGAAAC GATTTATGTA TTAACTATGT "  
       "AATAGCTGTA CTCACAAGAT ATAGC"TTTTT TTTAGGGTTG ATAAACTTTG CTAAATACAT AATTGATACA   
  
013581 "ATTAATATAG TAGTAGAAAG AGTACCATGC TACG"TCTGGA CTTCAAACGT TTTAACCATG TTAATAGTCC   
       TAATTATATC ATCAT"CTTTC TCATGGTACG ATGCAGACCT GAAGTTTGCA AAATTGGTAC AATTATCAGG "  
  
013651 CACATTATTG GGTTGATAGA GAATCAAAGT TGAT"TTACCA ATGAATCGCG AAATGCTATG GTTCTTCAAT "  
       "GTGTAATAAC CCAAC"TATCT CTTAGTTTCA ACTAAATGGT TACTTAGCGC TTTACGATAC CAAGAAGTTA   
  
013721 "ATGATTTCTT CATTTTGTCA GAAGTAATTC GCGG"GATCAT GCACCTTTTC GTAGTTATAA CTAGAAAAGT   
       TACTAAAGAA GTAAAACA"GT CTTCATTAAG CGCCCTAGTA CGTGGAAAAG CATCAATATT GATCTTTTCA "  
  
013791 ACAGTTGGTT GTATCCAACT TATTCTTGAA ATAA"ACAACT CGCACACACT CCCTTTCCAA AAAAAATCAA "  
       "TGTCAACCAA CATAGGTT"GA ATAAGAACTT TATTTGTTGA GCGTGTGTGA GGGAAAGGTT TTTTTTAGTT   
  
013861 "TACACCAAGC ACTACGCTTA GATTTATTGG ATTT"GTTGCT AAAATATCGG TATTAAATCC GAAACTCTCG   
       ATGT"GGTTCG TGATGCGAAT CTAAATAACC TAAACAACGA TTTTATAGCC ATAATTTAGG CTTTGAGAGC "  
  
013931 GCGGATGGCC AATAA"CCCAA AGAAATGAAA GAATCGGTTA CATTTTTCAT ATGATCTCCT TTTATAGATA "  
       "CGCC"TACCGG TTATTGGGTT TCTTTACTTT CTTAGCCAAT GTAAAAAGTA TACTAGAGGA AAATATCTAT   
  
014001 "AAACTAATTA TCTAT"TTCTT TCTATTTTTT TTATTTTTTA TTAATTTCCT ATTTCTATTC GAAATAGAGT   
       TTTGATTAAT AGATAAAGAA AGATAAAAAA AATAAAAAAT AATTAAAGGA TAAAGATAAG CTTTATCTCA   
  
014071 AAAAAATATG TTGTAAGAAT CCTAAAAAAA AA"AGTTCCAT TCGAGATTGG ACTAAGAAAG GGAAGGAAGA "  
       TTTTTTATAC AACATTCTTA GGATTTTTTT TT"TCAAGGTA AGCTCTAACC TGATTCTTTC CCTTCCTTCT "  
  
014141 "AGGCGAGTCA GTATGCTAAT GCCTCATCCT CA"AATCAATC CTTCCCATAA GTTATTGTCC CAACGAATAA   
       "TCCGCTCAGT CATACGATTA CGGAGTAGGA GT"TTAGTTAG GAAGGGTATT CAATAACAGG GTTGCTTATT   
  
014211 GTAATTGTCG GAGTGAA"AGC TTCATATAAT TCGAAAAAGC AGCAAGAGCA GCAAATCCAA GTAAATAAAA "  
       CATTAACAGC CTCACTTTCG A"AGTATATTA AGCTTTTTCG TCGTTCTCGT CGTTTAGGTT CATTTATTTT "  
  
014281 "TACGAAAAAA AATACGT"AAT TTTTTTAGGA TTAAACAAAA GGATTCGCAA ATAAAAGTGC TAATGCTACA   
       "ATGCTTTTTT TTATGCATTA A"AAAAATCCT AATTTGTTTT CCTAAGCGTT TATTTTCACG ATTACGATGT   
  
014351 ACCAGTCCAT AAATTGTTAA A"GCTTCCATA AAAGCCAGAC TAAGCAATAA AGTACCTCGG ATTTTTCCCT "  
       "TGGTCAGGTA TTTAACAATT TCGAAGGTAT TTTCGGTCTG ATTCGTTATT TCATGGAGCC TAAAAAGGGA"   
  
014421 "CCGCCTCGGG TTGTCTCGCG A"TCCCTTCTA CAGCTTGGCC CGCAGCAGTA CCTTGACCAA TCCCAGGTCC   
       GGCGGAGCCC AACAGAGCGC TAGGGAAGAT GTCGAACCGG GCGTCGTC"AT GGAACTGGTT AGGGTCCAGG "  
  
014491 AATAGAAGCA AGCC"CCACGG CCAATCCAGC AGCAATAACG GAAGCGGCAG AAATCAGTGG ATTCATGATA "  
       "TTATCTTCGT TCGGGGTGCC GGTTAGGTCG TCGTTATTGC CTTCGCCG"TC TTTAGTCACC TAAGTACTAT   
  
014561 "AGTTCCTCAT ACTA"AAATAA AAGAAAGAAA TGGTTAATGA TACGATGAAC CAATGAATTA TAACTTATTA   
       TCAAGGAGTA TGATTTTATT TTCTTTCTTT ACCAATT"ACT ATGCTACTTG GTTACTTAAT ATTGAATAAT "  
  
014631 TTCCATCACT AAGATTT"ATC CAACTAAAAA TTACGAATTG AAGTAATAAT ATTATTGAAT CGTTCAAACT "  
       "AAGGTAGTGA TTCTAAATAG GTTGATTTTT AATGCTT"AAC TTCATTATTA TAATAACTTA GCAAGTTTGA   
  
014701 "ACTTCAATCT CTCTTTT"TTA GTTCCTATCC ATCCACGTCC TTTTTGTGAA TCCATACGAC TTTTGTTCTT   
       TGAAGTTAGA GAGAAAAAAT CAAGGA"TAGG TAGGTGCAGG AAAAACACTT AGGTATGCTG AAAACAAGAA "  
  
014771 CCATTTCTTG ATTTTTGCTA AAGCATTCTT TGA"ATTCTTC ATTGTTTTTC TTGATTTAAT CTCTCTATTC "  
       "GGTAAAGAAC TAAAAACGAT TTCGTA"AGAA ACTTAAGAAG TAACAAAAAG AACTAAATTA GAGAGATAAG   
  
014841 "ATTCAATATT GAATTCAAAA TTACATTACA GCC"GAAAGAG AAGGACTTCC ATTGTAAGCC CTATCTAAAT   
       TAAGTTATAA CTTAAGTTTT AATGT"AATGT CGGCTTTCTC TTCCTGAAGG TAACATTCGG GATAGATTTA "  
  
014911 TCACAGTAGT AGGGCGGATT AGATATAT"CT TTAGTTCCTA TATAACTAGT TAATATCTAA TATAACATAT "  
       "AGTGTCATCA TCCCGCCTAA TCTAT"ATAGA AATCAAGGAT ATATTGATCA ATTATAGATT ATATTGTATA   
  
014981 "ACATGTGTTT CTTCCCTAAC GTCAACCA"AT TATTCATATC TTGGATTCCA TCGAATTTGA GAATCATTCT   
       TGTACACAAA GAAGGGAT"TG CAGTTGGTTA ATAAGTATAG AACCTAAGGT AGCTTAAACT CTTAGTAAGA "  
  
015051 TCGAAATATC CACA"AGGGTT GTCTTATAGC AATTCGATTC AATACATCTA GTTGACTCCA CTCTCCTCTA "  
       "AGCTTTATAG GTGTTCCC"AA CAGAATATCG TTAAGCTAAG TTATGTAGAT CAACTGAGGT GAGAGGAGAT   
  
015121 "CTCTACCCTA CCCA"ATCCTT TCCTTTTTTC TCGTTTTTTG TAAACCCTTT CTTTTTAGAA TTATCCCACA   
       GAGATGGGAT GGGTTAGGAA AGGAAAAAAG AGCAAAA"AAC ATTTGGGAAA GAAAAATCTT AATAGGGTGT "  
  
015191 TGGAGACAAT CAATCTAA"AT GAACGAATTT ATTAGTCCGA ATCCTTGCAT AGAAATATCA ATATTTTATT "  
       "ACCTCTGTTA GTTAGATTTA CTTGCTTAAA TAATCAG"GCT TAGGAACGTA TCTTTATAGT TATAAAATAA   
  
015261 "AATCTAAGTT CATGTAAT"TT TTTTTGTTCA ATCGTTGAGA GGAAAAAGAT CTTTTAGATC TCTTTCCTTT   
       TTAGATTCAA GTACATTAAA AAAAAC"AAGT TAGCAACTCT CCTTTTTCTA GAAAATCTAG AGAAAGGAAA "  
  
015331 TTTTACAATT CCCGAATATC GTAATCTTTC TAG"TCTTACT CCCTAGATAG TATATACCTT CCTTATTTTT "  
       "AAAATGTTAA GGGCTTATAG CATTAG"AAAG ATCAGAATGA GGGATCTATC ATATATGGAA GGAATAAAAA   
  
015401 "CGATTATTTG TATATTTATG TTCCCTAAGT AGA"TTACCTT GAGCCATGTA TACATTGCCT TGGTCTAAGC   
       GCTAATAAAC ATATAAATA"C AAGGGATTCA TCTAATGGAA CTCGGTACAT ATGTAACGGA ACCAGATTCG "  
  
015471 TAAAAAAAAG ACTATTTAAA AAACTAATCA A"TGATGACCC TCCATTGATT CGCCTATATA AGCTGCAGCT "  
       "ATTTTTTTTC TGATAAATT"T TTTGATTAGT T"ACTACTGGG AGGTAACTAA GCGGATATAT TCGACGTCGA "  
  
015541 "AAAGTTGCAA AAATAAGAGC TTGAATACCA C"TTGTAAATA ATCCAAGGAA CATAACAGGT ATAGGAACTA   
       "TTTCAACGTT TTTATTCTCG AACTTATGGT G"AACATTTAT TAGGTTCCTT GTATTGTCCA TATCCTTGAT   
  
015611 CTAAAGGTAC TAAAGAAACA AGAACAA"CAA CTACTAATTC ATCAGCTAAT ATATTTCCGA AAAGTCGAAA "  
       GATTTCCATG ATTTCTTTGT TCTTGTTGTT GATGATTAAG TAGTCGATTA TATAAAGGCT TTTCAGCTTT   
  
015681 "ACTAAGTGAT AGGGGTTTTG TGAAATC"TTC TAAGATGTTA ATGGGTAAAA GGATTGGAGT TGGTTGAATG   
       TGATTCACT"A TCCCCAAAAC ACTTTAGAAG ATTCTACAAT TACCCATTTT CCTAACCTCA ACCAACTTAC "  
  
015751 TATTTACCGA AATAACTTAA TCCTTTTTTG CTA"AGACCCG CATAAAAATA TGCTACTGAT GTGAGTAAAG "  
       "ATAAATGGC"T TTATTGAATT AGGAAAAAAC GATTCTGGGC GTATTTTTAT ACGATGACTA CACTCATTTC   
  
015821 "CTAAAGCAAC AGTAGTATTT ATATCATTTG TGG"GTGCGGC TAACTCTCCG TGAGGTAACT GTATGATTTT   
       GATTTCGTTG TCATCATAAA TATAGTAAAC ACCCACGCCG ATTGAGAGGC ACTCCATTGA CATACTAAAA   
  
015891 CCAAGGTAAA AGAGCCCCTG ACCAATTCGA AACA"AAAATA AATAGAAACA TAGTTCCAAT AAAGGGAACC "  
       GGTTCCATTT TCTCGGGGAC TGGTTAAGCT TTGTTTTTAT TTATCTTTGT ATCAAGGTTA TTTCCCTTGG   
  
015961 "CATGGACCAT ATTCTTCGCC AATTTGAGTT TTGC"TCACGT CTCGAATGAA TTCAAGGACA TATTCGAAGA   
       GTACCTGGTA TA"AGAAGCGG TTAAACTCAA AACGAGTGCA GAGCTTACTT AAGTTCCTGT ATAAGCTTCT "  
  
016031 AATTCTGACC GTCAGTAG"GA ATGGTTTGTG GATTACGAAC AGCTATAATG GCTGAACCTA ATAAGATAGC "  
       "TTAAGACTGG CA"GTCATCCT TACCAAACAC CTAATGCTTG TCGATATTAC CGACTTGGAT TATTCTATCG   
  
016101 "AATTACAACC CAAGAAGT"AA TAAGTACTTG GGCATGTACT TGGAAACCTC CTATTTGCCA ATACAAATGT   
       TTAATGTTGG GTTCTTCATT ATTCATGAAC CCGTACATGA ACCTTTGGAG GATAAAC"GGT TATGTTTACA "  
  
016171 TGGCCGACTT CCACACCAGA TATAT"CGTAT AACCCTTTTT GTGTGTTGAT AGAACATAAT AGAATATTCA "  
       "ACCGGCTGAA GGTGTGGTCT ATATAGCATA TTGGGAAAAA CACACAACTA TCTTGTA"TTA TCTTATAAGT   
  
016241 "TATTGCCCTC TGAAAGAAAT AGAAC"TGTAA AACAAATTAT TTTGATTCGA CTGTCTTTTT TTTTTTGACT   
       ATAACGGGAG ACTTTCTTTA TCTTGACATT TTGTTTAATA AAACTAAGCT GACAGAAAAA AAAAAACTGA   
  
016311 TGCCTTGAGT TGTCTATTTT T"GATACCAAT TTATTACATT AGAATATCCC TAGTTTTTTT TATCTCTTTT "  
       ACGGAACTCA ACAGATAAAA ACTATGGTTA AATAATGTAA TCTTATAGGG ATCAAAAAAA ATAGAGAAAA   
  
016381 "GTGCGATTCG GGAATAGTAA C"CAATTCAAT TCCCGAAATC TATGGAGTTC CCCCCAAAAT TGATTTATCT   
       CACGCTA"AGC CCTTATCATT GGTTAAGTTA AGGGCTTTAG ATACCTCAAG GGGGGTTTTA ACTAAATAGA "  
  
016451 TATTATTAAT CAAGAATT"TC GTATAGAGCT AGAACGACCC TCCGAAATAG CAAATACTAA TTTGTTAAGA "  
       "ATAATAA"TTA GTTCTTAAAG CATATCTCGA TCTTGCTGGG AGGCTTTATC GTTTATGATT AAACAATTCT   
  
016521 "ATTAATCGGA TTGAAGCT"AT AGCATCATCA TTCGCTGGAA TCGAAATATC GGCGAGATCG GGGTCACAAT   
       TAATTAGCCT A"ACTTCGATA TCGTAGTAGT AAGCGACCTT AGCTTTATAG CCGCTCTAGC CCCAGTGTTA "  
  
016591 TTGTATCAAT TAAACAAAT"A GTTGGAATTC CCAACGTGAT ACATTCTTGA AGAGCCCGAT ATTCTTCTTG "  
       "AACATAGTTA A"TTTGTTTAT CAACCTTAAG GGTTGCACTA TGTAAGAACT TCTCGGGCTA TAAGAAGAAC   
  
016661 "TTGATCAACG ATTATTACA"A TATCGGGTAA CCCCGTCATA TATTTAATCC CGCCCAGATA TGTTTGCAAG   
       AACTAGTTGC TAATAATGTT ATAGCCCATT GGGGCAGTAT ATAAATTAGG GCGGGTCTAT ACAAACGTTC   
  
016731 TGAGATAATT GTCTCTTG"AA CATAGCGGCA TCCCTTTTCG GAAGACGGTT GAGTCCCCCC GTCTTTTGTT "  
       ACTCTATTAA CAGAGA"ACTT GTATCGCCGT AGGGAAAAGC CTTCTGCCAA CTCAGGGGGG CAGAAAACAA "  
  
016801 "CCGTTCTCAA GTCCCTGA"AC TTATGAAGTC TCGTTTCTGT AGTAGACCAA TTTGTTAACA TCCCCCCGAG   
       "GGCAAGAGTT CAGGGA"CTTG AATACTTCAG AGCAAAGACA TCATCTGGTT AAACAATTGT AGGG"GGGCTC "  
  
016871 CCATTTTTTA TTAACATAA"T GACACCGAGT CCTTGTTGCA GCCCGGGCTA CTGAATCCGC TGCTTTATTT "  
       "GGTAAAAAAT AATTGTATTA CTGTGGCTCA GGAACAACGT CGGGCCCGAT GACTTAGGCG ACGA"AATAAA   
  
016941 "TTGGTACCAA CAATTAAGA"A TTGTTTGCCC CTAGTTGCTG CATCAAAAAC TAAATCACAA GCTTCTGATA   
       AACCATGGTT GTTAATTCTT AACAAACGGG GATCAACGAC GTAGTTTTTG ATTTAGTGTT CGAAGACTAT   
  
017011 AAAAACGAGC AGTTCTAGTA AGATTTATAA TA"TGAATACC TTTACGTTTT GCAGAGATAT AAGGTGCCAT "  
       TTTTTGCTCG TCAAGATCAT TCTAAATATT ATACTTATGG AAATGCAAAA CGTCTCTATA TTCCA"CGGTA "  
  
017081 "TCTAGGATTC CATTTCCTAG TACCATGACC AA"AATGAACT CCTGCTTCCA TCATCTCTTC CAAATGGATG   
       "AGATCCTAAG GTAAAGGATC ATGGTACTGG TTTTACTTGA GGACGAAGGT AGTAGAGAAG GTTTA"CCTAC   
  
017151 TTCCAATATC TTCTTGTCAT TTCTCCCCCC CACT"TCCTCG CTTTTTTTTC TTAAAAAAAG AGACGAGCTG "  
       AAGGTTATAG AAGAACAGTA AAGAGGGGGG GTGAAGGAGC GAAAAAAAAG AATTTTTTTC TCTGCTCGAC   
  
017221 "AAATAGAAAT AAATAATTGT TCCCATGGAA CCTT"ATCCTC TACCTCGACC GGGGATTGAC TGTTGATACA   
       TTTATCTTTA TTTATTAACA AGGGTACCTT GGAA"TAGGAG ATGGAGCTGG CCCCTAACTG ACAACTATGT "  
  
017291 CGATCCAAGC CATTAATTTT TTTTCTAT"TC GTTATTATCT TATTACCAAA TCAACGCGGC TAGTTAACAG "  
       "GCTAGGTTCG GTAATTAAAA AAAAGATAAG CAAT"AATAGA ATAATGGTTT AGTTGCGCCG ATCAATTGTC   
  
017361 "GATGACTCCA CTTTTATGAA TCATTAAA"TC CTAGAAATTC TTGTTCTGAT GTATCATGTA AATTCGTTGA   
       CTACTGAGGT GAAAATACTT AGTAATTTAG GATCTTTAAG AACAAGACTA CATAGTACAT TTAAGCAACT   
  
017431 AATGAAAAAA TCAAATAATT CT"TTGTGGTG GAACAAAATA TCTCTCATTT CCCCCTCGAA AATTTTTTTT "  
       TTACTTTTTT AGTTTATTAA GAAACACCAC CTTGTTTTAT AGAGAGTAAA GGGGGAGCTT TTAAAAAAAA   
  
017501 "GGGGGGGTTT CTAAAGGAAT AT"TGTTATGT TGCCTCGAAC GGTGCGCTAA TTCTCTGAAT CCCGTACCAA   
       CCCCCCCAAA GATTTCCTTA TAACAATACA ACGGAGCTTG CCACGCGATT AAGAGACTTA GGGCATGGTT   
  
017571 CGGGTATCAT GCCTCCTA"GA ACAACGTTCT CTTTCAAACC TCTCAACCAA TCGATACGAC CTCGGAGGGC "  
       GCCCATAGTA CGGAGGATCT TGTTGCAAGA GAAAGTTTGG AGAGTTGGTT AGCTATGCTG GAGCCTCCCG   
  
017641 "GGCTTTTGCT AAAACTCG"AG CAGTTTCTTG AAAACTTGCT TCCGATATGA AACTTTGAGT ATTTAGAGAT   
       CCGAAAACGA TTTTGAGCTC GTCAAAGAAC TTTTGAACGA AGGCTATACT TTGAAACTCA TAAATCTCTA   
  
017711 GCTTTCGTTA TTCCCAATAA TACGGCTC"GG TAACAGATCG CTTCTTCCAA AGCACGCCCT GTTCGTTCCG "  
       CGAAAGCAAT AAGGGTTATT ATGCCGAGCC ATTGTCTAGC GAAGAAGGTT TCGTGCGGGA CAAGCAAGGC   
  
017781 "CTCGTAACAA CCCAATGAGT TCTCCAGG"TG AAAAAACATT AGACATTCCA TCTTCTGAAA CCAACACTTT   
       GAGCATTGTT GGGTTACTCA AGAGGTCCAC TTTTTTGTAA TCTGTAAGGT AGAAGACTTT GGTTGTGAAA   
  
017851 TGATGTTATT TGACGTACAA TAATTTCGAT A"TGTCTATTA TGGATCTCCA CCCCCTGGGA TCGATAAACC "  
       ACTACAATAA ACTGCATGTT ATTAAAGCTA TACAGATAAT ACCTAGAGGT GGGGGACCCT AGCTATTTGG   
  
017921 "TTTTGGATCT TATTAACCAA AGAGATACGA C"TTTGCACTA TCGTTAGTTC AGCACCAATC AAAAATCCCC   
       AAAACCTAGA ATAATTGGTT TCTCTATGCT GAAACGTGAT AGCAATCAAG TCGTGGTTAG TTTTTAGGGG   
  
017991 AAGGAATTCC AAGAATT"CTT GTTATACGCT CGTTCCAACC CTCAATTCTC TTTTCTAGGC TCATCGATAT "  
       TTCCTTAAGG TTCTTAAGAA CAATATGCGA GCAAGGTTGG GAGTTAAGAG AAAAGATCCG AGTAGCTATA   
  
018061 "TGAATCAATC GAACGCA"CTT CTAATACTTG TTCGACTTTT GGAAGACCCT GCGTTATATC ACCAGATCGT   
       ACTTAGTTAG CTTGCGTGAA GATTATGAAC AAGCTGAAAA CCTTCTGGGA CGCAATATAG TGGTCTAGCA   
  
018131 GATTTTTCAT ATATAA"ATGT AACTAAAGTA TCTCCTTCGT AAAGGATTTC GCCATAATGG CGATGAACAG "  
       CTAAAAAGTA TATATTTACA TTGATTTCAT AGAGGAAGCA TTTCCTAAAG CGGTATTAC"C GCTACTTGTC "  
  
018201 "TTGCTCCTGA AGTGGC"CAAA TAAGGCTTAG CTGATCTTAT TACTAGAGAA TCAACTTGAA CAATTATAAT   
       "AACGAGGACT TCACCGGTTT ATTCCGAATC GACTAGAATA ATGATCTCTT AGTTGAACT"T GTTAATATTA   
  
018271 TTGACCCGAT TTTA"AGTGTG GTCTATTTTT GGCTATACAT ACATTTTCAA AAAAAAACTG TCCAAGACTA "  
       AACTGGGCTA AAATTCACAC CAGATAAAAA CCGATATGTA TGTAAAAGTT TTTTTTTGAC AGGTTCTGAT   
  
018341 "ATTCTTGTAG GTGT"TTCTTC ACAATAATTA TTGTGATAAT TATCATGCAG AAAATGCCAA TTCAAATTAA   
       TAAGAACATC CACAAAGAAG TGTTATTAAT AACACTATTA ATAGTACGTC TTTTACGGTT AAGTTTAATT   
  
018411 ATGTATTGAA AACGATGTTA ATGCAT"GGAT CGGAATTTAA AATCCCCCCG TTTTCATCCA TTAAATAATA "  
       TACATAACTT TTGCTACAAT TACGTACCTA GCCTTAAATT TTAGGGGGGC AAAAGTAGGT AATTTATTAT   
  
018481 "TTTAAGTACT TGAAAAATCT GTTTTA"AATT GTCAAGTTGC AAATATTTAG TTAGGGAGAT CGGATTATGA   
       AAATTCATGA ACTTTTTAGA CAAAATTTAA CAGTTCAACG TTTATAAATC AATCCCTCTA GCCTAATACT   
  
018551 GTTATTAAGT AATAAAATGA ATAAAAATTC ACA"ATTTGAG GGGCCGCCCC TAAAGGTCCT AACGAATTCC "  
       CAATAATTCA TTATTTTACT TATTTTTAAG TGTTAAACTC CCCGGCGGGG ATTTCCAGGA TTGCTTAAGG   
  
018621 "TAATTGAAAT TAGAGGATCC TTTTTACTTG ATT"CTTTTAT GACATTATAA TCTTTTACAT CGTTGAATAG   
       ATTAACTTTA ATCTCCTAGG AAAAATGAAC TAAGAAAATA CTGTAATATT AGAAAATGTA GCAACTTATC   
  
018691 ATCTATTTGC AAACAATTCG ATGCTGA"CAA AATTATCAAA GATTGGCGTT CTTTATTTCT ATTCAACAAC "  
       TAGATAAACG TTTGTTAAGC TACGACTGTT TTAATAGTTT CTAACCGCAA GAAATAAAGA TAAGTTGTTG   
  
018761 "GTACGAATAG TTCCTTGATT TTGACTC"GGA GATTGTGGAA CCCTTGTCTT GGAATAAATA GAAAAAAATG   
       CATGCTTATC AAGGAACTAA AACTGAGCCT CTAACACCTT GGGAACAGAA CCTTATTTAT CTTTTTTTAC   
  
018831 GATTGATATT GGTACGATCT GACTCATTAT TAAA"GATCAA TCCTGAACCT GACGGATCTT TCCTTTTTCT "  
       CTAACTATAA CCATGCTAGA CTGAGTAATA ATTTCTAGTT AGGACTTGGA CTGCCTAGAA AGGAAAAAGA   
  
018901 "GATATACGCA GTATCGGATT TCCCTAAGTC TATT"CGTAGG AAATTTCGAA CCAGACCATT TGTACTTACT   
       CTATATGCGT CATAGCCTAA AGGGATTCAG ATAAGCATCC TTTAAAGCTT GGTCTGGTAA ACATGAATGA   
  
018971 TCAACAAAGG AAGCGCGCGC GTCTTCGA"TA GAAGAGCTTT TTTTGTCTCG GTCCCAATTC AACAATAAAC "  
       AGTTGTTTC"C TTCGCGCGCG CAGAAGCTAT CTTCTCGAAA AAAACAGAGC CAGGGTTAAG TTGTTATTTG "  
  
019041 "AAGTCCGAAC TAATTGAATG CTTGTGCC"AG AAATTCCCCG AATTGGTTTG CCATTTCCAT AAAGTATATA   
       "TTCAGGCTT"G ATTAACTTAC GAACACGGTC TTTAAGGGGC TTAACCAAAC GGTAAAGGTA TTTCATATAT   
  
019111 ATTGGCAACT CTAAGCTCCA GATTA"TCCCT TTCTTGCAAT AGATCCTGTG GGAAAAGTTT GACTAAATTT "  
       TAACCGTTGA GATTCGAGGT CTAATAGGGA AAGAACGTTA TCTAGGACAC CCTTTTCAAA CTGATTTAAA   
  
019181 "ATACCGTCGG CTAGTTCATA TATGA"TTACA GGTCGAACAA AAACAAAATA CTTTTTCTTG GTAGGTGTGA   
       TATGGCAGCC GATCAAGTAT ATACTAATGT CCAGCTTGTT TTTGTTTTAT GAAAAAGAAC CATCCACACT   
  
019251 TCCACTGGAC ATAAATCCAA TTT"TTCGATT TTTTTGATTT CTTAGAATTT TTTTTTACTC TTTCTGGTGG "  
       AGGTGACCTG TATTTAGGTT AAAAAGCTAA AAAAACTAAA GAATCTTAAA AAAAAATGAG AAAGACCACC   
  
019321 "TATCAAGATC CCATTGTGTC GGG"ATATCTT ATCCATCTCT CCAGGAAAAT GTATATCTCC AGAAAATATT   
       ATAGTTCTAG GGTAACACAG CCCTATAGAA TAGGTAGAGA GGTCCTTTTA CATATAGAGG TCTTTTATAA   
  
019391 TTGAGTTCAA TCCTTTTTTT TTTTTTCT"CC ACTCGGACCA ATCCGCCTAC TCGGCTTCTT ATACTTAAAG "  
       AACTCAAGTT AGGAAAAAAA AAAAAAGAGG TGAGCCTGGT TAGGCGGATG AGCCGAAGAA TATGAATTTC   
  
019461 "TTAGTCGTGT AGCTACTCCA ATGATACT"AT TGTTCCGTAC CATTATGGAA GAAGATTCAG GTAAAATATG   
       AATCAGCACA TCGATGAGGT TACTATGATA ACAAGGCATG GTAATACCTT CTTCTAAGTC CATTTTATAC   
  
019531 CACTTCCTCG GGAATGAAAA "AAAATCGATC CACTTTCATT TGCATTTGGT ATTTTGGCTT AAAGTCTTTG "  
       GTGAAGGAGC CCTTACTTTT TTTTAGCTAG GTGAAAGTAA ACGTAAACCA TAAAACCGAA TTTCAGAAAC   
  
019601 "ACTCCTCGAT ACTCAATCAA" ATCCTCTTTT TTGAGGATTG AATGCACCCC TATAGTCCCA TATTTAGTAA   
       TGAGGAGCTA TGAGTTAGTT TAGGAGAAAA AACTCCTAAC TTACGTGGGG ATATCAGGGT ATAAATCATT   
  
019671 TTCCTGAACT ATTTC"TTCTG TATTGAGGAT CATCGAAAAA AGCAAGAATA CTATTTCTAC GAAAAATACC "  
       AAGGACTTGA TAAAGAAGAC ATAACTCCTA GTAGCTTTTT TCGTTCTTAT GATAAAGATG CTTTTTATGG   
  
019741 "ATTTATGGGT ATTTC"AATCG AAATACCGGA AGGGGGTCGT AGTTCTTTTT CTCGTTCTTG AAGTGATTCA   
       TAAATACCCA TAAAGTTAGC TTTATGGCCT TCCCCCAGCA TCAAGAAAAA GAGCAAGAAC TTCACTAAGT   
  
019811 AATTGAATGA TGAATCCATT TCT"TCGCCTC TTTGCCAATA AATCAGAATT ACCATGGAAA ATAGCAGGAT "  
       TTAACTTACT ACTTAGGTAA AGAAGCGGAG AAACGGTTAT TTAGTCTTAA TGGTACCTTT TATCGTCCTA   
  
019881 "ATGTGAGATT AGATTGACCC GTA"CATAGGA TTGGATTAAG TTCTGAATAA TCAGGAATTC CGTTTTCGTT   
       TACACTCTAA TCTAACTGGG CATGTATCCT AACCTAATTC AAGACTTATT AGTCCTTAAG GCAAAAGCAA   
  
019951 TTTACCAGAG AGAT"CCGGAC CATAAAATTT TTGTCCCACT TTATCATTAT TTACTGAAAG GCTAGAAATA "  
       AAATGGTCTC TCTAGGCCTG GTATTTTAAA AACAGGGTGA AATAGTAATA AATGACTTTC CGATCTTTAT   
  
020021 "TATCTTCTTT CGAC"ACAAAG AGAATGAACG TTAATTTGAT CTTGATCCTT GTAGAGTGAA AAAGGGACTA   
       ATAGAAGAAA GCTGTGTTTC TCTTACTTGC AATTAAACTA GAACTAGGAA CATCTCACTT TTTCCCTGAT   
  
020091 GACTGCATCC GCACGAACCT CCTGATAATA T"CCATAAATG ACTTGTTTTT GGTAAGAGAT GGACATTACT "  
       CTGACGTAGG CGTGCTTGGA GGACTATTAT AGGTATTTAC TGAACAAAAA CCATTCT"CTA CCTGTAATGA "  
  
020161 "ATATGTAAAT TCGGGTGCAT GGTACACGTC G"GTACTCCAA TGCATTTCTC CCTCTGAGTC AGAATAAATA   
       "TATACATTTA AGCCCACGTA CCATGTGCAG CCATGAGGTT ACGTAAAGAG GGAGACT"CAG TCTTATTTAT   
  
020231 TGTTTTCGAA CTCTCTCTTT TAAATTAAGA GT"GTATGTTC CCGCGCGAAT CTCAGCAATC ACTTGTTCTG "  
       ACAAAAGCTT GAGAGAGAAA ATTTAATTCT CACATACAAG GGCGCGCTTA GAGTCGTTAG TGAACAAGAC   
  
020301 "ATTCTACATA TTGATCGTTT TGAACTAAAA GT"AAACTTTT TGGTGGAATA GTGACGTTAT GTATAATATC   
       TAAGATGTAT AACTAGCAAA ACTTGATTTT CATTTGAAAA ACCACCTTAT CACTGCAATA CATATTATAG   
  
020371 CTCACTCTCA ATAGTTACAT ACAA"GTCTAT ATAACATAAA AAAGCAGGAT GCCCATGGCG TGTACGTGTG "  
       GAGTGAGAGT TATCAATGTA TGTTCAGATA TATTGTATTT TTTCGTCCTA CGGGTACCGC ACATGCACAC   
  
020441 "GGATGAACCA AATCCTCATT GAAT"TTTATT TTGCCATTAG AAGGAGCTCG TATATGTTCT GCAGTCCCCC   
       CCTACTTGGT TTAGGAGTAA CTTAAAATAA AACGGTAATC TTCCTCGAGC ATATACAAGA CGTCAGGGGG   
  
020511 CTGTGAATAC TCCGCCGGTA TGA"AAAGTTC TTAATGTTAG TTGAGTACCC GGTTCTCCAA TAGATTGACC "  
       GACACTTATG AGGCGGCCAT ACTTTTCAAG AATTACAATC AACTCATGGG CCAAGAGGTT ATCTAACTGG   
  
020581 "CGCAATAATA CCTACGGCTT CTC"CCAATTC GACCAGGTCC CCGTGAGTAG GACTTCGTCC ATAACATAAT   
       GCGTTATTAT GGATGCCGAA GAGGGTTAAG CTGGTCCAGG GGCACTCATC CTGAAGCAGG TATTGTATTA   
  
020651 CGGCAGATCC AAGATGTACT "CCTACAAGTA AAGGGAGTCC GAATAGATAT TGGTTGTGTT TGAAAGGTTA "  
       GCCGTCTAGG TTCTACATGA GGATGTTCAT TTCCCTCAGG CTTATCTATA ACCAACACAA ACTTTCCAAT   
  
020721 "TGAATCGATT AATAAGTCCA" ATACCAATAT CTTGATTTCG AACGCCAATG CATCGCGGGC CCATATATAT   
       ACTTAGCTAA TTATTCAGGT TATGGTTATA GAACTAAAGC TTGCGGTTAC GTAGCGCCCG GGTATATATA   
  
020791 ATCGTCCGCT AATACACGAC CAATTAAT"GT TTGGATAAAA ATTCTTTCCG GAATCATCCT ATTTCCAGGA "  
       TAGCAGGCGA TTATGTGCTG GTTAATTACA AACCTATTTT TAAGAAAGGC CTTAGTAGGA TAAAGGTCCT   
  
020861 "CTCACTAAAA TCCCTCGGAT GGTTCCAC"AA TCGGTTCTAC GTACAACAAT GTGTTGAACT ACTTCAACAA   
       GAGTGATTTT AGGGAGCCTA CCAAGGTGTT AGCCAAGATG CATGTTGTTA CACAACTTGA TGAAGTTGTT   
  
020931 GTCTACGTGT AAGA"TATCCA GCATCCGATG TTCGTACAGC AGTATCCACA ACTCCTTTCC GCGCTCCGTA "  
       CAGATGCACA TTCTATAGGT CGTAGGCTAC AAGCATGTCG TCATAGGTGT TGAGGAAAGG CGCGAGGCAT   
  
021001 "GCAAGAAATT ATAT"ATTCCG TTAAGGACAG CCCCTCACGT AAATTGCTTT GAATGGGTAA ATCAATCATT   
       CGTTCTTTAA TATATAAGGC AATTCCTGTC GGGGAGTGCA TTTAACGAAA CTTACCCATT TAGTTAGTAA   
  
021071 TGTCCTTGTG GATCTGACAT TAAT"CCTCTC ATACCTACTA ATTGGTGTAC TTGAGATGCA TTTCCTCGAG "  
       ACAGGAACAC CTAGACTGTA ATTAGGAGAG TATGGATGAT TAACCACATG AACTCTACGT AAAGGAGCTC   
  
021141 "CTCCCGAAAA AGACATTATA TGGA"CTGGAT TAAAAGGGTC AGTCATCCTA AAATTAGGAT TCATTTCTTG   
       GAGGGCTTTT TCTGTAATAT ACCTGACCTA ATTTTCCCAG TCAGTAGGAT TTTAATCCTA AGTAAAGAAC   
  
021211 TCGCAAATAT TCACTT"GTAG CATACCATAT CTCAATGGAC TGGCGTAATT TTTCTACTGC GTGTACGTTT "  
       AGCGTTTATA AGTGAACATC GTATGGTATA GAGTTACCTG ACCGCATTAA AAAGATGACG CACATGCAAA   
  
021281 "CCATAATGAT GGTGTT"TTTC CAAAATCAAA CTTTGTTGTT CAGCATCTTG GACTAGCCAT CCCTTAGAAG   
       GGTATTACTA CCACAAAAAG GTTTTAGTTT GAAACAACAA GTCGTAGAAC CTGATCGGTA GGGAATCTTC   
  
021351 GGATTGTTAA AAGATCATCA ATT"CCTAATG AAATGGATGT AGCAGTGGCC TGCTGGAACC CCAGAGTCTT "  
       CCTAACAATT TTCTAGTAGT TAAGGATTAC TTTACCTACA TCGTCAC"CGG ACGACCTTGG GGTCTCAGAA "  
  
021421 "TAGTTGATCC AGGATGTGTG ATG"TATATGC CATTCCGAAG TGATCTATTA ATCTGCTAAT AAGTCGTTTA   
       "ATCAACTAGG TCCTACACAC TACATATACG GTAAGGCTTC ACTAGAT"AAT TAGACGATTA TTCAGCAAAT   
  
021491 ATGGCAGTTC CATCTATCAC T"TTATTGTGA AAGACCAGAT CGGCCCGTTC CGCCATAAGT ACCTCCATAT "  
       TACCGTCAAG GTAGATAGTG AAATAACACT TTCTGGTCTA GCCGGGCAAG GCGGTATTCA TGGAGGTATA   
  
021561 "TACGCTGAGT GGGGTTCGAC A"ATAGGTTTG AGTTAATGAT TAGAAAACTT CCTTTTCTCG ATCTTGATTC   
       ATGCGACTCA CCCCAAGCTG TTATCCAAAC TCAATTACTA ATCTTTTGAA GGAAAAGAGC TAGAACTAAG   
  
021631 GCGTAGAAAT TCAGG"ACCTA TGGTCCTAGT TGAACCGGAG AGAACAAAAT TCATACCGGT GTCATATAAT "  
       CGCATCTTTA AGTCCTGG"AT ACCAGGATCA ACTTGGCCTC TCTTGTTTTA AGTATGGCCA CAGTATATTA "  
  
021701 "TACTTAGCTG AGTGA"GATAC CAGAGGATTA GGTACCATAT GAGCAGGCCC GGCAAAATCC TTGTATAGCC   
       "ATGAATCGAC TCACTCTA"TG GTCTCCTAAT CCATGGTATA CTCGTCCGGG CCGTTTTAGG AACATATCGG   
  
021771 TCTTCGATTT CTCGATAAA"G AGAAATATGA CCAACAGTGG TTCGAATGTA TATACAAAGA ATTTCTTTTT "  
       AGAAGCTAAA GAGCTATTTC TCTTTATACT GGTTGTCACC AAGCTTACAT ATATGTTTCT TAAAGAAAAA   
  
021841 "TTATACTTTT GACTATTAG"A TAGTACCCAT AAATCTCATG ATGGGTACCC AAAGATTCAT AGTGAACTTC   
       AATATGAAAA CTGATAATCT ATCATGGGTA TTTAGAGTAC TACCCATGGG TTTCTAAGTA TCACTTGAAG   
  
021911 GATAGGGGCT TCTCTTGAAG CAATAAC"GCG TTGATCTAGT CGCCATCGGA GCCACAAAGG ACTATCTAAA "  
       CTATCCCCGA AGAGAACTTC GTTATTGCGC AACTAGATCA GCG"GTAGCCT CGGTGTTTCC TGATAGATTT "  
  
021981 "TTGATCCTTT TCTGGCGATA AGCTCCA"ATT GCATCATAGG AATTACAAAA AAAGGGTTCT TTCGTATATT   
       "AACTAGGAAA AGACCGCTAT TCGAGGTTAA CGTAGTATCC TTA"ATGTTTT TTTCCCAAGA AAGCATATAA   
  
022051 TATAGTTATT ATTGTAAATT TTTTCATTTT "TATGATTTTT GCGATTAGAT GGATTATACC GATTTGCACA "  
       ATATCAATAA TAACATTTAA AAAAGTAAAA ATACTAAAAA CGCTAATCTA CCTAATATGG CTAAACGTGT   
  
022121 "AATACCTCGG CGATTCCCGC TCGTTAATAC" ATAGAGTCCA ATAAGCATAT CTTGAGTTGG TACAGAAATG   
       TTATGGAGCC GCTAAGGGCG AGCAATTATG TATCTCAGGT TATTCGTATA GAACTCAACC ATGTCTTTAC   
  
022191 GGATCCCCAA TAGCTGAAGA CAGGAG"ATTC ATATGAGAAA ACATAAGTAA ACGCGCCTCT GCTTGAGCCT "  
       CCTAGGGGTT ATCGACTTCT GTCCTCTAAG TATACTCTTT TGTATTCATT TGCGCGGAGA CGAACTCGGA   
  
022261 "CCCAAGATAA AGGTACATGA ACAGCC"ATTT GATCCCCATC AAAGTCTGCA TTGAATCCCT TACAAACTAA   
       GGGTTCTATT TCCATGTACT TGTCGGTAAA CTAGGGGTAG TTTCAGACGT AACTTAGGGA ATGTTTGATT   
  
022331 TGGATGTAAA CAAATAGCAC GT"CCTTCCAC TAAAATAGGT TGGAAGGCCT GGATGCCTAA TCTATGCAGA "  
       ACCTACATTT GTTTATCGTG CAGGAAGGTG ATTTTATCCA ACCTT"CCGGA CCTACGGATT AGATACGTCT "  
  
022401 "GTAGGCGCTC TATTTAGCAA TA"CGGGATGC CCCTGCATAA CTTCCTGAAG TATTTCCCAT ACAATGGGTT   
       "CATCCGCGAG ATAAATCGTT ATGCCCTACG GGGACGTATT GAAGG"ACTTC ATAAAGGGTA TGTTACCCAA   
  
022471 CTTTTTCCCG AATTTTACTC TTAGCAAC"TC CTATGTTCGA AGCAAAATGT TGTCGAATTA GACCACGAAT "  
       GAAAAAGGGC TTAAAATGAG AATCGTTGAG GATACAAGCT TCGTTTTACA ACAGCTTAAT CTGGTGCTTA   
  
022541 "TAAAAATGTC TGGAAAAGCT CTATTGCG"AT TTCGCGAGGC AATCCACATC GATGTAATGA AAGTGAGGGC   
       ATTTTTACAG ACCTTTTCGA GATAACGCTA AAGCGCTCCG TTAGGTGTAG CTACATTACT TTCACTCCCG   
  
022611 CCTACAACAA TCACA"GAACG TCCTGAATAA TCGACCCGTT TGCCAAGCAG AGTCTCCCGA AATCTTCCCT "  
       GGATGTTGTT AGTGTCTTGC AGGACTTATT AGCTGGGCAA ACGGTTCGTC TCAGAGGGCT TTAGAAGGGA   
  
022681 "CTTTGCCTTC AATTA"CATCA GAAAACGACT TGTAAACCTT ATTATGGCCA TCCCTCATTG GTTGTCCGCG   
       GAAACGGAAG TTAATGTAGT CTTTTGCTGA ACATT"TGGAA TAATACCGGT AGGGAGTAAC CAACAGGCGC "  
  
022751 GATTCCATTA TCAAGAAGTG TATCCA"CGGC TTCTTGTACC AATTTCTCCT GACACATTAC TAATTCTCCT "  
       "CTAAGGTAAT AGTTCTTCAC ATAGGTGCCG AAGAA"CATGG TTAAAGAGGA CTGTGTAATG ATTAAGAGGA   
  
022821 "GGCGTAGATC TACTTGTTGT TAATAG"ATCA ATAAGAGTAT TGTTCCGATA TATAACTCTT CTATAGAGTT   
       CCGCATCTAG ATGAACAACA ATTATCTAGT TATTCTCATA ACAAGGCTAT ATATTGAGAA GATATCTCAA   
  
022891 CATTAATATC CGAGCT"CATT AGTTTACCCC CATCTATCTG AATGATCGGT CTCAGCTCGG GAGGAAGAAC "  
       GTAATTATAG GCTCGAGTAA TCAAATGGGG GTAGATAGAC TTACTAGCCA GAGTCGAGCC CTCCTTCTTG   
  
022961 "AGGTAATAGA CATAAA"ACCA TCCATTCGGG TTCTATATTT GTTCGAATAA AATGCTTAGC TAATTCCATA   
       TCCATTATCT GTATTTTGGT AGGTAAGCCC AAGATATAAA CAAGCTTATT TTACGAATCG ATTAAGGTAT   
  
023031 CGTCTAACCA AAAAATCCTT TCTTCTTC"CA ACTTTGCGAT CTTCCCATTC ATTACCTGCG GGTCCTTCTT "  
       GCAGATTGGT TTTTTAGGAA AGAAGAAGGT TGAAACGCTA GAAGGGTAAG TAATGGACGC CCAGGAAGAA   
  
023101 "CGCCTAATTC TTTCCATTCT ACCAACGA"AT AATCTATAAT AAGTCGCAAA TCCAGATCGG CTAATTGTTC   
       GCGGATTAAG AAAGGTAAGA TGGTTGCTTA TTAGATATTA TTCAGCGTTT AGGTCTAGCC GATTAACAAG   
  
023171 TCGTATAGCA CCTGCTCCGG TAGAAAT"TTC TCGATTTCGA AATGTATCGA AGCCTTGGGT AGTAAAAAAA "  
       AGCATAT"CGT GGACGAGGCC ATCTTTAAAG AGCTAAAGCT TTACATAGCT TCGGAACCCA TCATTTTTTT "  
  
023241 "AGTGGGATGC TGTATTTCCA GGATTGG"ATT TCATATTCGA ATGAACCCCG TAATCGTAAG AAAGTAGGTT   
       "TCACCCT"ACG ACATAAAGGT CCTAACCTAA AGTATAAGCT TACTTGGGGC ATTAGCATTC TTTCATCCAA   
  
023311 TTTTAGCTAT GGGCCT"AGCA AAAGAAAAAT TTGGATAGGA TACTATAGGA TCTCCCCCTT TCAAAATCGG "  
       AAAATCGATA CCCGGATCGT TTTCTTTTTA AACCTATCCT ATGATATCCT AGAGGGGGAA AGTTTTAGCC   
  
023381 "ACGTGAAAGT TTCTTT"TCAT CCGGCTCAAG TAGTTACATC AAATAAAGGG ATTCCCGCTT TCAAATTCTA   
       TGCACTTTCA AAGAAAAGTA GGCCGAGTTC ATCAATGTAG TTTATTTCCC TAAGGGCGAA AGTTTAAGAT   
  
023451 TAACCCCCCC CAACTACTCT TTACTCAA"GT TCTCAATTAA GACCAAAGAA CATTACATTG ATTCTTTTGC "  
       A"TTGGGGGGG GTTGATGAGA AATGAGTTCA AGAGTTAATT CTGGTTTCTT GTAATGTAAC TAAGAAAACG "  
  
023521 "TTTTTTATTT CAATTTTCTG AGTTCTTT"AT TCAATATATC AATATTCAAT ATATCAATTA CAACATAAAA   
       "A"AAAAATAAA GTTAAAAGAC TCAAGAAATA AGTTATATAG TTATAAGTTA TATAGTTAAT GTTGTATTTT   
  
023591 AAAAAAAAAA AAGAAATGTG AAATTTTT"GA GTAGTCTATT TCCCTTGGAA TGACGAATCC CCTTAATGAA "  
       TTTTTTTTTT T"TCTTTACAC TTTAAAAACT CATCAGATAA AGGGAACCTT ACTGCTTAGG GGAATTACTT "  
  
023661 "AATTAATAAG GAGTGTATCT TGGAATTC"AT AAGGGATTTA TTTGTTTGTC TTTGTATCGT TCCATTCGAT   
       "TTAATTATTC C"TCACATAGA ACCTTAAGTA TTCCCTAAAT AAACAAACAG AAACATAGCA AGGTAAGCTA   
  
023731 CTTTTAGGTC CCTACTTTAC CTCGA"CGGTT ATGCCACGAT GTCCTTAAAG CCTATATGCG ATGGATAGAC "  
       GAAAATCCA"G GGATGAAATG GAGCTGCCAA TACGGTGCTA CAGGAATTTC GGATATACGC TACCTATCTG "  
  
023801 "TCCTTTAACC ATGACATATT TGCTT"ATTTG AACATAATTT CATTTCTTTC TGAAAGAAAA AGGAATGGTT   
       "AGGAAATTG"G TACTGTATAA ACGAATAAAC TTGTATTAAA GTAAAGAAAG ACTTTCTTTT TCCTTACCAA   
  
023871 AATTACACAA AAGAAATAAG TTTTTT"CTTC ACGAGGTAGA ACTAAAAATT CCTATTTATT ATTTATTACT "  
       TTAATGTGTT TTCTTT"ATTC AAAAAAGAAG TGCTCCATCT TGATTTTTAA GGATAAATAA TAAATAATGA "  
  
023941 "GAATCGACCA TAGACCAATT CCCCTT"TTAT TTGGGATGGG AGTATTGACT ACACCCAAAA TTCTGAGCTT   
       "CTTAGCTGGT ATCTGG"TTAA GGGGAAAATA AACCCTACCC TCATAACTGA TGTGGGTTTT AAGACTCGAA   
  
024011 CATGTTACTC CTACCAAGCA A"GACACATGT CAGATCCGGG GCATCCCAAT TCGATTGAAT GGGATGACAG "  
       GTACAAT"GAG GATGGTTCGT TCTGTGTACA GTCTAGGCCC CGTAGGGTTA AGCTAACTTA CCCTACTGTC "  
  
024081 "TTTCTCATTC TGAATCTTAA A"AATAAAAAT TTTGATCAAA TCACACATCG CAATATACTA AGCCTTCTAA   
       "AAAGAGT"AAG ACTTAGAATT TTTATTTTTA AAACTAGTTT AGTGTGTAGC GTTATATGAT TCGGAAGATT   
  
024151 TTCTTTAAGA GGTTT"ATCTA AAAGATTCGC GATATAACTA GGAAGACGTT TTAAATACCA CACATGGGTT "  
       AAGAAATTCT CCA"AATAGAT TTTCTAAGCG CTATATTGAT CCTTCTGCAA AATTTATGGT GTGTACCCAA "  
  
024221 "ACTGGGCATG CCAGT"TTAAT ATAGCCCATT TGATATCTTC GTATCCGAGA ATCAACAAAT TCGACTCCGC   
       "TGACCCGTAC GGT"CAAATTA TATCGG"GTAA ACTATAGAAG CATAGGCTCT TAGTTGTTTA AGCTGAGGCG "  
  
024291 ATTGTTCACA AAATTTCGAA TCT"TCTTTTT TATCTCCGAT TACTCGATAA TTTCCACAAG CACAAATCCC "  
       "TAACAAGTGT TTTAAAGCTT AGAAGA"AAAA ATAGAGGCTA ATGAGCTATT AAAGGTGTTC GTGTTTAGGG   
  
024361 "GCTTTTTATA GGCCCAAAAA TTC"TTTCACA AAATAATCCA TCTTTTTCAG GTTTATTGGT TTTGTAATGA   
       CGAAAA"ATAT CCGGGTTTTT AAGAAAGTGT TTTATTAGGT AGAAAAAGTC CAAATAACCA AAACATTACT "  
  
024431 AAAGTATAGG GTTTTGTT"AC CTCTCCAACT ATCTCGCCAT TAGGTAGAGT TTTATTGGCC CAAGCACTTA "  
       "TTTCAT"ATCC CAAAACAATG GAGAGGTTGA TAGAGCGGTA AT"CCATCTCA AAATAACCGG GTTCGTGAAT "  
  
024501 "TTTGTTGAGG AGAAACTA"AT CCAATTCGGA GTTGTTGATG TTTATACCGA TCGATCATAG AAAAAATTTT   
       "AAACAACTCC TCTTTGATTA GGTTAAGCCT CAACAACTAC AA"ATATGGCT AGCTAGTATC TTTTTTAAAA   
  
024571 CTGATTCATT TTGATTAAGC TTCCTTCCTA TTA"ATCTGGA AGTTCTTCTC AGATACAAGG AAATGATTCA "  
       GACTAAGTAA AACTAATTCG AAGGAAGGAT AATTAGACCT TCAAGAAGAG TCTATGTTCC TTTACTAAGT   
  
024641 "GTTCCAAAGC CAAAGATCGT AATTCTCGAA CGA"GCAATCG AAAAGATTCT GGAGTATCCT CGGGTTTAGG   
       CAAGGTTTCG GTTTCTAGCA TTAAGAGCTT GCTCGTTAGC TTTTCTAAGA CCTCATAGGA GCCCAAATCC   
  
024711 TATTGTTCCT CCAATGATCG TAGTACCA"AG TACTTCTTGC CGAGCTCTAA TATGATCAGA TTTATAAGTA "  
       ATAACAAGGA GGTTACTAGC ATCATGGTTC ATGAAGAACG GCTCGAGATT ATACTAGTCT AAATATTCAT   
  
024781 "AGCATCTCTT GTAAAATATG AGCAACCC"CA AATCCTTCTA GAGCCCAAAC CTCCATTTCT CCCACCCGTT   
       TCGTAGAGAA CATTTTATAC TCGTTGGGGT TTAGGAAGAT CTCGGGTTTG GAGGTAAAGA GGGTGGGCAA   
  
024851 GTCCACCTTG CTTGG"CCCTT CCTCTAAGGG GTTGTTGTGT AACAAGTGCA TAATGTCCAC TGGAACGTCC "  
       CAGGTGGAAC GAACCGGGAA GGAGATTCCC CAACAACACA TTGTTCACGT ATTACAGGTG ACCTTGCAGG   
  
024921 "GTGTATTTTA TCATC"AACTT GATGAATTAA TTTCAAGATA TAAGGCTTTC CTATTATAAC AGGTTGTTCA   
       CACATAAAAT AGTAGTTGAA CTACTTAATT AAAGTTCTAT ATTCCGAAAG GATAATATTG TCCAACAAGT   
  
024991 AAAGGATTTC CCGTTCTTCC ATCA"AATATT CTGCTTTTTC CCGGATACTC GGGTTCAAAT ACCCATGGGG "  
       TTTCCTAAAG GGCAAGAAGG TAGTTTATAA GACGAAAAAG GGCCTATGAG CCCAAGTTTA TGGGTACCCC   
  
025061 "ACTTCGCTGT TTGCTTACTG GCTT"CATATA ATTCAGAAAA GACTAGTTTT CTAGAAGCCT CTTGTTCATA   
       TGAAGCGACA AACGAATGAC CGAAGTATAT TAAGTCTTTT CTGATCAAAA GATCTTCGGA GAACAAGTAT   
  
025131 TCTCTCATCA AAAGGTACTA T"TCGATAATG TCTATCTAGC AAACTCCCCG CTAACCCGAG TGAGCACTCA "  
       AGAGAGTAGT TTTCCATGAT AAGCTATTAC AGATAGATCG TTTGAGGG"GC GATTGGGCTC ACTCGTGAGT "  
  
025201 "AATATCTGTC CTACATTCAT T"CGTGAGGGG ACTCCTAATG GATTGAAAAC CATATCAACA GGTCTTCCAT   
       "TTATAGACAG GATGTAAGTA AGCACTCCCC TGAGGATTAC CTAACTTT"TG GTATAGTTGT CCAGAAGGTA   
  
025271 TTTGCAAATA AGGCATATCT TCTCT"GGGCA AAATTTTTGA AACAATACCT TTATTTCCAT GTCTTCCCGC "  
       AAACGTTTAT TCCGTATAGA AGAGACCCGT TTTAAAAACT TTGTTATGGA AATAAAGGTA CAGAAGGGCG   
  
025341 "TACCTTATCG CCTACTTTGA TTTCA"CGTTT CTGTGAAATA TATACACGAA TAATTTCTGG ATTATAACTC   
       ATGGAATAGC GGATGAAACT AAAGTGCAAA GACACTTTAT ATATGTGCTT ATTAAAGACC TAATATTGAG   
  
025411 GAACCCCCTT TTTTCTGGAT CCAT"CTCACA TCAATAACTC GACCCCTACC GCCGATAGGT AGTTTTAGAC "  
       CTTGGGGGAA AAAAGACCTA GGTAGAGTGT AGTTATTGAG CTGGGGATGG CGGCTATCCA TCAAAATCTG   
  
025481 "AGGTTTCTTT TGAAGTGGAT ACCT"GAATGC CAAGTATGGC TCGTAATAAT CTATCTTCCG GGGCATACGA   
       TCCAAAGAAA ACTTCACCTA TGGACTTACG GTTCATACCG AGCATTATTA GATAGAAGGC CCCGTATGCT   
  
025551 TGATTCTTTC GCCATCT"GAG GCGTTAATTT ACCTACTAAA ATATCGCCCG TCTCCACCCA CGATCCCAGC "  
       ACTAAGAAAG CGGTAGACTC CGCAATTAAA TGGATGATTT TATAGCGGGC AGAGGTGGGT GCTAGGGTCG   
  
025621 "ATAACAATTC CATTTTT"GTC TAAATTACAG AGTAAGCGGG CTTCTAGATG TGGTATTTCG TTAGTGATTC   
       TATTGTTAAG GTAAAAACAG ATTTAATGTC TCATTCGCCC GAAGATCTAC ACCATAAAGC AATCACTAAG   
  
025691 TTTCAGGTCC GTGGCTTGT"C ACATGAGTTT GAATTTCATA TTTCCGTATG TGGAAAGAAG TATAAATATC "  
       AAAGTCCAGG CACCGAACAG TGTACTCAAA CTTAAAGTAT AAAGGCATAC ACCTTTCTTC ATATTTATAG   
  
025761 "TTCATATATC AAACGCTCG"C TAATTAGTAC CGCATCTTCA AAATTGTAAC CTTCCCATGG CATATAAGCT   
       AAGTATATAG TTTGCGAGCG ATTAATCATG GCGTAGAAGT TTTAACATTG GAAGGGTACC GTATATTCGA   
  
025831 ACTAATACGT TTTTTCCCAA AGCAAGTTCG C"CACCAACTG TAGCAGCACC GTCCGCTAAA ATTTGCCCTT "  
       TGATTATGCA AAAAAGGGTT TCGTTCAAGC GGTGGTTGAC ATCGTCGTGG CAGGCGATTT TAAACGGGAA   
  
025901 "TTTTTATGCA TTTACCCTGG TGAACCTGGG A"TTTTTGCTG CATACAAGTA TTTTTGTTAG AACGTTGATA   
       AAAAATACGT AAATGGGACC ACTTGGACCC TAAAAACGAC GTATGTTCAT AAAAACAATC TTGCAACTAT   
  
025971 CATAACTAAT GGAATTCTTC "GAGTATATCC ATTGCCCGAA AAAATGATCT TGTCAGTATC GGTATAAATG "  
       GTATTGATTA CCTTAAGAAG CTCATATAGG TAACGGGCTT TTTTACTAGA ACAGTCATAG CCATATTTAC   
  
026041 "ATCTTTCCCC CGTGTTCGGC" TATAGCGGCA ACCCCTGAAT CTCGAGCCAC TTGGCGTTCC AACCCAGTTC   
       TAGAAAGGGG GCACAAGCCG ATATCGCCGT TGGGGACTTA GAGCTCGGTG AACCGCAAGG TTGGGTCAAG   
  
026111 CAACAATGCA CTTTTCGGAC CGAGAAA"GCG GGACTGCTTG ACGTTGCATA TTAGAACTCA TTAAAGCACG "  
       GTTGTTACGT G"AAAAGCCTG GCTCTTTCGC CCTGACGAAC TGCAACGTAT AATCTTGAGT AATTTCGTGC "  
  
026181 "ATTCGCATCA TTATGTTCGA TAAAAGG"AAT GAGGGAAGCT CCAATAGAAA AATATTGGAA GGGAAAAATA   
       "TAAGCGTAGT A"ATACAAGCT ATTTTCCTTA CTCCCTTCGA GGTTATCTTT TTATAACCTT CCCTTTTTAT   
  
026251 CTTCGAAGAT GAACTTGTTC CCATTCAATA G"TCAAGAATT CTTGACGGTA TCGAGCTGGA ACAATCTGTT "  
       GAAGCTTCTA CTTGAACAAG GGTAAGTTAT CAGTTCTTAA GAACTGCCAT AGCTCGACCT TGTTAGACAA   
  
026321 "CTTCCTGAAT ACCTGGATTC AGTGCCAAAG A"ATTTCCTGT CGCTACCATA TAGTATTCAT CTCGACTTGG   
       GAAGGACTTA TGGACCTAAG TCACGGTTTC TTAAAGGACA GCGATGGTAT ATCATAAGTA GAGCTGAACC   
  
026391 TGATAAATAA AGCA"TCCGCA CTTTTTTTGA TCTCTCTGAA ATTTCATAAA ATGGAGTTTC TAGAGATCCC "  
       ACTATTTATT TCGTAGGCGT GAAAAAAACT AGAGAGACTT TAAAGTATTT TACCTCAAAG ATCTCTAGGG   
  
026461 "CAATGACCAA TCCT"TGCATG AATTGCTAAT GATCCAATAA GTCCAACATT AATTCCTTCA GACGTGTCAA   
       GTTACTGGTT AGGAACGTAC TTAACGATTA CTAGGTTATT CAGGTTGTAA TTAAGGAAGT CTGCACAGTT   
  
026531 TTGGGCAAAT GCGTCCATAA T"GACTAGGAT GGATATCTCG TATCCGAAAA CTAGCAGTTC GCCCTGTCAA "  
       AACCCGTTTA CGCAGGTATT ACTGATCCTA CCTATAGAGC ATAGGCTTTT GAT"CGTCAAG CGGGACAGTT "  
  
026601 "TCCTCCTGGG CCCAAATAAC T"CAATTTTCG CCCATGAACT ATTTGTGTCA ATGGATTAGT TCGATCTAAA   
       "AGGAGGACCC GGGTTTATTG AGTTAAAAGC GGGTACTTGA TAAACACAGT TAC"CTAATCA AGCTAGATTT   
  
026671 ACTTGAGATA ATGG"GTGTAA TCCGAAAAAA GATTCAAATG TGGTTGTTAA TGGAGTTGAA GTTACCAAAT "  
       TGAACTCTAT TACCCACATT AGGCTTTTTT CTAAGTTTAC ACCAACAATT ACCTCAACTT CAATGGTTTA   
  
026741 "TCTGAGGAGT CGGT"ATCAAT TTATGCCTAA TTGCTCCACA TATAGTTCCT CTAACCATAT TTTCTAAACG   
       AGACTCCTCA GCCATAGTTA AATACGGATT AACGAGGTGT ATATCAAGGA GATTGGTATA AAAGATTTGC   
  
026811 AACTAGAGCC AATCCGAATT G"ATCTTGTAA GAGATCCGCG ACAGAACGAA TCCGTTTATT TTTCAAATGA "  
       TTGATCTCGG TTAGGCTTAA CTAGAACATT CTCTAGGCGC TGTCTTGCTT AGGCAAATAA AAAGTTTACT   
  
026881 "TTCATATCGT CAAGTGTACC C"ATTCCAAAT TTAAGTCCAA TCAAATGATC CGCAGCTGCC AATATATCTC   
       AAGTATAGCA GTTCACATGG GTAAGGTTTA AATTCAGGTT AGTTTACTAG GCGTCGACGG TTATATAGAG   
  
026951 GCTGTAACAA AAATGTAT"TG TTCTCAGGTA TATCAAGATT AAGTCTCTGG TTCAGATTTC GTCGACCAAT "  
       CGACATTGTT TTTACATAAC AAGAGTCCAT ATAGTTCTAA TTCAGAGACC AAGTCTAAAG CAGCTGGTTA   
  
027021 "CCTTCCTAAT TCACATCT"TT GTTGAAAGAA TTTCTTTTGT AATTCCTTAC ATAAGGATTC AGAAAATACC   
       GGAAGGATTA AGTGTAGAAA CAACTTTCTT AAAGAAAACA TTAAGGAATG TATTCCTAAG TCTTTTATGG   
  
027091 GGATCTCCGC CTACACAAGA "AAATTGTTGA TAAAACTCCA AAATGGCATT TTCTTTTGAT CCAATTTTTT "  
       CCTAGAGGCG GATGTGTTCT TTTAACAACT ATTTTGAGGT TTTACCGTAA AAGAAAACTA GGTTAAAAAA   
  
027161 "TTTTCTCCTT ATCATTCAGG" AAAGACAAAA AAATTTCAGG GTAGCAAACA TTCTCGAGAA TTTCTCTTAG   
       AAAAGAGGAA TAGTAAGTCC TTTCTGTTTT TTTAAAGTCC CATCGTTTGT AAGAGCTCTT AAAGAGAATC   
  
027231 ATTCGAACCC ATAGC"TGATG ATAGAACTAG AATAGATATT TTCTGTTTCC TACTCACACG AGCCCATATC "  
       TAAGCTTGGG TATCGACTAC TATCTTGATC TTATCTATAA AAGACAAAGG ATGAGTGTGC TCGGGTATAG   
  
027301 "CTTGCTTTTC GATCA"ATCTC TAATTCTAAT CTTCCTCCCC AATCAGATAT TATGGTGCCG GTATAGACCG   
       GAACGAAAAG CTAGTTAGAG ATTAAGATTA GAAGGAGGGG TTAGTCTATA ATA"CCACGGC CATATCTGGC "  
  
027371 AAATTCCGTT ATGGTCCAAT "TCTGACCGGT AATAGATACC GGGGCTTTGC AATATTTGAC TGATCACAAT "  
       "TTTAAGGCAA TACCAGGTTA AGACTGGCCA TTATCTATGG CCCCGAAACG TTA"TAAACTG ACTAGTGTTA   
  
027441 "TCGGTATAGT CCATTTACTA" TAGAAGTTCC CAGGGAATTC ATTAGAGGAA TGTTTCCAAT AAAAATTGTT   
       AGCCATATCA GGTAAATGAT ATCTTCAAGG GTCCCTTAAG TAATCTCCTT ACAAAGGTTA TTTTTAACAA   
  
027511 TGTTCTTGCA TATCCCTA"CG GGTTTTCCAA ATTAATCCTG CGGATACGTA TAATTCAGAA GAATATGTGA "  
       ACAAGAACGT ATAGGGATGC CCAAAAGGTT TAATTAGGAC GCCTATGCAT ATTAAGTCTT CTTATACACT   
  
027581 "GTGATTCATA TACAGCAT"CT CGTTCTTTTA TCAATGGTTC TACCAATTTA TATGTTTCCA CAAATAATTG   
       CACTAAGTAT ATGTCGTAGA GCAAGAAAAT AGTTACCAAG ATGGTTAAAT ATACAAAGGT GTTTATTAAC   
  
027651 AAATTCAATT TCTTGATCTG TATCTTCAAT T"TTTGGAAAC TTAGAAAGTT CTTCTGTTAA GCCATGATCA "  
       TTTAAGTTAA AGAACTAGAC ATAGAAGTTA AAAACCTTTG AATCTTTCAA GAAGACAATT CGGTACTAGT   
  
027721 "ATGAACCTAC AAAACCCTTC AAATTGTATC T"GATTAAACG CAGGTATTGT AGACATTCTC TCATTTCCAC   
       TACTTGGATG TTTTGGGAAG TTTAACATAG ACTAATTTGC GTCCATAACA TCTGTAAGAG AGTAAAGGTG   
  
027791 CCCCAAGCAT TTTATTTATT TC"CCATTTAT AAAAAAAATC CCATTATTTG CTTATTCATC ATAAAATGGA "  
       GGGGTTCGTA AAATAAATAA AGGGTAAATA TTTTTTTTAG GGTAATAAAC GAATAAGTAG TATTTTACCT   
  
027861 "TCGATCTAGC AATGATGGAA TT"TATATTAT GTTTACTGAA TCACATGAAA TTTTACCTAA CTTCATAGGT   
       AGCTAGAT"CG TTACTACCTT AAATATAATA CAAATGACTT AGTGTACTTT AAAATGGATT GAAGTATCCA "  
  
027931 GTAATAGATG TAATGCATGA AAT"ACATATG AACGTAGGAA TAAAGAGACT TTGATACTCA AATGAAATTG "  
       "CATTATCT"AC ATTACGTACT TTATGTATAC TTGCATCCTT ATTTCTCTGA AACTATGAGT TTACTTTAAC   
  
028001 "GAATTTGCAA CAACTCCAAA TGA"ATTGGTA AATTCTATTT AGAATTCTGG AGTTTTGTAT AAAATATCTA   
       CTTAAACGTT GTTGAGGTTT ACTTAA"CCAT TTAAGATAAA TCTTAAGACC TCAAAACATA TTTTATAGAT "  
  
028071 TATCAAAACA AAAGTGAGTC AATTTCTACC ATTA"TTATGG TATTCCAATC CGATTGGGTA CTATAAATAG "  
       "ATAGTTTTGT TTTCACTCAG TTAAAG"ATGG TAATAATACC ATAAGGTTAG GCTAACCCAT GATATTTATC   
  
028141 "ATTCAGGATT TGATCTGCAG AAACAATATA GAAT"TTTTTT TTATTTAACA ACATGGAACT TTTTCGTAGA   
       TAAGTCCTAA ACTAGA"CGTC TTTGTTATAT CTTAAAAAAA AATAAATTGT TGTACCTTGA AAAAGCATCT "  
  
028211 TTTCACTGTT AAAAAAAATT C"GCATACAAG AGAGATATTT TACCTATACC TATATTTTTT TTTAGTAGGA "  
       "AAAGTGACAA TTTTTT"TTAA GCGTATGTTC TCTCTATAAA ATGGATATGG ATATAAAAAA AAATCATCCT   
  
028281 "TTCGTAGAAT CCGATTGAAG T"ATGTATAAG GACTTGTATT TATTAATAAA GATGTGCAGA TATATATAGT   
       AAGCATCT"TA GGCTAACTTC ATACATATTC CTGAACATAA ATAATTATTT CTACACGTCT ATATATATCA "  
  
028351 TAGTTATATA TATCGCCTCC TTTATT"ACAG TTCTATGGGG GACATCACAT GTCGTACTCT ATCAAAAATT "  
       "ATCAATAT"AT ATAGCGGAGG AAATAATGTC AAGATACCCC CTGTAGTGTA CAGCATGAGA TAGTTTTTAA   
  
028421 "TCTATTTTCT ATTTAATGAT AATGAA"AAAA AAAATTGTAA AAACGGAATT CCGAACATTT CCTATAAACA   
       AGATAAAAGA TAAATTACTA T"TACTTTTTT TTTTAACATT TTTGCCTTAA GGCTTGTAAA GGATATTTGT "  
  
028491 TCTTATATTA TATATATA"TA AGATAACCAA TTAGATTTAG ATAATAGTAA TCGTTTATGT TCTGGGGTTT "  
       "AGAATATAAT ATATATATAT T"CTATTGGTT AATCTAAATC TATTATCATT AGCAAATACA AGACCCCAAA   
  
028561 "ACATATCCTC ATAATCGC"TA TTAAAATTTT CATTAATAAG GATTTTTTTA TGGGTATGGA AACGAATCAA   
       TGTATAGG"AG TATTAGCGAT AATTTTAAAA GTAATTATTC CTAAAAAAAT ACCCATACCT TTGCTTAGTT "  
  
028631 TACGGATGGA TTAGTAATGA TTAGTTATCT ATCA"ATTTTT ATTTATTTAG GTAAGGTATC CATTTTGGGA "  
       "ATGCCTAC"CT AATCATTACT AATCAATAGA TAGTTAAAAA TAAATAAATC CATTCCATAG GTAAAACCCT   
  
028701 "TTGTTTTCTT ATATCATTAG GGAAACCGAA TTTG"CAATTT CAATCCAAGA ATCGTTTATG AATTCACAGT   
       AACAAAAGAA TATAGTAATC CCTTTG"GCTT AAACGTTAAA GTTAGGTTCT TAGCAAATAC TTAAGTGTCA "  
  
028771 CAATAGTTAA AGTTAAC"GGT TCCCATTTGT CCTGAATTTT TTCTTTTGTG AGTGAAAATC CACATTTTTT "  
       "GTTATCAATT TCAATTGCCA AGGGTA"AACA GGACTTAAAA AAGAAAACAC TCACTTTTAG GTGTAAAAAA   
  
028841 "TTTTCTTTGA ATAGAAA"AAG CAAAGTTTTG CGGTTTTTAG TTTTAGATAT TGTGTGTTGT AAGATACTAT   
       AAAAGAAACT TATCTTTTTC GTTTCAAA"AC GCCAAAAATC AAAATCTATA ACACACAACA TTCTATGATA "  
  
028911 CTATCTAAGA GATAGAGAAA ATCCAATATT TTGTATCGTT TTGGCG"ACAT GGCCGAGCGG TAAGGCGGGG "  
       "GATAGATTCT CTATCTCTTT TAGGTTAT"AA AACATAGCAA AACCGCTGTA CCGGCTCGCC ATTCCGCCCC   
  
028981 "GACTGCAAAT CCCTTTTTCC CCAGTTCAAA TCCGGGTGTC GCCTCA"TCAA CAAACAAAAG AATCAAAATA   
       CTGACGTTTA GGGAAAAA"GG GGTCAAGTTT AGGCCCACAG CGGAGTAGTT GTTTGTTTTC TTAGTTTTAT "  
  
029051 CCTTCTTCTG TTTTGCTGAT ATTGATATAA CTTTATCATT TTTT"TTTCCA GCAGAAGAAA AGCCTCTTTA "  
       "GGAAGAAGAC AAAACGAC"TA TAACTATATT GAAATAGTAA AAAAAAAGGT CGTCTTCTTT TCGGAGAAAT   
  
029121 "GATTTGCTTG ATTCTAAGCA TCGTTGGGGT TCTCTAAAAC GTTA"TAAATC CTTGCGTATA GGTTTCGTTT   
       CTAAACGAAC TAAGATTCGT A"GCAACCCCA AGAGATTTTG CAATATTTAG GAACGCATAT CCAAAGCAAA "  
  
029191 CCAGAATTTA GTAGAGTAAT GAAAAATAAT CGTTAAATCT TGA"GATGATA GCCCTTCGAC TACTACTACT "  
       "GGTCTTAAAT CATCTCATTA C"TTTTTATTA GCAATTTAGA ACTCTACTAT CGGGAAGCTG ATGATGATGA   
  
029261 "AATGTAGTGT CTACTCATTG GGTCTTGAAT TAGGGAATAG AAT"TTCATTT TTCGTTACGG AAAGGAGGAA   
       TTACATCACA GATGAGTAAC CCAGAACT"TA ATCCCTTATC TTAAAGTAAA AAGCAATGCC TTTCCTCCTT "  
  
029331 GATACTTTAT ATACGGACTC ATGAAA"GTAT CTGAGTACTC GAGCATTCAA TCAATATTAT ATTGAATGGA "  
       "CTATGAAATA TATGCCTGAG TACTTTCA"TA GACTCATGAG CTCGTAAGTT AGTTATAATA TAACTTACCT   
  
029401 "TAATTGTCTT TTTTTTTTAT TTTAAT"CTTA AAAAAAAGGA ACTTATTTCT TTTCGATAAG CTCTAACCAC   
       ATTAACAGAA AAAAAAAATA AAA"TTAGAAT TTTTTTTCCT TGAATAAAGA AAAGCTATTC GAGATTGGTG "  
  
029471 ACATGTAATA GGCCATCCCA TCTTCCGATT GTCTCTATGA AACAA"TCGGG GGACAGTAAA GATTAGATCA "  
       "TGTACATTAT CCGGTAGGGT AGA"AGGCTAA CAGAGATACT TTGTTAGCCC CCTGTCATTT CTAATCTAGT   
  
029541 "AATGAGAAAG GAATAATAGT AGGGTTATGT GATAGATACT ATCTT"GATTC TCTATTTTGA GACTTTTTAC   
       TTACTCTTTC CTTATTATCA TCCCAAT"ACA CTATCTATGA TAGAACTAAG AGATAAAACT CTGAAAAATG "  
  
029611 TTAATGTAAT GAAATGCAGG ACAACAAA"AG AAAGACTAGG TCTTATTATT CCTACATGTT ACTAACATTC "  
       "AATTACATTA CTTTACGTCC TGTTGTT"TTC TTTCTGATCC AGAATAATAA GGATGTACAA TGATTGTAAG   
  
029681 "CTTTGATACT TCCAAGAGTT TTTCTGCC"TC GTTTATTTAT TTGTACTGAA CTTTTTCGAT TATACTAGAA   
       GAAACTAT"GA AGGTTCTCAA AAAGACGGAG CAAATAAATA AACATGACTT GAAAAAGCTA ATATGATCTT "  
  
029751 ATCATATAAT AACTTGGGAT AATTCGATTT TTTGTATTGT T"TCATCAATG GTGTTGTGCC CGAATCTCTT "  
       "TAGTATAT"TA TTGAACCCTA TTAAGCTAAA AAACATAACA AAGTAGTTAC CACAACACGG GCTTAGAGAA   
  
029821 "TTTGACTATG CGCTAGTGAT TCCACTATTA TTAGTGAATA A"TAATTATTA GTGAGCAATA ATGGAATAAT   
       AAACTGATAC GCGATCACTA AGGTGAT"AAT AATCACTTAT TATTAATAAT CACTCGTTAT TACCTTATTA "  
  
029891 TCTTTTATAT TCATAGAGAT AGGGGACATA ATTCACATGG ATAT"GGTAAG TCTCGCTTGG GCTGCTTTAA "  
       "AGAAAATATA AGTATCTCTA TCCCCTG"TAT TAAGTGTACC TATACCATTC AGAGCGAACC CGACGAAATT   
  
029961 "TGGTAGTCTT TACATTTTCT CTTTCCCTCG TAGTATGGGG AAGA"AGTGGA CTCTAAAA"GT ACTACTAATT "  
       ACCATCAGAA "ATGTAAAAGA GAAAGGGAGC ATCATACCCC TTCTTCACCT GAGATTTTCA TGATGATTAA "  
  
030031 "GAAAAAAAAA AACTGTATTG TTTTTGGTTT ATTTTATAGA TCATTCTGCA AAACTTTT"TT TCTTTTATTT   
       "CTTTTTTTTT" TTGACATAAC AAAAACCAAA TAAAATATCT AGTAAGACGT TTTGAAAAAA AGAAAATAAA   
  
030101 TGAACAGTAA AAAAAAAAGA GTTCGGATTA TAAGTTTTTA AGTGGATCCA TACTTTCGAC ACGAAAGAA"C "  
       ACTTGTCATT TTTTTTT"TCT CAAGCCTAAT ATTCAAAAAT TCACCTAGGT ATGAAAGCTG TGCTTTCTTG "  
  
030171 "ATTAGTGGTT GTCCAATGAG ATACTACGCA TAATAATATA CTACCTCATA ATAAGATAGT ACCTCGGGG"T   
       "TAATCACCAA CAGGTTA"CTC TATGATGCGT ATTATTATAT GATGGAGTAT TATTCTATCA TGGAGCCCCA   
  
030241 AGCCACCCCA CATATTACGT GCTTACCACT TCTTTTATTT ATTATTATTA GTATTTTAGT TATTTTC"AAA "  
       TCGGTGGGGT GTAT"AATGCA CGAATGGTGA AGAAAATAAA TAATAATAAT CATAAAATCA ATAAAAGTTT "  
  
030311 "ATAGGATTTT TTCTTGTTTT AGGGAACTCA TTTCATATCA TGCTTCAAAC GTTAAATAAC GATGGGG"TTT   
       "TATCCTAAAA AAGA"ACAAAA TCCCTTGAGT AAAGTATAGT ACGAAGTTTG CAATTTATTG CTACCCCAAA   
  
030381 GCTAATTCTT TTCGAAATCC GAAGATAAAA AGTTCCCATG GAACCGAACT CCTGGATCAT CTGTCAA"CTG "  
       CGATTAAGAA AAGCTTTAGG CTTCTATT"TT TCAAGGGTAC CTTGGCTTGA GGACCTAGTA GACAGTTGAC "  
  
030451 "CTCAATCAAT TACTTCTTTC GAATGCTAAA AAAAGATTAG TGGCCTTTCG ATTATTTGAT TTCAGAT"TCA   
       "GAGTTAGTTA ATGAAGAAAG CTTACGAT"TT TTTTCTAATC ACCGGAAAGC TAATAAACTA AAGTCTAAGT   
  
030521 TTGCAATCAA CATGAATTAT GAAGCAAAGA AATAGAATTG GGCCACTATG TATATTAGAT TAATATTACA   
       AACGTTAGTT GTACTTAATA CTT"CGTTTCT TTATCTTAAC CCGGTGATAC ATATAATCTA ATTATAATGT "  
  
030591 TATAT"GTAAT TGATATGATA TCTATATATA AATAGAAAGA TATATATTGT ATATTCATCT ATATTCAAAT "  
       "ATATACATTA ACTATACTAT AGA"TATATAT TTATCTTTCT ATATATAACA TATAAGTAGA TATAAGTTTA   
  
030661 "TGATC"AATAT TCAACCTCTA TTTTTATAGA GTACAATTTT ATACACTTCA ATAACAATAA TAGATAGTAT   
       ACTAGTTAT"A AGTTGGAGAT AAAAATATCT CATGTTAAAA TATGTGAAGT TATTGTTATT ATCTATCATA "  
  
030731 GGTAG"AAGGA TTCATATATT TCTTTCTACC ATACTATCGG ATCTCATAGA ATACTTCTCT CGTAGACTAC "  
       "CCATCTTCC"T AAGTATATAA AGAAAGATGG TATGATAGCC TAGAGTATCT TATGAAGAGA GCATCTGATG   
  
030801 "TGATA"ATTCT AGTCCGCCAA TTTCATTTAA GACGCGAAAT TTTAATGCTT TTCATTTTTC TTATCTTCAA   
       ACTATTAAGA TCAGGCGGTT AAA"GTAAATT CTGCGCTTTA AAATTACGAA AAGTAAAAAG AATAGAAGTT "  
  
030871 T"CATTGATAA GAACTAAAAC GGCAAGGTTA ATTCAAATTA ATCACTTTGA CTGATTGTTT TTACGTATAT "  
       "AGTAACTATT CTTGATTTTG CCG"TTCCAAT TAAGTTTAAT TAGTGAAACT GACTAA"CAAA AATGCATATA "  
  
030941 "T"ATAAGTAAA AAGGCGGTCG GAACTAGAAT GAACAGTGCA GTAGCAATAA ATGCGAGAAT ATT"TACTTCC "  
       "ATATTCATTT TTCCGCCAGC CTTGATCTTA CTTGTCACGT CATCGTTATT TACGCT"CTTA TAAATGAAGG   
  
031011 "ATAATATCAT CGTTTCATTT TTTATTCGCA ATAACTCGGG ATTTAATCCC ATAGAGATGA TAA"ATGTTTC   
       TA"TTATAGTA GCAAAGTAAA AAATAAGCGT TATTGAGCCC TAAATTAGGG TATCTCTACT ATTTACAAAG "  
  
031081 GCTTGTAAAT TCAATGGGAT GCATTGTATC TCGATGATAT CGAATCGTAT TAAAATATCA TGAATAACAA   
       "CG"AACATTTA AGTTACCCTA CGTAACATAG AGCTACTATA GCTTAGCATA ATTTTATAGT ACTTATTGTT   
  
031151 TATC"GGAGCT ATCAAATCGA TTCATCGTCG AGAATTGAAT AGTATAACAT AGGAAGATCT TTTATCCATA "  
       ATAG"CCTCGA TAGTTTAGCT AAGTAGCAGC TCTTAACTTA TCATATTGTA TCCTTCTAGA AAATAGGTAT "  
  
031221 "CCGA"ATTTAA ACAAAATGGG ATTCCTGATG CAATCAAGAA TTTCGTTACT TTTTTTATTT ATAATTTTTT   
       "GGCT"TAAATT TGTTTTACCC TAAGGACTAC GTTAGTTCTT AAAGCAATGA AAAAAATAAA TATTAAAAAA   
  
031291 GCAT"TCTTCC TTTTCTATAA TCTACCGCCC TCTTATTCAA TGCAATCATC TGATGAAGTC TCATCAGACT "  
       CGTAAGAA"GG AAAAGATATT AGATGGCGGG AGAATAAGTT ACGTTAGTAG ACTACTTCAG AGTAGTCTGA "  
  
031361 "ACCT"TTACCC TTACATTGTT TATAAACAAA CTCAAGCAAA GAATAGTAGT GAAATTCAAA AA"AGGAAAAA "  
       "TGGAAATG"GG AATGTAACAA ATATTTGTTT GAGTTCGTTT CTTATCATCA CTTTAAGTTT TTTCCTTT"TT "  
  
031431 "GAGGTAGGTT CGAACTAAAC TCCTTCCTTT TTTTGTACTT ATGAAATCTT CTTAAAAAAA AG"AAATAAAA   
       "CTCCATCCAA GCTTGATTTG AGGAAGGAAA AAAACATGAA TACTTTAGAA GAATTTTTTT TCTTTATT"TT   
  
031501 ACGAAACTTA AACTTTCAAA AATTCTTAAT TACCTCACTA AGAGAGATAG ATGGGAT"CCT TGTTTGTATG "  
       TGCTTTGAAT TTGAAAGTTT TTAAGAATTA ATGGAGTGAT TCTCTCTATC TACCCTAGGA ACAAACATAC   
  
031571 "AATATCCTCT TTCCTTGAAT TAGTAGTGGG ACGTATATAT CAAAAGTTAA GTGTGAA"ATT TGAATAAGAT   
       TTA"TAGGAGA AAGGAACTTA ATCATCACCC TGCATATATA GTTTTCAATT CACACTTTAA ACTTATTCTA "  
  
031641 GGATTATTTC AAATTAGTAA TTGAATTTAC TAATTGAGGT TACAAAAAAT CGGAGCCAGA ACGGATATAT   
       "CCT"AATAAAG TTTAATCATT AACTTAAATG ATTAACTCCA ATGTTTTTTA GCCTCGGTCT TGCCTATATA   
  
031711 TTT"GCTTTAA GACGAAAAAT TAGATCAAAG TTTACTATGT AAACTTTATT TTGTATCGGT CCAAATTCCC "  
       AAA"CGAAATT CTGCTTTTTA ATCTAGTTTC AAATGATACA TTTGAAATAA AACATAGCCA GGTTTAAGGG "  
  
031781 "TTT"ATGTATG TGCTTTCCGG AAACGTATAG TACTCTATTT CATTAGAAAA ATCGGGCGTA ATCAACT"AGA "  
       "AAA"TACATAC ACGAAAGGCC TTTGCATATC ATGAGATAAA GTAATCTTTT TAGCCCGCAT TAGTTGA"TCT "  
  
031851 "CCCAGTACGC CTTCGAATCC TGAATATGAT GCTACCAATA ATTGTAGTAA TTCACATATG GCACATA"TGT   
       "GGGTCATGCG GAAGCTTAGG ACTTATACTA CGATGGTTAT TAACATCATT AAGTGTATAC CGTGTAT"ACA   
  
031921 CTTTCTCCCA TCAATTAGTA CTCGTTGAAG AAATAAAAAT TCCCATATTT TTTTGTTGAA AAATGTGGAA   
       GAAAGAGGGT AGTTAATCAT GAGCAACTTC TTTATTTTTA AGGGTATAAA AAAACAACTT TTTACACCTT   
  
031991 AA"AAATCAGA GAGATCCCGT TTGGAATAAA ATTACGTTAT GTTATTTGTT CTCTCTTTCA CAGGAAGGGA "  
       TTTTTAGTCT CTC"TAGGGCA AACCTTATTT TAATGCAATA CAATAAACAA GAGAGAAAGT GTCCTTCCCT "  
  
032061 "AG"AGATTAAG TATTTTTTTT TATTGGATTT GGATCCATCG GGACTGACGG GGCTCGAACC CGCAG"CTTCC "  
       "TCTCTAATTC ATA"AAAAAAA ATAACCTAAA CCTAGGTAG"C CCTGACTGCC CCGAGCTTGG GCGTCGAAGG "  
  
032131 "GCCTTGACAG GGCGGTGCTC TGACCAATTG AACTACAATC CCAGGGAAAT AAAGTGTACA ACATA"TGTTG   
       "CGGAACTGTC CCGCCACGAG ACTGGTTAAC TTGATGTTA"G GGTCCCTTTA TTTCACATGT TGTATACAAC   
  
032201 TTGATTTAAT TTCCTTCAAA CCTTTCTATG TAGATTTTTT ATTCGAATTG TCATATT"CTA CCAGACAGAG "  
       AACTAAATTA AAGGAAGTTT GGAAAGATAC ATCTAAAAAA TAAGCTTAAC AGTATAA"GAT GGTCTGTCTC "  
  
032271 "ACGCGAGTAA TAGAGTAATA GATTTATATG CGCGGAGACA TGGACTTTAG TGTGAGA"AGC ATGGATTAAT   
       "TGCGCTCATT ATCTCATTAT CTAAATATAC GCGCCTCTGT ACCTGAAATC ACACTCT"TCG TACCTAATTA   
  
032341 ATTTATTTTT TTGTTATTGT AAAATTGATT GATAATCAAT CTCAATCTGT CAATGAATAA AAAAAGAGTT   
       TAAATAAAAA AACAATAACA TTTTAACTAA CTATTAGTTA GAGTTAGACA GTTACTTATT TTTTTCTCAA   
  
032411 TTTTT"TATTC TTTCATATCA AGTCAAGCAT TTCTGTAGAA GGACAAATAG GTTATATCAT TTTATGGTGG "  
       AAAAAA"TAAG AAAGTATAGT TCAGTTCGTA AAGACATCTT CCTGTTTATC CAATATAGTA AAATACCACC "  
  
032481 "ATACG"CGAAT TATTGGGCCG AGCTGGATTT GAACCAGCGT AGACATATTG CCAACGAATT TACA"GTCCGT "  
       "TATGCG"CTTA ATAACCCGGC TCGACCT"AAA CTTGGTCGCA TCTGTATAAC GGTTGCTTAA ATGTCAGGCA "  
  
032551 "CCCCATTAAC CGCTCGGGCA TCGACCCGGG AAGAATCAAT TCCAAGCTTG TTGATAATCC ATGA"TCAACT   
       "GGGGTAATTG GCGAGCCCGT AGCTGGG"CCC TTCTTAGTTA AGGTTCGAAC AACTATTAGG TACTAGTTGA   
  
032621 TCCTTTCGTA GTACCCTACC CCCAGGGGAA GTCGAATCCC CGCTGCCTCC TTGAAA"GAGA GATGTCCTGA "  
       AGGAAAGCAT CATGGGA"TGG GGGTCCCCTT CAGCTTAGGG GCGACGGAGG AACTTTCTCT CTACAGGACT "  
  
032691 "ACCACTAGAC GATGGGGGCA TACTTGCCCG ACTGCCATCA TACTATGATC ATAGTA"TGAT CAGTTTTTTG   
       "TGGTGATCTG CTACCCC"CGT ATGAACGGGC TGACGGTAGT ATGATACTAG TATCATAC"TA GTCAAAAAAC "  
  
032761 AAATTGTCAA TAGAATATAA ATATAATGGA CTAATATGAT GCAATTCGAA GGATTTTGCT TTCTTTCATT   
       "TTTAACAGTT ATCTTATATT TATATTACCT GATTATACTA CGTTAAGCTT CCTAAAAC"GA AAGAAAGTAA   
  
032831 ATT"CCATCGA AATTTTTTTA TTTGTGATTT CTATTTATGA ATCGTTCATT CTAATCACCA TTCCCCATAT "  
       TAAGGTAGCT TTAAAAAAAT AAACACTAAA GATAAATACT TAGCAAGTAA GATTAG"TGGT AAGGGGTATA "  
  
032901 "AAT"TCTATAT AGAACTTCTT TTATTTAAAA TTCAATTGCA TTTTTTTTTT ATTTATTTAA TTTCTTTTTT   
       "TTAAGATATA TCTTGAAGAA AATAAATTTT AAGTTAACGT AAAAAAAAAA TAAATA"AATT AAAGAAAAAA   
  
032971 TTATT"TATTT AATTTAAATA ATATACATAA ATTTGCAAAG TTGGAATATA TAGTTATATT ACTTATTATA "  
       AATAAATAAA TTAAATTTAT TATATGTATT TAAACGTTTC AACCTTATAT ATCAATA"TAA TGAATAATAT "  
  
033041 "GAATA"TCAAA TGAAAATAGT GATTAGTAGT GATTAGAAGA AACGTCTAAA ATAAACAAAA ATAATAAATA   
       "CTTATAGTTT ACTTTTATCA CTAATCATCA CTAATCTTCT TTGCAGATTT TATTTGT"TTT TATTATTTAT   
  
033111 TTCAA"AAAAA ATATTCTTTT TTTATTTAAA TAATACGAAG GCTAGTACCC TTCGGGAAGT GATTGGTCTG "  
       AAGTTTTTTT TATAAGAAAA AAATAAATTT ATTATGCTTC CGATCATGGG AAGCCCTTCA CTAACC"AGAC "  
  
033181 "CCATA"TGCTA TAAACGGGAT TAAACTCCAT TTCTCATACT TTCACTCATT GATTCA"CTCA TTGTTAAGAT "  
       "GGTATACGAT ATTTGCCCTA ATTTGAGGTA AAGAGTATGA AAGTGAGTAA CTAAGTGAGT AACAAT"TCTA   
  
033251 "TAGGTTAGGT ATATTTCGAT CTCACACTAA GCCAAGAAAT TCAAAAACGA TAAATT"TAAA AATCTGGGGA   
       ATCCAATCCA TATAAAGCTA GAGTGTGATT CGGTTCTTTA AGTTTTTGCT ATTTAAATT"T TTAGACCCCT "  
  
033321 TAGGGATCAA CAAGTTATTG AAAATTTTTT CCTCGCCAAG TAGAATTGCT TTATCAATGA TTCGGTGAAT   
       "ATCCCTAGTT GTTCAATAAC TTTTAAAAAA GGAGCGGTTC ATCTTAACGA AATAGTTAC"T AAGCCACTTA   
  
033391 GTAT"CTATGT TCAATTCGTG TGTGTACATG TATGAATCAA ATTCATTTCG TTAGGATGGG GCTCATCAAT "  
       CATAGATACA AGTTAAGCAC ACACATGTAC ATACTTAGTT TAAGTAAAGC AATCCT"ACCC CGAGTAGTTA "  
  
033461 "TTAA"TTAGGG ATCGGTCTTA TGATGAAACA ATTCATTGCA TTGATCAAAT CCAATA"TAAA AAACCATTTT "  
       "AATTAATCCC TAGCCAGAAT ACTACTTTGT TAAGTAACGT AACTAGTTTA GGTTAT"ATTT TTTGGTAAAA   
  
033531 "ACCTATACTC ATTAGATAAA TCCAGTTCCT CAATGAGCGA CTATGTGGAT AAGATA"TATT TATGTACATA   
       TGGATATGAG TAATCTATTT AGGTCAAGGA GTTACTCGCT GATACACCTA TTCTATATAA ATACATGTAT   
  
033601 TCTTATTATT TATAATTTAT AATAATTATA TACACTAGAC TCATCGTGGC TAGTGGCTTA CTCAGAAATT   
       AGAA"TAATAA ATATTAAATA TTATTAATAT ATGTGATCTG AGTAGCACCG ATCACCGAAT GAGTCTTTAA "  
  
033671 GAATCAAATA GGC"CTTTTTA ACTCAGTGGT AGAGTAACGC CATGGTAAGG CGTAAGTCAT CGGTTCAAAT "  
       "CTTA"GTTTAT CCG"GAAAAAT TGAGTCACCA TCTCATTGCG GTACCATTCC GCATTCAGTA GCCAAGTTTA "  
  
033741 "CCGATAAGGG GCT"TTGGCTT TTTTCATAAA ACTCCAGCCG TAGTATTCAT ATTTGATT"GA GGGGAGAATA "  
       "GGCTATTCCC CGA"AACCGAA AAAAGTATTT TGAGGTCGGC ATCATAAGTA TAAACTAACT CCCCTCTTAT   
  
033811 "GACGACATAT TTTTTTTTTT TGTAATAAAA AAAGTACAAA CCGTATAATT TTAATTTC"AT TTTTTTATAA   
       CTGCTGTATA AAAAAAAAAA ACATTATTTT TTTCATGTTT GGCATATTAA AATTAAAGTA AAAAAATAT"T "  
  
033881 AAAAGTTATC TTTAATTATA CCAAGTTATT ATTATAATGA ATAATATAAT AGTAATTATA GTT"AGAAACT "  
       "TTTTCAATAG AAATTAATAT GGTTCAATAA TAATATTACT TATTATATTA TCATTAATAT CAATCTTTG"A   
  
033951 "AAAAATTCGA AATTCTATAG TGACACATTT TTTTATTATT TTGATTCTAG AATAAAATTC TAG"ATTCTAT   
       TTTTTAAGCT TTAAGATATC ACTGTGTAAA AAAATAATAA AACTAAGATC TTATTTTAAG ATCTAAGATA   
  
034021 TCTAATCCTT AGAATAGATT CAAATAATCT ATCATGATTC TAATGAATCA TGATAAGTT"G TTTCCTTGAA "  
       AGATTAGGAA TCTTA"TCTAA GTTTATTAGA TAGTACTAAG ATTACTTAGT ACTATTCAAC AAAGGAACTT "  
  
034091 "TTCCAAAATA TTCCTATTTT CCATTATTCC AATCAATTAT AAAGATTCGA AATCAACAA"A AAAAAAGTAA   
       "AAGGTTTTAT AAGGA"TAAAA GGTAATAAGG TTAGTTAATA TTTCTAAGCT TTAGTTGTTT TTTTTTCATT   
  
034161 GTGGACCTAA CCCGTTGAAT CATGACTATA TCCACTATTC TGATATTCAA ATTCGATAA"G GATGAAATTA "  
       CA"CCTGGATT GGGCAACTTA GTACTGATAT AGGTGATAAG ACTATAAGTT TAAGCTATTC CTACTTTAAT "  
  
034231 "GAACAGTGGA TCTTTTTTAT TTCATTTTCA TATTTATTTG GACTCCACAC ACAGGAATC"T GTCGATATTT   
       "CT"TGTCACCT AGAAAAAATA AAGTAAAAGT ATAAATAAAC CTGAGGTGTG TGTCCTTAGA CAGCTAT"AAA "  
  
034301 CCGATTAAAT CCTCTTGTTT GTAGACGTTC ATATAGGAAA AAATTGGTAT TGCTCTT"TCT CCACAGAGAA "  
       "GGCTAATTTA GGAGAACAAA CATCTGCAAG TATATCCTTT TTTAACCATA ACGAGAAAGA GGTGTCT"CTT   
  
034371 "ACGTTTATTC CAAGTCACAA CATATGAGCC GTTTAAAAAT ATCTTTCTTT GATTCCA"AAA CAGAAGATGA   
       TGCAAATAAG GTTCAGTGTT GTATACTCGG CAAATTTTTA TAGAAAGAAA CTAAGGTTTT GTCTTCTACT   
  
034441 AGATAACAGA GGATGAGATA AATTTCTATC GCTATAAGAT AAGATAAGAT ATTTTCTATC TATATAAAAA   
       "TCTATTGTCT CCTACTCTAT TTAAAGATAG CGATATTCTA TTCTATTCTA TAAAAGATAG ATATATTTTT"   
  
034511 AGATA"AATTA TATATCAGAT CATGGCTCCA TGTGCCAAAT ATTTCCATAT TGATACATAT TCAATATATG "  
       TCTATTTAAT ATATAGTCTA GTACCGAGGT ACACGGTTTA TAAAGGTATA ACTATGTATA AGTTATATAC   
  
034581 "TTCCA"ACAAT GTGACGAAGA ATGGAGACGA GAACCGATAC TTTAATTTAC AGTATT"GAGT TTATAATATT "  
       AAGGTTGTTA CACT"GCTTCT TACCTCTGCT CTTGGCTATG AAATTAAATG TCATAACTCA AATATTATAA "  
  
034651 "GATCTAGGTC AGGTTATGGA TTGGATCAAT CAAAAAATGA TTTTTATATT CGAAAC"CCAT TAGAAAGAAG   
       "CTAGATCCAG TCCA"ATACCT AACCTAGTTA GTTTTTTACT AAAAATATAA GCTTTGGGTA ATCTTTCTTC   
  
034721 GGACAGTAGA AATCATACAG AAATGCTAGA CTCGAAGGCC CTGAAAATGC AATGAGGTGT TCGAAAATGG   
       CCTGTCATCT T"TAGTATGTC TTTACGATCT GAGCTTCCGG GACTTTTACG TTACTCCACA AGCTTTTACC "  
  
034791 TTGAAGTTAG TTGAATAGAG GATCACTATG ACTATAGCCG TTGGTAAATT TACCAAAGAC GAAAATGATT   
       "AACTTCAATC A"ACTTATCTC CTAGTGATAC TGATATCGGC AACCATTTAA ATGGTTTCTG CTTTTACTAA   
  
034861 TATTCGATAT TATGGATGAC TGGTTACGGA GGGACCGTTT CGTTTTTGTA GGTTGGTCCG GTCTATTGCT   
       ATAAGCTATA ATA"CCTACTG ACCAATGCCT CCCTGGCAAA GCAAAAACAT CCAACCAGGC CAGATAACGA "  
  
034931 CTTTCCTTGT GCCTATTTTG CCGTCGGAGG TT"GGTTTACA GGTACAACCT TTGTAACTTC ATGGTATACC "  
       "GAAAGGAACA CGG"ATAAAAC GGCAGCCTCC AACCAAATGT CCATGTTGGA AACATTGAAG TACCATATGG   
  
035001 "CATGGATTGG CAAGTTCCTA TTTGGAAGGA TG"CAACTTCT TAACCGCCGC AGTTTCGACT CCTGCTAATA   
       GTACCTAACC GTTCA"AGGAT AAACCTTCCT ACGTTGAAGA ATTGGCGGCG TCAAAGCTGA GGACGATTAT "  
  
035071 GTTTAGCCCA CTCTTTGTTG TTAC"TATGGG GCCCTGAAGC ACAAGGAGAT TTTACTCGTT GGTGTCAATT "  
       "CAAATCGGGT GAGAA"ACAAC AATGATACCC CGGGACTTCG TGTTCCTCTA AAATGAGCAA CCACAGT"TAA "  
  
035141 "AGGTGGTCTG TGGACTTTTG TTGC"TCTCCA CGGTGCTTTC GGACTAATAG GTTTCATGTT ACGTCAATTT   
       "TCCACCAGAC ACCTGAAAAC AACGAGAGGT GCCACGAAAG CCTGATTATC CAAAGTACAA TGCAGTT"AAA   
  
035211 GAACTTGCTC GATCTGTTCA ATTGCGACCT TATAATGCAA TCGCATTCTC GGGCCCAATT GCTGTTTTTG   
       CTTGAACGAG CTAGACAAGT TAACGCTGGA ATATTACGTT AGCGTAAGAG CCCGGGTTAA CGACAAAAAC   
  
035281 TTTCTGTATT CCTGATTTAT CCACTAGGTC AGTCTGGTTG GTTCTTTGCG CCTAGTTTTG GTGTAGCAGC   
       AAAGA"CATAA GGACTAAATA GGTGATCCAG TCAGACCAAC CAAGAAACGC GGATCAAAAC CACATCGTCG "  
  
035351 TATATTTCGA TTCATCCTAT TTTTCCAAGG GTTTCATAAT TGGACATTGA ACCCATTTCA TATGATGGGA   
       "ATATA"AAGCT AAGTAGGATA AAAAGGTTCC CAAAGTATTA ACCTGTAACT TGGGTAAAGT ATACTACCCT   
  
035421 GTTGCGGGGG TATT"GGGCGC TGCTCTGCTA TGCGCTATTC ATGGTGCTAC CGTAGAAAAT ACCTTATTTG "  
       CAACGCCCCC ATAA"CCCGCG ACGAGACGAT ACGCGATAAG TACCACGATG GCATCTTTTA TGGAATAAAC "  
  
035491 "AGGATGGGGA TGGT"GCAAAT ACATTCCGTG CTTTTAACCC AACTCAAGCT GAAGAAACTT ATTCAATGGT   
       "TCCTACCCCT ACCA"CGTTTA TGTAAGGCAC GAAAATTGGG TTGAGTTCGA CTTCTTTGAA TAAGTTA"CCA "  
  
035561 CACTGCTAAC CGCTTTTGGT CCCAAATCTT TGGGGTTGCT TTTTCCAATA AACGTTGGTT ACATTTCTTT   
       "GTGACGATTG GCGAAAACCA GGGTTTAGAA ACCCCAACGA AAAAGGTTAT TTGCAACCAA TGTAAAG"AAA   
  
035631 ATGTTATTTG TACCAGTAAC TGGTTTATGG ATGAGTGCT"C TTGGAGTAGT TGGTCTGGCC CTGAACCTAC "  
       TACAATAAAC ATGGTCATTG ACCAAATACC TACTCACGAG AACCTCATCA ACCAGACCGG GACTTGGAT"G "  
  
035701 "GTGCCTATGA CTTCGTTTCT CAGGAAATCC GTGCAGCGG"A AGATCCGGAA TTTGAGACTT TCTATACCAA   
       "CACGGATACT GAAGCAAAGA GTCCTTTAGG CACGTCGCCT TCTAGGCCTT AAACTCTGAA AGATATGGT"T   
  
035771 AAATATTCTC TTAAACGAAG GTATTCGTGC TTGGATGGCG GCTCAAGATC AGCCTCATGA AAACCTTATA   
       TTTATAAGAG AATTTGCTTC CATAAGCACG AACCTACCGC CGAGTTCTAG TCGGAGTACT TTTGGAATAT   
  
035841 TTCCCTGAGG AGGTTCTACC CCGTGGAAAC GCTCT"TTAAT GGAACTTTAG CTTTAGCTGG TCGTGACCAA "  
       AAGGGA"CTCC TCCAAGATGG GGCACCTTTG CGAGAAATTA CCTTGAAATC GAAATCGACC AGCACTGGTT "  
  
035911 "GAAACCACCG GTTTCGCTTG GTGGGCCGGG AATGC"CCGAC TTATCAATTT ATCCGGTAAA CTACTGGGGG   
       "CTTTGG"TGGC CAAAGCGAAC CACCCGGCCC TTACGGGCTG AATAGTTAAA TAGGCCATTT GATGACCCCC   
  
035981 CTCATGTAGC CCATGCCGGA TTAATCGTAT TCTGGGCCGG AGCAATGAAC CTATTCGAAG TGGCTCATTT   
       GAGTACATCG GGTACG"GCCT AATTAGCATA AGACCCGGCC TCGTTACTTG GATAAGCTTC ACCGAGTAAA "  
  
036051 CGTACCGGAG AAGCACATGT ACGAACAAGG ATTAATTTTA CTTCCCCACC TAGCTACTTT AGGTTGGGGG   
       "GCATGGCCTC TTCGTG"TACA TGCTTGTTCC TAATTAAAAT GAAGGGGTGG ATCGATGAAA TCCAACCCCC   
  
036121 GTAGGTCCTG GTGGGGAAGT TATAGACACC TTTCCTTACT TTGTGTCTGG AGTACTTCAC TTAATTTCCT   
       CAT"CCAGGAC CACCCCTTCA ATATCTGTGG AAAGGAATGA AACACAGACC TCATGAAGTG AATTAAAGGA "  
  
036191 CCGCAGTATT GGGTTTTGGC GGTATTTATC ATGCACTTCT GGGACCCGAG ACCCTTGAAG AATCTTTTCC   
       "GGC"GTCATAA CCCAAAACCG CCATAAATAG TACGTGAAGA CCCTGGGCTC TGGGAACTTC TTAGAAAAGG   
  
036261 ATTCTTCGGT TATGTGTGGA AAGATAGAAA TAAAATGACT ACCATTTTGG GTATTCACTT AATTTTGTTA   
       TA"AGAAGCCA ATACACACCT TTCTATCTTT ATTTTACTGA TGGTAAAACC CATAAGTGAA TTAAAACAAT "  
  
036331 GGTATAGGTG CTTTTCTTCT AGTATTCAAA GC"TCTTTATT TTGGGGGCGT ATATGATACC TGGGCTCCCG "  
       "CC"ATATCCAC GAAAAGAAGA TCATAAGTTT CGAGAAATAA AACCCCCGCA TATACTATGG ACCCGAGG"GC "  
  
036401 "GGGGGGGAGA TGTAAGAAAA ATTACCAACT TG"ACCCTTAG CCCAAGTGTT ATATTTGGTT ATTTACTAAA   
       "CCCCCCCTCT ACATTCTTTT TAATGGTTGA ACTGGGAATC GGGTTCACAA TATAAACCAA TAAATGAT"TT   
  
036471 ATCTCCTTTT GGCGGAGAAG GATGGATTGT TAGTGTGGAC GATTTGGAAG ATATAGTTGG GGGACATGTA   
       TAGAGGAAAA CCGCCTCTTC CTACCTAACA ATCACACCTG CTAAACCTTC TATATCAACC CCCTGTACAT   
  
036541 TGGTTAGGTT CCATTTGTAT ACTTGGTGGA ATTTGG"CATA TCTTAACTAA ACCCTTTGCA TGGGCTCGCC "  
       ACCAATCCAA GGTAA"ACATA TGAACCACCT TAAACCGTAT AGAATTGATT TGGGAAACGT ACCCGAGCGG "  
  
036611 "GCGCACTTGT ATGGTCTGGA GAAGCTTACT TGTCTT"ATAG TTTAGGTGCT TTATCTGTTT TTGGTTTTAT   
       "CGCGTGAACA TACCA"GACCT CTTCGAATGA ACAGAATATC AAATCCACGA AATAGACAAA AACCAAAA"TA "  
  
036681 TGCTTGTTGT TTTGTCTGGT TCAATAATAC TGCTTATCCG AGTGAGTTTT ACGGGCCGAC TGGACCAGAA   
       "ACGAACAACA AAACAGACCA AGTTATTATG ACGAATAGGC TCACTCAAAA TGCCCGGCTG ACCTGGTC"TT   
  
036751 GCTTCTCAAG CTCAAGCATT TACTTTTCTA GTTAGAGACC AACGTCTTGG AGCTAACATT GGATCCGCTC   
       CGAAGAGTTC GAGTTCGTAA ATGAAAAGAT CAATCTCTGG TTGCAGAACC TCGATTGTAA CCTAGGCGAG   
  
036821 AAGGACCTAC TGGTTTAGGT AAATATCTAA TGCGTTCTCC GACCGGAGAA GTCATTTTTG GGGGAGAGAC   
       TTCCTGG"ATG ACCAAATCCA TTTATAGATT ACGCAAGAGG CTGGCCTCTT CAGTAAAAAC CCCCTCTCTG "  
  
036891 TATGCGTTTT T"GGGATCTGC GTGCTCCGTG GTTAGAACCA CTAAGGGGTC CTAATGGTTT GGACTTGAGT "  
       "ATACGCA"AAA ACCCTAGACG CACGAGGCAC CAATCTTGGT GATTCCCCAG GATTACCAAA CCTGAACTCA   
  
036961 "AGACTGAAAA A"AGACATCCA AACCTTGGCA AGAACGACGT TCCGCGGAAT ATATGACCCT GCTCCTTTAG   
       TCT"GACTTTT TTCTGTAGGT TTGGAACCGT TCTTGCTGCA AGGCGCCTTA TATACTGGGA CGAGGAAATC "  
  
037031 GTTCTTTAAA TTCCGTGGGG GGCGTAGCTA CCGAAATTAA TGCAGTCAAT TATGTCTCTC CTAGAAGTTG   
       "CAA"GAAATTT AAGGCACCCC CCGCATCGAT GGCTTTAATT ACGTCAGTTA ATACAGAGAG GATCTTCAAC   
  
037101 GTTAGCTACC TCTCATTTTG TTCTAGGATT CTTCTTATTC GTAGGTCATT TATGGCATGC AGGAAGGGCT   
       CAATCGAT"GG AGAGTAAAAC AAGATCCTAA GAAGAATAAG CATCCAGTAA ATACCGTACG TCCTTCCCGA "  
  
037171 CGTGCAGCTG CCGCAGGATT TGAAAAAGGA ATTGATCGTG ATTTTGAACC TGTTCTGTTC ATGACTCCTC   
       "GCACGTCG"AC GGCGTCCTAA ACTTTTTCCT TAACTAGCAC TAAAACTTGG ACAAGACAAG TACTGAGGAG   
  
037241 TTAACTGAGA "CAGGCGATCT AATGCTTGAA GTAAAAATCA ATTGGATTCC ACCATACATA TCAGGTTGGA "  
       AATTGACTC"T GTCCGCTAGA TTACGAACTT CATTTTTAGT TAACCTAAGG TGGTATGTAT AGTCCAACCT "  
  
037311 "CAGATTGGGT" TATATTTTAA AAGTCTTCCT TTTTCCTTTC TTCATTTCTA TCTAATCTTT TTTTCTGGTT   
       "GTCTAACCC"A ATATAAAATT TTCAGAAGGA AAAAGGAAAG AAGTAAAGAT AGATTAGAAA AAAAGACCAA   
  
037381 TGGCTCGGCT ATCACACCTA G"CCGAGCCAT TCCCTTTTAT GATACTGAGC CGGGCAAACC CAATAAAATA "  
       ACCGAGC"CGA TAGTGTGGAT CGGCTCGGTA AGGGAAAATA CTATGACTCG GCCCGTTTGG GTTATTTTAT "  
  
037451 "AAGAAACAAA TTGATTCGCC G"AACAAAAGG AGAGAGAGGG ATTCGAACCC TCGATAGTTC TTTGTTTCGA   
       "TTCTTTG"TTT AACTAAGCGG CTTGTTTTCC TCTCTCTCCC TAAGCTT"GGG AGCTATCAAG AAACAAAGCT "  
  
037521 ACTATACCGG TTTTCAAGAC CGGAGCTATC "AACCACTCGG CCATCTCTCC GAAAGATAAT TTCTATTTTA "  
       "TGATATGGCC AAAAGTTCTG GCCTCGATAG TTGGTGAGCC GGTAGAG"AGG CTTTCTATTA AAGATAAAAT   
  
037591 "TTTATATTCC ATCCCATCGA ATAGAACATT" GACATATAAG TTGCTACCAT TACTGTCTAT ATTATAGACA   
       AAATATAAGG TAGGGTAGCT TATCTTGTAA CTGTATATTC AACGATGGTA ATGACAGATA TAATATCTGT   
  
037661 AATATAAGGC GTGAATCTAG AAGTCTATCT "TATATACATA TAGATATATA TGATCCAGTA TACCCCTTTT "  
       TTATATTC"CG CACTTAGATC TTCAGATAGA ATATATGTAT ATCTATATAT ACTAGGTCAT ATGGGGAAAA "  
  
037731 "TGTGAAGTAA AAAAAGCTAC CCTCAATCCC" ATGCATGCTA AAGTATTAAA AAAGTAAGAA GTAATATAGA   
       "ACACTTCA"TT TTTTTCGATG GGAGTTAGGG TACGTACGAT TTCATAATTT TTTCATTCTT CATTATATCT   
  
037801 ATCAATGGAT TTATGGTAAA "ATCCCTCCCT GATACATTTT ATCAAAATTT TGGCTGGCTG GTAAAGGGAT "  
       TA"GTTACCTA AATACCATTT TAGGGAGGGA CTATGTAAAA TAGTTTTAAA ACCGACCGAC CATTTCCCTA "  
  
037871 "CAAATGGTAT ATTTTGTTGG" TAGATTGGAG GATTAGAAAC A"TGACTATTG CTTTCCAATT GGCTGTTTTT "  
       "GT"TTACCATA TAAAACAACC ATCTAACCTC CTAATCTTTG TACTGATAAC GAAAGGTTAA CCGACAAAAA   
  
037941 "GCATTAATTG TTACTTCATC AATCTTACTG ATTAGTGTGC C"CGTTGTATT TGCTTCTCCT GATGGTTGGT   
       CGTAATTAAC AAT"GAAGTAG TTAGAATGAC TAATCACACG GGCAACATAA ACGAAGAGGA CTACCAACCA "  
  
038011 "CGGGTAACAA AAATGTTGTA TTTTCCGGTA CATCGTTATG GATTGGATTA GTCTTTCTGG TGGGTATCCT"   
       "GCCCATTGTT TTT"ACAACAT AAAAGGCCAT GTAGCAATAC CTAACCTAAT CAGAAAGACC ACCCAT"AGGA "  
  
038081 TAATTCTCTT ATCTCTTGA"A CCTCTTCGTT CTTGATACAA AAATGAAACA ACCCTCCCCT AATTCTTTCG "  
       "ATTAAGAGAA TAGAGAACTT GGAGAAGCAA GAACTATGTT TTTACTTTGT TGGGAGGGGA TTAAGA"AAGC   
  
038151 "AATTGTGAGA CACATTAAA"A TACAATATAA GTCCTAAAAA TGCAAATAAA GAAAAAAATT TAGAGGGAGG   
       TTAACACTCT GTGTAATTTT ATGTTATATT CAGGATTTTT ACGTTTATTT CTTTTTTTAA ATCT"CCCTCC "  
  
038221 GGTCTCTTCC TTGTATTGTC AAAATTGAAT ATATCT"CACA TAATTCATAT AAATATATAA ATATAATATA "  
       "CCAGAGAAGG AACATAACAG TTTTAACTTA TATAGAGTGT ATTAAGTATA TTTATATATT TATA"TTATAT   
  
038291 "TATTAAAGTA TATAATAATA CTTAGATATT GATAAT"AGAG AAACTATATA TTATATATAA TTATATAGAC   
       ATAATTTCAT ATATTATTAT GAATCTATAA CTATTATCTC TTTGATATAT AATATATATT AATATATCTG   
  
038361 AAAAATATAT AATTAT"CTTT ATTTAGTTAT AAAGATTAAT TCCAAATATT AGAAAAAGAA ATAGTATACT "  
       TTTTTATATA T"TAATAGAAA TAAATCAATA TTTCTAATTA AGGTTTATAA TCTTTTTCTT TATCATATGA "  
  
038431 "ATAAAATATA TTATAT"AATA AATATATAAT TGGTTAAATT TATATATAAA TATATATAAA AATAGCCTAG   
       "TATTTTATAT A"ATATATTAT TTATATATTA ACCAATTTAA ATATATATTT ATATATATTT TTATCGGATC   
  
038501 TATACTCTAA CCTAGTATTA ATAGCCTAGT ATAATAGGCT AGCTCTGCAC TGATCTATAC TATAGATTGC   
       AT"ATGAGATT GGATCATAAT TATCGGATCA TATTATCCGA TCGAGACGTG ACTAGATATG ATATCTAACG "  
  
038571 AGATAAAAAA ATG"CGGATAT AGTTGAATGG TAAAATTTCT CCTTGCCAAG GAGAAGATGC GGGTTCGATT "  
       "TC"TATTTTTT TACGCCTATA TCAACTTACC ATTTTAAAGA GGAACGGTTC CTCTTCTACG CCCAAGCTAA   
  
038641 "CCCGCTATCC GCC"CAGGAT"G AGGATAATGG GTTAATGTTT TTCATTTAAT AGGATAAATT TTTAAGTATA "  
       GGGCGAT"AGG CGGGTCCTAC TCCTATTACC CAATTACAAA AAGTAAATTA TCCTATTTAA AAATTCATAT "  
  
038711 "GTCGACCGTG ATAGTCTAG"T GGTTCTATCC TCCCCCCTTC CTCCCATCTG GAAAGAAAAA AGCGAATATT   
       "CAGCTGG"CAC TATCAGATCA CCAAGATAGG AGGGGGGAAG GAGGGTAGAC CTTTCTTTTT TCGC"TTATAA "  
  
038781 ATATAGTAAT TAATTAATAT AGTAATTAA"T TAATAGTTAC CAAGATCAAA CCTCAATTTT TTAGAAAAAA "  
       "TATATCATTA ATTAATTATA TCATTAATTA ATTATCAATG GTTCTAGTTT GGAGTTAAAA AATC"TTTTTT   
  
038851 "AATTTTGCGG AGACAGGATT TGAACCCGT"G ACTTCAAGGT TATGAGCCTT GCGAGCTACC AAGCTGCTCT   
       TTAAA"ACGCC TCTGTCCTAA ACTTGGGCAC TGAAGTTCCA ATACTCGGAA CGCTCGATGG TTCGACGAGA "  
  
038921 ACCCCGCGAT AAAGAGAAGA "ACTGTGAATT AATAGACAAA CAAAGATTGA ATGCACCCCT ATACCATATC "  
       "TGGGG"CGCTA TTTCTCTTCT TGACACTTAA TTATCTGTTT GTTTCTAACT TACGTGGGGA TATGGTATAG   
  
038991 "TGTACAAATA GAATAGTCCA" TTTATACAGA ACTCGTAAAG GCGGTCCCTC TATGATCATA GCAATTAATA   
       ACATGTTTA"T CTTATCAGGT AAATATGTCT TGAGCATTTC CGCCAGGGAG ATACTAGTAT CGTTAATTAT "  
  
039061 GAAAGGGATA TTTGAATCCT TACCAACTTG ATC"TTGTTGC CCCAGGCAAC AAGCATGCAT GAACCATTTC "  
       "CTTTCCCTA"T AAACTTAGGA ATGGTTGAAC TAG"AACAACG GGGTCCGTTG TTCGTACGTA CTTGGTAAAG "  
  
039131 "ACGAAGTATG TGTCCAGAGA GCCCAAAGTC TCG"ATAGTTA GCTCTCGGTC TTCCGGTTAA AAAACAACGT   
       "TGCTTCATAC ACAGGTCTCT CGGGTTTCAG AGC"TATCAAT CGAGAGCCAG AAGGCCAATT TTTTGTTGCA   
  
039201 CGATGAAGAC GTGTGGG"TGC ACTATTACGC GGTGGGGATT GTAACTTTCC ATGAATTTCC CATTTCTCAC "  
       GCTACTTC"TG CACACCCACG TGATAATGCG CCACCCCTAA CATTGAAAGG TACTTAAAGG GTAAAGAGTG "  
  
039271 "TCAACGATGG AACTTTG"CTT ATTTCGTTTT T"TAAGGATTG ACGAATCAAA TGATATTTTT GTTCCAATTT "  
       "AGTTGCTA"CC TTGAAACGAA TAAAGCAAAA A"ATTCCTAAC TGCTTAGTTT ACTATAAAAA CAAGGTTAAA "  
  
039341 "TTGCCTCTTC TTCTCCCTCT GAATTAAACT T"TTCCTTGCC ATAATGAATC AATTCCTAGT AGTATCAATG   
       "AACGGAGAAG AAGAGGGAGA CTTAATTTGA A"AAGGAACGG TATTACTTAG TTAAGGATCA T"CATAGTTAC "  
  
039411 ATACAAGTCG GATCCTAGAT GTAGAAATAT AAGAAGGAAG ATCCCCTTCT CCATCGAAAG AAATGATATT   
       "TATGTTCAGC CTAGGATCTA CATCTTTATA TTCTTCCTTC TAGGGGAAGA GGTAGCTTTC T"TTACTATAA   
  
039481 TTTGCGGATA CAACACATAC AAAATTAACC AAA"TTTACCT GATGTAGAGG CAATCAAGAA AGCCGCATAA "  
       AAACGCCTAT GTTGTGTATG TTTTAATTGG TTTAAATGGA CTACATCTCC GTTAGTTCTT TCGGCGTATT   
  
039551 "GTAAATATAT AACCTACAGA AAAGTGGGCT AAT"CCAACTA ATCTTGCTTG CACAATGGAA AGAGCCACTG   
       CATTTATATA TTGGATGTCT TTTCACCCGA TTAGGTTGAT TAGAACGAAC GTGTTACCTT TCTCGGTGAC   
  
039621 GTTTATCTCT CCATCGA"ATC AAATTAGCCA AAGGTGTACG TTCGTGAGCC CATGCTAAAG TTTCAATCAA "  
       CAAATAGAGA GGTAGCTTAG TTTAATCGGT TTCCACATGC AAGCACTCGG GTACGATTTC AAAGTTAGTT   
  
039691 "TTCTTGCCAA TATCCGC"GCC AGGAAATTAA GAACATAAAG CCAGTAGCCC AAACAAGATG TCCAAATAAG   
       AAGAACGGTT ATAGGCGCGG TCCTTTAATT CTTGTATTTC GGTCATCGGG TTTGTTCTAC AGGTTTATTC   
  
039761 AACATCCACG CCCAGACTGA TAAA"CTATTC ATACCAAAAG GATTATATCC ATTGATAAGT TGTGAAGAGT "  
       TTGTAGGTGC GGGTCTGACT ATTTGATAAG TATGGTTTTC CTAATATAGG TAACTATTCA ACACTTCTCA   
  
039831 "TTAACCATAG ATAATCTCTT AACC"ATCCCA TCAAATAAGT GGAAGATTCA TTAAACTGTG AAACGTTACC   
       AATTGGTATC TATTAGAGAA TTGGTAGGGT AGTTTATTCA CCTTCTAAGT AA"TTTGACAC TTTGCAATGG "  
  
039901 CTGCCATAAT GTGATGT"GCT TCCAGTGCCA ATAAAAAGTA ACCCATCCAA TGGTATTTAA CATCCAGAAA "  
       "GACGGTATTA CACTACACGA AGGTCACGGT TATTTTTCAT TGGGTAGGTT AC"CATAAATT GTAGGTCTTT   
  
039971 "ACTGCCAAAT AAAAGGC"GTC CCAAGCCGAA ATATCACAAG TACCACCTCG TCCCGGACCG TCGCAAGGAA   
       TGACGGTTTA TTTTCCGCAG GGTTCGGCTT TATAGTGTTC ATGGTGGAGC AGGGCCTGGC AGCGTTCCTT   
  
040041 AACTATAACC AAAATCCTTT TTATCCGGCA TTAATT"TGGA ACCACGTGCA TCTAAAGCAC CTTTTACTAA "  
       TTGATATTGG TTTTAGGAAA AATAGGCCGT AATTAAACCT TGGTGCACGT AGATTTCGTG GAAAATGATT   
  
040111 "GATCAATGTA GTTGTATGTA AACCTAAAGC AATAGC"ATGA TGAACCAAGA AGTCTCCGGG CCCTATTGTT   
       CTAGTTACAT CAACATACAT TTGGATTTCG TTATCGTACT ACTTGGTT"CT TCAGAGGCCC GGGATAACAA "  
  
040181 AAGAATAGTG AATTACTATT C"TCATTAACA GCATTTAACC AACCTGGCAA CCATATGCTT CGACCCGCAT "  
       "TTCTTATCAC TTAATGATAA GAGTAATTGT CGTAAATTGG TTGGACCG"TT GGTATACGAA GCTGGGCGTA   
  
040251 "TGAACGCCGG GCCGCTTGTT G"AAGATAAAA GTACATCGAA GCCATATGAA GTTTTACCAT GAGCGGATTG   
       ACTTGCGGCC CGGCGAACAA CTTCTATTTT CATGTAGCTT CGGTATACTT CAAAATGGTA CTCGCCTAAC   
  
040321 AATCCATTGG GCAAATAT"GG GTTCGATTAA GATTTGTTTT TCCGGAGTAC CAAAAGCAAG CATGACATCA "  
       TTAGGTAACC CGTTTATACC CAAGCTAATT CTAAACAAAA AGGCCTCATG GTTTTCGTTC GTACTGTAGT   
  
040391 "TTATGAACAT AAAGCCCC"AA AGTATGGAAC CCCAGAAAAA GGCTGGCCCA ACTTAAATGG GATATGATAG   
       AATACTTGTA TTTCGGGGTT TCATACCTTG GGGTCTTTTT CCGACCGGGT TGAATTTACC CTATACTATC   
  
040461 CTTCTTTATG GTCTAACATT CTTGCCAATA CATTATC"CTC ATTCTGTTCT GGATTGTAAT CTCTAATAAA "  
       GAAGAAATAC CAGATTGTAA GAACGGTTAT GTAATAGGAG TAAGACAAGA CCTAACATTA GAGATTATTT   
  
040531 "AAATATAGCT CCATGAGCAA AAGCTCCTGT CATAATG"AAT CCTGCTATGT ATTGGTGATG GGTATATAAC   
       TTTATATCGA GGTACTCGTT TTCGAGGACA GTATTACTTA GGACGATACA TAACCACTAC CCATATATTG   
  
040601 GCAGCTTGAG TAGTAAAGTC TTGTGCTATG A"ACGCATAAG CAGGTAAAGC GTACATGTGT TGAGCTACCA "  
       CGTCGAACTC ATCATTTCAG AACACGATAC TTGCGTATTC GTCCATTTCG CATGTACACA ACTCGATGGT   
  
040671 "AGGAAGTAAT AACCCCTAAA GAGGCTAAAG C"AAGACCTAA TTGAAAATGA AGCGAATTAT TAATTGTGTC   
       TCCTTCATTA TTGGGGATTT CTCCGATTTC GTTCTGGATT AACTTTTACT TCGCTTAATA ATTAACACAG   
  
040741 ATAAAGACCT TTATGTC"CAC GCCCCAATCG TCCCCCCGGT GGAATATGTG CTTCTAAAAG ATCTTTTATA "  
       TATTTCTGGA AATACAGGTG CGGGGTTAGC AGGGGGGCCA CCTTATACAC GAAGATTTTC TAGAAAATAT   
  
040811 "CTGTGTCCAA TCCCGAA"GTT AGTTCTATAC ATATGACCAG CAACAAGAAA AATAAATGCA ATAGCTAAAT   
       GACACAGGTT AGGGCTTCAA TCAAGATATG TATACTGGTC GTTGTTCTTT TTATTTACGT TATCGATTTA   
  
040881 GATGATGAGC CATATCAGTC AGCCATA"AAC TTTGGGTTTG CGGATGGAAT CCCCCGAGAA GGGTTAGAAT "  
       CTACTACTCG GTATAGTCAG TCGGTATT"TG AAACCCAAAC GCCTACCTTA GGGGGCTCTT CCCAATCTTA "  
  
040951 "GGCAGTTCCT GCTCCTTCGG AAGTACC"AAA TAAATGATTA TTTGAATCGG GGTTTTGAGC ATAAAGATTC   
       "CCGTCAAGGA CGAGGAAGCC TTCATGGT"TT ATTTACTAAT AAACTTAGCC CCAAAACTCG TATTTCTAAG   
  
041021 CACTGACCTG TAAAAAGTGG T"CCTAATCCT TGGGGATGTG GCAATACATC TAAGAAATTA TTCCATCGAA "  
       GTGACTGGAC ATTTTTCACC AGGATTAGGA ACCCCTACAC CGTTATGTAG ATTCTTTAAT AAGGTAGCTT   
  
041091 "CAGATTCTCC CCTGGATGCA G"GAATAGCGA CATGGACTAA ATGTCCTGTC CAAGCCAAGG AACTTACTCC   
       GTCTAAGAGG GGACCTACGT CCTTATCGCT GTACCTGATT TACAGGACAG GTTCGGTTCC TTGAATGAGG   
  
041161 GAATAGTCCT GACAAATGAT GATTCAGA"CG AGATTCGGCA TTTTTAAACC AGGAAACGCT CGGTTTCCAT "  
       CTTATCAGGA CTGTTTACTA CTAAGTCTGC TCTAAGCCGT AAAAATTTGG TCCTTTGCGA GCCAAAGGTA   
  
041231 "TTCGGTTGTA GATGTAACCA ACCCGCTA"TT AAAGATATCG CAGAAAGAAA TAATAGAAAA AGAGCTCCAG   
       AAGCCAACAT CTACATTGGT TGGGCGATAA TTTCTATAGC GTCTTTCTTT ATTATCTTTT TCTCGAGGTC   
  
041301 TATAAAGATC CCCATTAGTG CGTAAACCGA TTGTATA"CCA CCACTGATAA ACACCAGAAT AAGCGATATT "  
       ATATTTCTAG GGGTAATCAC GCATTTGGCT AACATATGGT GGTGACTATT TGTGGTCTTA TTCGCTATAA   
  
041371 "CACTGGGCCA AGAGCACCAC CTCGAGTAAA AGCTTCG"ACG GCCGGTTGAC CAAAATGAGG ATCCCAAATT   
       GTGACCCGGT TCTCGTG"GTG GAGCTCATTT TCGAAGCTGC CGGCCAACTG GTTTTACTCC TAGGGTTTAA "  
  
041441 GCATGAGCAA TAGGTCTTA"C ATGTAAAGGG TCCTGTACCC ATGCTTCAAA ATTTCCTTGC CAAGCTACAT "  
       "CGTACTCGTT ATCCAGA"ATG TACATTTCCC AGGACATGGG TACGAAGTTT TAAAGGAACG GTTCGATGTA   
  
041511 "GAAACAGATT TCCGGAAGT"C CACAGAAAAA TTATTGCTAA TTGCCCGAAA TGAGAAGCAA AAATATTCTG   
       CTTTGTCTAA AGGCCTTCAG GTGTCTTTTT AATAACGATT AACGGGCTTT ACTCTTCGTT TTTATAAGAC   
  
041581 ATAAAGACGT TCTTCAGTAA TATCATCATG ACTTTCG"AAG TCATGCGCGG TAGCAATACC AAACCAAATA "  
       TATTTCTGCA AGAAGTCATT ATAGTAGTAC TGAAAGCTTC AGTACGCGCC ATCGTTATGG TTTGGTTTAT   
  
041651 "CGACGAGTAG TGGGGTCCTG AGCTAAGCCT TGGCTAA"ACC TTGGAAATCT TAGTGCCATA ATGCCTTTCA   
       GCTGCTCATC ACCCCAGGAC TCGATTCGGA ACCGATTTGG AACCTTTAGA ATCACGGTAT TACGGAAAGT   
  
041721 AATCCTCCTA GCCATTATCC GACTGCAAT"A ATTCTTGCTA AGAAGAACGC CCATGTTGTG GCAATTCCAC "  
       TTAGGAGGAT CGGTAATAGG CTGACGTTAT TAAGAACGAT TCTTCTTGCG GGTACAACAC CGTTAAGGTG   
  
041791 "CCAGAAGGTA ATGGGTTACT CCTACAGCA"C GTCCTTGGAC AATGCTCAAG GCTCTAGGCT GAGTAGCAGG   
       GGTCTTCCAT TACCCAATGA GGATGTCGTG CAGGAACCTG TTACGAGTTC CGAGATCCGA CTCATCGTCC   
  
041861 AGCAACTTTT AATTTAT"TAT GAGCCCAAAC GATGGATTCA ATAAGTTCTT GCCAATAACC ACGCCCGCTG "  
       TCGTTGAAAA TTAAATAATA CTCGGGTTTG CTACCTAAGT TATTCAAGAA CGGTTATTGG TGCGGGCGAC   
  
041931 "AATAGAAACA TTAAACT"AAA AGCCCATACA AAATGAGCAC CTAGGAAAAA CAGGCCATAT GCAGATAATG   
       TTATCTTTGT AATTTGATTT TCGGGTATGT TTTACTCGTG GATCCTTTTT GTCCGGTATA CGTCTATTAC   
  
042001 AAGAACCATA AGACTGAATT ACCTGGGA"TG CCTGTGCCCA TAAGAAATCG CGAAGCCACC CATTAATAGT "  
       TTCTTGGTAT TCTGACTTAA TGGACCCTAC GGACACGGGT ATTCTTTAGC GCTTCGGTGG GTAATTATCA   
  
042071 "AATAGAACTC TGCGCAAAGT TTCCTCCC"GT AATATGAGTT AGTACCCCTT GATCACTTAC ACTACCCCAG   
       TTATCTTGAG ACGCGTTTCA AAGGAGGGCA TTATACTCAA TC"ATGGGGAA CTAGTGAATG TGATGGGGTC "  
  
042141 ACATCTGACT GCATTTTCCG ACTGAAATGG AATATTA"CTA CAGAAATGGA ATTGTACATC CAAAATAGCC "  
       "TGTAGACTGA CGTAAAAGGC TGACTTTACC TTATAATGAT GT"CTTTACCT TAACATGTAG GTTTTATCGG   
  
042211 "CTAAGAAGAC ATGATCCCAA GCGGATACTT GACATGT"ACC CCCTCTTCCC GGTCCATCAC AAGGGAAACG   
       GATTCTTCTG TACTAGGGTT CGCCTATGAA CTGTACATGG GGGAGAAGGG CCAGGTAGTG TTCCCTTTGC   
  
042281 AAAACCAAGA TTTGCTTTAT CCGGTATCA"A ACGCGAGCTA CGAGAAAATA GAACACCTTT CAGGAGTATC "  
       TTTTGGTTCT AAACGAAATA GGCCATAGTT TGCGCTCGAT GCTCTTTTAT CTTGTGGAAA GTCCTCATAG   
  
042351 "AATACCGTTA CATGAATCGT AAATGCATG"A ATATGGTGTA CCAAAAAATC TGCGGTTCCT AACGGAATAG   
       TTATGGCAAT GTACTTAGCA TTTACGTACT TATACCACAT GGTTTTTTAG ACGCCAAGGA TTGCCTTATC   
  
042421 GTAACAAAGC AACTTTGCCG CCCACTGCCA "CTAAATCACC ACCCCCCCAA GTCAAACTGG TGCCTGTTGT "  
       CATTGTTTCG TTGAAACGGC GGGTGACGGT GATTTAGTGG TGGGGGGGTT CAGTTTGACC ACGGACAACA   
  
042491 "TGCACCGGGG GCCGTTATCC TAGGTGCTAA" AGCATGGGTG TTTGTATCCA TTGAGCAAAG ACGGGGTTGT   
       AC"GTGGCCCC CGGCAATAGG ATCCACGATT TCGTACCCAC AAACATAGGT AACTCGTTTC TGCCCCAACA "  
  
042561 AATTGTATAG CGGTATCTGA "AAACATATCT TGTGGACGCC CTAAAGCACT CATGGTATCG TTATGAATAT "  
       "TT"AACATATC GCCATAGACT TTTGTATAGA ACACCTGCGG GATTTCGTGA GTACCATAGC AATACTTATA   
  
042631 "ACAAACCAAA ACTGTGAAAG" CCTAAAAATA TACACACCCA GTTCAGATGT GATATGATTG CATCACGATG   
       TGTTTGGTTT TGACACTTTC GGATTTTTAT ATGTGTGGGT CAAGTCTACA CTATACTAAC GTAGTGCTAC   
  
042701 CCTAAGTACA CGATCTAATA GATC"GTTGTA GCGAGTAGTT GGATCATAGT CTCTTACCAT AAAAATGGCT "  
       GGATTCATGT GCTAGATTAT CTAGCAACAT CGCTCATCAA CCTAGTATCA GAGAATGGTA TTTTTACCGA   
  
042771 "GCATGCGCAG CAGCACCAAC TATG"AGAAAT CCACCAATCC ACATGTGATG TGTGAACAAT GAAAGTTGTG   
       CGTACGCGTC GTCGTGGTTG ATACTCTTTA GGTGGTTAGG TGTACACTAC ACACTTGTTA CTTTCAACAC   
  
042841 TACCATAGTC AGTAGCTAGA TATGGAT"ACG GGGGCATTGC ATACATATGG TGAGCTACAA TAATGGTTAA "  
       ATGGTATCAG TCATCGATCT ATACCTATGC CCCCGTAACG TATGTATACC ACTCGATGTT ATTACCAATT   
  
042911 "AGAGCCTAAC ATAGCTAGGT TAATAGA"TAA TTGAGCATGC CATGACGTTG TTAGGATCTC ATATAGTCCT   
       TCTCGGATTG TATCGATCCA ATTATCTATT AACTCG"TACG GTACTGCAAC AATCCTAGAG TATATCAGGA "  
  
042981 TTATGGCCCT GGCCTGT"AAA TGGACCTTTA TGAGCCTCTA AAATATCTTT TATACCATGA CCAATGCCCC "  
       "AATACCGGGA CCGGACATTT ACCTGGAAAT ACTCGG"AGAT TTTATAGAAA ATATGGTACT GGTTACGGGG   
  
043051 "AGTTGGTCCT ATACATG"TGA CCCGCTATCA GGAAAAGAAT TGCAATAGCT AAATGATGGT GTGCAATATC   
       TCAACCAGGA TATGTACACT GGGCGATAGT CCTTTTCTTA ACGTTATCGA TTTACTACCA CACGTTATAG   
  
043121 GGTTAACCAT AGACCCCCGG TTACTG"GATC TAACCCTCCG CGAAAAGTAA GAAATTCCGC ATATTTTGAC "  
       CCAATTGGTA TCTGGGGGCC AATGACCTAG ATTGGGAGGC GCTTTTCATT CTTTAAGGCG TATAAAACTG   
  
043191 "CAATTCAAAG TGAAAAATGG GGTTGC"TCCC TCAGCAAAAC TAGGATAAAG TTGCGCCAAA AGATCCCGAT   
       GTTAAGTTTC ACTTTTTACC CCAACGAGGG AGTCGTTTTG ATCCTATTTC AACGCGGTTT TCTAGGGCTA   
  
043261 TCAAGATAAA TTCATGA"GGA AGTGGAATCT CTTTAGGATC TACTCCAGCA TTTAGAAATT GGTTAATCGG "  
       AGTTCTATTT AAGTACTCCT TCACCTTAGA GAAATCCTAG ATGAGGTCGT AAATCTTTAA CCAATTAGCC   
  
043331 "TAAAGATACG TGGACTT"GAT GTCCCGCCCA AGAAAGAGAC CCAAGTCCTA GCAGACCCGC TAAATGGTGA   
       ATTTCTATGC ACCTGAACTA CAGGGCGGGT TCTTTCTCTG GGTTCAGGAT CGTCTGGGCG ATTTACCACT   
  
043401 TTCAACATGG ATTCTACAT"C TTGGAACCAA GCCAATTTTG GAGCAGCCTT GTGATAATGA AACCAACCAG "  
       AAGTTGTACC TAAGATGTAG AACCTTGGTT CGGTTAAAAC CTCGTCGGAA CACTATTACT TTGGTTGGTC   
  
043471 "CAAAAAGCAT TAAGGCTGC"A AAGACTAATG CACCAATTGC GGTACAATAG AGTTGTAATT CATTAGTTAT   
       GTTTTTCGTA ATTCCGACGT TTCTGATTAC GTGGTTAACG CCATGTTATC TCAACATTAA GTAATCAATA   
  
043541 TCCAGATGCT CGCCACATCT GAAAAAACCC AGATGT"TATT TGTATTCCTC GAAAACCCCC GCCTACATCA "  
       AGGTCTACGA GCGGTGTAGA CTTTTTTGGG TCTACAATAA ACATAAGGAG CTTTTGGGGG CGGATGTAGT   
  
043611 "CCATTCAATA TTTCTTGGCC CACTATTGGC CAAACC"ACCT GGGCACTAGG GCCAATGTGA GTAGGATCAC   
       GGTAAGTTAT AAAGAACCGG GTGATAACCG GTTTGGTGGA CCCGTGATCC CGGTTACACT CATCCTAGTG   
  
043681 CTAGCCATGC TTCATAATTG "GAAAAACGAG CACCGTGGAA ATACATCCCA CTCAGCCAAA GAAAGATGAT "  
       GATCGGTACG AAGTATTAAC CTTTTTGCTC GTGGCACCTT TATGTAGGGT GAGTCGGTTT CTTTCTACTA   
  
043751 "GGAGAGTTGG CCGAAATGGG" CACTAAAGAC TTTTCGAGAG ATCTCCTCCA AATCACTAGT ATGGCTATCG   
       CCTCTCAACC GGCTTTACCC GTGATTTCTG AAAAGCTCTC TAGAGGAGGT TTAGTGATCA TACCGATAGC   
  
043821 AAATCGTGAG CATCAGCATG AAGGTT"CCAG ATCCAAGTGG TAGTATCAGG ACCCTTAGCT ATTGTTCTCG "  
       TTTAGCACTC GTAGTCGTAC TTCCAA"GGTC TAGGTTCACC ATCATAGTCC TGGGAATCGA TAACAAGAGC "  
  
043891 "AGAAATGACC CGGTCTGGCC CAGTCC"TCGA AAGAAGTTTT TATGGGATCC CTATCTACCA AAATTTTGAC   
       "TCTTTACTGG GCCAGACCGG GTCAGG"AGCT TTCTTCAAAA ATACCCTAGG GATAGATGGT TTTAAAACTG   
  
043961 TTCTGGTTCC GGCGAACGAA TAATCATTGA GT"CCTCCTCT TTCCGGACAA CACATACAAA GAGACCCGCC "  
       AAGACCAAGG CCGCTTGCTT ATTAGTAACT CAGGAG"GAGA AAGGCCTGTT GTGTATGTTT CTCTGGGCGG "  
  
044031 "AATAGTTAAG TAATTAGTGA ACCTATGAGA GA"TTTTTCTA CTTAGTTTCT TTCTCTGCTA TCTCCCATCT   
       "TTATCAATTC ATTAATCACT TGGATACTCT CTAAAA"AGAT GAATCAAAGA AAGAGACGAT AGAGGGTAGA   
  
044101 ATTTTATTTT TTTTTTATTT AGTTATTCAC TAGAGCA"ATT ATGATCTAGA AGTTGATCCG GGGCAAGTGT "  
       TAAAATAAAA AAAAAATAAA TCAATAAGTG ATCTCGTTAA TACTAGAT"CT TCAACTAGGC CCCGTTCACA "  
  
044171 "TCGGATCTAT TATGACATAG CCATGAGGCG CTCAACG"GAC CTTTTGAATC TTATAAAACC TTTTTCCGGG   
       "AGCCTAGATA ATACTGTATC GGTACTCCGC GAGTTGCCTG GAAAACTT"AG AATATTTTGG AAAAAGGCCC   
  
044241 TTTTGAATTG ACACAAATTC AATTTTTTTG TA"GAACCTAA TGTATTCACA TATCTCGATT AGAAGTTCCT "  
       AAAACTTAAC TGTGTTTAAG TTAAAAAAAC ATCTTG"GATT ACATAAGTGT ATAGAGCTAA TCTTCAAGGA "  
  
044311 "AAATGGAACT ACTTTATGTC TTACATAGAA CA"TCTTTTTT TATATAAAAT ACATTCTCTA ATTCTAATGC   
       "TTTACCTTGA TGAAATACAG AATGTATCTT GTAGAA"AAAA ATATATTTTA TGTAAGAGAT TAAGATTACG   
  
044381 ATCTCTGTAT CGTTTACCCC TACAAAATA"C CAAAAAAAAT TGAACGATCT TAGATTAGAA GGGATATAAT "  
       TAGAGACATA GCAAATGGGG ATGTTTTATG GTTTTTTTTA ACT"TGCTAGA ATCTAATCTT CCCTATATTA "  
  
044451 "GAAATTCCTT GATTGGTTCT TCACTGATA"A ATTATCCCTT TTATTTGATT TGACTGATGC GGACAACAAA   
       "CTTTAAGGAA CTAACCAAGA AGTGACTATT TAATAGGGAA AAT"AAACTAA ACTGACTACG CCTGTTGTTT   
  
044521 CAATTATAAC AAATATCAAA ATATC"CAAAT TTCTAACTAA TACTAGCTAA ATATAATAGC TATATATTTA "  
       GTTAATATTG TTTATAGTTT TATAGGTTTA AAGATTGATT ATGATCGATT TATATT"ATCG ATATATAAAT "  
  
044591 "TACAAAATAT AAAAAATTCA TTCAT"ATTTG AAAATATTTA TCAAAAGAAA AGTTTAATAA TAGCATAGCA   
       "ATGTTTTATA TTTTTTAAGT AAGTATAAAC TTTTATAAAT AGTTTTCTTT TCAAAT"TATT ATCGTATCGT   
  
044661 GTTAATAATT AAAATAAAGA AAAAAAAAAA ATAGAGT"TTT CTTATTTGAA ACGCCTTGTG ATCTTCAACC "  
       CAATTATTAA TTTTATTTCT TTTTTTTTTT TATCTCAAAA GAATAAACTT TGCGGAACAC TAGAAGTTGG   
  
044731 "AATTTTGGGC TTCAATATAA TTACCGGGAG TAAGCGC"TAT AGCTTGTTTC CAATACTCAG CGGCTTGATT   
       TTAAAACCCG AAGTTATATT AATGGCCCTC ATTCGCGATA TCGAACAAAG GTTA"TGAGTC GCCGAACTAA "  
  
044801 GAACCAAGCC TCCGCAATTT CAGAATC"TCC CTGTCGAATG GCCTGTTCTC CCCGGTCGGA ATAGGCAGGT "  
       "CTTGGTTCGG AGGCGTTAAA GTCTTAGAGG GACAGCTTAC CGGACAAGAG GGGC"CAGCCT TATCCGTCCA   
  
044871 "AAATTCCTTC CCTTAGAACC GTACTTG"AGA GTTTCCTACC TCATACGGCT CAACAGTCAA TTCTTTTGCT   
       TTTAAGGAAG GGAATCTTGG CATGAACTCT CAAAGGATGG "AGTATGCCGA GTTGTCAGTT AAGAAAACGA "  
  
044941 GTCCCATATT TTAAAAAATT "TACCATATCT AATCTAATTG AATGAAATTT TTCATAGATC TAGGCCATTT "  
       "CAGGGTATAA AATTTTTTAA ATGGTATAGA TTAGATTAAC" TTACTTTAAA AAGTATCTAG ATCCGGTAAA   
  
045011 "TTTTTTTCTC GAGTTAAGCA" AAAGAGGTTA ATTATATAAG TTTCAAACTT CAATTTTGCT GAATAATTAA   
       AAAAAAAGAG CTCAATTCGT TTTCTCCAAT TAATATATTC AAAGTTTGAA GTTAAAA"CGA CTTATTAATT "  
  
045081 ATAAGTTTTA TCTTTTCTCC CACCTTCAGA AAAATAA"AGC ATAGGCATTC CACCCACTAT CGTTACAATT "  
       "TATTCAAAAT AGAAAAGAGG GTGGAAGTCT TTTTATTTCG TATCCGTAAG GTGGGTG"ATA GCAATGTTAA   
  
045151 "TTCTGAAAGA TAACTGTCTC GGTTTCATCT AGAAATT"TAT ATAGAATCCT TGAAAAAGAC TTTCCTTCAT   
       AAGACTTTCT ATTGACAGAG CCAAAGTAGA TCTTTAAATA TATCTTAGGA AC"TTTTTCTG AAAGGAAGTA "  
  
045221 AAGAAAGAAA AGACTTACTG TCTTT"GGGAT CTGATGCTAC ACCGCTGCTC AATATTGTAG GGGATCCACC "  
       "TTCTTTCTTT TCTGAATGAC AGAAACCCTA GACTACGATG TGGCGACGAG TT"ATAACATC CCCTAGGTGG   
  
045291 "CTATTACATA AGTGGATTCC TTCAT"TTTGA TCTTATATCA TGATATAAGT AAGCAGTTTT TATTGTATCG   
       GATAATGTAT TCACCTAAGG AAGTAAAACT AGAATATAGT "ACTATATTCA TTCGTCAAAA ATAACATAGC "  
  
045361 GCCAAAAACC TGACTAA"TTG ATCTTTACGG TGCTTCTTCT ATCAATTAGA TCCTTTATCC ATAGAATAAA "  
       "CGGTTTTTGG ACTGATTAAC TAGAAATGCC ACGAAGAAGA" TAGTTAATCT AGGAAATAGG TATCTTATTT   
  
045431 "GTATCTAGGC ATACCTA"TTT CTTCATATTT CTATTTCTAT GAAGTTTCTT TCTTTGCTAC AGCTGATACA   
       CATAGATCCG TATGGATAAA GAAGTATAAA GATAAAGATA "CTTCAAAGAA AGAAACGATG TCGACTATGT "  
  
045501 AATCGTTAGT TTGGACAATA CATATGTAGA AA"GCCTATTT TTTTTTTAGT ATTTATTAGC GAATTTTCTC "  
       "TTAGCAATCA AACCTGTTAT GTATACATCT TTCGGATAAA" AAAAAAATCA TAAATAATCG CTTAAAAGAG   
  
045571 "TTTTTTTTCT TTCTATAGTG GAGATAGTCG CA"CGTAATGA CAGATCACAG CCATATTATT AAAAGCTTGT   
       AAAAAAAAGA AAGATATCAC CTCTATCAGC GTGCATTACT GTCTAGTGTC GGTATAATAA TTTTCGAACA   
  
045641 GGTAAGAACG GGTTTCGTTC "TAGTGCCCGA AAATAATATT CCAAAGCTTT CGTATGTTCT CCGTTCCTTG "  
       CCATT"CTTGC CCAAAGCAAG ATCACGGGCT TTTATTATAA GGTTTCGAAA GCATACAAGA GGCAAGGAAC "  
  
045711 "TGTGGATAAG ACCTATGTTA" TAGAGTATAT AACTTCGATC ATAGGGATCG ATTTCTAGTC GCATAGCTTC   
       "ACACC"TATTC TGGATACAAT ATCTCATATA TTGAAG"CTAG TATCCCTAGC TAAAGATCAG CGTATCGAAG "  
  
045781 ATAATAATTC TGTAGAGCTT CCGCAT"AATT TCCTTCGGAT TGAGCCGACA TCCGTTACGG TCGTTCCTTA "  
       "TATTATTAAG ACATCTCGAA GGCGTATTAA AGGAAG"CCTA ACTCGGCTGT AGGCAATGCC AGCAAGGAAT   
  
045851 "TTCCATTCAA AGAATCTCCG TTCCAG"AACC GTACGTGAGA TTTTCATCTC ATACGGCTCC CCCTTTATGT   
       AAGGTAAGTT TCTTAGAGGC AAGGTCTTGG CATGCACTCT AAAAGT"AGAG TATGCCGAGG GGGAAATACA "  
  
045921 GATGCGCATA ATGAGATGAG AATAATACAT GAAATCA"AAA AACATTGAAA TACTCTCATT ATGAACTCAT "  
       "CTACGCGTAT TACTCTACTC TTATTATGTA CTTTAGTTTT TTGTAA"CTTT ATGAGAGTAA TACTTGAGTA   
  
045991 "GGGACTAGCG TTTTTACAAA AAATCTCTAG CCAACCT"TCC TGTAAAAGAT CTTTTCTTAA CATCAAGCAT   
       CCCTGATCGC AAAAATGTTT TTTAGAGATC GGTTGGAAGG ACATTTTCTA GAAAAGAATT GTAG"TTCGTA "  
  
046061 GGTGTTACTA GATAAAAATA AAAATGGTAA "CTCTAACAAT TTCTTTGTCC TCAACGCCTC TAAATTTCCA "  
       "CCACAATGAT CTATTTTTAT TTTTACCATT GAGATTGTTA AAGAAACAGG AGTTGCGGAG ATTT"AAAGGT   
  
046131 "GGAATTAGTC ACTTCAACAG TCTTCGATGG" TTATACAGGT ATCCAAAATA CAAACGAGAT GGATGTTTGT   
       CCTTAATCAG TGAAGTTGTC AGAAGCTACC AATATGTCCA TAGGTTT"TAT GTTTGCTCTA CCTACAAACA "  
  
046201 TGTCCCAACC ATTTTTCTAA GTTCCGATCC CGATAA"GGAA AAGGTATAAT TTATAACAAA GTTTTCGTGT "  
       "ACAGGGTTGG TAAAAAGATT CAAGGCTAGG GCTATTCCTT TTCCATA"TTA AATATTGTTT CAAAAGCACA   
  
046271 "TGTTGATTCC TAGGTGTAGT GCTTCTTCCC CTCTGC"TACC TATTTTTACT AGTGAATATT TTGACTAGTG   
       ACAACTAAGG ATCCACATCA CGAAGAAGGG GAGACGATGG ATAAAAATGA TCACTTATA"A AACTGATCAC "  
  
046341 GAGTAGGGTT GACTTAAT"CC ATAGGTTACA CCTTTCGCTT AATACTAGAA TCGACAATTA AAGCATCTGA "  
       "CTCATCCCAA CTGAATTAGG TATCCAATGT GGAAAGCGAA TTATGATCTT AGCTGTTAA"T TTCGTAGACT   
  
046411 "GGTTACATTA ATCGGGGA"TA CACGACAGAA GGAATTTTTT TATTTTCAAA TTGCACCTCC AAGAAGCGTA   
       CCAATGTAAT TAGCCCCTAT GTGCTGTCTT CCTTAAAAAA ATAAAA"GTTT AACGTGGAGG TTCTTCGCAT "  
  
046481 GATTTATTTC AATTATT"GTT TTCTTTCTAT CCCGAATACC GTGTCTTTCT ACTAAGACGG AGAGCGCTAA "  
       "CTAAATAAAG TTAATAACAA AAGAAAGATA GGGCTTATGG CACAGA"AAGA TGATTCTGCC TCTCGCGATT   
  
046551 "AGAAAATTGA AAAAAAA"ATC AAATCGCACC ATCTCTGTAA TAGGTGAATG CCTCTTTTTC CCCGGAAGTT   
       TCTTTTAACT TTTTTTTTAG TTTAGCGTGG TAGAGACATT AT"CCACTTAC GGAGAAAAAG GGGCCTTCAA "  
  
046621 GTCGGAATTA TTCGTAATAA GATATTGGCT ACAAT"TGAAA AGGTCTTATC GATAAAATTT CCATTTATCC "  
       "CAGCCTTAAT AAGCATTATT CTATAACCGA TGTTAACTTT TC"CAGAATAG CTATTTTAAA GGTAAATAGG   
  
046691 "CACCAGATCT AGGCATCGGT AACAACCCCA TTCTA"TAATT TCTTTTAATT ACCTCTCGTG AGAAAATGAT   
       GTGGTCTAGA TCCGTAGCCA TTGTTGGGGT AAGATATTAA AGAAAATTAA TGGAGAGCAC TCTTTTACTA   
  
046761 CCCACAAACA AAGGAATTGC ATAGTACGAA ATAA"CATAAA AACGGATTCA TTAAAAAATC CCACTCGATA "  
       GGGTGTTTGT TTCCTTAACG TATCATGCTT TATT"GTATTT TTGCCTAAGT AATTTTTTAG GGTGAGCTAT "  
  
046831 "TTCAAATTGT TTCTTTTTGA TTTCACAGGA ATCA"ATAAAA CAGAAGAAAT ATTTCAATCC GGTTAAATTT   
       "AAGTTTAACA AAGAAAAACT AAAGTGTCCT TAGT"TATTTT GTCTTCTTTA TAAAGTTAGG CCAATTTAAA   
  
046901 CATCCAAATG TAGTAGGATC AGAA"TGAAGA GAACTATTCT GATTTCAGCG AAGTTGAAGA AAAAAAGAAT "  
       GTAGGTTTAC ATCATCCTAG TCTT"ACTTCT CTTGATAAGA CTAAAGTCGC TTCAACTTCT TTTTTTCTTA "  
  
046971 "TTTATTTATC TGACAGATTG GGGG"TAATTC TAGAAGTTTT TTGATTGAAT TATGATACAA AAAAGAGCAA   
       "AAATAAATAG ACTGTCTAAC CCCC"ATTAAG ATCTTCAAAA AACTAACTTA ATACTATGTT TTTTCTCGTT   
  
047041 AAAGAATCGA ATAATCAT"TC TATGATTCAT AAATTGTGAC TAGATATATG TATGGGATAA ACAGTTGTTG "  
       TTTCTTAGCT TATTAGTA"AG ATACTAAGTA TTTAACACTG ATCTATATAC ATACCCTATT TGTCAACAAC "  
  
047111 "GTGAAAAATC GAAAACTG"TA AAATAAAAGT CTAATTTTTA GAAAAAAGGA ATCATAGTTT AAAAAACTAA   
       "CACTTTTTAG CTTTTGAC"AT TTTATTTTCA GATTAAAAAT CTTTTTTCCT TAGTATCAAA TTTTTTGATT   
  
047181 ATAGAATCAT GAGCTAAA"AG TATCCATTAA ACAGAGTAAA AAAAAATTAT TTGTATAGTT AGAATTGGTT "  
       TATCTTAGTA CTCGATTT"TC ATAGGTAATT TGTCTCATTT TTTTTTAATA AACATATCAA TCTTAACCAA "  
  
047251 "GGACGTGCCT ATAAAAAA"AA AAATTGAATA TTAATGACTC ACTATTAACT CGGTTTCTGG GCCATAATCA   
       "CCTGCACGGA TATTTTTT"TT TTTAACTTAT AATTACTGAG TGATAATTGA GCCAAAGACC CGGTATTAGT   
  
047321 TTCTGTAGGA GAGATGGCCG A"GTGGTTGAA GGCGTAGCAT TGGAACTGCT ATGTAGGCTT TTGTTTACCG "  
       AAGACATCCT CTCTACCGGC TCACCAACTT C"CGCATCGTA ACCTTGACGA TACATCCGAA AACAAATGGC "  
  
047391 "AGGGTTCGAT CCTTCTCTTC C"GTACTAATT CACAATGTTA CT"GACCACAA TGTATCAATC AAATAACAAT "  
       "TCCCAAGCTA GGAAGAGAAG GCATGATTAA G"TGTTACAAT GACTGGTGTT ACATAGTTAG TTTATTGTTA   
  
047461 "GGATACTATT ATTCCAACGG TTAGACCTTT ATGGATTCTT TA"TTTCGAAT TTTTTTGGTA CGGAAAGGAA   
       CCTATGATAA TAAGGTTG"CC AATCTGGAAA TACCTAAGAA ATAAAGCTTA AAAAAACCAT GCCTTTCCTT "  
  
047531 AAGACTGGAA AGAATGGACA AGAGATGAAA AT"CCCCCTAC CGATCAATGA TAGATGAATG ATAGAAAAAT "  
       "TTCTGACCTT TCTTACCT"GT TCTCTACTTT TAGGGGGATG GCTAGTTACT ATCTACTTAC TATCTTTTTA   
  
047601 "TTCCGGATCA AAACCTTACT TTCCTTCGGT CT"TTTTACTT AGACTTCGGC GAAGGGGAGG ACGAAATTGT   
       AAGGCCTAGT TTTGGAATGA AAGGA"AGCCA GAAAAATGAA TCTGAAGCCG CTTCCCCTCC TGCTTTAACA "  
  
047671 CCGAACCCTT GTTTTTTAGT TAGGTTTAAG T"CTGACGAGA ATAATATTCT ACAACGAGCA ATTCATTTAT "  
       "GGCTTGGGAA CAAAAAATCA ATCCA"AATTC AGACTGCTCT TATTATAAGA TGTTGCTCGT TAAGTAAATA   
  
047741 "TTTTAAACTG ACCCATTTAC TATCTATTAT T"TGATTGACT AATCCTTTAT ATTGCAATGG CTGAAGAGTC   
       AAAATTTGAC TGGGTAAATG ATAGATAATA AACTA"ACTGA TTAGGAAATA TAACGTTACC GACTTCTCAG "  
  
047811 AAATGTTTTG GCAATTCCTG AGGGGGGGAT GAATCAAGAT AATTTTGAAT "CAGTGTTCTC GATTTTTTTT "  
       "TTTACAAAAC CGTTAAGGAC TCCCCCCCTA CTTAG"TTCTA TTAAAACTTA GTCACAAGAG CTAAAAAAAA   
  
047881 "CATCTTTTGC TGTAATAATA TCTCGGGGTT TGCACCGATA ACTTGGTATA" TCCACTATAG AACCATTAAC   
       "GTAGAAAACG ACATTATTAT AGAGCCCCAA ACGTGGCTAT TGAACCATAT AGGTGATATC TTGGTAATTG"   
  
047951 TAAAATATGT CTATGATTAA CTAATTGTCG GGCTTGAGGA ATAGTC"GAAG CCATACCCAA TCGAAAAAGG "  
       ATTTTATACA GATACTAATT GATTAACAGC CCGAACTCCT TATCAGCTTC GGTATGGGTT AGCTTTTTCC   
  
048021 "ATGTTATCCA AACGCATTTC AAGTAATTGT AGTAAAACTT GACCCG"TTGA TCCTTTGGCT TTTCCGGCGA   
       TACAATAGGT "TTGCGTAAAG TTCATTAACA TCATTTTGAA CTGGGCAACT AGGAAACCGA AAAGGCCGCT "  
  
048091 TACGAACGTA TTTAAGTAAT TGTCGTTCTG TAAGACCATA AT"GAAAACGC AATTTTTGTT TTTCTTCTAA "  
       "ATGCTTGCAT" AAATTCATTA ACAGCAAGAC ATTCTGGTAT TACTTTTGCG TTAAAAACAA AAAGAAGATT   
  
048161 "ACGAATACGA TATTGAGATT TTTTACCGGA ACGCGATTGG TT"TTTAAGAT CGTTTCCGGT TTTGGGCCTT   
       TGCTTATGCT ATAACTCTAA AAAATGGCCT TGCGCTAACC AAAAATTCTA GCAAAGGCCA AAACCCGGAA   
  
048231 TTACTAGTTA GTCCCGGTAA AGCCCCCAGA CGGCGTATTT TTTT"GAAACG AGGCCTTCTG TAACGCGACA "  
       A"ATGATCAAT CAGGGCCATT TCGGGGGTCT GCCGCATAAA AAAACTTTGC TCCGGAAGAC ATTGCGCTGT "  
  
048301 "TAAAGACTCC TTATTTTATT TTATTTAAAT TTCATAAACT TAAA"TGAAAA CTTAAATGAT AAATATTATA   
       "A"TTTCTGAGG AATAAAATAA AATAAATTTA AAGTATTTGA ATTTACTTTT GAATTTACTA TTTATAATAT   
  
048371 AATGAAGCGA AATCCAGTAA AGTATTGTAC TACAAAACAA AGAATAATTA "GATAACTTGG ATCAATATCC "  
       TTACTTCGCT TTAGGTCATT TCATAACATG ATGTTTTGTT TCTTATTAAT CTATTGAACC TA"GTTATAGG "  
  
048441 "GGATCCTATA CTATTGTATA TGTAAATGAT ATAAAAAAGT AAGAGTTCCT" TTCTTGATTT GTTCTACAGA   
       "CCTAGGATAT GATAACATAT ACATTTACTA TATTTTTTCA TTCTCAAGGA AAGAACTAAA CA"AGATGTCT   
  
048511 AATAGAACCC CTTTCTTTAG TCATAAAGGT AAGT"AAGTTC CTACGACATG ATAGATTGGT GACCTTTGAA "  
       TTATCTTGGG GAAAGAAATC AGTATTTCCA TTCATTCAAG GATGCTGTAC TATCTAA"CCA CTGGAAACTT "  
  
048581 "AAAGGGTAAG AAGTAGTTTC GTCTATTTAT TTGA"TTTTTG ACATAATTGA TAGACTCTAG AGGATCCAAA   
       "TTTCCCATTC TTCATCAAAG CAGATAAATA AACTAAAAAC TGTATTAACT ATCTGAG"ATC TCCTAGGTTT   
  
048651 TAGAGAAAAG CCGGCTATCG GAATCGAACC GATGACCATC G"CATTACAAA TGCGATGCTC TAACCTCTGA "  
       ATCTCTTT"TC GGCCGATAGC CTTAGCTTGG CTACTGGTAG CGTAATGTTT ACGCTACGAG ATTGGAGACT "  
  
048721 "GCTAAGCGGG CTCACATAAC AGAAATGTTA CATGCATAGT A"ATTTACTAA ATTACTGGGA TCTTAGCTAT   
       "CGATTCGC"CC GAGTGTATTG TCTTTACAAT GTACGTATCA TTAAATGATT TAATGACCCT AGAAT"CGATA "  
  
048791 TCCTAATTAA TATGAATATA GAATCTAATT TCA"TTTCAAA TAAATATTCA ATATTGGAAT TGTTCTATTA "  
       "AGGATTAATT ATACTTATAT CTTAGATTAA AGTAAAGTTT ATTTATAAGT TATAACCTTA ACAAG"ATAAT   
  
048861 "TAGAACATAT TAATTAATAT AACGATTAAT AGA"ACGATTA ATAGAATATA GCGATAGAAA ATTTTGATCT   
       ATCTTGTATA ATTAATTATA TTGCTAATTA TCTTGCTAAT TATCTTATAT CGCTAT"CTTT TAAAACTAGA "  
  
048931 ATTTATCAAA TGGATGTGTA TCAATAATTA "ATGGGTTAAT CTCAATTAGA TAGTAAATTT TTGTTTTTGA "  
       "TAAATAGTTT ACCTACACAT AGTTATTAAT TACCCAATTA GAGTTAATCT ATCATT"TAAA AACAAAAACT   
  
049001 "ATTCAAATGT GAAATTCTTA TTATTATTAT" TTTTAAGATT TTTGGTTATT TTTCTTTTAG GTTTTTTAGT   
       TAAGTTTACA CTTTAAGAAT AATAATAATA AAAATTCTAA AAACCAATAA AAAGAAAATC CAAAAAATCA   
  
049071 TATTTACTCT TTTCTTTATT TTTTTTTACT AT"GCTATTTG AATGTCTGTC TATTTAAATG AAATCAAAAT "  
       ATAAA"TGAGA AAAGAAATAA AAAAAAATGA TACGATAAAC TTACAGACAG ATAAATTTAC TTTAGTTTTA "  
  
049141 "ATTACAATAT TTCATTACAT AATTTTGCTT TT"CTCTTTTT ATATCAATAT AAAAAAATAT ATTGAAATTT   
       "TAATG"TTATA AAGTAATGTA TTAAAACGAA AAGAGAAAAA TATAGTTATA TTTTTTTATA TAA"CTTTAAA "  
  
049211 CTTATTTTTT TTTATATTAT ATAATAATAT TATATAATGG GTTATA"GAAT AATTTATCGA ATAATAAAGT "  
       "GAATAAAAAA AAATATAATA TATTATTATA ATATATTACC CAATATCTTA TTAAATAGCT TAT"TATTTCA   
  
049281 "TATTTTTAGT TATGTTATAG AGGATCTCCC CTAAGGGTAA GAATAA"ATAA AGAAAAATGA AAAGGTGCAA   
       ATAAAAATCA ATACAATATC TCCTAGAGGG GATTCCCATT CTTATTTATT TCTTTTTACT TTTCCA"CGTT "  
  
049351 TCCAACCAAC TAAATGCAAT TGAGATGATA AT"GAAGCATT TCTATGTTTT CATTCTGAAA GGTGGAAGAT "  
       "AGGTTGGTTG ATTTACGTTA ACTCTACTAT TACTTCGTAA AGATACAAAA GTAAGACTTT CCACCT"TCTA   
  
049421 "AAGACGAAGA AAAGAATCGA CCGTTTAAGT AT"TCATTTTT TTACTAAAAA AAGAATAGAA AGAGGGGCAA   
       TTCTGCTTCT TTTCTTAGCT GGCAAATTCA TAAGTAAAAA AATGATTTTT TTCTTATCTT T"CTCCCCGTT "  
  
049491 ATTTATATGT CTATATATCT ATCTATATTG ATATATAT"TG AATTGCAGAT ACAGAAATGA TAGAATCATT "  
       "TAAATATACA GATATATAGA TAGATATAAC TATATATAAC TTAACGTCTA TGTCTTTACT A"TCTTAGTAA   
  
049561 "TTGGATCGGA ACAAATACGG GTCTCCGATA GAGAGAGA"TT AAAGAAGATA GACAAGAAAT CCAATCCAAT   
       AACCTAGCCT TGTTTATGCC CAGAGGCTAT CTCTCTCTAA TTTCTTCTAT CTGTTCTTTA GGTTAGGTTA   
  
049631 AGGAGGAAAC ACTTTTCAAT AGAGGAATCG GTATTCTAAT GACTT"CAGTG GTTCGTTCCA GTATAATACA "  
       "TCCTCCTTTG TGAAAAGTTA TCTCCTTAGC CATAAGATTA CTGAAGTCAC CAAGCAAGGT CATATTATGT"   
  
049701 "AATGAAAGAA ACGGGGAAGG ACATCATAGT GCGATACTAA TCTTA"AAGAG "GGGGATATGG CGAAATCGGT "  
       TTACTTTCTT TGCCCCTTCC TGTAGTATCA CGCTATGATT AGAATTTCTC CCCCTATACC GCTT"TAGCCA "  
  
049771 "AGACGCTACG GGCTT"AATTA GATTGAGCCT TGGTA"TGGAA ACCTACTAAG TGATAACTTT CAAATTCAGA "  
       "TCTGCGATGC CCGAATTAAT CTAACTCGGA ACCATACCTT TGGATGATTC ACTATTGAAA GTTT"AAGTCT   
  
049841 "GAAACCCTGG AATTAAAAAT GGGCAATCCT GAGCC"AAATC CTTTTTCCGA AAAAAAAAAA GGGGTAGGTG   
       CTTTGGGACC TTAATTTTTA CCCGTTAGGA CTCGGTTTAG GAAAAAGGCT TTTTTTTTTT "CCCCATCCAC "  
  
049911 CAGAGACTCA ATGGAAGCTG TTCTAACAAA T"GGAGTTGAC GACGTTGCGT TAGTAAAGGA ATGAATCCTT "  
       "GTCTCTGAGT TACCTTCGAC AAGATTGTTT ACCTCAACTG CTGCAACGCA ATCATTTCCT" TACTTAGGAA   
  
049981 "CCATCGAAAC TCCAGAAACG ATGAAAGATA A"ACTTATTAC GTACTGAAAT ACTATATTGA TTAATGACAA   
       GGTAGCTTTG AGGTCTTTGC TACTTTCTAT TTGAATAATG CATGACTTTA TGATA"TAACT AATTACTGTT "  
  
050051 CTCGAATCTC TATTTTTTAT ATTTATATAT TTTTTAGATT TATATATATT T"TTTTATTTA TATAGATATA "  
       "GAGCTTAGAG ATAAAAAATA TAAATATATA AAAAATCTAA ATATATATAA AAAAA"TAAAT ATATCTATAT   
  
050121 "TAAAATTATA TATAAAAATG AACGAATTGT TATGAATCGA TTCCAAGACC T"CCAAGTTGA AAAAAGAATC   
       ATTTTAATAT ATATTTTTAC TTGCTTAACA ATACTTAGCT AAGGTTCTGG AGGTTCAACT TTTTTC"TTAG "  
  
050191 GAATATTCAT TGATCAAATC ATTTACTCCA TCATACATAG TCTGATAGAT "CTTTTGAAGA ACTGATTAAT "  
       "CTTATAAGTA ACTAGTTTAG TAAATGAGGT AGTATGTATC AGACTATCTA GAAAACTTCT TGACTA"ATTA   
  
050261 "CAGATCGAAT AGAATAAAGA TAGAGTCCCA TTCTACATGT CAATACCGAC" AAAAATGAAA TTTATAGTAA   
       GTCTAGCTTA TCTTATTTCT ATCTCAGGGT AAGATGTACA GTTATGGCTG TTTTTACTTT AAATATCATT   
  
050331 GAGG"AAAATC CGTCGACTTT TAAAATCGTG AGGGTTCAAG TCCCTCTATC CCCA""AAACCC GAAAAAGGGC "  
       CTCC"TTTTAG GCAGCTGAAA ATTTTAGCAC TCCCAAGTTC AGGGAGATAG GGGTTTTGGG CTTTTTCCCG "  
  
050401 "CCGTTGGCCT CTTTAATTAT TTATCCTTTC ATTAGCAATT CACAATTTGT TATC"TTTCTC ATTCATTCGA   
       "GGCA"ACCGGA GAAATTAATA AATAGGAAAG TAATCGTTAA GTGTTAAACA ATAGAAA"GAG TAAGTAAGCT "  
  
050471 CTCTTTCACA AACGTATTTG AGCGGAAATT TGATTTCTTA TCACAAGGCT TGTGGTATAT AT"TCTATATG "  
       "GAGAAAGTGT TTGCATAAAC TCGCCTTTAA ACTAAAGAAT AGTGTTCCGA ACACCAT"ATA TAAGATATAC   
  
050541 "ATACACGTAC AAACGAACAT CCTTGCGCAA GTAATCTGTG AAATTTGAAT GATTAACAAT AC"TATCTACT   
       TATGTGCATG TTTGCTTGTA GGAACGCGTT CATTAGACAC TTTAAACTTA CTAATTGTTA TGATA"GATGA "  
  
050611 GTACTGAAAC TTCGAAAGTC TTATCCAAGC CCTGAAATTT CGTGTATCTT CAAAAAGAAG ACTT"TGGAAT "  
       "CATGACTTTG AAGCTTTCAG AATAGGTTCG GGACTTTAAA GCACATAGAA GTTTTTCTTC TGAAA"CCTTA   
  
050681 "ACCTTTTTTC TTATTTACAA TTGACATAGA CCAAAGTCAT CTATTAAAAT AAGGATAATG TGTC"GGAAAT   
       TGGAAAAAAG AATAAATGTT AACTGTATCT GGTTTCAGTA GATAATTTTA TTCCT"ATTAC ACAGCCTTTA "  
  
050751 G"GCCGGGATA GCTCAGTTGG TAGAGCAGAG GACTGAAAAT CCTCGTGTCA CCAGTTCAAA TCTGGTTCTT "  
       "CCGGCCCTAT CGAGTCAACC ATCTCGTCTC CTGACTTTTA GGAGCACAGT GGTCA"AGTTT AGACCAAGAA   
  
050821 "G"GCACATCAT GAATTTGTAT GAGTATCTAT TCTACGGATT CATTAATAAT AT"AGATCATG AAAAATATTG "  
       CCGTGTAGTA CTTAAACATA CTCATAGATA AGATGCCTAA GTAATTATTA TATCTAGTA"C TTTTTATAAC "  
  
050891 "ATACATCATG ATGTTTGGAA ATCCACCCTT TAACTAAACT ATATATATTT TT"TACTCTAT TTATAGATGT   
       "TATGTAGTAC TACAAACCTT TAGGTGGGAA ATTGATTTGA TATATATAAA AAATGAGAT"A AATATCTACA   
  
050961 CGAAAATAGA CAAGTAAAGA TAAAGAGGAT ATTTATAGAA ATATGTAAAG GAT"GTATATA GATATGTATA "  
       GCTTTTATCT GTTCATTTCT ATTTCTCCTA TAAATATCTT TATACATTTC CTACATATAT CTATACATAT   
  
051031 "TGTAAAAGAA AAGAATTTTA TTTTGTTTCC TCTTCTTTTT TATTTGTTCA TAC"TCTGTCT CCTGGCGTTC   
       ACATT"TTCTT TTCTTAAAAT AAAACAAAGG AGAAGAAAAA ATAAACAAGT ATGAGACAGA GGACCGCAAG "  
  
051101 AATTAATATT TTATACATAT TTGAAAGTTT CAATTAGTTG CTTATAAG"AC CCAAAAGTCT AGTCTAGGGG "  
       "TTAAT"TATAA AATATGTATA AACTTTCAAA GTTAATCAAC GAATATTCTG GGTTTTCAGA "TCAGATCCCC "  
  
051171 "AGTTGAAAGG CGGGAATGGC CAAGATTCAT CTCAGATACA GTACAAAT"AG AATCCGATTC TCTTTTCATA   
       "TCAACTTTCC GCCCTTACCG GTTCTAAGTA GAGTCTATGT CATGTTTATC TTAGGCTAAG" AGAAAAGTAT   
  
051241 TTATATTTCT TCATTTCACC CTATGTTTTT AAAGCACAT"C TATAAGTCAT ATGCGTGATA CATAGTTCAT "  
       AATATAAAGA AGTAAAGTGG GATACAAAAA TTTCGTGTAG ATATTCAGTA TACGCACTAT GTATCAAGTA   
  
051311 "AATGCAGAAC TCGTTTAGTT CATCCTATTA GCTTGTGGC"T CATCCAAAAT AAGTATCTTA CAAATTGGAT   
       TTA"CGTCTTG AGCAAATCAA GTAGGATAAT CGAACACCGA GTAGGTTTTA TTCATAGAAT GTTTAACCTA "  
  
051381 AATCTAACCA AATCTCTAAT TGTATTTTTT TTTTTATTTC AAATCAATAA TAATAA"GATG AATGAGACTT "  
       "TTA"GATTGGT TTAGAGATTA ACATAAAAAA AAAAATAAAG TTTAGTTATT ATTATTCTAC TTACTCTGAA   
  
051451 "CAATTACTAT GCTATATCTA TAAGAGAACG TAAAAATGTA TTTGAATATC TTGAGT"TGTC GAAGTGAAGA   
       GTTAA"TGATA CGATATAGAT ATTCTCTTGC ATTTTTACAT AAACTTATAG AACTCAACAG CTTCACTTCT "  
  
051521 TTAGTTTCGT ATTATTTAAT GAGCATCTTG TATTTCATAA AAATT"GGGGG CAATATAATC CTTACGTAAG "  
       "AATCA"AAGCA TAATAAATTA CTCGTAGAAC ATAAAGTATT TTTAA"CCCCC GTTATATTAG GAATGCATTC "  
  
051591 "GGCCATCCTA CCCAACTTTC AGGCATTAAG ATACGCTTTA GACGT"GGATG ATTATCATAA TAAATTCCCA   
       "CCGGTAGGAT GGGTTGAAAG TCCGTAATTC TATGCGAAAT CTGCA"CCTAC TAATAGTATT ATTTAAGGGT   
  
051661 ACATATCATA AGATTCCCTT TCTGGAAAAT CCGCACTTTT CCAAACCCAG AAAA"CAGACG GAATTCTAGG "  
       TGTATAGTAT TCTAAGGGAA AGACCTTTTA G"GCGTGAAAA GGTTTGGGTC TTTTGTCTGC CTTAAGATCC "  
  
051731 "ATTTATCCTT GCGGCAAATA CTTTTATGCA TACCTCTTCC GGTTGATCTA TACC"ATACTC GATTCTCGTA   
       "TAAATAGGAA CGCCGTTTAT GAAAATACGT A"TGGAGAAGG CCAACTAGAT ATGGTATGAG CTAAGAGCAT   
  
051801 AGATGATACA CACTAGCTAA CAGTCCGCCT GGTGCTACAT CATAAGCA"CA TTGGGAACGT AGATAATTGT "  
       TCTACTATGT GT"GATCGATT GTCAGGCGGA CCACGATGTA GTATTCGTGT AACCCTTGCA TCTATTAACA "  
  
051871 "AACCATATAC ATATAAAATG ACTGCAATGG AATGCCATTC CTCGGGCT"TT ATTTGTAAAG TCTCTATTCC   
       "TTGGTATATG TA"TATTTTAC TGACGTTACC "TTACGGTAAG GAGCCCGAAA TAAACATTTC AGAGATAAGG "  
  
051941 TTGGTAATCG AAACCCAAAG ATCTATGAAC CAGTCC"ATGT TTGACTAGCC AAGAAGACAA ATTACCCTGC "  
       "AACCATTAGC TTTGGGTTTC TAGATACTTG" GTCAGGTACA AACTGATCGG TTCTTCTGTT TAATGGGACG   
  
052011 "ATCTTTTTTA TCCCCCCCCC CCATTTTTTA TTTTTA"TAAG TATTTCCCAT TTACAATGAA AAATTTATGA   
       TAGAAAAAAT AGGGGGGGGG GGTAAAAAAT AAAAATATTC ATAAAGGGTA "AATGTTACTT TTTAAATACT "  
  
052081 AGATTCACTC GTTCTTTGCT TTGTTATTCT GTACAAAAAA A"GTATACGGC CTAATTTACT AATTCGTAGG "  
       "TCTAAGTGAG CAAGAAACGA AACAATAAGA CATGTTTTTT TCATATGCCG" GATTAAATGA TTAAGCATCC   
  
052151 "AAGAGACTGC ACTTTTGTAT TTGAAAAAAG TTTCAGGAGG G"ATGATCTCT GAAGTAGATG ATGGTTGATA   
       TTCTCTGACG TGAAAACATA AACT"TTTTTC AAAGTCCTCC CTACTAGAGA CTTCATCTAC TACCAACTAT "  
  
052221 GAGTAATTCT TGATCGTAAT TTCCAGTATG AGTACT"TGGG CCAACATGAA ATTTGTGATT GGTAGTAAAA "  
       "CTCATTAAGA ACTAGCATTA AAGG"TCATAC TCATGAACCC GGTTGTACTT TAAACACTAA CCATCATTTT   
  
052291 "CACCGATTTT CCTCTTGAGA CTTAATTCGA TCTTCA"TAGA TTTCTCGAGA TATTTTCTTA CGAAGTTTTG   
       GTGGCTAAAA GGAGAACTCT GAATTAAGCT AGAAGTATCT AAAGAGCTCT ATAAAAGAAT GCTTCAAAAC   
  
052361 TTATAGCATC TATAACAGCC TCGGGTTTAG GTGGAC"AACC CGGCAAATAC ACATCGACAG GAATTAACTT "  
       AATATCGTAG ATATTGT"CGG AGCCCAAATC CACCTGTTGG GCCGTTTATG TGTAGCTGTC CTTAATTGAA "  
  
052431 "ATCGACTCCT CGAACAGTAC TATAAGAATC GGTACT"GAAC ATCCCTCCTG TAATTGTACA GGCTCCCATA   
       "TAGCTGAGGA GCTTGTC"ATG ATATTCTTAG CCATGACTTG TAGGGAGGAC ATTAACATGT CCGAGGGTAT   
  
052501 GCAATAACAT ATTTTGGTTC AGGCATTTGC TCATATAATC TTACTAAAGA AGGAG"CCATT TTCATTGTTA "  
       CGTTATTGTA TAAAACCAAG TCCGTAAACG AGTATATTAG AATGATTTCT TCCTCGGTAA "AAGTAACAAT "  
  
052571 "CCGTACCCGC TGTTAAAATT AAGTCTGCCT GTCTAGGACT AGATCTTGGT ACCAG"TCCAT AACGATCAAA   
       "GGCATGGGCG ACAATTTTAA TTCAGACGGA CAGATCCTGA TCTAGAACCA TGGTCAGGTA" TTGCTAGTTT   
  
052641 GTCGAATCGT GAACCTATTA ATGAAGCGAA TTCAATGAAG CAACAAC"TGG TACCATAGAG AAGCGGCCAT "  
       CAGCTTAGCA CTTGGATAAT TACTTCGCTT AAGTTACTTC GTTGTTG"ACC ATGGTATCTC TTCGCCGGTA "  
  
052711 "AAACTGGAAA GTCTTGACCA ATTTGAAAGA TCATTTGATG TAGTTGA"AAT AACTGAAGTT TGAGTTGTTC   
       "TTTGACCTTT CAGAACTGGT TAAACTTTCT AGTAAACTAC ATCAACT"TTA TTGACTTCAA ACTCAACAAG   
  
052781 GATCAAGTAA GGGAAACTCG ATGGAATTCA TAACTGTCTC AATGTTTTTT TTAAT"TGTCT GAATATTCAA "  
       CTAGTTCATT CCCTTTGAGC TACCTTAAGT ATTGACAGAG TTACAAAAAA AATTAACAGA CTTATAAGTT   
  
052851 "AAACTAAGAC CATTCCAATG CTCCTTTTCG CCATGCATAA ACTAAACCGA CAATT"AGGAT AAGCACGAAA   
       TTTGATTCTG GTAAG"GTTAC GAGGAAAAGC GGTACGTATT TGATTTGGCT GTTAATCCTA TTCGTGCTTT "  
  
052921 ATTAAAGCTT CTATAAAGAC GGATACCCCT AATACA"TCAA AACTCATTGC CCACGGATAA AGAAAAACTG "  
       "TAATTTCGAA GATAT"TTCTG CCTATGGGGA TTATGT"AGTT TTGAGTAACG GGTGCCTATT TCTTTTTGAC "  
  
052991 "TTTCAACATC AAAAACAACA AAAACTAGAG CAAACA"TATA ATAACGGATT CTAAATTGTA CCCAAGCATC   
       "AAAGTTGTAG TTTTTGTTGT TTTTGATCTC GTTTGT"ATAT TATTGCCTAA GATTTAACAT GGGTT"CGTAG "  
  
053061 GCCCATTGGT TCTATTCCCG ATTCATAACT CGAAAGTTT"C TCTGGCTCTT TGCTAAGAGG GGCTAAAACT "  
       "CGGGTAACCA AGATAAGGGC TAAGTATTGA GCTTTCAAAG AGACCGAGAA ACGATTCTCC CCGAT"TTTGA   
  
053131 "CCAGAAATTA GAAATGCCAA AATAGGAATA ACACTTGAT"A TTATTAGAAA TACCCAGAAA ATATCATATT   
       GGTCTTTAAT "CTTTACGGTT TTATCCTTAT TGTGAACTAT AATAATCTTT ATGGGTCTTT TATAGTATAA "  
  
053201 CGTAAAGCAG AAACATAAAC GTACTCCTAT CAATGTGGAA AA"TATACCGG ATTAGTCGAT CGGTACATGT "  
       "GCATTTCGTC" TTTGTATTTG CA"TGAGGATA GTTACACCTT TTATATGGCC TAATCAGCTA GCCATGTACA "  
  
053271 "CTCGTTTGAA GATTCATCCA CTAGAATCCT ATTTATATAA TA"TCTATTAT ATCCATAATA AATATATATT   
       "GAGCAAACTT CTAAGTAGGT GA"TCTTAGGA TAAATATATT ATAGATAATA TAGGTATTAT TTATATATAA   
  
053341 AAATATTATA TATTTATATA GAGAGGATAC ATATAG"TAGG AATTTCCATT TAGGAACTTT TAGTGATTGA "  
       TTTATAATAT ATAAATATAT CTCTCCTATG TATATC"ATCC TTAAAGGTAA ATCCTTGAAA ATCACTAACT "  
  
053411 "AGAAGGAAAG AGAAGACGAC TACTTTAGTT TTGTGC"TTTA CTAGATAAGG TATACCAAGA GAAAAGCCTA   
       "TCTTCCTTTC TCTTCTGCTG ATGAAATCAA AACACG"AAAT GATCTATTCC ATATGGTTCT CTTTTCGGAT   
  
053481 TTTGAGAATG TTTACAATGA AAGTTACCAA AGATCTTCGT TTCTCAAACT CTA"GCTTGTC CACAAATCAA "  
       AAACTCTTAC AAATGTTACT TTCAATGGTT TCTA"GAAGCA AAGAGTTTGA GATCGAACAG GTGTTTAGTT "  
  
053551 "ATACAGTAAG TCGTTCCTAG ATCCATGTGA TTTACTAGTG CATTCGATTT GCA"TTGGGTT ATGGTGGAGT   
       "TATGTCATTC AGCAAGGATC TAGGTACACT AAAT"GATCAC GTAAGCTAAA CGTAACCCAA TACCACCTCA   
  
053621 TTTTAACCGG TTTCATGTCA TGATTTTGAC TCCCAAAATT T"ACTAGAATT GGGTAGGTAT CCCAAAGAAA "  
       AAAATTGGCC AAAGTACAGT ACTAAAACTG AGG"GTTTTAA ATGATCTTAA CCCATCCATA GGGTTTCTTT "  
  
053691 "AGAAGTATTA CTAATTTCTT GATTTTGTAT GGGAAAATAT G"AAAATTTTC ATATGTAATT AATCTACAAA   
       "TCTTCATAAT GATTAAAGAA CTAAAACATA CCC"TTTTATA CTTTTAAAAG TATACATTAA TTAGATGTTT   
  
053761 GATAAGACAT TAATGATGAA CCAAATCAAG TGGCTAGTTA CAAACAATAC AAACA"ATAAT GAGGAAAGGA "  
       CTATTCTGTA ATTACTACTT GGTTTAGTT"C ACCGATCAAT GTTTGTTATG TTTGTTATTA CTCCTTTCCT "  
  
053831 "AAACGATTCT ATTTTTGAAT TATTAATAAT ATAGGGCTAT ACGGACTCGA ACCGT"AGACC TTCTCGGTAA   
       "TTTGCTAAGA TAAAAACTTA ATAATTATT"A T"ATCCCGATA TGCCTGAGCT TGGCATCTGG AAGAGC"CATT   
  
053901 AACAGATCAA ACTTATTATT ATCAAAATAA TTTGAACTGT T"TCAAAGACC CAACATGCAT TTTTTTGCAT "  
       TTGTCTAGTT TGAATAATAA TAGTTTTATT AAACTTGACA AAGTTTCTGG GTTGTACGTA AAAAAACGTA   
  
053971 "TGGGCTCTTT CATTAACTGT TATAAATAAG TTAGTCTACC A"TATTTTTTC TTGGCAGAAA GTAAAGGGAG   
       ACCCGAGAAA GTAATT"GACA ATATTTATTC AATCAGATGG TATAAAAAAG AACCGTCTTT CATTTCCCTC "  
  
054041 ATGGTTCCAT GTGCTCTGAT TCATTATTTT GATTCGAATC TCGGAGCACT AC"CAAAGTGT TTCAAAGAAG "  
       "TACCAAGGTA CACGAG"ACTA AGTAATAAAA CTAAGCTTAG AGCCTCGTGA TGGTTTCACA AAGTTTCTTC   
  
054111 "GGTTATCCTG ACGTAGGTAT GCTTCTGACC TAGATCAACT TAAGTTAAAT GG"AATCTCTA TCGTCCTGCT   
       CCAATA"GGAC TGCATCCATA CGAAGACTGG ATCTAGTTGA ATTCAATTTA CCTTAGAGAT AGCAGGACGA "  
  
054181 TCTGCTTAAA GAATAAAATA TGAAACTTCA TACACCTTA"A AGTTGTTCAT AAGATAGGAC GAAAGATCAT "  
       "AGACGA"ATTT CTTATTTTAT ACTTTGAAGT ATGTGGAATT TCAACAAGTA TTCTATCCTG CTTTCTAGTA   
  
054251 "TTTTTTTTTT GAGGTCCTGA TACTCATTAT GCCTAGCAT"T GAATAGACTG GGTATTCACC TTATCAATAT   
       AAAAAAAAAA CTCCA"GGACT ATGAGTAATA CGGATCGTAA CTTATCTGAC CCATAAGTGG AATAGTTATA "  
  
054321 CTCAAATCAA TGATGGATTT TTTTAGTGCC TAAATG"GGGC ACTCGATTCG GACCGAACTT TTTGTCAGGC "  
       "GAGTTTAGTT ACTAC"CTAAA AAAATCACGG ATTTACCCCG TGAGCTAAGC CTGGCTTGAA AAACAGT"CCG "  
  
054391 "TACTGTTCTC TTGTTTTGTT CCCTAAAAGT AATAGA"GTAA GACATCGATT TCTCAATAAG ATCAACTTTT   
       "ATGACAAGAG AACAAAACAA GGGATTTTCA TTATCTCATT CTGTAGCTAA AGAGTTATTC TAGTTGA"AAA   
  
054461 TTGATTACAT GATGTACTTC TCTGAAAAAC ATTGGCGCGC GT"GTAAACCG AGGTGCTTCT ACCCAACTGA "  
       AACTAATGTA CTACATGAAG AGACTTTTTG TAACCGCGCG "CACATTTGGC TCCACGAAGA TGGGTTGACT "  
  
054531 "GCTATAGCCC TTGTGCCTTG TGATACATAT CTTATCATGT AG"ATAATTTC TTGTCAAGAT AAATATTCTA   
       "CGATATCGGG A"ACACGGAAC ACTATGTATA GAATAGTACA TCTATTAAAG AACAGTTCTA TTTATAAGAT   
  
054601 TGATCTATTT TGTTGATTTG GTTGATTGGT ATTGCTTATC AATAATATTC GATTTATAAT CTATCGGTGT   
       ACTAGATAAA ACAACTAAAC CAACTAACCA TAACGAATAG TTATTATAAG CTAAATATTA GATAGCCACA   
  
054671 GATAGGGCTC ATTTGTTTTT CTTTGTGATG ATAAATGACC "TACTTAACTC AGTGGTTAGA GTATCGCTTT "  
       CTATCCCGAG TA"AACAAAAA GAAACACTAC TATTTACTGG ATGAATTGAG TCACCAATCT CATAGCGAAA "  
  
054741 "CATACGGCGG GAGTCATTGG TTCAAATCCA ATAGTAGGTA" GAACTTATTA GATACCACAG AAGCAATGGT   
       "GTATGCCGCC CT"CAGTAACC AAGTTTAGGT TATCATCCAT CTTGAATAAT CTATGGTGTC TTCGTTACCA   
  
054811 ATCTAATAAG TTTTTTTACT CAACTTT"GTG CTAGTGATTT TCTATCTTTT CCATCCGACT CTATATTTAT "  
       "TAGATTATTC AAAAAAATGA GTTGAAACAC GATCACTAAA AGATAGAAAA GGTAGGCTGA GATATAAATA"   
  
054881 "TTCATTTTTG AAATGTAATT CTTTTTT"CCA TGAAAATTGA ATTTCACTTG ATTATTTAGA CGCATTAACT   
       AAGTAAAAAC TTTACATTAA GAAAAAAGGT ACTTTTAACT TAAAGTGAAC TAATAAATCT GCGTAATTGA   
  
054951 AGGTACGCCA TTGATAGCCT CTA"CTCGTGT CCTAGCTCGT CTGAGAGCTA AATTTGCCTC GATTGTTTGT "  
       TCCATGCGGT AACTATCGGA GATGA"GCACA GGATCGAGCA GACTCTCGAT TTAAACGGAG CTAACAAACA "  
  
055021 "CTCTTGCCCT GAGCTTTCCT CAA"GTTTGCT TCTGCTATTT CAAGAGTTTG CTGAGCTTCT TGTGGATCAA   
       "GAGAACGGGA CTCGAAAGGA GTTCA"AACGA AGACGATAA"A GTTCTCAAAC GACTCGAAGA ACACCTAGTT "  
  
055091 TGTCACTAGC CTTCTCCGCA TC"ATTTACTA AAATAGTGAC CTCATTATTG CCTATTCTAG CAAAACCACC "  
       "ACAGTGATCG GAAGAGGCGT AGTAAATGAT TTTATCACT"G GAGTAATAAC GGATAAGATC GTTTTGGTGG   
  
055161 "CATCAGAGCC ATCGTTAACC AT"CCATCATT AGGAGTAAGG CGTATTTTCA AAATACCTAT ATCTACAGCT   
       "GTAGTCTCGG TAGCAATTGG TAGGTAGTAA TCCTCATTCC GCATAAAAGT TTTATGGATA TAGATGTCGA"   
  
055231 GTGGCAATAG GT"GCGTGATC TGGTAATACA CCAATTTGGC CACTATTCGT AACTAAAATG ATTTCTTTCA "  
       CACCGTTATC CACGCACTAG ACCATTATGT GGTTA"AACCG GTGATAAGCA TTGATTTTAC TAAAGAAAGT "  
  
055301 "CTTCTGAATC CC"AAATAATT CGATTCGGAG TCAGGACACT AAGATTTAAG GTCATTTCTT CAAATTGCTC   
       "GAAGACTTAG GGTTTATTAA GCTAAGCCTC AGTCC"TGTGA TTCTAAATTC CAGTAAAGAA GTTTAACGAG   
  
055371 TCCATTTCTA AGTTCGTAGC CTTCGCAGTA G"CTTCATCGA TGTTACCTAC CAAATAAAAG GCTTGTTCGG "  
       AGGTAAAGAT TCAAGCATCG GAAGCGTCAT CGAAGTAGCT ACAATGGATG GTTTATTTTC CGAACAAGCC   
  
055441 "GAAGACCATC TAATTCTCCG GAAAGGATTA A"TTTAAACCC TCTAATTGTT TCTGCTAGGC CAACGTATTT   
       CTTCTGGTAG ATTAAGAGGC CTTTCCTAAT TAAATTTGGG AGATTAACAA AGACGATCCG GTTGCATAAA   
  
055511 CCCCGGGGAG CCGGTAAATA CTTCTGCTA"C GAAAAAAGGT TGTGATAAGA AACGTTCAAT TTTTCGTGCT "  
       GGGGCCCCTC GGCCATTTAT GAAGACGATG CTTTTTTCCA ACACTATTCT TTGCAAGTTA AAAA"GCACGA "  
  
055581 "CGTGCTACGG TTAAGCGATC CTCTTCGGA"T AATTCGTCCA ACCCAAGGAT AGCTATAATG TCTTGCAGTT   
       "GCACGATGCC AATTCGCTAG GAGAAGCCTA TTAAGCAGGT TGGGTTCCTA TCGATATTAC AGAA"CGTCAA   
  
055651 CTTTGTAACG TTGTAAGGTT TGTTTAACTC "TTTGCGCAGT TTCATAATGT TCTTCACCAA CGATTCTCGG "  
       GAAACATTGC AACATTCCAA ACAAATTGAG AAACGCGTCA AAGTATTACA AGAAGTGGTT GCTAAGAGCC   
  
055721 "TTGTAGCATA GTTGAGGTTG AATCTAAAGG" ATCTACTGCT GGATAGATAC CTTTGGCAGC TAATCCTCTT   
       AACATCGTAT CAACTCCAAC TTAGATTTCC TAGATGACGA CCTATCTATG GAAACCGTCG ATTAGGAGAA   
  
055791 GATAGTACGG TAGTAGCATC "CAAATGTGCA AATGTGGTGG CAGGAGCAGG ATCGGTCAAA TCGTCCGCAG "  
       CTATCATGCC ATCATCGTAG GTTTACACGT TTACACCACC GTCCTCGTCC TAGCCAGTTT AGCAGGCGTC   
  
055861 "GTACATAAAC AGCTTGAATA" GAAGTTATGG ACCCTTCCTT GGTAGAAGTA ATTCTTTCTT GTAAGGAACC   
       CATGTATTTG TCGAACTTAT CTTCAATACC TGGGAAGGAA CCATCTTCAT TAAGAAAGAA CATTCCTTGG   
  
055931 CATTTCGGTA CTAAGAG"TGG GTTGATAACC CACAGCCGAA GGCATTCTAC CCAGTAACGC GGATACTTCG "  
       GTAAAGCCAT GATTCTC"ACC CAACTATTGG GTGTCGGCTT CCGTAAGATG GGTCATTGCG CCTATGAAGC "  
  
056001 "GATCCCGCTT GAACGAA"ACG AAAGATATTG TCGATAAATA GAAGCACGTC TTGTTCATTA ACATCTCGGA   
       "CTAGGGCGAA CTTGCTT"TGC TTTCTATAAC AGCTATTTAT CTTCGTGCAG AACAAGTAAT TGTAGAGCCT   
  
056071 AATATTCCGC CATAGTTA"GG GCAGTTAAAC CAACTCTCAT ACGAGCTCCC GGCGGTTCAT TCATCTGACC "  
       TTATAAGGCG GTATCAATCC CGTCAATTTG GTTGAGAGTA TGCTCGAGGG CCGCCAAGTA AGTAGACTGG   
  
056141 "GTAGACTAGA GCCACTTT"TG ATTCTGCAAA ATTTTCTTCA TTAATTACTC CGGATTCTTT CATTTCCATG   
       CATCTGATCT CGGTGAAAAC TAAGACGTTT TAAAAGAAGT AATTAATGAG GCCTAAGAAA GTAAAGGTAC   
  
056211 TAAAGATCAT TTCCTTCACG AGTACGTT"CA CCTACTCCTC CAAATACAGA TACACCTCCA TGAGCTTTGG "  
       ATTTCTAGTA AAGGAAGTGC TCATGCAAGT GGATGAGGAG GTTTATGTCT ATGTGGAGGT ACTCGAAACC   
  
056281 "CAATGTTGTT GATCAATTCC ATAATGAG"TA CTGTTTTACC CACTCCAGCC CCTCCGAATA GTCCTATTTT   
       GTTACAACAA CTAGTTAAGG TATTACTCAT GACAAAATGG GTGAG"GTCGG GGAGGCTTAT CAGGATAAAA "  
  
056351 TCCTCCACGT C"GATAAGGGG CTAACAGATC CACTACTTTA ATTCCTGTTT CAAAAATAGA TAATTTTGTA "  
       "AGGAGGTGCA GCTATTCCCC GATTGTCTAG GTGATGAAAT TAAGG"ACAAA GTTTTTATCT ATTAAAACAT   
  
056421 "TCTAACTGGA T"AAAAGCAGG GGCGGATCTA TGAATAGGAG ATGTTGTGCG AGTATCTACA GGACCCAAAT   
       AGATTGACCT ATTTTCGTCC CCGCCTAGAT ACTTATCCTC TACAACACGC TCATAGATGT CCTGGGTTTA   
  
056491 TATCAATAGG CTCTCCAAGC A"CGTTGAAAA TTCGGCCGAG AGTCGCTCCG CCGACTGGAA CACTTAGAGG "  
       ATAGTTATCC GAGAGGTTCG TGCAACTTTT AAGCCGGCTC TCAGCGAGGC GGCTGACCTT GTGAATCTCC   
  
056561 "AGCTCTCGTG TCAACCACTT C"CATTCCTCT CCTTAGACCA TCTGTAGCAC TCATAGCTAC AGCTCTAACT   
       TCGAGAGCAC AGTTGGTGAA GGTAAGGAGA GGAATCTGGT AGACATCGTG AGTATCGATG TCGAGATTGA   
  
056631 CGATTATTTC CTAATAATTG CTGTACTTCA "CAAGTCACAT TAATTTCTTG ACCGGAAATA TCTTGACCTT "  
       GCTAATAAAG GATTATTAAC GACATGAAGT GTTCAGTGTA ATTAAAGAAC TGGCCTTTAT AGAACTGGAA   
  
056701 "TAACTATCAA AGCGTTGTAA ATATTAGGCA" TTTTGCCCGG GGGAAAAGCT ACATCTAGTA CCGGACCAAT   
       ATTGATAGTT TCGCAACATT TATAATCCGT AAA"ACGGGCC CCCTTTTCGA TGTAGATCAT GGCCTGGTTA "  
  
056771 TATTTGAGCG A"TACGTCCCA AGTTGTTTTT TTCAAGCGTG GAAACTTCAG GACTAGAAGT CGTAGGATTT "  
       "ATAAACTCGC TATGCAGGGT TCAACAAAAA AAG"TTCGCAC CTTTGAAGTC CTGATCTTCA GCATCCTAAA   
  
056841 "ATTCTCATAA T"AAAATGAAC TATGTCGAAA TTTTTTTGTT TCGAAAATTA TTGAAATCAA AAATAAATGT   
       TAAGAGTATT ATTTTACTTG ATACAGCTTT A"AAAAAACAA AGCTTTTAAT AACTTTAGTT TTTATTTACA "  
  
056911 TCGATGGAAA AGCA"AGTTGA TCGGTTAATT CAATAAAAAA TGCAATGGGA GTTCGCACTG GATTTCGTTG "  
       "AGCTACCTTT TCGTTCAACT AGCCAATTAA G"TTATTTTTT ACGTTACCCT CAAGCGTGAC CTAAAGCAAC   
  
056981 "GTACCGTCCA ATGG"AATCAA ATTCCATTGT TGACTTATGC CATTTGAATG AGTCAAATTG CAAGTTCAAC   
       CATGGCAGGT TACCTTAGTT TAAGGTAAC"A ACTGAATACG GTAAACTTAC TCAGTTTAAC GTTCAAGTTG "  
  
057051 CAACCCATTT TCAAAATATC ACGTGGATGA A"AAATCTTTC GAAAGTCTTT CATTTGCCTA TCATTATATA "  
       "GTTGGGTAAA AGTTTTATAG TGCACCTAC"T TTTTAGAAAG CTTTCAGAAA GTAAACGGAT AGTAATATAT   
  
057121 "CATTACTTAT CTATGGAATT CGAACCCGAA C"TCTATTTAT GAGTCATTTT TTCATTTCAT TGGTCCTTAT   
       GTAATGAATA GATACCTTAA GCTTGGGCT"T GAGATAAATA CTCAGTAAAA AAGTAAAGTA ACCAGGAATA "  
  
057191 TTCTTATTTC AGCATATCGA TTTA"GGTCTA ATCTATTAGT TTTTTCTTTT TTCTACTTAT ACCTTTTGTT "  
       "AAGAATAAAG TCGTATAGCT AAATCCAGA"T TAGATAATCA AAAAAGAAAA AAGATGAATA TGGAAAACAA   
  
057261 "TTTTTTTTCA TAGGAATTCT GCAT"ATTTTC ACATCTAGGA TTTACATATA CAACATATAT CACTGTCAAG   
       AAAAAAAAGT ATCCTTAAGA CGTATAAAAG TGTAGATCCT AAA"TGTATAT GTTGTATATA GTGACAGTTC "  
  
057331 AGTTAATTTT TT"TTATTTCA ATATTTTGAG GCAAAAGATT AGATTACAAA CTTGAAAAAA GCAGTTGGGT "  
       "TCAATTAAAA AAAATAAAGT TATAAAACTC CGTTTTCTAA TCT"AATGTTT GAACTTTTTT CGTCAACCCA   
  
057401 "TGCGCCATAC AT"ATGAAAGA GTATACAATA ATGCTGTATT TGGTGAATAA AATACCATGG TCTAATAACG   
       ACGCGGTATG TATACTTTCT CATATGTTAT T"ACGACATAA ACCACTTATT TTATGGTACC AGATTATTGC "  
  
057471 AACCATTCTG ATTAGTTGAT AATATTAGTT "GAGAATTTTG TGAAAGGATT CCTATAAAAG TTTTCATTAA "  
       "TTGGTAAGAC TAATCAACTA TTATAATCAA C"TCTTAAAAC ACTTTCCTAA GGATATTTTC AAAAGTAATT   
  
057541 "CTCCTAATTC ATGTCGAGTA GACCTTGTTG" TTGCGAGAAT TCTTAATTCA TGAGTTGTAG GGAGGGACTT   
       GAGGATTAAG TACAGCTCAT CTGGAACAAC AACGCTCTTA AGAA"TTAAGT ACTCAACATC CCTCCCTGAA "  
  
057611 ATGTCACCAC AAACAGAGAC TAAAGCAAGT GTTGGATTCA AAGCTGGTGT TAAAGATTAT AAATTGACTT   
       "TACAGTGGTG TTTGTCTCTG ATTTCGTTCA CAACCTAAGT TTCG"ACCACA ATTTCTAATA TTTAACTGAA   
  
057681 ATTATACTCC TGAATATGAA ACCAAAGATA C"TGATATCTT GGCAGCATTC CGAGTAACTC CTCAACCGGG "  
       TAATATGAGG ACTTATACTT TGGTTTCTAT G"ACTATAGAA CCGTCGTAAG GCTCATTGAG GAGTTGGCCC "  
  
057751 "AGTTCCACCT GAGGAAGCAG GGGCCGCTGT A"GCTGCTGAA TCTTCTACTG GTACATGGAC AACTGTGTGG   
       "TCAAGGTGGA CTCCTTCGTC CCCGGCGACA T"CGACGACTT AGAAGATGAC CATGTACCTG TTGACACACC   
  
057821 ACCGATGGGC TTACCAGTCT TGATCGTTAC AAAGGACGAT GCTATGGAAT CGAGCCTGTT GCTGGAGAAG   
       TGGCTACCCG AATGGTCAGA ACTAGCAATG TTTCCTGCTA "CGATACCTTA GCTCGGACAA CGACCTCTTC "  
  
057891 AAAATCAATA TATTGCTTAT GTAGCTTATC CTCTAGACCT TTTTGAAGAA GGTTCTGTTA CTAACATGTT   
       "TTTTAGTTAT ATAACGAATA CATCGAATAG GAGATCTGGA" AAAACTTCTT CCAAGACAAT GATTGTACAA   
  
057961 TACTTCCATT G"TGGGTAATG TATTTGGATT CAAGGCTCTA CGTGCTCTAC GTCTGGAGGA TTTGCGAATC "  
       ATGAAGGTAA CACCCATTAC ATAAACC"TAA GTTCCGAGAT GCACGAGATG CAGACCTCCT AAACGCTTAG "  
  
058031 "CCTACTGCTT A"TATTAAAAC TTTCCAAGGC CCGCCTCATG GTATCCAGGT TGAAAGAGAT AAATTGAACA   
       "GGATGACGAA TATAATTTTG AAAGGTT"CCG GGCGGAGTAC CATAGGTCCA ACTTTCTCTA TTTAACTTGT   
  
058101 AGTATGGTCG CCCTCTATTG GGATGTACTA TTAAACCAAA ATTGGGATTA TCCGCTAAGA ATTATGGTAG   
       TCATACCAGC GGGAGATAAC CCTACATGAT AA"TTTGGTTT TAACCCTAAT AGGCGATTCT TAATACCATC "  
  
058171 AGCAGTTTAT GAATGTCTAC GCGGTGGACT TGATTTTACC AAAGATGATG AAAACGTGAA TTCCCAACCA   
       "TCGTCAAATA CTTACAGATG CGCCACCTGA AC"TAAAATGG TTTCTACTAC TTTTGCACTT AAGGGTTGGT   
  
058241 TTTATGCGTT GGAGAGACCG TTTCCTATTT TGTGCGGAAG CTATTTTTAA ATCACAGGCT GAAACAGGTG   
       AAATACGCAA CCTCTCTGGC AAAGGATAAA ACACGCCTTC G"ATAAAAATT TAGTGTCCGA CTTTGTCCAC "  
  
058311 AAATCAAAGA CATACTGAAT GCTACTGCGG GTACATGCGA A"GAAATGATG AAAAGGGCTA TATTTGCCCG "  
       "TTTAGTTTCT GTATGACTTA CGATGACGCC CATGTACGCT T"CTTTACTAC TTTTCCCGAT ATAAACGGGC   
  
058381 "AGAATTGGGA GCTCCTATCG TAATGCATGA CTACTTAACA G"GTGGATTCA CTGCAAATAC TAGCTTGGCT   
       TCTTAACCCT CGAGGATAGC ATTACGTAC"T GATGAATTGT CCACCTAAGT GACGTTTATG ATCGAACCGA "  
  
058451 CATTATTGCC GAGATAATGG TCTACTTCTT CACATTCACC GTGCAATGCA TGCTGTTATT GATAGACAGA   
       "GTAATAACGG CTCTATTACC AGATGAAGA"A GTGTAAGTGG CACGTTACGT ACGACAATAA CTATCTGTCT   
  
058521 AGAATCATGG TATGCACTTC CGTGTACTAG CTAAAGCGTT ACGTATGTCT GGTGGAGACC ATATTCACGC   
       TCTTAGTACC ATACGTGAAG GCACATGATC GA"TTTCGCAA TGCATACAGA CCACCTCTGG TATAAGTGCG "  
  
058591 TGGTACCGTA GTAGGTAAAC TTGAAGGGGA AAGAGAAATC ACTTTAGGCT TTGTTGATTT ACTACGTGAT   
       "ACCATGGCAT CATCCATTTG AACTTCCCCT TT"CTCTTTAG TGAAATCCGA AACAACTAAA TGATGCACTA   
  
058661 GATTTTGTTG AAAAAGACCG AAGCCGCGGT ATTTATTTCA CTCAAGAT"TG GGTCTCTTTA CCAGGTGTTC "  
       CTAAAACAAC TTTTTCTGGC TTCGGCGCCA TAAATAAAG"T GAGTTCTAAC CCAGAGAAAT GGTCCACAAG "  
  
058731 "TGCCAGTGGC TTCCGGTGGT ATTCACGTTT GGCATATGCC TGCTCTAA"CC GAGATTTTTG GAGATGATTC   
       "ACGGTCACCG AAGGCCACCA TAAGTGCAAA CCGTATACG"G ACGAGATTGG CTCTAAAAAC CTCTACTAAG   
  
058801 TGTACTACAA TTCGGCGGAG GAACTTTGGG GCACCCTTGG GGTAATGCAC CTGGTGCCGT AGCTAACCGA   
       ACATGATGTT AAGCCGCCTC CTTGAAACC"C CGTGGGAACC CCATTACGTG GACCACGGCA TCGATTGGCT "  
  
058871 GTAGCTCTAG AAGCATGTGT ACAAGCTCGT AATGAGGGAC GTGATCTTGC TCGTGAGGGT AATGAAATTA   
       "CATCGAGATC TTCGTACACA TGTTCGAGC"A TTACTCCCTG CACTAGAACG AGCACTCCCA TTACTTTAAT   
  
058941 TCCGTGAGGC TAGTAAATGG AGTCCTGAAC TAGTCTGCGT GACTTGGAGG AAGTATGGTA AGGAGATCAA   
       AGGCACTCCG ATCATTTACC TCAGGACTTG ATCA"GACGCA CTGAACCTCC TTCATACCAT TCCTCTAGTT "  
  
059011 ATTTGAATTT GAGGCAATGG ATACTTTGTA AGCCAGTAAT TAC"TCTTCGT TCTTTTAATT GACTTTCAAT "  
       "TAAACTTAAA CTCCGTTACC TATGAAACAT TCGG"TCATTA ATGAGAAGCA AGAAAATTAA CTGAAAGTTA   
  
059081 "TGAAACTGCA AACTCGGCCC AATCTTTTAC CAAAAGGATT GAG"CCGAATA CAATACAAAG ATATTATTGT   
       ACTTTGACGT TTGAGCCGGG TTAGAAAATG "GTTTTCCTAA CTCGGCTTAT GTTATGTTTC TATAATAACA "  
  
059151 ATATCTTTTT ACTAGATATA CAAAATAGAA AATTGAAATA AAAAT"ATAAG AGGAAACGAA ACAACTTAAA "  
       "TATAGAAAAA TGATCTATAT GTTTTATCTT" TTAACTTTAT TTTTATATTC TCCTTTGCTT TGTTGAATTT   
  
059221 "CATTTTTTTT TATTGTTGTG CTGGATCCGC AATTAAGCCT ATGGA"TCCTT AGGATTGGTG TAGTCGGTTA   
       GTAAAAAAAA ATAACAACAC GACCTAGGCG TTAATT"CGGA TACCTAGGAA TCCTAACCAC ATCAGCCAAT "  
  
059291 ATATATTATA TCCCGTAGCG CGTTT"CAGAT CCGGGATCGA GCCAAGTATC ACAACTTCTT CTACCCATCC "  
       "TATATAATAT AGGGCATCGC GCAAAGTCTA GGCCCT"AGCT CGGTTCATAG TGTTGAAGAA GATGGGTAGG   
  
059361 "TGTATATTGC CTTTTTGTCT GTCTT"GGAAT AAAAAATTTT TATTTTTAGT AGACGAGATT TTACGAAAAA   
       ACATATAACG GAAAAACAGA CAGAACCTTA TTT"TTTAAAA ATAAAAATCA TCTGCTCTAA AATGCTTTTT "  
  
059431 AGTCTTCATA TTCATAGGGA GACAACAATT TTT"TTTTTAT GTGAATTTGA CAATTTGGCA TAACCGGTGA "  
       "TCAGAAGTAT AAGTATCCCT CTGTTGTTAA AAA"AAAAATA CACTTAAACT GTTAAACCGT ATTGGCCACT   
  
059501 "GAAACCACTA TTTAGAAAAT AATAATTTAA TTG"AAAAAAA AAAAATATAT AATTCTCTTT GGCGAGAACG   
       CTTTGGTGAT AAATCTTTTA TTATTAAATT AACTTTTTTT TTTTTAT"ATA TTAAGAGAAA CCGCTCTTGC "  
  
059571 CTAAGTATCA CTTCACTATA TATATAACGG AACCTTTTTT TTTA"AGACCG ACTCGTTAAT AGTTAATAAT "  
       "GATTCATAGT GAAGTGATAT ATATATTGCC TTGGAAAAAA AAATTCT"GGC TGAGCAATTA TCAATTATTA   
  
059641 "CCTAGTGATT GGATTTAGAT CCTATTCTGA TAGGAAATGA GATA"TTCAAA TGATTTTTTT TATCGAATGA   
       GGATCACTAA CCTAAATCTA GGATAAGACT ATCCTTTACT CTAT"AAGTTT ACTAAAAAAA ATAGCTTACT "  
  
059711 CTATTCATCT ATTGTATTTT CATGTAAATA GGGGCAAGAA AGCTCTATGG AAAAAAGATG GTTAAATTC"G "  
       "GATAAGTAGA TAACATAAAA GTACATTTAT CCCCGTTCTT TCGA"GATACC TTTTTTCTAC CAATTTAAGC   
  
059781 "ATGTTGTCTA AAGGGGAGTT AGAATACAGG TGTAGGCTAA GTAAATCAAT CAATAGTCTT GGTCCTATT"G   
       TACAACAGAT TTCCCCTCAA TCTTATGTC"C ACATCCGATT CATTTAGTTA GTTATCAGAA CCAGGATAAC "  
  
059851 AAAGTGAAGG ATCAATTATA AATAATATGA ATAAAAACAT TCCTAGTCAT AGTGATAGTT ACAATTCTAG   
       "TTTCACTTCC TAGTTAATAT TTATTATAC"T TATTTTTGTA AGGATCAGTA TCACTATCAA TGTTAAGATC   
  
059921 TTACAGTACT GTTGATGATT TAGTCGGCAT TCGAAATTTC GTATCTGATG ACACTTTTTT AGTTAGGGAT   
       AATGTCATGA CAACTACTAA ATCAGCCGTA A"GCTTTAAAG CATAGACTAC TGTGAAAAAA TCAATCCCTA "  
  
059991 AGTAATAGCA GCAGTTATTC CATATATTTG GATATTGAAA ATCAAATTTT TGAGATTGAC AATGATCCTT   
       "TCATTATCGT CGTCAATAAG GTATATAAAC C"TATAACTTT TAGTTTAAAA ACTCTAACTG TTACTAGGAA   
  
060061 CTTTTGTAAG TGAACTAGAA AGTTCTTTTT ATAGTTTTCG GAACTCTACT TATCAAAATA ATATATCTAA   
       GAAAACATTC ACTTGATCTT TCAAGAAAAA TATCAAAA"GC CTTGAGATGA ATAGTTTTAT TATATAGATT "  
  
060131 GAATGATGAT TCCCACTATG ATCGTTACAT GTATGATACT AAATATAGTT GGAATAATCA CATTAATAGT   
       "CTTACTACTA AGGGTGATAC TAGCAATGTA CATACTAT"GA TTTATATCAA CCTTATTAGT GTAATTATCA   
  
060201 TGCATTGACA GTTATCTTCG GACTCAAATC TGTATTGATA GTTACATTTT AAGTGGTAGT CACAATTACA   
       ACGTAACTGT CAATAGAAGC CTGAGTTTAG ACATAACTAT CAATGTA"AAA TTCACCATCA GTGTTAATGT "  
  
060271 GTGACAGTTA CATTTATAGT TATATTTGTG GCGAAGGTGG AAATAGTAGT GAAAGTGAGA GTTTCAGTAT   
       "CACTGTCAAT GTAAATATCA ATATAAACAC CGCTTCCACC TTTATCA"TCA CTTTCACTCT CAAAGTCATA   
  
060341 AAGAACTAGC ACGCATGGAA ATAATTTAAC TATAACAGAA AGTTCTAATG ATCTAGATAA T"GATCGAACT "  
       TTCTTGATCG TGCGTACCTT TATTAAATTG ATATTGTCTT T"CAAGATTAC TAGATCTATT ACTAGCTTGA "  
  
060411 "ACAAACTACA GCGATTTTTG GGTTATATGC GAAAATTGTC ATAAATTCAA TTATAAGAGA C"TTTTCAAAT   
       "TGTTTGATGT CGCTAAAAAC CCAATATACG CTTTTAACAG T"ATTTAAGTT AATATTCTCT GAAAAGTTTA   
  
060481 CAAAAATGAA TATTTGTGAA GAATGTGGAT ACCATTTGAA AATGAATAGT TCAGATAGAA TCGAACTTTT   
       GTTTTTACTT ATAAACACTT CTTACACCTA TGGTAAACTT TTACTT"ATCA AGTCTATCTT AGCTTGAAAA "  
  
060551 GATTGA"TCCA GGTACTTGGG ACGCTCTATG GTATGTACAG ACATGGTCTC TGTGGATCCC ATATGAATGG "  
       "CTAACTAGGT CCATGAACCC TGCGAGATAC CATACATGTC TGTACC"AGAG ACACCTAGGG TATACTTACC   
  
060621 "GATTCG"GAGG TGGATCCCAT TCAATGGAAG TCGCAGGTGG ATCCCATTGA AGGGGATTCG GAGGTGGATC   
       CTAAGCCTCC ACCTAGGGTA AGTTACCTTC AGCGTCCACC TAGGGTA"ACT TCCCCTAAGC CTCCACCTAG "  
  
060691 TCATTGAAGG GGATTCGGAG GAGTACAAGG ACTCTTCTTA TAAAGATCGT ATTTCTTCTT CTCAAATAGA   
       "AGTAACTTCC CCTAAGCCTC CTCATGTTCC TGAGAAGAAT ATTTCTA"GCA TAAAGAAGAA GAGTTTATCT   
  
060761 GACAGGATTA CCTGAGGCTA TTCAAACAGG CACAGGTAAA TTAAATGGTA TTCCCGTAGC AATTGGGGTT   
       CTGTCCTAAT GGACTCCGAT AAGTTTGTCC GTGTCCATTT AATTTA"CCAT AAGGGCATCG TTAACCCCAA "  
  
060831 ATGGAGTTTG AATTTATGGG GGGTAGTATG GGATCTGTAG TAGGCGAGAA AATCACCCGT TTGATCGACT   
       "TACCTCAAAC TTAAATACCC CCCATCATAC CCTAGACATC ATCCGC"TCTT TTAGTGGGCA AACTAGCTGA   
  
060901 ATGCTAGCAA TCAATTTTTA CCTCTTATTT TAGTGTGTGC TTCGGGAGGA GCACGCATGC AAGAAGGAAG   
       TACGATCGTT AGTTAAAAAT GGAGAATAAA ATCACAC"ACG AAGCCCTCCT CGTGCGTACG TTCTTCCTTC "  
  
060971 TTTGAGCTTG ATGCAAATGG CAAAAATATC TTCTGCTTTA TATGATTATA AATATCAATC AAATAAAAAA   
       "AAACTCGAAC TACGTTTACC GTTTTTATAG AAGACGA"AAT ATACTAATAT TTATAGTTAG TTTATTTTTT   
  
061041 TTATTCTATG TAGCAATCCT TACATCTCCT ACTA"CTGGTG GGGTGACAGC TAGTTTTGCT ATGTTGGGGG "  
       AATAAGATAC ATCGTTAGGA ATGTAGAGGA TGATGA"CCAC CCCACTGTCG ATCAAAACGA TACAACCCCC "  
  
061111 "ATATCATTAT TGCCGAACCG AATGCCTACA TCGC"ATTTGC GGGTAAAAGA ATAATTGAAG AAACATTGAA   
       "TATAGTAATA ACGGCTTGGC TTACGGATGT AGCGTA"AACG CCCATTTTCT TATTAACTTC TTTGTAACTT   
  
061181 GATGGAAGTC CCTGAGGGTT CACAGAAGAC TGAACCTTTA TTCGAAAAGG GCTTATTAGA TCTAATCGTA   
       CTACCTTCAG GGACTCCCAA GTGTCTTCTG ACTTGGAAAT AAGCTTT"TCC CGAATAATCT AGATTAGCAT "  
  
061251 CCACGTAATC CTTTAAAAGA TGTTGTGAGT GAGTTATTTC AGCTCCACGC TTTCGTTCCC TCGAATCAAA   
       "GGTGCATTAG GAAATTTTCT ACAACACTCA CTCAATAAAG TCGAGGT"GCG AAAGCAAGGG AGCTTAGTTT   
  
061321 ATTCAATCAA GTAAAGCCTT ACTTAAAACT TCAAAAATGA ATTCTTTTTT TTTTTATTTC TT"CCGCAAAA "  
       TAAGTTAGTT CATTTCGGAA TGAATTTTGA AGTTTTTACT TAAGAAA"AAA AAAAATAAAG AAGGCGTTTT "  
  
061391 "TTTGGCACGG GGAATAAAAT GAATTTAATG CTCCCCTCTT AACATACATG TGTATATATA CT"TCAACTAT   
       "AAACCGTGCC CCTTATTTTA CTTAAATTAC GAGGGGAGAA TTGTATG"TAC ACATATATAT GAAGTTGATA   
  
061461 AGCAAATAGC GGCATAGAGT CTTGCATCTT TCTACATCTC TAGAGGATTT CTAGTTTTA"C TAAGAATCCC "  
       TCGTTTATCG CCGTATCTCA GAACGTAGAA AGATGTA"GAG ATCTCCTAAA GATCAAAATG ATTCTTAGGG "  
  
061531 "TGTTGTTGGA TCGGATTATA ACTACCTTTT GGGGAATTTG TCGAAAATCT TTATGAAAT"T TTATTAAAAA   
       "ACAACAACCT AGCCTAATAT TGATGGAAAA CCCCTTA"AAC AGCTTTTAGA AATACTTTAA AATAATTTTT   
  
061601 AAAATGCTAA GACAAGATAA TAACTAAAAT AATGCAAACG TCTATTATGA TACAGATATT "TCATGCGAAA "  
       TTTTACGATT CTGTTCTATT ATTGATTTTA "TTACGTTTGC AGATAATACT ATGTCTATAA AGTACGCTTT "  
  
061671 "GATGAGTAAG CCATTTATTT AATTCGACAT TCCTCATTCC TTTTCTATAT ATACTCACGT" AGATATTACT   
       "CTACTCATTC GGTAAATAAA TTAAGCTGTA" AGGAGTAAGG AAAAGATATA TATGAGTGCA TCTATAATGA   
  
061741 TAGTATAATT ATATATTGAA TTAGTATAAT TATAAGATAC CTTTTATAAC AATCAATAAA TAACAGGTAA   
       ATCATATTAA TATATAACTT AATCATATTA ATATTCTATG GA"AAATATTG TTAGTTATTT ATTGTCCATT "  
  
061811 AACTATTAAT ATAACAATTA ATATAACAAT AAATACAGGT CCAAATATTA AGTCGAGGTA TCCATTCTAT   
       "TTGATAATTA TATTGTTAAT TATATTGTTA TTTATGTCCA GG"TTTATAAT TCAGCTCCAT AGGTAAGATA   
  
061881 GACAA"CTCTC ACCACCTTAC CCTCTATTTT TGTGCCTTTA GTGGGCCTAG TATTTCCGGC AATTGCAATG "  
       CTGTTGAGAG TGGTGGAATG GGAGATAAAA ACACGGAA"AT CACCCGGATC ATAAAGGCCG TTAACGTTAC "  
  
061951 "GCTTC"TTTAT TTCTTCATGT TCAAAAAAAC AAGATTTTTT AAATATGCTA GTGCCGTCTA TTTTTTTTTC   
       "CGAAGAAATA AAGAAGTACA AGTTTTTTTG TTCTAAAA"AA TTTATACGAT CACGGCAGAT AAAAAAAAAG   
  
062021 AA"CACTTAGG CTTTGACGAT AACACAGCTA TCTATTTAGT AGACTAGAGT CTAACATGTG CGCTTACTCC "  
       TTGTGAATCC GAAACTGCTA TTGTGTCGAT AGATAAATCA "TCTGATCTCA GATTGTACAC GCGAATGAGG "  
  
062091 "AA"ACATAAGC GATTATACAT ACGTAAACAT GATATCTGAG GAAATAAATT TGAAGTATTT CAAAATAGAA   
       "TTTGTATTCG CTAATATGTA TGCATTTGTA CTATAGACTC" CTTTATTTAA ACTTCATAAA GTTTTATCTT   
  
062161 AA"TATATAAG AGATCGGGCG ACGAATGTTT GAGATATAAA ATCAATATAT CTATCTGGAT GTAGGTATCT "  
       TTATATATTC TCTAGCCCGC TGCTTACAAA CTCTATATTT TAGTTAT"ATA GATAGACCTA CATCCATAGA "  
  
062231 "AT"AGGGAATC ATATAAAGGG TGATGTTATT ATTTTAGATC TAAGTAATTC AT"CGAATTAC TTAGATCTAA "  
       "TATCCCTTAG TATATTTCCC ACTACAATAA TAAAATCTAG ATTCATT"AAG TAGCTTAATG AATCTAGATT   
  
062301 "AGGAAAGGTT CACATCAAAA TAGTGCTAGT TGATTAGAGT TACTTCGGAA AC"AAAAAAAG TAAAATAGAT   
       TCCTTTCCAA GTGTAGTTTT ATCACGATCA ACTAATCTCA ATGA"AGCCTT TGTTTTTTTC ATTTTATCTA "  
  
062371 TTTTTTTGAT TCTATCAATT CCAATAAAAT GCAACGAGAT CTTAGTATGA CATTG"GCGAT CAGAACGTAT "  
       "AAAAAAACTA AGATAGTTAA GGTTATTTTA CGTTGCTCTA GAAT"CATACT GTAACCGCTA GTCTTGCATA   
  
062441 "ATGATAGGAT TTCTAAGAGG ATCTCGAAAA ATCAGCAATT TCTGCTGGGC CTTTA"TCCTT TTTTTAGGTT   
       TACTATCCTA AAGATTCTCC TAGAGCTTTT TAGTCGTTAA AGACGA"CCCG GAAATAGGAA AAAAATCCAA "  
  
062511 CATTAGGGTT TTTATTGGTT GGAACTTCCA GTTATCTTGG TAGGGATTTG ATACCTTTAT TTCCGTCTCA   
       "GTAATCCCAA AAATAACCAA CCTTGAAGGT CAATAGAACC ATCCCT"AAAC TATGGAAATA AAGGCAGAGT   
  
062581 GCAAATAATT TTTTTTCCAC AGGGGATCGT AATGTCTT"TC TATGGGATTG CCGGTCTCTT TATTAGCTCC "  
       CGTTTATTAA AAAAAAGGTG TCCCCTAGCA TTACAGAAA"G ATACCCTAAC GGCCAGAGAA ATAATCGAGG "  
  
062651 "TATTTGTGGG GCACAATTTT GTGGAATGTA GGTAGCGG"TT ATGATCGATT CGATAGAAAA GAAGAAATAG   
       "ATAAACACCC CGTGTTAAAA CACCTTACAT CCATCGCCA"A TACTAGCTAA GCTATCTTTT CTTCTTTATC   
  
062721 TGTCTATTTT "TCGTTGGGGG TTTCCTGGAA AAAATCGTCG AATCTTCCTA CGATTCCTTA TGAAAGACAT "  
       ACAGATAAAA AGCAACCCCC AAAGGACCTT TTTTAGCAGC TTAGAA"GGAT GCTAAGGAAT ACTTTCTGTA "  
  
062791 "TCAGTCCATC" AGAATAGAAG TTAAAGAGGG TATTTATGCA "CGTCGTGTCC TTTATATGGA AATAAGAGGC "  
       "AGTCAGGTAG TCTTATCTTC AATTTCTCCC ATAAATACGT GCAGCA"CAGG AAATATACCT TTATTCTCCG   
  
062861 "CAAGGGGCCA TTCCCTTGAC TCGTACAGAT CAGAATCTGA" CCCCACGAGA AATTGAGCAA AAGGCTGCCG   
       GTTCCCCGGT AAGGGAACTG AGCATGTCTA GTCTTA"GACT GGGGTGCTCT TTAACTCGTT TTCCGACGGC "  
  
062931 AATTGGCCTA TTTCTTGCGT GTACCAATTG AAGTTTTTTG AAAAATGAAG TTCAGA"ATGA ATTTTGTTCG "  
       "TTAACCGGAT AAAGAACGCA CATGGTTAAC TTCAAA"AAAC TTTTTACTTC AAGTCTTACT TAAAACAAGC   
  
063001 "TAGCAAGAGG GATAAAACGG AAATGAAAGA ATAGTCTTTT TTATAGGCCA TAGGAA"TAGT ATAATAACTT   
       ATCGTTCTCC CTATTTTGCC TTTACTTTCT TATCAGAAAA AATAT"CCGGT ATCCTTATCA TATTATTGAA "  
  
063071 AACTGGAATT TCTTCAGAAT GCTCATTTGC CCCAAAGCA"G ACGTATACGG AACAGCCAGA AAACTGTCTT "  
       "TTGACCTTAA AGAAGTCTTA CGAGTAAACG GGGTTTCGTC TGCAT"ATGCC TTGTCGGTCT TTTGACAGAA   
  
063141 "TGTCCGAAAT TTTGATCCGT ATGTAAATCA ACTCCACAG"T AACAAAAAAA AGTGGATTCT TTGTTATTTT   
       ACAGGCTTTA AAACTAGGCA TACATTTA"GT TGAGGTGTCA TTGTTTTTTT TCACCTAAGA AACAATAAAA "  
  
063211 CTTTCTGTAT TATTTCGAAA TTCAAGGATT AACTAATGAA TCACAAA"TCA AAAACGAAAA AACAGGGAAT "  
       "GAAAGACATA ATAAAGCTTT AAGTTCCT"AA TTGATTACTT AGTGTTTAGT TTTTGCTTTT TTGTCCCTTA   
  
063281 "GGATTCATAA TAGTATCCAT GTGGTCTTGT AATAGAACTT ATGTTTG"ATA TAAATATTAG TAGTGTAGAG   
       CCTAAGTATT ATCATAGGTA CACCAGA"ACA TTATCTTGAA TACAAACTAT ATTTATAATC ATCACATCTC "  
  
063351 AGTTAACGAA TGAAGTGAGT TCATTAAAAA ATAAAAGACG AAAAAATGGA AAAAAAGAAA GCATTCATTC   
       "TCAATTGCTT ACTTCACTCA AGTAATT"TTT TATTTTCTGC TTTTTTACCT TTTTTTCTTT CGTAAGTAAG   
  
063421 CCCTTCTATA TCTTGCATCT ATAGTATTTT T"GCCCTGGTG GATCTCTCTT TCATTTAATA AAAGCCTAGA "  
       GGGAAGATAT AGAACGTAGA TATCATAAAA A"CGGGACCAC CTAGAGAGAA AGTAAATTAT TTTCGGATCT "  
  
063491 "ATCTTGGGTT ACTAATTGGT GGAATACTGG T"CAATCCGAA AATTTTTTGA ATGATATTCA AGAAAAGAGT   
       "TAGAACCCAA TGATTAACCA CCTTATGACC A"GTTAGGCTT TTAAAAAACT TACTATAAGT TCTTTTCTCA   
  
063561 ATTATAAAAA AAGTTCTAGA ATTAGAGGAA CT"CTTTCTCT TGGACGAAAT GCTAAAGGAA TACCCAGAAA "  
       TAATATTTTT TTCAAGATCT TAATCTCCTT GA"GAAAGAGA ACCTGCTTTA CGATTTCCTT ATGGGTCTTT "  
  
063631 "CACATCTACA AAAGCTTCGT ATCGCAATCT CC"AAAGAAAC GATCCAATTG ATCAAGATGC ACAATGAGGA   
       "GTGTAGATGT TTTCGAAGCA TAGCGTTAGA GG"TTTCTTTG CTAGGTTAAC TAGTTCTACG TGTTACTCCT   
  
063701 TCGTATCCAT ACGATTTTGC ACTTCTCGAC AAATATAATC TGTTTCGTTA TTCTAAGTGG TTATTCTATT   
       AGCATAGGTA TGCTAAAACG TGAAGAGCTG TTTATATTAG ACA"AAGCAAT AAGATTCACC AATAAGATAA "  
  
063771 "CTTGGTAATG AAGAACTTGT TATTCTTAAC TCTTGGGTTC AAGAGTTCCT ATATAACTTA AGCGACACAA"   
       "GAACCATTAC TTCTTGAACA ATAAGAATTG AGAACCCAAG TTC"TCAAGGA TATATTGAAT TCGCTGTGTT   
  
063841 TAAAAGCTTT TTCTATTCTT TTATTAACTG ATTTATGTAT AGGATTCCAT TCACCCCATG GTTGGGAACT   
       ATTTTCGAAA AAGATAAGAA AATAATTGAC TAAATACAT"A TCCTAAGGTA AGTGGGGTAC CAACCCTTGA "  
  
063911 AATGATTGGT TCTGTCTACA AAGATTTTGG ATTTTCTCAT AACGATCAAA TTATATCCAG TCTTGTTTCC   
       "TTACTAACCA AGACAGATGT TTCTAAAACC TAAAAGAGT"A TTGCTAGTTT AATATAGGTC AGAACAAAGG   
  
063981 ACTTTTCCA"G TCATTCTTGA CACAATTTTT AAATATTGGA TCTTCCGTTA TTTAAATCGT GTATCTCCGT "  
       TGAAAAGGTC AGTAAGAACT GTGTTAAAAA TTTATAACCT A"GAAGGCAAT AAATTTAGCA CATAGAGGCA "  
  
064051 "CACTTGGAG"T GATTTATCAT TCACTGAATG ACTGAAAAAT ATAATGTTTG TTACTTTGTA CATAAGTAAA   
       "GTGAACCTCA CTAAATAGTA AGTGACTTAC TGACTTTTTA T"ATTACAAAC AATGAAACAT GTATTCATTT   
  
064121 ACATTCAAAA TTGTACTTAT "TCCTTCTACC CATCGCGAAG GATGCCTCCT ATTCGAGTAA CTTTATTTCA "  
       TGTAAGTTTT AACATGAATA AGGAAGATGG GTAGCGCTTC CTAC"GGAGGA TAAGCTCATT GAAATAAAGT "  
  
064191 "GTAAATAGCA GAATCATGGA" TAGGGAACTA TACTAGGGAC CTACCTAATT TTATTGTAGA AATTTTTGGG   
       "CATTTATCGT CTTAGTACCT ATCCCTTGAT ATGATCCCTG GATG"GATTAA AATAACATCT TTAAAAACCC   
  
064261 CTCAATGATT GGACCATGCA AACTAGAAAT ACCTTTTCTT CGATAAAGGA AGAGATTACT CGATCTATTT   
       GAGTTACTAA CCTGGTACGT TTGATCTTTA TGGAAAAGAA GCTATTT"CCT TCTCTAATGA GCTAGATAAA "  
  
064331 CCGTATCACT CATGATATAT ATAATAACTT GGGCACCCGT TTCA"AATGCA TATCCCATTT TTGCACAACA "  
       "GGCATAGTGA GTACTATATA TATTATTGAA CCCGTGGGCA AAGTTTA"CGT ATAGGGTAAA AACGTGTTGT   
  
064401 "GGGGTATGAA AATCCACGAG AAGCAACCGG CCGTATTGTC TGCG"CCAACT GCCATTTAGC TAATAAACCC   
       CCCCATACTT TTAGGTGCTC TTCGTTGGCC GGCATAACA"G ACGCGGTTGA CGGTAAATCG ATTATTTGGG "  
  
064471 GTGGATATCG AGGGTCCACA AGCGGTACTT CCTGATACTG TATTTGAAGC AGGTGGTCGA ATCCCTTATG   
       "CACCTATAGC TCCCAGGTGT TCGCCATGAA GGACTATGA"C ATAAACTTCG TCCACCAGCT TAGGGAATAC   
  
064541 ATATGCAACT GAAACAAGTT C"TTGCGAATG GGAAAAAGGG CTGGTTGAAT GTGGGGGCCG GTCTTATTTT "  
       TATACGTTGA CTTTGTTCAA GAACGCTTAC CCTTTTTCCC "GACCAACTTA CACCCCCGGC CAGAATAAAA "  
  
064611 "ACCCGAAGGG TTTGAATTAG C"CCCCCCCGA TCGGATTTCT CCCGAGATTA AAGAAAAAAA GGGCCAATCT   
       "TGGGCTTCCC AAACTTAATC GGGGGGGGCT AGCCTAAAGA" GGGCTCTAAT TTCTTTTTTT CCCGGTTAGA   
  
064681 GTGCTTTTCA GAGCCTATCG TCCCACGAAA AAAAATATTC TTGTGATAGG TCCTGTTCCT GGTCAGAAAT   
       CACGAAAAGT CTCGGATAGC AGGGTGCTTT TTTTTATAAG AAC"ACTATCC AGGACAAGGA CCAGTCTTTA "  
  
064751 ATAGTGAAAT CACCTTTCC"T ATTCTTTCCC CGGACCCTGC GACTAAGAAA GATGTTCACT TCTTAAAATA "  
       "TATCACTTTA GTGGAAAGGA TAAGAAAGGG GCCTGGGACG CTG"ATTCTTT CTACAAGTGA AGAATTTTAT   
  
064821 "TCCCATATAC GTAGGCGGG"A ACAGGGGAAG GGGTCAGATT TATCCCGACG GGAGTAAGAG TAATAATAAT   
       AGGGTATATG CATCCGCCCT TGTCCCCTT"C CCCAGTCTAA ATAGGGCTGC CCTCATTCTC ATTATTATTA "  
  
064891 GTTTATAATG CTACAGCAGC AGGGATAGTA AGTAAAATAA TACGAAAAGA AAAAGGAGGA TATGAAATAA   
       "CAAATATTAC GATGTCGTCG TCCCTATCA"T TCATTTTATT ATGCTTTTCT TTTTCCTCCT ATACTTTATT   
  
064961 CCATAGTGGA TGCCGCGAAT GGGCGTCAAG TGGTTGATAT TATCCCTCCA "GGACCAGAAC TTCTTGTTTC "  
       GGTATCACCT ACGGCGCTTA CCCGCAGTTC ACCAACTATA ATAGGG"AGGT CCTGGTCTTG AAGAACAAAG "  
  
065031 "AGAGGGCGAA TCCTTCAAAC TTGATCACCC ATTAACGAGT AATCCTAATG" TGGGAGGATT TGGTCAGGGA   
       "TCTCCCGCTT AGGAAGTTTG AACTAGTGGG TAATTGCTCA TTAGGA"TTAC ACCCTCCTAA ACCAGTCCCT   
  
065101 GATGCGGAAA TCGTCCTTCA AGATCCATTA CGTGTCCAAG GCCTTTTGTT CTTTTTTGCA TCTGTTATTT   
       CTACGCCTTT AGCAGGAAGT TCTAGGTAAT GCACAGGTTC CGGAAAA"CAA GAAAAAACGT AGACAATAAA "  
  
065171 TGGCACAAAT CTTTTTAGTT CTTAAAAAGA AACAGTTTGA GAAGGTCAAT TGTCCGAAAT GAATTTCTAA   
       "ACCGTGTTTA GAAAAATCAA GAATTTTTCT TTGTCAAACT CTTCCAG"TTA ACAGGCTTTA CTTAAAGATT   
  
065241 ATCTGGGGAT TTCTCAAATA AAGTTCGGAA AAAA"AACAAA ATTCTTGTTG GTAATTCTCT AATTTAATTA "  
       TAGACCCCTA AAGAGTTTAT TTCAAGCCTT TTTT"TTGTTT TAAGAACAAC CATTAAGAGA TTAAATTAAT "  
  
065311 "TGTATCATCA AAAAATGCTG AAAAACTTTT TCCC"TTGTTT TTGTTTATAT TCTTTTTCGA CCGTATTTCG   
       "ACATAGTAGT TTTTTACGAC TTTTTGAAAA AGGG"AACAAA AACAAATATA AGAAAAAGCT GGCATAAAGC   
  
065381 GGAATGACTT GGCCGCATTA CTAGTCATAG TATGGATATT GT"CAGGAAGA CTATTTTACC TCTTCTTTAT "  
       CCTTACTGAA CCGGCGTAAT GATCAGTATC ATACCTATAA CA"GTCCTTCT GATAAAATGG AGAAGAAATA "  
  
065451 "TTCTTTGTTT TTTACAATAC AAATGGGAGC GGTATTATTA TG"ATTATTAT GTCACAGGTG CCAGATTGAA   
       "AAGAAACAAA AAATGTTATG TTTACCCTCG CCATAATAAT AC"TAATAATA CAGTGTCCAC GGTCTAACTT   
  
065521 ATGTCATTGA ATGTGTCTAT TTTTATTCTA ATAGAATAAA AATAGA"GTAG ATATTAAAAA TAAGATAAGT "  
       TACAGTAACT TACACAGATA AAAATAAGAT TATCTTATTT TTATCT"CATC TATAATTTTT ATTCTATTCA "  
  
065591 "AAATGGAGAA TAGAAAAAAA ATGAAGGGAA CAAAGGATTC TAGAAG"GAAT TATTCATCTT TCTAGTATTC   
       "TTTACCTCTT ATCTTTTTTT TACTTCCCTT GTTTCCTAAG ATCTTC"CTTA ATAAGTAGAA AGATCATAAG   
  
065661 GATAGCAGAA AAATTCCTTT TCTTGTTCTT "GTGTCGAAAT AATAAACTAA TGAATTATTC TTGATCCCGT "  
       CTATCGTCTT TTTAAGGAAA AGAACAA"GAA CACAGCTTTA TTATTTGATT ACTTAATAAG AACTAGGGCA "  
  
065731 "TCGTCAAAGA TCCCTATTCT TAGTTTCAAT" TTTTCCCGGT TTATCAGAAC CTTTTTTTCG ATTTATTACA   
       "AGCAGTTTCT AGGGATAAGA ATCAAAG"TTA AAAAGGGCCA AATAGTCTTG GAAAAAAAGC TAAATAATGT   
  
065801 TAAGTAGTGA ACAAATTCAA TGAATTTTTA AATTCTATTT TATATTAAA"A TAACTAGAGA AAATTCTATT "  
       ATTCATCACT TGTTTAAGTT ACTTAAAAAT TTAAGATAAA ATAT"AATTTT ATTGATCTCT TTTAAGATAA "  
  
065871 "AGTATATTTT AATATATTTA AAGGGTTTGT GATATCTGAT CGATAGAAA"T ATATTATTGT TATATTGGGA   
       "TCATATAAAA TTATATAAAT TTCCCAAACA CTATAGACTA GCTA"TCTTTA TATAATAACA ATATAACCCT   
  
065941 TTGTGTCATC CAGAAAGAGG GAATCGAGTG ATTCTCTCTT T"CTTTTTTTC GCTTTGAAAT GAAAGTATCG "  
       AACACAGTAG GTCTTTCTCC CTTAGCTCAC TAAGAGAGAA A"GAAAAAAAG CGAAACTTTA CTTTCATAGC "  
  
066011 "ACCGATACGA TTGCGAAAGA GATGGTCTGA ATCAATTTTT T"GAAGCAATT CAGAATCAGA AAGAAATGAA   
       "TGGCTATGCT AACGCTTTCT CTACCAGACT TAGTTAAAAA A"CTTCGTTAA GTCTTAGTCT TTCTTTACTT   
  
066081 GTTTTTTTTG AAAAAATGGG AAAGATCCAT TTTTTCATTT ATATGAATA"A ATTAGTCTAA TTCTAATTAG "  
       CAAAAAAAAC TTTTTTACCC TTTCTAGGTA AAAAAGTAAA TATACTT"ATT TAATCAGATT AAGATTAATC "  
  
066151 "TAAGAGCTCA ACGGGACCTA CCCCCTCTTT TTTTTCTGAT TCGAGGGGG"A TCCTGTTGAG TTCTTACGCT   
       "ATTCTCGAGT TGCCCTGGAT GGGGGAGAAA AAAAAGACTA AGCTCCC"CCT AGGACAACTC AAGAATGCGA   
  
066221 TTCATGTCTA CAACTCAGTT CATCCGATTA TTACA"GAGAT GAACCTAAGC CGGAATATGA ACCATAAAAG "  
       AAGTACAGAT GTTGAGTCAA GTAGGCTAAT AATGTCTCTA CTTGGATTCG GCCTTATACT TGGTATTTTC   
  
066291 "AAAACACCGA CTAAACCGAT CACAGGAATA CCAGT"TACAG TACCGATTAG CCAAAGAGGA ATCCTTCCAG   
       TTTT"GTGGCT GATTTGGCTA GTGTCCTTAT GGTCAATGTC ATGGCTAATC GGTTTCTCCT TAGGAAGGTC "  
  
066361 TAGTCTCGGC CATTTGTCCC ACTTCCCTCC CCATTT"CATC AAGTGGTCGT GCTAGAGACA GAAACAGTCA "  
       "ATCA"GAGCCG GTAAACAGGG TGAAGGGAGG GGTAAAG"TAG TTCACCAGCA CGATCTCTGT CTTTGTCAGT "  
  
066431 "TAGATAATTA TGAGATGATA TCCTTCCGAA TGGGAT"AAGA TAATTCGTAC TATTATTTAG TATTTTCTTT   
       "ATCTATTAAT ACTCTACTAT AGGAAGGCTT ACCCTAT"TCT ATTAAGCATG ATAATAAATC ATAAAAGAAA   
  
066501 TATTTAATTG AAGAAATAAT TGGAAAATAA AACA"GCAAGT ACAAAAATGA GTAATAACCC CCAGTAAAGA "  
       ATAAATTAAC TTCTTTATTA ACCTTTTATT TTGT"CGTTCA TGTTTTTACT CATTATTGGG GGTCATTTCT "  
  
066571 "CTGGTACGAT TCAATTCAAC ATTTTGTTCG TTCG"GATTTG ATTGTGTCAT AGCTCTATAA TTCGGATTAG   
       "GACCATGCTA AGTTAAGTTG TAAAACAAGC AAGC"CTAAAC TAACACAGTA TCGAGATATT AAGCCTAATC   
  
066641 GTTTATCGTT GGATGAATTG CATTGCTGAT ATTGACCCTA AAAAAGAAAC "GGTAGGTACA GCTAGTCCGT "  
       CAAATAGCAA CCTACTTAAC "GTAACGACTA TAACTGGGAT TTTTTCTTTG CCATCCATGT CGATCAGGCA "  
  
066711 "GAACAGTTAA CCATCGCACT GTAAAAATAG GATAAGTTTT ATCGATAGTC" ATTTGGTCCT CCTAAAAAGA   
       "CTTGTCAATT GGTAGCGTGA" CATTTTTATC CTATTCAAAA TAGCTATCAG TAAACCAGGA GGATTTTTCT   
  
066781 TCTACTAAAT TCATCGAGTT GTTCCAAAGA ATCAAAACGG CCAGTT"ATTA ATGGAATTCC TTGTCGGCTC "  
       AGATGATTTA AGTAGCTCAA CAAGGTTTCT TAGTTTTGCC GGTCAA"TAAT TACCTTAAGG AACAGCCGAG "  
  
066851 "TCTGTAAAAT ACTCATTTGG ACGAGGGCTT CCAAAAACAT CGTAAG"CTAA ACCCGTGCTG ACGAATAACC   
       "AGACATTTTA TGAGTAAACC TGCTCCCGAA GGTTTTTGTA GCATTC"GATT TGG"GCACGAC TGCTTATTGG "  
  
066921 AACCTGCAAT GAATAGAGAA GGTATAGTAA TGCTATGAAT GACCCAG"TAT CGAATACTGG TGATAATATC "  
       "TTGGACGTTA CTTATCTCTT CCATATCATT ACGATACTTA CTGGGTCATA GCT"TATGACC ACTATTATAG   
  
066991 "AGCAAAAGAA CGTTCTCCTG TGCTTCCAGA CATGCCGAGC TCCACAT"ATT CTTGACAGTC AAAAGGGGGA   
       TCGTTTTCTT GCAAGAGGAC ACGAAGGTCT GTACGGCTCG AGGTGTATAA GAACTG"TCAG TTTTCCCCCT "  
  
067061 TCGATTCCGT AAAAGATGAG ATCAGTAAAT GGAAATTAAC T"GAAATTCGA TCTTTGTAAG ATCGTCAATA "  
       "AGCTAAGGCA TTTTCTACTC TAGTCATTTA CCTTTAATTG ACTTTAAGCT AGAAAC"ATTC TAGCAGTTAT   
  
067131 "TTGACCGAGG GTGTTTTTAG AGTATACCGA ATCAGTATAG C"TATCCTTCT TCTCACGCAG CAACGCAATT   
       AACTGGCTCC CACAAAAATC TCATATGGCT TAGTCATATC GATAGGAAGA AGAGTGCGTC GTTGCGTTAA   
  
067201 TCACAATAAG TATAAAAATG AAGCACTAAA TAATATAATT TGTTTCTTCT "TTTCTTGGTG CTTGGCGATG "  
       AGT"GTTATTC ATATTTTTAC TTCGTGATTT ATTATATTAA ACAAAGAAGA AAAGAACCAC GAACCGCTAC "  
  
067271 "TAGAATCATG TACCATTCAT TCAATAGAAA ATTCTGACAA TGCCTGTCTG" TAGTATTAAA GATTCTCTCG   
       "ATC"TTAGTAC ATGGTAAGTA AGTTATCTTT TAAGACTGTT ACGGACAGAC ATCATAATT"T CTAAGAGAGC "  
  
067341 GGTATTGGCT TCGGGCTAAA AAGCCGAAGG TT"GGGTAAAA TGTGAATCCA ACTGGTTGAT TTATAATAAT "  
       "CCATAACCGA AGCCCGATTT TTCGGCTTCC AACCCATTTT ACACTTAGGT TGACCAACT"A AATATTATTA   
  
067411 "TCAGGAAGCA GATTCTAATA GTCCCAAAAT TC"AATTAAAT TAGCCGTGAA ATCTAGAAAT ATTTCTTTTG   
       AGTCCTTCGT CTAAGATTAT CAGGGTTTTA AGTTAATTTA ATCGGCACTT TAGATCTTTA TAAAGAAAAC   
  
067481 TGTTTGTAGT TATATTTTAG TTTGAATCTT TTTTTTTTTT TT"AGGAATTG GTTAGTCGTC CAGTAACAAG "  
       ACAAAC"ATCA ATATAAAATC AAACTTAGAA AAAAAAAAAA AATCCTTAAC CAATCAGCAG GTCATTGTTC "  
  
067551 "TAACTGTAGT ACATAGGATT TGATGAACGA AACAGAACAA CG"AATAGAAT AATAATAAAT ATTCGTAATG   
       "ATTGAC"ATCA TGTATCCTAA ACTACTTGCT TTGTCTTGTT GCTTATCTTA TTATTATTTA TAA"GCATTAC "  
  
067621 CACGCATTAT TGTTTTATAT TAGACTCAAA AGATCGTTCT TCAACTAATT "ACAAGAATGT TTTTAATATA "  
       "GTGCGTAATA ACAAAATATA ATCTGAGTTT TCTAGCAAGA AGTTGATTAA TGTTCTTACA AAA"ATTATAT   
  
067691 "ATATGGAAAG AATAAATGGA GAACCTAGTT TCGAGTGATC TGATCGTGCG" ATACTTTTTT CTTTTTTAAA   
       TATACCTTTC TTATTTACCT CTTGGATCAA AGCTCACTAG ACTAGCACGC TATGAAA"AAA GAAAAAATTT "  
  
067761 AAGATTTTAT GGGAATGAAC CCACTACAGA AAATCTAATT "AGTGGGAATA CAATTTAATA AATAAAGGGC "  
       "TTCTAAAATA CCCTTACTTG GGTGATGTCT TTTAGATTAA TCACCCTTAT GTTAAAT"TAT TTATTTCCCG   
  
067831 "ATTATAAGGT CATTCATTCT TCAGCGATTC AAAGAGCATT" TCTTTTTTGG AATCAAGTTA CACAACATAG   
       TAATATTCCA GTAAGTAAGA AGTCGCTAAG TTTCTCGTAA AGAAAAAACC TTAGTTCAA"T GTGTTGTATC "  
  
067901 TTTATTTATT ATTATTTTTT TTTCTAATTA TTTAGAATCC ATTTAATATT "AATAGATTTA TATATTTACT "  
       "AAATAAATAA TAATAAAAAA AAAGATTAAT AAATCTTAGG TAAATTATAA TTATCTAAA"T ATATAAATGA   
  
067971 "AGATTTATAT ATTAAATTAT TGTTTATAAA TTGTTTATAA TAAGAATATT" TGTTTTGACA ACTCAAGAGT   
       TCTAAATATA TAATTTAATA ACAAATATTT AACAAATATT ATTCTTATAA ACAAAA"CTGT TGAGTTCTCA "  
  
068041 TGTCCAATGA ATCCCTTGAT TCCGAATTGC GG"GATAGAGA GACAGATGAT GAATTGATTT CTTACCTATG "  
       "ACAGGTTACT TAGGGAACTA AGGCTTAACG CCCTATCTCT CTGTCTACTA CTTAAC"TAAA GAATGGATAC   
  
068111 "TCACAAGATT TTTGTTAGTA GCATCTATAA TC"ATAATAAT AGATGAATCA AAAACTTTCA ATTGAATATA   
       AGTGTTCTAA AAACAATCAT CGTAGATATT AGTATTATTA TCTACTTAGT TTTTGAAAGT TAACTTATAT   
  
068181 TTCTTTCAAT TGGTATTTTT ACTTATCCAT CCGCGTAT"CT TTCAAAAATG GAAACTTAGG GAAGTGCGTT "  
       AA"GAAAGTTA ACCATAAAAA TGAATAGGTA GGCGCATAGA AAGTTTTTAC CTTTGAATCC CTTCACGCAA "  
  
068251 "ATAACATATG GATAAAAATA ACGTATTTCA TTTAGCCT"CG TCATGCTTAC TAT"CACTAGT TATTTCGGTT "  
       "TA"TTGTATAC CTATTTTTAT TGCATAAAGT AAATCGGAGC AGTACGAATG ATAGTGATCA ATAAAGCCAA   
  
068321 "TTCTACTAGC AGTTTTAACT ATAACCTCAG CTCTATTTAT CGGTCTGAAC AAG"ATAAGAC TTATTTGATT   
       AAGAT"GATCG TCAAAATTGA TATTGGAGTC GAGATAAATA GCCAGACTTG TTCTATTCTG AATAAACTAA "  
  
068391 AAAATTAATT GAATGCACAA TTCAAAAAAA AGAAAGATTT CGGTGTTATT CAATATATTC TATATATT"GT "  
       "TTTTA"ATTAA CTTACGTGTT AAGTTTTTTT TCTTTCTAAA GCCACAATAA GTTATATAAG ATATATAA"CA "  
  
068461 "AGAGTTCTTT ACCTTGTCAA TTCAATTTCC AATTCTTGGT CATTGAGATT CATGGGCAAT ACCGATTA"AT   
       "TCTCAAGAAA TGGAACAGTT AAGTTAAAGG TTAAGAACCA GTAACTCTAA GTACCCGTTA TGGCTAAT"TA   
  
068531 TTTTTAAGGA TAGATATTAC CTCTCCTTTT CCTCGTTCAA CTAAATTGAA ATGATTGAAG TTTTTTTATT   
       AAAAATTCCT ATCTATAATG GAGAGGAAAA GGAGCAAGTT GATTTAACTT TACTAACTTC AAAAAAATAA   
  
068601 TGGAATCGTA TTAGGTCTAA TTCC"TATTAC TTTGGCTGGA TTATCTGTGA CCGCATATTT ACAATACAGA "  
       AC"CTTAGCAT AATCCAGATT AAGGATAATG AAACCGACCT AATAGACACT GGCGTATAAA TGTTATGTCT "  
  
068671 "CGGGGGGATC AGTTGGACCT TTGA"TTAATT AACAGTTCTT TTTGTTGATT GACCTCCTAC TTTGCTTT"AT "  
       "GC"CCCCCTAG TCAACCTGGA AACTAATTAA TTGTCAAGAA AAACAACTAA CTGGAGGATG AAACGAAATA   
  
068741 "AGGAGGTCAA ATTTTTATTT CTGTTGAATT ATTTCAGTAT AATTTTGATC TAACAAAACA AGAATCGA"AT   
       "TCCTCCAGTT TAAAAATAAA GACAACTTAA TAAAGTCATA TTAAAACTAG ATTGTTTTGT TCTTAGCTTA"   
  
068811 CACGCTCTAT AGGATTTGAA CCTACGACAT CGGGTTTTGG AGACCCGC"GT TCTACCGAAC TGAACTAAGA "  
       "GTGCGAGATA TCCTAAACTT GGATGCTGTA GCCCAAAACC TCTGGGCGCA AGATGGCTTG ACTTGATTCT"   
  
068881 "GCGTTTTATT ATCAAACTAA ATGCAACCCA AATATATCTT CCATACAT"AT ATTATCATAT AGAATATCAT   
       CGCAAAATAA TAGTTTGATT TACGTTGGGT TTATATAGAA GGTATGTATA TAATAGTATA TCTTATAGTA   
  
068951 AAAATTAAAC AAAAAAAGAT TGATTCGATA TATGTATGTT CAATTTTTAT GG"ATCTCAAT TGATTCCTCG "  
       TTTTAA"TTTG TTTTTTTCTA ACTAAGCTAT ATACATACAA GTTAAAAATA CCTAGAGTTA ACTAAGGAGC "  
  
069021 "TTACTGTTCA GAAGATAAGT AATAGGTAGG GATGACAGGA TTTGAACCCG TG"ACATTTTG TACCCAAAAC   
       "AATGAC"AAGT CTTCTATTCA TTATCCATCC "CTACTGTCCT AAACTTGGGC ACTGTAAAAC ATGGGTTTTG "  
  
069091 AAACGCGCTA CCAAGCTGCG CTACATCCCT TTCAATTGGT CTACAGTGTT "ATTGTAGAGA ATCCCTGTCT "  
       "TTTGCGCGAT GGTTCGACGC GATGTAGGGA" AAGTTAACCA GATGTCACAA TAACATCTCT TAGGGACAGA   
  
069161 "TGTTTTCCAC ATTTTTTTTT TATTTCCTCC ATTGGTATAC ACAAATTATC" TTGCCATTTC TTCTTTTTTG   
       ACAAAAGGTG TAAAAAAAAA ATAAAGGAGG TAACCATATG TGTTTAATAG AACGGTAAAG AAGAAAAAAC   
  
069231 TCTCATATCC TATATAAAAT ATAATAAAAA GACTTATAGA CGTATGAAA"T AAGAAATAGG AAATCCCCGT "  
       AGAGTATAGG ATATATTTTA TATT"ATTTTT CTGAATATCT GCATACTTTA TTCTTTATCC TTTAGGGGCA "  
  
069301 "AAAGAAAAAT GAATATTTGG GGTGGGGCAG AAAGCGACAT ATTTTTTTT"A ATAAAAAAGG GAATTTCCCC   
       "TTTCTTTTTA CTTATAAACC CCAC"CCCGTC TTTCGCTGTA TAAAAAAAAT TATTTTTTCC CTTAAAGGGG   
  
069371 CCGAATTGCA CATGTTGGCA CACATTCCAA TTTGAATTAG GAAAACCGCG GACAAAACA"A GCGGGTATTA "  
       GGCTTA"ACGT GTACAACCGT GTGTAAGGTT AAACTTAATC CTTTTGGCGC CTGTTTTGTT CGCCCATAAT "  
  
069441 "ACTATATATA AATTTGATGG TATGTAAACA CCAGTATGTA TTACCAATTA CTATCCTTA"T GTTTTCCAAA   
       "TGATAT"ATAT TTAAACTACC ATACATTTGT GGTCATACAT AATGGTTAAT GATAGGAATA CAAAAGGTTT   
  
069511 TTCCTATAAA GTAGGAGGGT TTTCA"ATGCG AGATCTAAAA ACATTCTCTC CCGTGGCACC GGTAGTAAGT "  
       AAGGATATTT CATCCTCCCA AA"AGTTACGC TCTAGATTTT TGTAAGAGAG GGCACCGTGG CCATCATTCA "  
  
069581 "ACTATATGGT CGGGCTTTAG CGGGT"CTATT AATCGAGATC AATCGTTTAT TCCCGGATGC GTGGGAATCC   
       "TGATATACCA GCCCGAAATC GC"CCAGATAA TTAGCTCTAG TTAGCAAATA AGGGCCTACG CACCCTTAGG   
  
069651 CCATTTTTTC CATCCTAGTA TTGACTGGAA GGGGGAAGAA GATTAGAAAC CAATAAAATA T"CCTTTGAAT "  
       GGTAAAAA"AG GTAGGATCAT AACTGACCTT CCCCCTTCTT CTAATCTTTG GTTATTTTAT AGGAAACTTA "  
  
069721 "AATCCCCTTC CCCCTTCTTT TTTTTAGAGT TTTTAGACTT AGAATAAGTA GAAAGAATCA A"AATAGATTC   
       "TTAGGGGA"AG GGGGAAGAAA AAAAATCTCA AAAATCTGAA TCTTATTCAT CTTTCTTAGT TTTATCTAAG   
  
069791 AACCTCAGTC AAACTCGAAC CCGGCGCCGG GTCAAATTGA ATTAATAAAA GAGGAGAACG AAA"ATGAAAA "  
       TTGGAGTCAG TTT"GAGCTTG GGCCGCGGCC CAGTTTAACT TAATTATTTT CTCCTCTTGC TTTTACTTTT "  
  
069861 "TGTGATGGCT AGGGCAACAA TATCATACAA AATACTTAAA TGAAAAACTG TACTGCGATT CTA"AATTTAG   
       "ACACTACCGA TCC"CGTTGTT ATAGTATGTT TTATGAATTT ACTTTTTGAC ATGACGCTAA GATTTAAATC   
  
069931 AAATCATTGT ATTACTATAT TTTAATATTT GATTGTAACG AAATCTTTCA TAATTTTATT TCGAGT"TACC "  
       TTTAGTAACA TAATGATATA AA"ATTATAAA CTAACATTGC TTTAGAAAGT ATTAAAATAA AGCTCAATGG "  
  
070001 "AATTTTTCTT TTTTTTCTTC ATTTTGGATC GGAAAATGGA AGAGTTGCGT GAATCAAAAA TCCAAG"GGAG   
       "TTAAAAAGAA AAAAAAGAAG TA"AAACCTAG CCTTTTACCT TCTCAACGCA CTTAGTTTTT AGGTTCCCTC   
  
070071 GTTC"ATGGCC AAGGGTAAAG ATGCCCGAGT AACAGTTATT TTGGAAGGAC TCGTGTGTCG AAACGGTGTT "  
       CAAGTACCGG TTCCCAT"TTC TACGGGCTCA TTGTCAATAA AACCTTCCTG AGCACACAGC TTTGCCACAA "  
  
070141 "AATA"AGCAAT CAATAGGGAT TTCCAGATAT ATTACTCAAA AGAATCGA"CA CAATACGCCT AGTCGATTGG "  
       "TTATTCGTTA GTTATCC"CTA AAGGTCTATA TAATGAGTTT TCTTAGCTGT GTTATGCGGA TCAGCTAACC   
  
070211 "AATTGAGAAA ATTCTGTCCC CGGTGTTACA AACATACGAT TCATGGGG"AG ATAAAGAAAT AGAT"CGAACC "  
       TTAACTCTTT TAAGACAGGG GCC"ACAATGT TTGTATGCTA AGTACCCCTC TATTTCTTTA TCTAGCTTGG "  
  
070281 "GATCGGCTGT GTGTCACCCT TTTCCAATCC AAGGAAGATG AAAAATTACA TATATTAGAC ATAT"TTTAGA   
       "CTAGCCGACA CACAGTGGGA AAA"GGTTAGG TTCCTTCTAC TTTTTAATGT ATATAATCTG TATAAAATCT   
  
070351 TTTAAATATA AACAAACCAA ATCCTATTTC GATCGGATCT AAAATGATTT AGAATTAAGA AATAGGATTT   
       AAATTTATAT TTGTTTGGTT TAG"GATAAAG CTAGCCTAGA TTTTACTAAA TCTTAATTCT TTATCCTAAA "  
  
070421 TCGGGATAAG GAATAAAAAA ACCATGGAT"A AATCCAAGCG ACTCTTTCTT AAATCCAAAC GATCTTTTCG "  
       "AGCCCTATTC CTTATTTTTT TGG"TACCTAT TTAGGTTCGC TGAGAAAGAA TTTAGGTTTG CTAGAAAAGC   
  
070491 "TAGGCGTTTG CCCCCGATTC CATCGGGGG"A TCGAATTGAT TATAGAAACA TGAGTTTAAT TAGT"CGATTT "  
       ATCCGCAA"AC GGGGGCTAAG GTAGCCCCCT AGCTTAACTA ATATCTTTGT ACTCAAATTA ATCAGCTAAA "  
  
070561 "ATTAGTGAAC AAGGAAAAAT ATTATCTAGA CGGGTAAATA GATTGACCTT AAAACAGCAA CGCT"TAATTA   
       "TAATCACT"TG TTCCTTTTTA TAATAGATCT GCCCATTTAT CTAACTGGAA TTTTGTCGTT GCGAATTAAT   
  
070631 CTATTGCTAT AAAACAAGCT CGTATTTTAT CTTTGTTA"CC TTTTCTTAAT AATGATAAAA ATGATAAACA "  
       GATAACGATA TTTTGTTCGA G"CATAAAATA GAAACAATGG AAAAGAATTA TTACTATTTT TACTATTTGT "  
  
070701 "ATTTGAAAGA AGCGAGTCGA CTCCTAGAAC TATTGGTC"TT AGAACCAGGA ATAAATAGGC TTAG"CTTACT "  
       "TAAACTTTCT TCGCTCAGCT G"AGGATCTTG ATAACCAGAA TCTTGGTCCT TATTTATCCG AATCGAATGA   
  
070771 "CTTTAATTGA ATTAAAATTC TAATCCAAAC TCAACCGCAG ATTGATGCTT TGTTCGAAAA ATCC"GAAAAT   
       GAAATTAACT TAATTTTAAG ATT"AGGTTTG AGTTGGCGTC TAACTACGAA ACAAGCTTTT TAGGCTTTTA "  
  
070841 CTAGATTCGA TTGTCGTAAG AAAAAGAAAG AATAGTGGAA GAAGAATAAA TCGTTTTTTT TTATTG"AACA "  
       "GATCTAAGCT AACAGCATTC TTT"TTCTTTC TTATCACCTT CTTCTTATTT AGCAAAAAAA AATAACTTGT   
  
070911 "TATTTGTTCA TTTTGACCAC TTTATGATAT TTTATCATAC TAATTTCTAC TCTACCTTCT CGGAGT"TCAT   
       ATAAACAAGT AAAACT"GGTG AAATACTATA AAATAGTATG ATTAAAGATG AGATGGAAGA GCCTCAAGTA "  
  
070981 TCTCCAGAGA ACTCCATTTT AAGCAATCCG CTGGATTCTT TCCAATCTTC GTATTTTATT TTATGA"TCTC "  
       "AGAGGTCTCT TGAGGT"AAAA TTCGTTAGGC GACCTAAGAA AGGTTAGAAG CATAAAATAA AATACTAGAG   
  
071051 "ATTGGAAATC ATATAAAGAC AATTTCTATT TAATATCGCG ATTTGGGCAA GTATTTTACG ATTAAG"AAGC   
       TAACCTT"TAG TATATTTCTG TTAAAGATAA ATTATAGCGC TAAACCCGTT CATAAAATGC TAATTCTTCG "  
  
071121 AACTGCCTCT TGTACAGATT GTGTATGAAT CTACTATAAC TGTAGTCTAC ATTATTAGAT CGAATT"ACTG "  
       "TTGACGG"AGA ACATGTCTAA CACATACTTA GATGATATTG ACATCAGATG TAATAATCTA GCTTAA"TGAC "  
  
071191 "CATTTATTCG AGCGATCCAC AACTGTCGAA AATTTCTTTT TTGCCTACCT CTATCCCGAT AAGCTG"AAGC   
       "GTAAATAAGC TCGCTAGGTG TTGACAGCTT TTAAAGAAAA AACGGATGGA GATAGGGCTA TTCGAC"TTCG   
  
071261 CAAAGCTCTT ATTTTCTGTT GAGTAATAGT TCGAGTAAGT CTTGAATGA"G CCCCTCGAAA GCTTGATGCA "  
       GTTTCGAGAA TAAAAGACAA CTCATTATCA AGCTCATTCA GAACTTA"CTC GGGGAGCTTT CGAACTACGT "  
  
071331 "AATAAACGAA TTTTTGTTCT ACGTCTCCGA GCTATATATC CTCGTTTAA"T TCTAGTCATT GAATAAATGA   
       "TTATTTGCTT AAAAACAAGA TGCAGAGGCT CGATATATAG GAGCAAA"TTA AGATCAGTAA CTTATTTACT   
  
071401 AACTTTGATG AATAACGAAT TGATTTCCTT TCTTTCAGTT ATTCCTTTCT CCTTTCCTAG TCTATTAAT"A "  
       TTGAAACTAC TTATTGCTTA ACTAAAGGAA AGAAAGTCAA TAAGGAAAGA GGAAAGGATC AGATAATTAT   
  
071471 "ACCAAACGTA TTTTTCCAAT GTATAAAATA AAAATTCCAA TGGCTTTTGG TACTATAACC TTCCCGACC"A   
       TGGTTTGCAT AAAAAGGTTA CATATTTTA"T TTTTAAGGTT ACCGAAAACC ATGATATTGG AAGGGCTGGT "  
  
071541 CGATTTCTTT TTTTGTTCTA ATCACCCCAA AATACGAAAT AGTATTTTGA CTAGGTATAA AAAACAAA"CA "  
       "GCTAAAGAAA AAAACAAGAT TAGTGGGGT"T TTATGCTTTA TCATAAAACT GATCCATATT TTTTGTTTGT   
  
071611 "TAGAGTAAAT AAATCAAAAT AGAAATGGAT AAAGAAATAG TGGGTTCCTT CGTTTCTATG GTTACTTC"TT   
       ATCTCATTTA TTTA"GTTTTA TCTTTACCTA TTTCTTTATC ACCCAAGGAA GCAAAGATAC CAATGAAGAA "  
  
071681 AAACGGTGAG GTCCTCTCTA TACACCGGAG CTCCTTCTTT TATTTCATCA ATGTTA"TTGT TAACTTGTAC "  
       "TTTGCCACTC CAGG"AGAGAT ATGTGGCCTC GAGGAAGAAA ATAAAGTAGT TACAATAACA ATTGAACATG   
  
071751 "AGGTCACCCT CTTTGGCTCT ACCCATGAAT TATCTAGTAA TCGGTCTTTC ACAACG"AGAT CTACCTATAC   
       TCCAGTGGGA GAAACCGAGA TGGGTACTT"A ATAGATCATT AGCCAGAAAG TGTTGCTCTA GATGGATATG "  
  
071821 AGTAACGGTA TTTAATTATG AAGATTAGTT GGGTAGCTGA CCCTCTTAG"T CCGTTCTTGG AAGAATAAGG "  
       "TCATTGCCAT AAATTAATAC TTCTAATCA"A CCCATCGACT GGGAGAATCA GGCAAGAACC TTCTTATTCC   
  
071891 "CCATAATCCT TCTGTTAAAT AGGATTTCCT CTGCTTAATG GATAAGCAT"T TGTTACCAAT GGGGAATTCT   
       GGTATTAGGA AGACAATTTA TCCTAA"AGGA GACGAATTAC CTATTCGTAA ACAATGGTTA CCCCTTAAGA "  
  
071961 TTATCATCTA AAATTGAAAA TTGAGATGAT TGGATTTGCA CCAATAGAAA CCATAAATT"T GATACACAAT "  
       "AATAGTAGAT TTTAACTTTT AACTCT"ACTA ACCTAAACGT GGTTATCTTT GGTATTTAAA CTATGTGTTA   
  
072031 "AAAAGGATAC GATAAATCTT TGTTTATTTA TTTTCGGTAG TGAACGGAGT TCTTCCATT"C ATTCTATCCT   
       TTTTCCTATG CTATTTAGAA ACAAATAAA"T AAAAGCCATC ACTTGCCTCA AGAAGGTAAG TAAGATAGGA "  
  
072101 AGTCACTGGT ACTTATCACT GATACGGGAA AAATTTTGTA CTTTCTTTTG TCCCGG"TCCA TGGTCTAAAC "  
       "TCAGTGACCA TGAATAGTGA CTATGCCCT"T TTTAAAACAT GAAAGAAAAC AGGGCCAGGT ACCAGATTTG   
  
072171 "GAGTCGCACA TACACCCTAG TACATGTTCC TCGACGCTGA GGGCATCCCC GAAGGG"CGGG CGATTTGGTG   
       CTCAGCGTGT ATGTGGGATC ATGTACA"AGG AGCTGCGACT CCCGTAGGGG CTTCCCGCCC GCTAAACCAC "  
  
072241 ACATTTCTGA TTGGCTGTCT TGTGTTTCTA ATAAGTTGTT TAATAGTTGG CATGTTG"AAT TGTATACATA "  
       "TGTAAAGACT AACCGACAGA ACACAAA"GAT TATTCAACAA ATTATCAACC GTACAAC"TTA ACATATGTAT "  
  
072311 "ATGAGTTGGT TTAGATCAAT CCTAACCGGA TGATTATGAA TTACTTCTAT ATTCGAT"ATT AATAATTAAT   
       "TACTCAACCA AATCTAGTTA GGATTGGCCT ACTAATACTT AATGAAGATA TAAGCTA"TAA TTATTAATTA   
  
072381 ATGATTAATA GTAATCCGGT AAAATTGTAA AATCTAAATT TCGGATTTTT GCGAGCAAT"A CTTATTAAAC "  
       TACTAATTAT CATTAGGCCA TTTTAACATT TTAGATTTAA AGCCTAAAAA CGCTCGTTAT GAATAATTT"G "  
  
072451 "CGCTACAAGA TCAACAATTC CATGAGCTTG GGCTTCTGGT GCTGACATAA AAACATCTC"T TTCCATATCT   
       "GCGATGTTCT AGTTGTTAAG GTACTCGAAC CCGAAGACCA CGACTGTATT TTTGTAGAGA AAGGTATAG"A   
  
072521 TCGGGTATAA CCCATAAGGG TTTGCCTGTT CTTTGGACAT AAACCTTTGT GATGGTTTC"G CGCAGGTTCA "  
       AGCCCATATT GGGTATTCCC AAACGGACAA GAAACCTGTA TTTGGAAACA CTACCAAAG"C GCGTCCAAGT "  
  
072591 "GTAGTTCGGT CGATTCCAGG ACAAATTCTC CCGTTTTTCC CTTAGAAAAA GAACTAGCA"G GTTGATGGAT   
       "CATCAAGCCA GCTAAGGTCC TGTTTAAGAG GGCAAAAAGG GAATCTTTTT CTTGATCGT"C CAACTACCTA   
  
072661 CATTACCCTG ATGATTTAAT AAATGGTTTT CTCTATCTTA CATCATTATC ATGATGAGTC AAAGATAAT"A "  
       GTAATGGGAC TACTAAATTA TTTACCAAAA GAGATAGAAT GTAGTAATAG TACTACTCAG TTTCTATTAT   
  
072731 "AAAAAAAGAT AGGATTAACA ACCGTACAGG CATCCTTTGT GCAGTGCATA CGGCTCCACA ATGGAATTC"A   
       TTTT"TTTCTA TCCTAATTGT TGGCATGTCC GTAGGAAACA CGTCACGTAT GCCGAGGTGT TACCTTAAGT "  
  
072801 TTTTTACCTT CCAGCGAAGG AATAGAAAAT AGAGGATCTA GCAGACCCAG A"GCAGTAAAT GATCCAATAA "  
       "AAAA"ATGGAA GGTCGCTTCC TTATCTTTTA TCTCCTAGAT CGTCTGGGTC TCGTCATTTA CTAG"GTTATT "  
  
072871 "CCACCCTTCC TTTTTTTAGA AATTTTTAAA ATACTAGGAT GGTTCCGTTG C"TTTATGATT TCGTTTGTTA   
       "GGTGGGAAGG AAAAAAATCT TTAAAAATTT TATGATCCTA CCAAGGCAAC GAAATACTAA AGCA"AACAAT   
  
072941 TTCAGCAATC CCAAAGTGTC TTTTTTTTTT TTTTTTTTCA AAAAAAAAAA "ACTCATTGGC GCCGAGCGTG "  
       AAGTCGTTAG GGTTTCACAG AAAAAAAAAA AAAAAAAAGT TTTTTTTTTT TGAGTAACCG CGGCTCGCAC   
  
073011 "AGGGAATGCT AGACGTTTGG TAATTTCTCC TCCGACTAGA ATAAAAGATC" CCATTGAAGC GGCTAATCCC   
       TCCCTTACGA TCTGCAAACC AT"TAAAGAGG AGGCTGATCT TATTTTCTAG GGTAACTTCG CCGATTAGGG "  
  
073081 ATACATATTG TATGGACATC TGGTGACACA AATTGCATAG TATCAAAAAG A"CCTACTCCG GGTATTACCG "  
       "TATGTATAAC ATACCTGTAG AC"CACTGTGT T"TAACGTATC ATAGTTTTTC TGGATGAGGC CCATAATGGC "  
  
073151 "ATCCCCCGGG AGAGTTTATA AATAAATACT GATCTTTGGT ACCATCCTCT A"TACTGAGAT ATACCATAAG   
       "TAGGGGGCCC TCTCAAATAT TTATTTATGA C"TAGAAACCA TGGTAGGAGA TATGACTCTA TATGGTATTC   
  
073221 ACCCATAATT TGATTCGAGA TCTCATTATT AACATCTTGG CCTAAAAAAA GTAATCTTTG TCGATAAA"GT "  
       TGGGTA"TTAA ACTAAGCTCT AGAGTAATAA TTGTAGAACC GGATTTTTTT CATTAGAAAC AGCTATTTCA "  
  
073291 "CGGTTGTATT AGGATAAAAT TGTATCCCGT AAGAAGCGAC GGGCACCTTT TGATGCATAC GGTTCAAA"AA   
       "GCCAAC"ATAA TCCTATTTT"A ACATAGGGCA TTCTTCGCTG CCCGTGGAAA ACTACGTATG CCAAGTTTTT "  
  
073361 AATAGCGAAA AAAAGAATCA ATGTTTAGAT TTTAGCCTTC TTTAGAGGAC TCTTTCTAA"C TTCTAGGAAG "  
       "TTATCGCTTT TTTTCTTAG"T TACAAATCTA AAATCGGAAG AAATCTCCTG AGAAAGATTG AAGATCCTTC   
  
073431 "GGGCTTTTTC TTACCTTCCA TTTTTTTTTC AAACTGGATA AGTTTTGTCC TTTGGCCCG"C GGTCGTTTAT   
       CCCGAAAAAG AATGGAAGGT AAAAA"AAAAG TTTGACCTAT TCAAAACAGG AAACCGGGCG CCAGCAAATA "  
  
073501 CGATACTATA AATTTCAATA AATAAAAAAC CAATTCATTT TCATTAAATT TTCAATTTAT "CGAACTAACT "  
       "GCTATGATAT TTAAAGTTAT TTATT"TTTTG GTTAAGTAAA AGTAATTTAA AAGTTAAATA GCTTGATTGA   
  
073571 "TTTCATTGAT GTATTGTTTT GTTTCATCGA GATTCAATTT AAATTGCGAT GTCATTTTCT" TGTTCCAAAA   
       AAAGTA"ACTA CATAACAAAA CAAAGTAGCT CTAAGTTAAA TTTAACGCTA CAGTAAAAGA ACAAGGTTTT "  
  
073641 CGGTCTTCTT CAATTCTTTT AGGTTTATGC TCTACTCCGA GTCAAGATCT "GTCCGATTTG GATTTGCACA "  
       "GCCAGA"AGAA GTTAAGAAAA TCCAAATACG AGATGAGGCT CAGTTCTAGA CAGGCTAAAC CTAAACGTGT   
  
073711 "TATAGGACAA ATGTCCCAAT AGCATGGCTT TTTGCTACGA CTTCTTTTTT" TTTTCAATTT ACTTCATATT   
       ATATCC"TGTT TACAGGGTTA TCGTACCGAA AAACGATGCT GAAGAAAAAA AAAAGTTAAA TGAAGTATAA "  
  
073781 ACTCGATTGA TTAAAAGTTT TCTATCAATG CTATTTAAAA ATCGAATATA TTAGTA"ATCA TAGAATAGAT "  
       "TGAGCT"AACT AATTTTCAAA AGATAGTTAC GATAAATTTT TAGCTTATAT AATCATTAGT ATCTTATCTA   
  
073851 "AGATTCCAAA TTTAGTTTTT TCTAAGCGGA GCCTGGATAC TTCATTTTAT TAGTAC"AACC GAGAAAACCA   
       TCTAAGGTTT AAATCAAAAA A"GATTCGCCT CGGACCTATG AAGTAAAATA ATCATGTTGG CTCTTTTGGT "  
  
073921 TAAATTATTC TAATTGATAA TATCAATCAG GATACCCCCC CAAAAAAATA GATCTAATTG CA"CTTCACGC "  
       "ATTTAATAAG ATTAACTATT A"TAGTTAGTC CTATGGGGGG GTTTTTTTAT CTAGATTAAC GTGAAGTGCG   
  
073991 "TCCAAATTTT TGATGATGAA ATCAATCCGT CTTGGGCGAA AGAAAGGATA TCTCGATCGG GG"GAGAGAAC   
       AGGTTTAAAA ACTACTACTT "TAGTTAGGCA GAACCCGCTT TCTTTCCTAT AGAGCTAGCC CCCTCTCTTG "  
  
074061 GGGGAAATAC CATATGACCC AATATATCTG ACAAGTCGCA CTATAGGTCA ACCCACGAT"G CATCTTCTTC "  
       "CCCCTTTATG GTATACTGGG" TTATATAGAC TGTTCAGCGT GA"TATCCAGT TGGGTGCTAC GTAGAAGAAG "  
  
074131 "TCCGGGAAGT CGAAAGGGTA CTTTTGGAAC ACCAACAGGC ATTAAAGCAA AGCAAAACG"A AGTAAGTAGT   
       "AGGCCCTTCA GCTTTCCCAT GAAAACCTTG TGGTTGTCCG TA"ATTTCGTT TCGTTTTGCT TCATTCATCA   
  
074201 ATAGCTAACG TTAATGTGGA AGCGTAACAA CGGGTTTATT GTCTTAATAA "ATAAGATGGG CCTTTATCAG "  
       T"ATCGATTGC AATTACACCT TCGCATTGTT GCCCAAATAA CAGAATTATT TATTCTACCC GGAAATAGTC "  
  
074271 "ATTTTATCTT TTAACATATT TATACATAGA TTTAATAATA ATAAGTTCAT" AAAAAAGGAA AACAGAATTA   
       "T"AAAATAGAA AATTGTATAA ATATGTATCT AAATTATTAT TATTCAAGTA TTTTTTCCTT TTGTCTT"AAT "  
  
074341 AATTAATAAA AGAAACTTCT TCCCGAATAG GTTTTTGAAT GATGAACAA"G TATGTATACA TTCATTCATA "  
       "TTAATTATTT TCTTTGAAGA AGGGCTTATC CAAAAACTTA CTACTTGTTC ATACATATGT AAGTAAG"TAT   
  
074411 "TAAAATATGA TCAATTCCCA TCTACCATTG CGTATTGGTA CTTATCGAG"T ATAGAATAGA TCTGCTTCTT   
       ATTTTATACT AGTTAAGGGT AGATGGTAAC GCATAACCAT GAATAGCTCA TAT"CTTATCT AGACGAAGAA "  
  
074481 TTTGTTCCTA CGAATAGAAT CGAATTGTTC CATTATTACT AAAAGAATAG ACTAAATATT AAT"CCTTTGG "  
       "AAACAAGGAT GCTTATCTTA GCTTAACAAG GTAATAATGA TTTTCTTATC TGA"TTTATAA TTAGGAAACC   
  
074551 "GCAAGAAAAC CCCCTCAAAG GGTGAGGTCC GTAGCATAGT TTTTTTTTCA GTGCAATAAA GTT"ACATAGT   
       CGTTCTTTTG GGGGAGTTTC CCACTCCAGG CATCGTATCA AAAAAAAAGT CACGTTATTT CAATGT"ATCA "  
  
074621 GTCTATTTTT CCTTGATAAA GGGGTATTTC CATGGGTTTG CCTTGGTATC GTGTTCATAC CGTTGTATTG   
       "CAGATAAAAA GGAACTATTT CCCCATAAAG GTACCCAAAC GGAACCATAG CACAAGTATG GCAACA"TAAC   
  
074691 AATGATCCCG GTCGTTTGCT TTCTGTTCAT ATAATGCAAA CAGCTCTAGT TGCTGGTTGG GCCGGTTCAA   
       TTACTAGGGC CAGCAAACGA AAGACAAGTA TATTACGTTT GTCGAGATCA AC"GACCAACC CGGCCAAGTT "  
  
074761 TGGCCCTATA CGAATTAGCA GTTTTTGAT"C CCTCTGATCC TGTTCTTGAT CCAATGTGGA GACAAGGTAT "  
       "ACCGGGATAT GCTTAATCGT CAAAAACTAG GGAGACTAGG ACAAGAACTA GG"TTACACCT CTGTTCCATA   
  
074831 "GTTCGTTATA CCCTCCATGA CTCGTTTAG"G AATAACCAAT TCATGGGGAG GTTGGAGTAT CACAGGAGGG   
       CAAGCAATAT GGGAGGTACT GAGCAAATCC TTATTGGTTA AGTACCCCTC CAA"CCTCATA GTGTCCTCCC "  
  
074901 ATTATAACGA ATCCGGGTAT TTGGAGTTAC GAAGGTGTGG CCGGAGCACA TATTTTGTTT TCTGGATTGT   
       "TAATATTGCT TAGGCCCATA AACCTCAATG CTTCCACACC GGCCTCGTGT ATA"AAACAAA AGACCTAACA   
  
074971 GCTTCTTAGC AGCTATCTGG CATTGGGTGT ATTGGGATCT AGAAATATTT TCTGATGAAC GTACAGGAAA   
       CGAAGAATCG TCGATAGACC GTAACCCACA TAACCCTAGA TCTTTATAAA AGACT"ACTTG CATGTCCTTT "  
  
075041 ACCTTCTTTG GATTTGCCGA AGATTTTTGG AATTCATTTA TTT"CTCTCAG GGTTGGGTTG TTTTGGTTTT "  
       "TGGAAGAAAC CTAAACGGCT TCTAAAAACC TTAAGTAAAT AAAGAGAGTC CCAAC"CCAAC AAAACCAAAA   
  
075111 "GGTGCATTTC ATGTAACCGG ATTGTACGGT CCGGGAATAT GGG"TATCCGA TCCTTATGGA TTAACCGGAA   
       CCACGTAAAG TACATTGGCC TAACATGCCA GGCCCTTATA CCCATAGGCT AGGAATACCT AATTGGCCTT   
  
075181 GGGTACAAGC CGTAAATCCT GCGTGGGGTG TCGAAGGGTT TGATCCTTTT GTTCCGGGCG GAATAGCCTC   
       "CCCATGTTCG GCATTTAGGA CGCACCCCAC AGCTTCCCAA ACTAGGAAAA CAAGGCCCGC CTTATCGGAG"   
  
075251 GCATCATATT GCAGCAGGTA CATTGGGTAT ATTAGCGGGC CTATTCCATC TTAGTGTTCG TCCGCCCCAA   
       CGTAGTATAA CGTCGTCCAT GTAACCCATA TAATCGCCCG GATAAGGTAG AATCACAAGC A"GGCGGGGTT "  
  
075321 CGTCTATACA AAGGATTACG TATGGGAAAT ATTGAAACCG TCCTTTCGAG TAGTATCGCT GCTGTATTTT   
       "GCAGATATGT TTCCTAATGC ATACCCTTTA TAACTTTGGC AGGAAAGCTC ATCATAGCGA C"GACATAAAA   
  
075391 TTGCAGCTTT TGTTGTTGCT GGAACTATGT GGTATGGTTC AGCAACTACC CCGATTGAAT TATTTGGGCC   
       AACGTCGAAA ACAACAACGA CCTTGATACA CCATACCAAG TCGTTGATGG GGCTAACTTA ATAAACCCGG   
  
075461 C"ACTCGTTAT CAATGGGATC AGGGATACTT CCAGCAAGAA ATATATCGAA GAGTTAGTAC CGGGTTAGCC "  
       G"TGAGCAATA GTTACCCTAG TCCCTATGAA GGTCGTTCTT TATATAGCTT CTCAATCATG GCCCAATCGG "  
  
075531 "G"AAAATCAAA GTTTATCAGA AGCTTGGTCT AAAATTCCTG AAAAATTAGC TTTTTATGAT TATATCGGGA   
       "C"TTTTAGTTT CAAATAGTCT TCGAACCAGA TTTTAAGGAC TTTTTAATCG AAAAATACTA ATATAGCCCT   
  
075601 ATAATCCCGC AAAAGGTGGA TTATTCAGAG CGGGTTCCAT GGACAACGGG GATGGAATAG CTGTTGGTTG   
       T"ATTAGGGCG TTTTCCACCT AATAAGTCTC GCCCAAGGTA CCTGTTGCCC CTACCTTATC GACAACCAAC "  
  
075671 GTTAGGACAC CCTGTTTTTA GAGATAAAGA AGGGCGCGAA CTTTTTGTAC GCCGTATGCC GACCTTTTTT   
       "C"AATCCTGTG GGACAAAAAT CTCTATTTCT TCCCGCGCTT GAAAAACATG CGGCAT"ACGG CTGGAAAAAA "  
  
075741 GAAACATTTC CGGTTGTTTT GGTAGACGGA GATGGAATTG TTAGAGCCGA TGTTCCTTTT CGAAGAGCAG   
       "CTTTGTAAAG GCCAACAAAA CCATCTGCCT CTACCTTAAC AATCTCGGCT ACAAGG"AAAA GCTTCTCGTC   
  
075811 AATCGAAG"TA TAGTGTTGAA CAGGTCGGTG TAACTGTTGA GTTCTATGGC GGTGAACTCA ATGGAGTCAG "  
       TTAGCTTCAT ATCACAACTT GTCCAGCCAC ATTGACAACT CAAGATACCG C"CACTTGAGT TACCTCAGTC "  
  
075881 "TTATAGTG"AT CCTGCTACTG TGAAAAAATA TGCTAGACGT GCTCAATTGG GTGAAATTTT TGAATTAGAT   
       "AATATCACTA GGACGATGAC ACTTTTTTAT ACGATCTGCA CGAGTTAACC C"ACTTTAAAA ACTTAATCTA   
  
075951 CGTGCTACTT TGAAATCCGA TGGGGTTTTT CGTAGCAGTC CAAGGGGTTG GTTTACTTTT GGGCATGCTT   
       GCACGATGAA ACTTTAGGCT ACCCCAAAAA GCATCGTCAG GTTCCCCAAC CAAATGAAAA C"CCGTACGAA "  
  
076021 CCTTTGCTTT GCTCTTCTTC TTCGGACACA TTTGGCATGG TGCGAGAACG TTGTTCAGAG ATGTCTTTGC   
       "GGAAACGAAA CGAGAAGAAG AAGCCTGTGT AAACCGTACC ACGCTCTTGC AACAAGTCTC T"ACAGAAACG   
  
076091 TGGGATTGAC CCAGATTTGG ACACTCAAGT AGAATTTGGG GCATTCCAGA AAACTTGGAG ATCCAACTAC   
       ACCCTAACTG GGTCTAAACC TGTGAGTTCA TCTTAAACCC CGTAAGGTCT T"TTGAACCTC TAGGTTGATG "  
  
076161 AAAAAGAGTC TAGTCTGATA CAAGATTGCT CAGTTTTGTT TTTTCTTTAT TTTTT"GATTT GACATAGATA "  
       "TTTTTCTCAG ATCAGACTAT GTTCTAACGA GTCAAAACAA AAAAGAAATA A"AAAACTAAA CTGTATCTAT   
  
076231 "GGGTACCGGA TAACTCTTTA TTCGGATTAC TTACTTTTCA TTTCTTTGAC TCTTG"TCTTG GTCTTTTTTT   
       CCCATGGCCT ATTGAGAAAT AAGCCTAATG AATGAAAAGT AAAGAAACTG AGAACAGA"AC CAGAAAAAAA "  
  
076301 TATCCTTAAA AAGATCCCAA CTAAACAGGT ATGGAAGCTA TAATTGTAAA CCGAAATCAA ATCTATGGAA   
       "ATAGGAATTT TTCTAGGGTT GATTTGTCCA TACCTTCGAT ATTAACATTT GGCTTTAG"TT TAGATACCTT   
  
076371 GCATTGGTTT ATACATTCCT CTTA"GTCTCG ACTCTAGGAA TAATTTTTTT CGCTATCTTT TTTCGCGAAC "  
       CGTAACCAAA TATGTAAGGA GAATCAGAGC TGAGATCCTT ATTAAAAAAA GCGATAGAAA AAAGCGCTTG   
  
076441 "CGCCTAAAGT TCCAGTTCCA ACTA"AAAAGG CGAAATGATT TCTCATTATT TCAATTGAAG TAATGAGCCT   
       G"CGGATTTCA AGGTCAAGGT TGATTTTTCC GCTTTACTAA AGAGTAATAA AGTTAACTTC ATTACTCGGA "  
  
076511 CACAATATT"G TGAGGCTCAT TACTTCAACT AGTCCCCGTG TTCCTCGAAT GGATCTCTTA GTTGTTGAGA "  
       "G"TGTTATAAC ACTCCGAGTA ATGAAGTTGA TCAGGGGC"AC AAGGAGCTTA CCTAGAGAAT CAACAACTCT "  
  
076581 "GGGTTGCCC"A AAAGCAGTAT ATAAGGCATA CCCAGTAAAA CTTACAAGTA AACCAGATAT AGAGA"TGGCA "  
       "CCCAACGGGT TTTCGTCATA TATTCCGTAT GGGTCATT"TT GAATGTTCAT TTGGTCTATA TCTCTACCGT   
  
076651 "ACTAGGGTTG CTGTTTCCAT TATTATATAA ATTAAAGACC ACAATGGATC TATAATAAGA TCCTT"TATTT   
       TGATCCCAAC GACAAAGGTA ATAATATATT TAATTTC"TGG TGTTACCTAG ATATTATTCT AGGAAATAAA "  
  
076721 ACAACGGAAT GGTATACAAA GTCAACAGAT CTCACTGAAT AAAAAAAAAT ATAGGATTAT TTATGGCTAC   
       "TGTTGCCTTA CCATATGTTT CAGTTGTCTA GAGTGAC"TTA TTTTTTTTTA TATCCTAATA AATACCGATG   
  
076791 ACAAACCGTT "GACGATAGTT CTAAATCTGG GCCAAGACGA ACTGTTGTAG GGGATTTATT GAAACCATTG "  
       TGTTTGGCAA CTGCTATCAA GATTTAGACC CGGTTCTGCT T"GACAACATC CCCTAAATAA CTTTGGTAAC "  
  
076861 "AATTCGGAAT" ATGGTAAAGT GGCTCCTGGG TGGGGAA"CTA CACCTTTAAT GGGTGTCGCG ATGAGTTTAT "  
       "TTAAGCCTTA TACCATTTCA CCGAGGACCC ACCCCTTGAT G"TGGAAATTA CCCACAGCGC TACTCAAATA   
  
076931 "TTGCGATATT CCTATGTATT ATTTTGGAGA TTTATAA"TTC TTCCATTTTA CTGGACGGAA TTTCAAGCAA   
       AACGCTATAA GGATACATAA TAAAACCTCT AAATA"TTAAG AAGGTAAAAT GACCTGCCTT AAAGTTCGTT "  
  
077001 TTAGATTC"GA TTCACAAGAA TCCCTAAATA ACTTTTCACT AAAAAGGAAA GTCTAAAGTA TGTAGGTTTC "  
       "AATCTAAGCT AAGTGTTCTT AGGGATTTAT TGAAA"AGTGA TTTTTCCTTT CAGATTTCAT ACATCCAAAG   
  
077071 "TGCTTTTT"AG CCTACTCTTT TTTGGTAGTT CGATCGTGGA ATTTATTTTT TCTGTATTTC CGGAATATGA   
       ACGAAAAATC GGATGAGAAA AAACCATCAA GCTAGCACCT TAAATAAAAA AGA"CATAAAG GCCTTATACT "  
  
077141 GT"GTGTGACT TGTTAGAATT GATCCTATGG ATACGACAGA GAAGAAGTCG GTCATCTTGA TCAAGATGAT "  
       "CACACACTGA ACAATCTTAA CTAGGATACC TATGCTGTCT CTTCTTCAGC CAG"TAGAACT AGTTCTACTA   
  
077211 "TT"TATCTCGT TGGATATTCA GTCTAGGCTA GTATCTGTAG CACAGAATAC ATGAAATAGA TTCCAAAATA   
       AAATAGAGCA ACCTATAAGT CAGATCCGAT CATAGACATC GTGTCTTATG TACTT"TATCT AAGGTTTTAT "  
  
077281 TTA"GAACAAA TATTAGAACT ATGATTCATA CTTATCAGAC CTCGTGGCCG GACTCCGAAA AAAGAGTTAG "  
       "AATCTTGTTT ATAATCTTGA TACTAAGTAT GAATAGTCTG GAGCACCGGC CTGAG"GCTTT TTTCTCAATC   
  
077351 "AAG"TGATAAA TTCAAAAATT GATTCTTTCG TTTCTACTTA TTTTGGTTAA AGGATAAACC TTTCTTTGGC   
       TTCACTATTT AAGTTTTTAA CTAAGAAAGC AAAGATGAAT AAAACCAATT TCCTA"TTTGG AAAGAAACCG "  
  
077421 TTTTTTCATT ATATTAAGTC AT"TGAATAAG CATTGAATAA GTGATAATCC AAGGGTTCTT ACTCAGGGAA "  
       "AAAAAAGTAA TATAATTCAG TAACTTATTC GTAACTTATT CACTATTAGG TTCCC"AAGAA TGAGTCCCTT   
  
077491 "TTTTTGAACT TTTTTGGAAG GT"TTTTTATT GAATCATCGT GGTTCTAGTC TGAATCTAAG GTTTTAATTG   
       AAAAACTTGA AAAAACCTTC CAAAAAATAA CTTAG"TAGCA CCAAGATCAG ACTTAGATTC CAAAATTAAC "  
  
077561 ATTCATAGA"G TCTTAACAAG ATAATTCCTA TCAATAATAA AGAAAACAAG AGTAAAGTCG CCTTACATCC "  
       "TAAGTATCTC AGAATTGTTC TATTAAGGAT AGTTA"TTATT TCTTTTGTTC TCATTTCAGC GGAATGTAGG   
  
077631 "AAATAAAAG"A AAAAAAATAG GTAAATAGAA GATTCAAGAG GCCCCTAATG ATCAATATAA ATACGAATGA   
       TTTATTTTCT TTTTTTTATC CATTTATCTT CTAAGTTCTC "CGGGGATTAC TAGTTATATT TATGCTTACT "  
  
077701 GCCGACTTGA T"ATTTGGGCA TTATAACCAC AAAGAAGACT TTTCGGATTT TGATTCTTTC GTATCTTCAT "  
       "CGGCTGAACT ATAAACCCGT AATATTGGTG TTTCTTCTGA" AAAGCCTAAA ACTAAGAAAG CATAGAAGTA   
  
077771 "ACAAAAAATG G"AATCATAGC ATTAAGAAGT TTTAAACTTT TTATGTACAC ATATCCGTTA CGAGCGGTAT   
       TGTTTTTTAC CTTAGTATCG TAATTCTTCA AAATT"TGAAA AATACATGTG TATAGGCAAT GCTCGCCATA "  
  
077841 ATTTGG"GTGT TTCTGTTTGA GCCGTACGAG ACGAAATTCT CATATACGGT TCTCAGAGGG GGGTCCCCTT "  
       "TAAACCCACA AAGACAAACT CGGCATGCTC TGCTT"TAAGA GTATATGCCA AGAGTCTCCC CCCAGGGGAA   
  
077911 "GGTTTA"CCTA TCTCAATAAA GTGTATGATT GG"TTCGAAGA ACGTCTTGAA ATTCAGGCGA TTGCGGATGA "  
       CCAAATGGAT AGAGTTATTT CACATACTAA CCAAGCTTCT TGCAGAACTT TAAG"TCCGCT AACGCCTACT "  
  
077981 "TATAACTAGT AAATACGTTC CTCCTCATGT CA"ACATATTT TATTGTCTAG GAGGAATTAC GCTTACTTGT   
       "ATATTGATCA TTTATGCAAG GAGGAGTACA GTTGTATAAA ATAACAGATC CTCC"TTAATG CGAATGAACA   
  
078051 TTTTTAGTAC AAGTAGCTAC CGGGTTTGCT ATGACTTTTT ACTACCGTCC GACCGTTACT GAGGCTTTTG   
       AAAAATCATG TTCATCGATG GCCCAAACGA TACTGAAAAA TGATGGCAGG CT"GGCAATGA CTCCGAAAAC "  
  
078121 CTTCTGTTCA ATATATAATG ACGGAAGCAA ATTTTGGTTG GTTAATCC"GA TCAGTTCATC GATGGTCGGC "  
       "GAAGACAAGT TATATATTAC TGCCTTCGTT TAAAACCAAC CAATTAGGCT AG"TCAAGTAG CTACCAGCCG   
  
078191 "AAGTATGATG GTCTTAATGA TGATCCTGCA CGTATTTCGC GTGTATCT"TA CTGGTGGATT TAAAA"AACCT "  
       TTCATACTAC CAGAATTACT ACTAGGACGT GCATAA"AGCG CACATAGAAT GACCACCTAA ATTTTTTGGA "  
  
078261 "CGTGAATTGA CTTGGGTTAC AGGTGTGGTT CTGGCTGTAT TGACCGCATC CTTTGGTGTA ACTGG"TTATT   
       "GCACTTAACT GAACCCAATG TCCACACCAA GACCGA"CATA ACTGGCGTAG GAAACCACAT TGACCAATAA   
  
078331 CCTTACCTCG GGACCAAATT GGTTATTGGG CAGTCAAAAT TGTAACAGGT GTACCGGAAG CTATTCCAGT   
       GGAATGGAGC CCTGGTTTAA CCAATAACCC GTCAGTTTTA ACAT"TGTCCA CATGGCCTTC GATAAGGTCA "  
  
078401 AATAGGG"TCG CCTTTGGTAG AGTTATTACG CGGAAGTGCT AGTGTGGGAC AATCTACCTT GACTCGTTTT "  
       "TTATCCCAGC GGAAACCATC TCAATAATGC GCCTTCACGA TCAC"ACCCTG TTAGATGGAA CTGAGCAAAA   
  
078471 "TATAGTT"TAC ACACTTTTGT ATTACCTCTT CTTACTGCCG TATTTATGTT AATGCACTTC CCAATGATAC   
       ATATCAAATG TGTGAAAACA TAATGGAGAA GAATGACGGC AT"AAATACAA TTACGTGAAG GGTTACTATG "  
  
078541 GTAAGCAAGG CATTTCTGGT CCTTTATAGA GAATAGAAA"T TAGAAATCAT AGATTTGTAA TTAGTTATTT "  
       "CATTCGTTCC GTAAAGACCA GGAAATATCT CTTATCTTTA AT"CTTTAGTA TCTAAACATT AATCAATAAA   
  
078611 "ATGATTACTC GGGGGGGGAA GAAGAGTATT TCATTGCTG"C AAATATGGAT TATTGAAAAA TAAGACATGT   
       TACTAATGAG CCCCCCCCTT CTTCTCATAA AGTAACGACG TTTA"TACCTA ATAACTTTTT ATTCTGTACA "  
  
078681 ATTTGGATAT TTCCCTTCAA CTCCACGAAA TTTTATTCGG TTATTTTTCA ATAATAAATA ATAGTTGAAG   
       "TAAACCTATA AAGGGAAGTT GAGGTGCTTT AAAATAAGCC AATA"AAAAGT TATTATTTAT TATCAACTTC   
  
078751 GGAATTCTTC GAAGAGAAAA TGGATTATGG GAGTGTGTGA CTTGAACTAT TGATTGGGCC GTGAAGATAT   
       CCTTAAGAAG CTTCTCTTTT ACCTAATACC CTCACACACT GAACT"TGATA ACTAACCCGG CACTTCTATA "  
  
078821 ATGTCTTTTT ATCTG"CCACA TTGGGATTCA CAACCAAATG TGTCTTTATT CCAACCACCG CGAAAGCTCC "  
       "TACAGAAAAA TAGACGGTGT AACCCTAAGT GTTGGTTTAC ACAGA"AATAA GGTTGGTGGC GCTTTCGAGG   
  
078891 "ATACAGAGGG TAGGC"TGGTT CGCTTGAAGA GAATCTTTTC CATGATCAGA CTTGACCATG TCATG"CATGA "  
       TATGTCTCCC ATCCGACCAA GCGAACTTCT CTTAGAAAAG GTACTA"GTCT GAACTGGTAC AGTACGTACT "  
  
078961 "CCAGGCTCCG TAAGATCCAG TAGAATAGGT GATATATATG GCATAATACA GATTATGTTT TATCT"ATTTC   
       "GGTCCGAGGC ATTCTAGGTC ATCTTATCCA CTATATATAC CGTATT"ATGT CTAATACAAA ATAGATAAAG   
  
079031 ACTTACTTAA TAGTATGAAA ATGCATTCAT TTCCTCTGCA TTGACTCGAT TTATAATACT ATCGGAGTGA   
       TGAATGAATT ATCATACTTT TACGTAAGTA AAGGAG"ACGT AACTGAGCTA AATATTATGA TAGCCTCACT "  
  
079101 AACAAGGGAT CTAAA"GAAGA ACATAGGCTA GACTCTATTA GTAACAAGTA AATCCTTTGG ATGTAAAAAG "  
       "TTGTTCCCTA GATTTCTTCT TGTATCCGAT CTGAGA"TAAT CATTGTTCAT TTAGGAAACC TACATTTTTC   
  
079171 "TATCAATATT TTTGG"GGGCT AAAGCCCCAT CGCAAGGTTT GAGACGACCC AGAAAGCACT TGAACAT"TAT "  
       ATAGTTATAA AAACCCCCGA TTTCGGGGTA GCGTT"CCAAA CTCTGCTGGG TCTTTCGTGA ACTTGTAATA "  
  
079241 "CAACTTTGTA AGCCTACTTG GGTATTGAGC ATTTATCTGT AAGAACTGAA TTCCTTGCAA TGGATAA"TTG   
       "GTTGAAACAT TCGGATGAAC CCATAACTCG TAAAT"AGACA TTCTTGACTT AAGGAACGTT ACCTATTAAC   
  
079311 TAACTCTTGA AAACGGAATC TGCTAAATTT TTTCTTGATC ATCTAGAGTC ACTCGTTTAT TTTCTATATT   
       ATTGAGAACT TTTGCCTTAG ACGATTTAAA AAAGAACTAG TA"GATCTCAG TGAGCAAATA AAAGATATAA "  
  
079381 CTTG"GATAAG ATATCGATTT TTTTTCTGGA TCCAGTTGGT TCGTTTGATT TGTGCTCGAG CCGGATGATG "  
       "GAACCTATTC TATAGCTAAA AAAAAGACCT AGGTCAACCA AG"CAAACTAA ACACGAGCTC GGCCTACTAC   
  
079451 "AAAA"ATTATC ATGTCCGGTT CCTTCGGGGG ATGGATCCAT AATAATTCAC CTATCCTAAT AACAAAAAAA   
       TTTTTAATAG TACAGGCCAA GGAAGCCCCC TACCTAGGTA TTATTAAGTG "GATAGGATTA TTGTTTTTTT "  
  
079521 CCAGATTTGA "ATGATCCTGT ATTAAGAGCT AAATTGGCTA AGGGTATGGG TCATAATTAT TACGGAGAAC "  
       "GGTCTAAACT TACTAGGACA TAATTCTCGA TTTAACCGAT TCCCATACCC" AGTATTAATA ATGCCTCTTG   
  
079591 "CCGCATGGCC" CAATGATCTT TTATATATTT TTCCAGTAGT C"ATTCTAGGT ACTATTGCAT GTAACGTGGG "  
       GGCGTACCGG GTTACTAGAA AATATATAAA AAGGTCATCA G"TAAGATCCA TGATAACGTA CATTGCACCC "  
  
079661 "CTTAGCGGTT CTAGAACCAT CAATGATTGG TGAACCCGCA G"ATCCATTTG CAACTCCTTT GGAAATATTG   
       "GAATCGCCAA GATCTTGGTA GTTACTAACC ACTTGGGCGT C"TAGGTAAAC GTTGAGGAAA CCTTTATAAC   
  
079731 CCGGAATGGT ATTTCTTTCC CGTATTTCAA ATACTT"CGTA CAGTGCCAAA TAAGTTATTG GGTGTTCTTT "  
       GGCCTTACCA TAAAGAAAGG GCATAAAGTT TATGAA"GCAT GTCACGGTTT ATTCAATAAC CCACAAGAAA "  
  
079801 "TAATGGTTTC CGTACCGGCG GGATTATTAA CTGTAC"CTTT TTTGGAGAAT GTCAATAAAT TCCAAAATCC   
       "ATTACCAAAG GCATGGCCGC CCTAATAATT GACATG"GAAA AAACCTCTTA CAGTTATTTA AGGTTTTAGG   
  
079871 ATT"TCGTCGT CCAGTAGCGA CAACCGTCTT TTTGATTGGT ACCGCAGTAG CCCTTTGGTT GGGTATTGGA "  
       TAAAGCAGCA GGTCATCGCT GTTGGCAGAA AAACTAACCA T"GGCGTCATC GGGAAACCAA CCCATAACCT "  
  
079941 "GCA"ACATTAC CGATTGATAA ATCCCTAACT TTAGGTCTTT TTTAAATTGA TTCAATTGTG AAATAAATAA   
       "CGTTGTAATG GCTAACTATT TAGGGATTGA AATCCAGAAA A"AATTTAACT AAGTTAACAC TTTATTTATT   
  
080011 AATATCACGA TGTGTGTATC TA"GGGAATAG TTGCTTCAAA GTGAATTACC CCTAGATACA CATATTTAAT "  
       TTATAGTGCT ACACACATAG ATCCCTTATC AACGA"AGTTT CACTTAATGG GGATCTATGT GTATAAATTA "  
  
080081 "GAAATTTTTG ATTTATTTTG AG"TATACGGA TTCTGTTAAA GATTCAAACT TCATTTCGTC TTTTTTTTAT   
       "CTTTAAAAAC TAAATAAAAC TCATATGCCT AAGAC"AATTT CTAAGTTTGA AGTAAAGCAG AAAAAAAATA   
  
080151 AAAGTTTTTC TACAACAAAA ATCCAA"TGGA TTTCTAATTT ATGCAAAATT CTTTTCCATT TCGAGAATGT "  
       TTTCAAAAAG ATGTTGTTTT TAGGTTACCT AAAGATTAAA TACGTTTTAA GAAAAGGTAA AGCTCTTACA   
  
080221 "CTAATATATG TTTTACGTCT TCTATG"CGAA AATGTTTGAT TTTCATAAGT TCGTCTGGAC TATTATTCAA   
       GATTATATAC AAAATGCAGA AGATACGCTT TTACAAACTA AAAGTATTCA AGCAGACCTG ATAATAAGTT   
  
080291 AAGGTCAAAT AATGTATGTA TATTGC"ACCT TTTGAGGCAA TTATAGATCC TAGGAGGCAA TTCTGATTGG "  
       TTCCAGTTTA TTACATACAT ATAACG"TGGA AAACTCCGTT AATATCTAGG ATCCTCCGTT AAGACTAACC "  
  
080361 "TCAATAAAAA TATATTTTAA TGCTAT"TTCT TTTTTCTTTT TCCTTAGTTT CGCCAATTTC TCATGAAAAG   
       "AGTTATTTTT ATATAAAATT ACGATA"AAGA AAAAAGAAAA AGGAATCAAA GCGGTTAAAG AGTACTTTTC   
  
080431 TAAAAAGGGG TAAAGTAACT TTTTTTTTAT TATTTTTTAA "ATGGAAGTTT TCTTTTTCTT CTTCCGCATG "  
       ATTTTTCCCC ATTTCATTGA AAAAAAAATA ATAAAAAATT TACCTTCAAA AGAAAAAGAA GAAGGCGTAC   
  
080501 "TAAAAAAGGA ATAAATAAAT CAATCAAATT CCGAGAGGCT" TCATGAAGTG CTTCTTTAGG AGTTAAACTT   
       ATTTTTTCCT TATTTATTTA GTTAGTTTAA "GGCTCTCCGA AGTACTTCAC GAAGAAATCC TCAATTTGAA "  
  
080571 CCATTGGTCC ATATTTCGAG AAAAAGTATC TCTTGTTTTT "CATTCCCATT TACATAAGAA TGTATACTAT "  
       "GGTAACCAGG TATAAAGCTC TTTTTCATAG" AGAACAAAAA GTAAGGGTAA ATGTATTCTT ACATATGATA   
  
080641 "GATTAACATT TCGAACAGGC ATGAATATAG CATCCATAGG" ATAACTTGCG TCTTGAAAAT TATTTGGCGT   
       CTAATTGTAA AGCTTGTCCG TACTTATATC GTAGGTATCC TATTGAACGC AGAACTTTTA ATAAACCGCA   
  
080711 TTGTATATGA TATCCGCGAT TCCTCTCGAT CTTTAATTCA "ATACACAAAT TAATTGGTTC CATTAGGTTA "  
       AACATATACT ATAGGCGCTA AGGAGAGCTA GAAATTAAGT TATGTGTTTA ATTAACCAAG GTAATCCAAT   
  
080781 "GCTATATGCT GTGTATTATC AACGATTTCC ACCGAAGGTG" GTAAGATGAT GTCTTGAGCA GTTACGCATC   
       CGATATACGA CACATAATAG TTGCTAAAGG TGGCTTCCAC CATTCTA"CTA CAGAACTCGT CAATGCGTAG "  
  
080851 CAGGACCCTT GACACAAATA GAC"GCATCAC GAGTTCCATA CAGATTACTT CTCAATACAA TTTCTTTCAA "  
       "GTCCTGGGAA CTGTGTTTAT CTGCGTAGTG CTCAAGGTAT GTCTAAT"GAA GAGTTATGTT AAAGAAAGTT   
  
080921 "ATTCATTAAA ATTTCATGTA CTG"ATTCTTG AATACCGACT ATGGTAGAGT ATTCATGTGG GATTTTTTCA   
       TAAGTAATTT TAAAGTACAT GACTAAGAAC TTATGGCTGA TACCATCTCA TAAGTACACC CTAAAAAAGT   
  
080991 GATTTTGCAC GTGTGATACA TGTTCCCTCT AT"TTCTCCAA GCAAAGCTTT TCGCATCGCA ATGCCTATAG "  
       CTAAAACGTG CACACTATGT ACAAGGGAGA TAAAGAGGTT CGTTTCGAAA AGCGTAGCGT TACGGATATC   
  
081061 "TATCCCCTTG CCCCTTCATA AGTGGAGACA GA"ATAAAGCG TCCATAATAA AGACGCTTAC TGTCTGCTCT   
       ATAGGGGAAC G"GGGAAGTAT TCACCTCTGT CTTATTTCGC AGGTATTATT TCTGCGAATG ACAGACGAGA "  
  
081131 TGATTCAACA CACTTCCACT TTAGT"GTCCG AGTCGATACT CTGATTTTCT CTCGAACCAT AGTAATATTT "  
       "ACTAAGTTGT G"TGAAGGTGA AATCACAGGC TCAGCTATGA GACTAAAAGA GAGCTTGGTA TCATTATAAA   
  
081201 "TTTGATCAAA TCATTGAATT ATTTA"TTTCT CTTGAAATTT CTCTTCAATG TTTATTTTTA CACACGTCGT   
       AAACTAGTTT AGTAACTTAA TAAATAAAGA GAACTTTAAA GAGAAGTTAC AAATAAA"AAT GTGTGCAGCA "  
  
081271 TTTTTAGGGG GCCTACAGCC ATTAT"GTGGC ATAGGGGTTA CATCCCGTAT GAAACTTAAT AATATACCGC "  
       "AAAAATCCCC CGGATGTCGG TAATACACCG TATCCCCAAT GTAGGGCATA CTTTGAA"TTA TTATATGGCG   
  
081341 "TTCTCCGAAT AGCTCTTAAT GCTGC"ATCTC TTCCGAGACC GGGGCCCTTT ATCATAACTT CGGCTCGTTG   
       AAGAGGCTTA TCGAGAATTA CGACGTAGAG AAGGCTCTGG CCCCGGGAAA TAGT"ATTGAA GCCGAGCAAC "  
  
081411 CATCCCTTGA TCCACTACTC C"CCGAATAGC ATTTCCTGCT GCGGTTTGAG CGGCAAATGG TGTCCCTCTT "  
       "GTAGGGAACT AGGTGATGAG GGGCTTATCG TAAAGGACGA CGCCAAACTC GCCG"TTTACC ACAGGGAGAA   
  
081481 "CTTGTACCTT TGAATCCACA C"GTACCGGCG GAGGACCAAG AAATTACCCG ACCCCGTACA TCTGTAACAG   
       GAAC"ATGGAA ACTTAGGTGT GCATGGCCGC CTCCTGGTTC TTTAATGGGC TGGGGCATGT AGACATTGTC "  
  
081551 TCACAATTGT ATTGTTGAAA CTTGCTTGAA CA"TGAATAAC GCCCTTTGGT ATTCTACGCG TACTTTTACG "  
       "AGTG"TTAACA TAACAACTTT GAACGAACTT GTACTTATTG CGGGAAACCA TAA"GATGCGC ATGAAAATGC "  
  
081621 "TGAGCTAATA CGTCCATTTC TACGTGAACC GA"TTCTTGGT ATGGGTTTTG CCATATTTTA TCATCTCATA   
       "ACTCGATTAT GCAGGTAAAG ATGCACTTGG CTAAGAACCA TACCCAAAAC GGT"ATAAAAT AGTA"GAGTAT "  
  
081691 AATCTAAGTC AGAGATATAT GGATATA"TCC ATTTCATGTC AAAACGGATC CTTTAATTTA TTTTTATGTG "  
       "TTAGATTCAG TCTCTATATA CCTATATAGG TAAAGTACAG TTTTGCCTAG GAAATTAAAT AAAA"ATACAC   
  
081761 "TATATTGTGG GTGGCCCTTA GGGGTAC"TTT TTTTTTATAA AGATTATCCT TGTCTTTGTT TATGTCTCGG   
       ATATAACACC CACCGGGAAT CCCCATGAAA AAAAAATATT TCTAATAGGA ACAGAAACAA ATACAGAGCC   
  
081831 ATTGGAACAA ATTACCATAA TT"CGTCTCCG CCTACGGATC AAGCGACATT TTTCACAAAT TTTACGAATG "  
       TAACCTTGTT "TAATGGTATT AAGCAGAGGC GGATGCCTAG TTCGCTGTAA AAAGTGTTTA AAATGCTTAC "  
  
081901 "GAGGCCCTTA TTTTCATATT TG"TTATTCCT TACCTTAATT CCGAATCTAC TTATTGGAAG AAAATAAGTT   
       "CTCCGGGAAT" AAAAGTATAA ACAATAAGGA ATGGAATTAA GGCTTAGATG AATAACCTTC TTTTATTCAA   
  
081971 TCTTGAAATT TTCTATTTCG AATTGTAT"CT CCCTACAAAA AAGAATATTG AGACTACTTC ACCTAATCAT "  
       AGAACTTTAA AAGATAAAGC TTAACATAGA GGGATGTTTT TT"CTTATAAC TCTGATGAAG TGGATTAGTA "  
  
082041 "TCGAATCTTT GTTTCGTAGT CTATAAAT"TA TACGCCTTCT GGTCCTGGTT GAATCGTAAC AACTTACTGA   
       "AGCTTAGAAA CAAAGCATCA GATATTTAAT ATGCGGAAGA CC"AGGACCAA CTTAGCATTG TTGAATGACT   
  
082111 AATTTTGACT ATCTATTACC TAGTATATGG ATA"AAACTAT GTCGAATCCT TCCTGAAACG TAACCTAGAA "  
       TTAAAACTGA TAGATAATGG ATCATATACC TAT"TTTGATA CAGCTTAGGA AGGACTTTGC ATTGGATCTT "  
  
082181 "TTGGATCCTC GTTTTCTAAA CAAACCCAAA ACA"TATCATT GGTAAGTGAT TCAGTAATTA AACTTTCAGA   
       "AACCTAGGAG CAAAAGATTT GTTTGGGTTT TGT"ATAGTAA CCATTCACTA AGTCATTAAT TTGAAAGTCT   
  
082251 AATCCTTTTT TGCTCTTTCA T"TCCAGATGA AACACCTTTC AAAGTATCAA TTAATGAAGG GGGAGTGGTA "  
       TTAGGAAAAA ACGAGAAAGT AAGG"TCTACT TTGTGGAAAG TTTCATAGTT AATTACTTCC CCCTCACCAT "  
  
082321 "TTATAAACTA CAAATCCACC T"CGTTTCTTT TTTTTTTTTT TACAAATAGG GAAGTTCTGT GTATAATTCG   
       "AATATTTGAT GTTTAGGTGG AGCA"AAGAAA AAAAAAAAAA ATGTTTATCC CTTCAAGACA CATATTAAGC   
  
082391 AATATCATAA AGATTACCAT ATATAACACA A"AATTTCGCC GCCGATTCCC TGTAGTCGAG CTTCTCGGTC "  
       TTATAGTATT TCTAATGG"TA TATATTGTGT TTTAAAGCGG CGGCTAAGGG ACATCAGCTC GAAGAGCCAG "  
  
082461 "TGTCATTATA CCCCGAGAAG TAGAAAGAAT T"ACAATGCCC ATTCCTCCTA AAATTCTAGG AATTTTTTGA   
       "ACAGTAATAT GGGGCTCT"TC ATCTTTCTTA ATGTTACGGG TAAGGAGGAT TTTAAGATCC TTAAAAAACT   
  
082531 TAGTTAGAAT ATATTCGGAG ACCGGGT"CGA CTGATCCGTT TTAAATTTAA AATCGTTTTA TATGGTCCTT "  
       ATCA"ATCTTA TATAAGCCTC TGGCCCAGCT GACTAGGCAA AATTTAAATT TTAGCAAAAT ATACCAGGAA "  
  
082601 "TCCTATTTCT TCTATGTCGT AGGGTTA"AAA CCCAAAAATC CTTGTTGCTT TCCCGATGTT TCCTTACGTT   
       "AGGA"TAAAGA AGA"TACAGCA TCCCAATTTT GGGTTTTTAG GAACAACGAA AGGGCTACAA AGGAATGCAA "  
  
082671 TTCAATAAAA CCTTCTCGTA AAAGTATTTT "AACAATGTTT TCGGTGATGT TAGTAGCTGG TATTCGAACC "  
       "AAGTTATTTT GGA"AGAGCAT TTTCATAAAA TTGTTACAAA A"GCCACTACA ATCATCGACC ATAAGCTTGG "  
  
082741 "ATTCCTTTTC TATTCATGTC AGCATTGCGA" ATAGAGGTTA TTATGTTAGC AATAGTGTCC TTACCCATGA   
       "TAAGGAAAAG ATAAGTACAG TCGTAACGCT TATCTCCAAT A"ATACAATCG TTATCACAGG AATGGGTACT   
  
082811 TAAACGAAAA TTATTGTTTA CTTAAAATTT TGAT"CTAATC AACATTCTTT TTTATTAAAT AGTTATGAAT "  
       ATTTGCTTTT AATAACAAAT GAATTTTAAA ACTAGATTAG TTGTAAGAAA AAATAATTTA TCAATACTTA   
  
082881 "TTGAAAAGGT ATATACGTGA TACACAATCT ACTA"ATTAAT TTCATTCAAA TAGTATAACT ATCTCACGGT   
       AACTT"TTCCA TATATGCACT ATGTGTTAGA TGATTAATTA AAGTAAGTTT ATCATATTGA TAGAGTGCCA "  
  
082951 CTGTCTCATT TTATAATACT TC"AGGTGCTA ATGAAATTAT TTTTGTGAAA TTTAACTGTC TCAATTCTCG "  
       "GACAG"AGTAA AATA"TTATGA AGTCCACGAT TACTTTAATA AAAACACTTT AAATTGACAG AGTTAAGAGC "  
  
083021 "GGCAATCGCA CCAAAAATTC GA"GTTCCTTT TGGATTTCCT TCTTGATCAA TAACAACCGC AGCATTGTCA   
       "CCGTTAGCGT GGTT"TTTAAG CTCAAGGAAA ACCTAAAGGA AGAACTAGTT AT"TGTTGGCG TCGTAACAGT "  
  
083091 TCATATCGTA TTATCATACC ATTTTCTCGT TTGA"GTTCTT TACAAGTACG TACAATTACA GCTCTGATCA "  
       "AGTATAGCAT AATAGTATGG TAAAAGAGCA AACTCAAGAA ATGTTCATGC AT"GTTAATGT CGAGA"CTAGT "  
  
083161 "CTTCTGATCT TTCTAAAGGT GTATTTGGGA CGGC"TTTCTT GATTACAGCA ACAATAACGT CACCAATATG   
       "GAAGACTAGA AAGATTTCCA CATAAACCCT GCCGAAAGAA CTAATGTCGT TGTTATTGCA GTGGT"TATAC   
  
083231 AGCATATCGT CGATTACTAG CCCCTATGAT TCGAATACA"C ATCAATTCTC GGGCTCCGCT GTTATCTGCT "  
       TCGTATAGCA GCT"AATGATC GGGGATACTA AGCTTATGTG TAGTTAAGAG CCCGAGGCGA CAATAGACGA "  
  
083301 "ACATTCAAAA GGGTTTGAGG TTGAATCATA TTATTTTGG"A ATCTTTTCTT TCAATGCAAA GGGCGAAGTA   
       "TGTAAGTTTT CCC"AAACTCC AACTTAGTAT AATAAAACCT TAGAAAAGAA AGTTACGTTT CCCGCTTCAT   
  
083371 AAAAAAAAAA GAAATATTGT TTTTCAAAAA "AAAAAAAAGA GAAATCTACA ATTGTTTTTT TTTCATCTCA "  
       TTTTTTTTTT CTTTATAACA AAAAGTTTTT "TTTTTTTTCT CTTTAGATGT TAACAAAAAA AAAGTAGAGT "  
  
083441 "AACAAAGATC GCTTTCCTTT GATTCTACAT" TTCTATCCCG AAGTAATGAA TTGGGTTCGT ATAGGCATTT   
       "TTGTTTCTAG CGAAAGGAAA CTAAGATGTA" AAGA"TAGGGC TTCATTACTT AACCCAAGCA TATCCGTAAA "  
  
083511 TGGATGCCGC TATTGAAATA GCTTTTCTGG CTAT"ATTTTC GGCTACTCCA CTCATTTCAT AAAGTATTCT "  
       "ACCTACGGCG ATAACTTTAT CGAAAAGACC GATA"TAAAAG CCGATGAGGT GAGTAAAGTA TTTCATAAGA   
  
083581 "ACCTGGTTTA ACAACAGCTA CCCAATATTC GGGG"GATCCT TTCCCTGAAC CCATACGTGT TTCTGTGGGT   
       TGGACCAA"AT TGTTGTCGAT GGGTTATAAG CCCCCTAGGA AAGGGACTTG GGTATGCACA AAGACACCCA "  
  
083651 CTTAGTGTAA CGGGTTTGTC TGGAAAT"ATA CGTACCCATA TTTTTCCACC GCGTCGTGCA TTTCGTGTCA "  
       "GAATCACA"TT GCCCAAACAG ACCTTTATAT GCATGGGTAT AAAAAGGTG"G CGCAGCACGT AAAGCACAGT "  
  
083721 "TTGCCCTTCG TCCGGCTTCG ATTTGTC"TAG ATGTGATCCA AGCGGGTTCA AGTGCCTGAA TAGCGTATCT   
       "AACGGGAAGC AGGCCGAAGC TAAACAGATC TACACTAGGT TCGCCCAAG"T TCACGGACTT ATCGCATAGA   
  
083791 CCCGAAGCAA ATACTATTGC CT"CGATAAGA TATTCCTTTC ATTCTTCCTC TATGGTGTTT ACGGAATCTG "  
       G"GGCTTCGTT TATGATAACG GAGCTATTCT ATAAGGAAAG TAAGAAGGAG ATACCACAAA TGCCTTAGAC "  
  
083861 "GTTCTTTTAG GGTTATAGTT GA"TGGTTATT TGTCAATTCC ATCTCTACTA CAGAACCGGA CATGAGAGTT   
       "C"AAGAAAATC CCAATATCAA CTACCAATAA ACAGTTAAGG TAGAGATGAT GTCTTGGCCT GTACTCTCAA   
  
083931 TCTTCTCATC CAGCTCCTCG CGAATGA"AAC GATTCAAAAA AAAAATATAA ATGTATTTAC TGCTATTTAC "  
       AGAAGAGTA"G GTCGAGGAGC GCTTACTTTG CTAAGTTTTT TTTTTATATT TACATAAATG ACGATAAATG "  
  
084001 "TGAATAATAG ATTCAATTAT GGGATTC"TTT GAAATTTCAT TTAATCAATC TATTCGAAAT TTGTATATCT   
       "ACTTATTAT"C TAAGTTAATA CCCTAAGAAA CTTTAAAGTA AATTAGTTAG ATAAGCTTTA AACATATAGA   
  
084071 TCTTTTCTTT TGATAGAAAA CTAAACTCTT TA"TAGATTCT AGAATAATAG AATAATTTTT TTTTTATTAT "  
       AGAAAAGAAA ACTAT"CTTTT GATTTGAGAA ATATCTAAGA TCTTATTATC TTATTAAAAA AAAAATAATA "  
  
084141 "AGTTTTCTAA AAAATTATAG ATAATATAAT TT"CGTTTTGT TATTTTTTCT TTTATTATTA AGGTTATAAC   
       "TCAAAAGATT TTTTA"ATATC TATTATATTA AAGCAAAACA ATAAAAAAGA AAATAATAAT TCCAATATTG   
  
084211 AAATCTTTAT TTTGTTTTAC CTTTTCTTTT TTTTATCTAT "TATTCTATTT GATCGACCCT AATTTAGAAA "  
       TTTAGAAATA AAACAAAATG GAAAAGAAA"A AAAATAGATA ATAAGATAAA CTAGCTGGGA TTAAATCTTT "  
  
084281 "TCTAATAAAA TTGAAACGTT CGCGGGCGAA TATTTACTCT" TTTTATATGT GTTTTGGTTC TCGGGTTAGT   
       "AGATTATTTT AACTTTGCAA GCGCCCGCT"T ATAAATGAGA AAAATATACA CAAAACCAAG AGCCCAATCA   
  
084351 TAGCCCATGA ACCCACCTCT CATAATAAAT GGATTGGTCT "TGGGTTCCTT CCGCCATCCT ACCCAATGAA "  
       ATCGGGTACT TGGGT"GGAGA GTATTATTTA CCTAACCAGA ACCCAAGGAA GGCGGTAGGA TGGGTTACTT "  
  
084421 "TTATTAGGAT TCGTTTTCAA TAGAATATTA TGTATTCACG" GGTTTCCTCG TTCCCATCGC CTCTCGATTA   
       "AATAATCCTA AGCAA"AAGTT ATCTTATAAT ACATAAGTGC CCAAAGGAGC AAGGGTAGCG GAGAGCTAAT   
  
084491 ATGGTTAGGT CTTAATTCTA "CAATGGAGCC CGTAATCAAA TTTGTTTTTG AGTCAATCTT CTCAGTCTTT "  
       TACCAATCCA GAATTAAGAT "GTTACCTCGG GCATTAGTTT AAACAAAAAC TCAGTTAGAA GAGTCAGAAA "  
  
084561 "ATTGTCTCGG GGCTCTTGGC" TTTTTTGTTC TATGAACAGA TTCATCTAAT TATGAATTCA TCAGTCTTAA   
       "TAACAGAGCC CCGAGAACCG" AAAAAACAAG ATACTTGTCT AAGTAGATTA ATACTTAAGT AGTCAGAATT   
  
084631 TGCTTTATTA CACTACCTTT "TATGAGATGA CCCATAGACC TTACATATTC CATATTGGAA TCATATATCA "  
       ACGAAATAAT G"TGATGGAAA ATACTCTACT GGGTATCTGG AATGTATAAG GTATAACCTT AGTATATAGT "  
  
084701 "TTCATATTCT TTTTCTCTCT" TTCTTTCACC CTTCCATTTA TCCGCATACT TTTATTTCTT CACAACTCAT   
       "AAGTATAAGA A"AAAGAGAGA AAGAAAGTGG GAAGGTAAAT AGGCGTATGA AAATAAAGAA GTGTTGAGTA   
  
084771 AATCGGATTG TTTTTTTTTT TTTTTATGCA AAAAAGATT"T CAGTTGCTAC AATGATATGA AAAATATAAC "  
       TTAGCCTAAC AAAAAAAAAA AAAAA"TACGT TTTTTCTAAA GTCAACGATG TTACTATACT TTTTATATTG "  
  
084841 "ATATCTTGAC TGGTTGGTTT TTTAGATCCA GATAATGCG"A AGCGATGAGT TGGTTATTAG TTTTATAATT   
       "TATAGAACTG ACCAACCAAA AAATC"TAGGT CTATTACGCT TCGCTACTCA ACCAATAATC AAAATATTAA   
  
084911 CTTAGTTCAG ATTATGTATG GGCTGATCCT TTTTTATTT"T TTGGTTTTAA TCCGAACACC TAAAAAAATA "  
       GAATCAAGTC TAATACAT"AC CCGACTAGGA AAAAATAAAA AACCAAAATT AGGCTTGTGG ATTTTTTTAT "  
  
084981 "AAAAAACCGA CGAGTCGCAC ACTAAGCATA GCAATTATC"T CAAATGATTA ATTTCATTTT TATTTAACCT   
       "TTTTTTGGCT GCTCAGCG"TG TGATTCGTAT CGTTAATAGA GTTTACTAAT TAAAGTAAAA ATAAATTGGA   
  
085051 TATCGAATTG CTCATTTTTT ATTTAAAGGA AAAAGA"CTGA TTTGTCATTT CTTGGTCGAT CATTGAATAA "  
       ATAGCTTAAC GAGTAAAAAA TAAATT"TCCT TTTTCTGACT AAACAGTAAA GAACCAGCTA GTAACTTATT "  
  
085121 "AACGTAAACA CAAGTTAAGT AAGGGCTTAT TATTGC"TCGT CTACAAATAT CCAAATTTTT ATGCCTAATA   
       "TTGCATTTGT GTTCAATTCA TTCCCG"AATA ATAACGAGCA GATGTTTATA GGTTTAAAAA TACGGATTAT   
  
085191 TCCCATAGAT AGTTCGAACT GGATAGCAAC AATAAT"CAAT TTTAGCTCGA ATGGTTTGTA GAGGAACCCG "  
       AGGGTATCTA TCAAGCTTGA CCTATCGTTG TTATTA"GTTA AAATCGAGCT TACCAAACAT CTCCTTGGGC "  
  
085261 "TCCTTCTCTG ATCCATTCGA CACGTGCAAT TTCTTT"TCCG TCTATACGTC CGGCAATTTG TACTTGAATT   
       "AGGAAGAGAC TAGGTAAGCT GTGCACGTTA AAGAAA"AGGC AGATATGCAG GCCGTTAAAC ATGAACTTAA   
  
085331 CCCTTTGTAC CCGCCTGTTC AGTTAATTCA ATAGCTTTTT "TCATTGCTTT GCGAAATGAA ACTCTATTCT "  
       GGGAAACATG GGCGGACAAG TCAATTAAGT TATCGAAAAA AGTAACGAAA C"GCTTTACTT TGAGATAAGA "  
  
085401 "TTAATTGTCC GGCTATAAAT TCTGCGAGAA TATTAGGGTG" TCCATAAGGG TTTCCTATTC TTGTAATAGC   
       "AATTAACAGG CCGATATTTA AGACGCTCTT ATAATCCCAC AGGTATTCCC A"AAGGATAAG AACATTATCG   
  
085471 AATGTTGAGT TTTCGGTTCC CAGAATTGAA TTCTTTTTGT "ACATTAATCT GTAATTCTTC GATTTTTCGA "  
       TTACAACTCA AAAGCCAAGG GTCTTAACTT AAGAAAAACA TGTAATTA"GA CATTAAGAAG CTAAAAAGCT "  
  
085541 "GACCCATCGT CTATTACTAA CTTTGGGAAT CCCATAAAGA" TTATCACTTG AATAAGATCA ATTCTTTTTT   
       "CTGGGTAGCA GATAATGATT GAAACCCTTA GGGTATTTCT AATAGTGA"AC TTATTCTAGT TAAGAAAAAA   
  
085611 GAATCTCGAT ACGTGCAATT CCTTCAACAC CAGAAA"AAAT TCTCATGTTT TTTTGTACAT AATTCTGGAT "  
       CTTAGAGCTA TGCACGTTAA GGAAGTTGTG GTCTTTTTTA AGAGTACAAA AAAACATGTA TTAAGACCTA   
  
085681 "ACAATCTCGT ATTTTTTGAT CTTCTTGTAA ACCTTC"AGAA TATTTTTTGG GGTGTGCAAA CCACAGAGAA   
       TGTTAGAGCA TAAAAAACTA GAAGAACATT TGG"AAGTCTT ATAAAAAACC CCACACGTTT GGTGTCTCTT "  
  
085751 TGATGCCCTT GGGTTGCACC AAGT"CGAAAA CCGAGTGGAT TTATTTTTTG TCCCATAATC CCCCATTATT "  
       "ACTACGGGAA CCCAACGTGG TTCAGCTTTT GGC"TCACCTA AATAAAAAAC AGGGTATT"AG GGGGTAATAA "  
  
085821 "ATACATGTCA TAACACCTCA TATC"TGTGTA CTTTTCTTTA GTTATCAATC CACCAGGTTG GTTTATGGTG   
       "TATGTACAGT ATTGTGGAGT ATAGACACAT GAAAAGAAAT CAATAGTTAG GTGGTCCA"AC CAAATACCAC   
  
085891 TATTGGTATC TTTTAAACTC TTCTTCATTC ACAGATG"TAT TTTTCAATAC AATAGTTATA GAACAAGTGG "  
       ATAACCATAG AAAATTTGAG AAGAAGTAAG TGTCTACATA AAAAGTTATG TTATCAA"TAT CTTGTTCACC "  
  
085961 "GTCGTTTTAT GGGATAACTT CGCCCTTGAG CTCGAGG"TTT TAATTTTTTC ACAGTAGTAC CTTGATTAAC   
       "CAGCAAAATA CCCTATTGAA GCGGGAACTC GAGCTCCAAA ATTAAAAAAG TGTCATC"ATG GAACTAATTG   
  
086031 TTCTGCTTTA CTAATGACTA AATCTCTTTC GTTAAAA"CCC ATATTGTGAC TAGCATTTGC TGCTGCCGAA "  
       AAGACGAAAT GATTACTGAT TTAGAGAAAG CAATTTTGGG TATAACACTG ATCGTAA"ACG ACGACGGCTT "  
  
086101 "TAAACCAATT TTAAAATTGG ATAACATGCT CGATAAG"GCA TGAGTTCGAG TATCATCATT GTTTCTTCGT   
       "ATTTGGTTAA AATTTTAACC TATTGTACGA GCTATTCCGT ACTCAAGCTC ATAGTAG"TAA C"AAAGAAGCA "  
  
086171 AGGAACGGCC ACGAATCTGA TCAA"TTACTC TTCGCGCTTT GTGAGGGGAC ATACATATAT GTTGACCTAA "  
       "TCCTTGCCGG TGCTTAGACT AGTTAATGAG AAGCGCGAAA CACTCCCCTG TATGTATATA C"AACTGGATT   
  
086241 "AGCGTATACT TCATATCGAT TTTT"TTTCGT CTTTATCATG TTTTTTCTCT TCTTTATTAT AAGGTTTACC   
       TCGCATATGA AGTATAGCTA AAAAAAAGCA GAAATAGTAC AAAAAAGAGA AGAA"ATAATA TTCCAAATGG "  
  
086311 TTTTAGTAAT GAACGATAGG TATCTATATT CACTATTCAC "TTTTTTTTTC ATAATATTAA TTATTAACGA "  
       "AAAATCATTA CTTGCTATCC ATAGATATAA GTGATAAGTG AAAAAAAAAG TATT"ATAATT AATAATTGCT   
  
086381 "CGAGATCTAT TATCATTTTT CACATGCCCC CGGAAATTGC" GAGTGGGTGC AAATTCTCCC AATTTATGAC   
       GCTCTAGATA "ATAGTAAAAA GTGTACGGGG GCCTTTAACG CTCACCCACG TTTAAGAGGG TTAAATACTG "  
  
086451 CTACCATGCG ATCCGTTATA TAAACAGG"CA AATGGTCCTT TCCATTATGA ACAGCGATAG TATGCCCAAT "  
       "GATGGTACGC" TAGGCAATAT ATTTGTCC"GT TTACCAGGAA AGGTAATACT TGTCGCTATC ATACGGGTTA "  
  
086521 "CATTGTTGGT ATAATGGTAG ATGCTCGG"GA CCAAGTTACT ATTATTTCTT TTTCTCCCTT TGTGTTAAGC   
       "GTAACAACCA TATTACCATC TACGAGCC"CT GGTTCAATGA TAATAAAGAA AAAGAGGGAA ACACAATTCG   
  
086591 TTATTTATTT TTTTTAATAA "ATGGTTTCCC ACAAAAGGAT TTTTTTTTAA TGATCGCGCC ATTTACCTCC "  
       AATAAATAAA AAAAATTATT TACCAAAGGG TGTTTTCCTA AAAAAAAATT ACTAGCGCGG TA"AATGGAGG "  
  
086661 "TATTTTTTTT TTTTTTGTAA" AGACGAAGAA ACAAATTAGA TTTTCTCTCT TATTTACTAC GGCGACGAAG   
       "ATAAAAAAAA AAAAAACATT TCTGCTTCTT TGTTTAATCT AAAAGAGAG"A ATAAATGATG C"CGCTGCTTC "  
  
086731 AATCAAATTA TCACTATATT TATTCCTTTT TCTACTTCT"T CTTCCAAGTG CAGGATAACC CCAAGGGGTT "  
       "TTAGTTTAAT AGTGATATAA ATAAGGAAAA AGATGAAGAA GAAGGTTCAC GTCCTATTGG G"GTTCCCCAA   
  
086801 "GCGGGTTTTT TTCTACCAAT TGGGGCCCTC CCTTCACCA"C CCCCATGGGG ATGGTCTACA GGGTTCATAA   
       CGCCCAAAAA AAGATGGTTA ACCCCGGGAG GGAAGTGGTG GGGGTACCC"C TACCAGATGT CCCAAGTATT "  
  
086871 CTACTCCTCT TACTACAGGA CGCT"TACCTA GCCAACATTT AGATCCGGCT CTACCCAAAC TTTTCTGGTT "  
       "GATGAGGAGA ATGATGTCCT GCGAATGGAT CGGTTGTAAA TCTAGGCCG"A GATGGGTTTG AAAAGACCAA   
  
086941 "TACCCCAGCA TTCCCCACTT GTCC"GACTGT TGCTGAGCAA TTTTTGGATA TCAAACGGAC CTCCCCAGAA   
       "ATGGGGTCGT AAGGGGTGAA CAGGCTGACA ACGACTCGTT AAAAACCTAT AGTTTGCCTG GAGGGGTCTT"   
  
087011 GGTAATTTTA ATGTGGCCGA TTTCCCCTCT TTTGCAA"TCA GTTTCGCTAC AGCACCTGCT GCTCTAGCTA "  
       CCATTAAAAT TACACCGGCT AAAGGGGAGA AAACGTTAGT CAAAGCGATG TCGTGGACGA CGA"GATCGAT "  
  
087081 "ATTGTCCCCC CTTCCCAAGT GTGATTTCTA TGTTATG"TAT GGCCGTGCCT AAGGGCATAT CGGTTGAAGT   
       "TAACAGGGGG GAAGGGTTCA CACTAAAGAT ACAATACATA CCGGCACGGA TTCCCGTATA GCC""AACTTCA "  
  
087151 AGATTCTTCT TTTTGATCAA TCAAAACCCC TTC"CCAAACT GTACAAGCTT CTTCCAAAGC ATACGGCTTT "  
       "TCTAAGAAGA AAAACTAGTT AGTTTTGGGG AAGGGTTTGA CATGTTCGAA GAAGGTTTCG TAT"GCCGAAA   
  
087221 "CTGGATGTAG ATGATGATAT CTATACATAT GGA"TCTTATA TATATGGTAC AATGAAGTAC CATATGAGTG   
       GACCTACATC TACTACTATA GATATGTATA CCTAGAATAT ATATACCATG TTACTTCATG GTATACTCAC   
  
087291 GATATATAGG AATCCAAATC TGCCGAATCA CTCATGTTA"T GATCTTCTAC ATCCTAGGTC TTCCCGTTCC "  
       "CTATATATCC TTAGGTTTAG ACGGCTTAGT GAGTACAATA CTAGAAGATG TAGGATCCAG AAGGGCAAGG"   
  
087361 "TTCATCTGGC TTATGTTCTT CATGTAGCAT TCAGACCGA"A TGACTCTATG AAATTACGTC GATACTTCCA   
       AAGTAGACCG AATACAAGAA GTACATCGTA AGTCTGGCTT ACTGAGATAC TTTAATGCAG CTATGAAGGT   
  
087431 CATATTACGG GTAACGTAGG AGAC"ATCTCT ATTTTTCCCC TCGGGAATCC TTAGAATTAC CACTGCTTAG "  
       GTATAA"TGCC CATTGCATCC TCTGTAGAGA TAAAAAGGGG AGCCCTTAGG AATCTTAATG GTGACGAATC "  
  
087501 "CTTTCAATTT GCCTCCGACC ATCA"AATGAA ATGTGAATAA CCCGTCCTCC TCTCTTTGAA ACAAGGGACG   
       "GAAAGT"TAAA CGGAGGCTGG TAGTTTACTT TACACTTATT GGGCAGGAGG AGAGAAACTT TGTTCCCTGC   
  
087571 CTTCTGGTTC TGTCGGTGCT TGAAACAATT TAGTCTTCT"C CATATTACTA TATCTCTAGA GTCAATAATT "  
       GAAGACCAAG ACA"GCCACGA ACTTTGTTAA ATCAGAAGAG GTATAATGAT ATAGAGATCT CAGTTATTAA "  
  
087641 "TTATATGAGG AACTACTGAA CTCAATCACT TGCTGCCGT"T ACTCTTCAGT TTTCTGTTGA GGTCTATCCT   
       "AATATACTCC TTG"ATGACTT GAGTTAGTGA ACGACGGCAA TGAGAAGTCA AAAGACAACT CCAGATAG"GA "  
  
087711 GTGGAGGTAC TCAAATTGGA TCAGTGATCG ATTT"CTAGGT TTCGTCGTAA ACCTAATTGG TTACTTCCAA "  
       "CACCTCCATG AGTTTAACCT AGTCACTAGC TAAAGATCCA AAGCAGCATT TGGATTAACC AATGAAGG"TT   
  
087781 "TTACGTAAAT CAATAGTTCA AACCGCACTC AAAG"GTAGGG CATTTCCCAT TTTTATAGGA ACTTCTGTAC   
       AATGCATTTA GTTATCAAGT TTGGCGTGA"G TTTCCATCCC GTAAAGGGTA AAAATATCCT TGAAGACATG "  
  
087851 CAGAAACAAT GGTATCTCCA AT"TATAGCCC CTCTGGGATG TAAAATATAT CTTTTCTCAC CATCCCCATA "  
       "GTCTTTGTTA CCATAGAGGT TAATATCGG"G GAGACCCTAC ATTTTATATA GAAAAGAGTG GT"AGGGGTAT "  
  
087921 "GTGTATGAGA CAAATGTATG CA"TTTCGATT AGGGTCGTAT TCTATGGTTA CGATTCTACC ATATATGTCT   
       "CACATACTCT GTTTACATAC GTAAAGCTAA TCCCAGCATA AGATACCAAT GCTAAGATGG TA"TATACAGA   
  
087991 TTTTCATTCC GTCGAAAATC GATTTTACGG T"ATAGACGCT TATGACCTCC CCCTCTATGC CCTGCGGTAA "  
       AAAAGTAAGG CAGCTTTTAG CTAAAATGCC ATATCTGCGA AT"ACTGGAGG GGGAGATACG GGACGCCATT "  
  
088061 "TGATTCCTCT GGCATTACGA CCTTTACCAC A"ACGATGCTG TCCATAGATC AAATTATTTC GTGGATTGGA   
       "ACTAAGGAGA CCGTAATGCT GGAAATGGTG TTGCTACGAC AG"GTATCTAG TTTAATAAAG CACCTA"ACCT "  
  
088131 TTTTACTTGA CTGTCTACGG CT"CCATTGCG TGTGCTCGGG GTAGAAGTTT TGTATAAATG TATCGCCATG "  
       "AAAATGAACT GACAGATGCC GAGGTAACGC ACACGAGCCC CATCTTCAAA ACATATTTAC ATAGCG"GTAC   
  
088201 "CTATTAAGTA TTTTGATTTA AG"TTCTTTTC TTTCTAAGAG GTGGAATAGA ATAACCCGGT TGAAGCGTAA   
       GATAATTCAT AAAACTAAAT TCAAGAAAAG AA"AGATTCTC CACCTTATCT TATTGGGCCA ACTTCGCATT "  
  
088271 TGATCATACG TCTGTAATGC AT"TGTATGTC CCATAATAGG TCCCATTCTT CTACCCTTTC CCGGGAGTCG "  
       "ACTAGTATGC AGACATTACG TAACATACAG GG"TATTATCC AGGGTAAGA"A GATGGGAAAG GGCCCTCAGC "  
  
088341 "ATGACTATTC ATAGCTATTA CC"TTGACACC AAAGAAGAGT TCGACCCAAT GCTTTATTTC TGTCCTAGTT   
       "TACTGATAAG TATCGATAAT GGAACTGTGG TTTCTTCTCA AGCTGGGTT"A CGAAATAAAG ACAGGATCAA   
  
088411 GATCCTGATT CGACATTAGA AGTATATTGA TTTT"TCCCCA ATAACCGAAT ACTTTTGTCT GTAAATACTG "  
       C"TAGGACTAA GCTGTAATCT TCATATAACT AAAAAGGGGT TATTGGCTTA TGAAAACAGA CATTTATGAC "  
  
088481 "CATATTTGAT TCCATCCATA AATTTTCTTC CCTA"TGAGTT CGAGTCTCAA TAAGAATGCG AGTTCTTACT   
       "G"TATAAACTA AGGTAGGTAT TTAAAAGAAG GGATACTCAA GCTCAGAGTT ATTCTTACGC TCAAGAATGA   
  
088551 GTTCATATGT TATGATATGA ATATACCACA CCAATTCGTT "ATGTATGGAT GATGAGATTC CATCGATACA "  
       CAAGTATACA ATACTATACT TATATGGTGT GGTTAAGCAA "TACATACCTA CTACTCTAAG GTAGCTATGT "  
  
088621 "GAGCCAATTC CAATAGACTT ATTGGAGGGT CCCATTGGCG" TGCATCCAGT AGGAATTGAA CCTACGAATT   
       "CTCGGTTAAG GTTATCTGAA TAACCTCCCA GGGTAACCGC" AC"GTAGGTCA TCCTTAACTT GGATGCTTAA "  
  
088691 CGCCAATTAT GAGTTGGGCG CTTTAACCAT TCAGCCAT"GG ATGCTTAGCG GGGATCCTCG TACATGGTGA "  
       "GCGGTTAATA CTCAACCCGC GAAATTGGTA AGTCGGTACC TA"CGAATCGC CCCTAGGAGC ATGTACCACT   
  
088761 "ATAACCAAAT TCCAATTGAA ATGAAATCTT TGGGGTAA"AT CAATGCAATT TAGGAGGACT CAATTCAGTG   
       TATTGGTTTA AGGTTAACTT TACTTTAGAA ACC"CCATTTA GTTACGTTAA ATCCTCCTGA GTTAAGTCAC "  
  
088831 AAAGGGCAGC AATTCAAATT ATGGATTTTA GAATTCAGAG AGATCAAGAA TTCTCATTAT TTCTTAGATT   
       "TTTCCCGTCG TTAAGTTTAA TACCTAAAAT CTT"AAGTCTC TCTAGTTCTT AAGAGTAATA AAGAATCTAA   
  
088901 CATGGACCCA ATTCAATTCA TTGGGATCTT TCATTCACAT TTTTTTCCAT CAAGAACGTT TTATAAAAC"T "  
       GTACCTGGGT TAAGTTAAGT AACCCTAGAA AGTAAGTGTA AAAAA"AGGTA GTTCTTGCAA AATATTTTGA "  
  
088971 "CTTAGACTCC CGAATTTGGA GTATCCTACT TTCACGCAAT TCACAGGGTT CAACAAGCAA TCGATATTT"C   
       "GAATCTGAGG GCTTAAACCT CATAGGATGA AAGTGCGTTA AGTGT"CCCAA GTTGTTCGTT AGCTATAAAG   
  
089041 ACGATCAAGG GTGTAGTACT ATTTGTAGTA GCGGTCGTCC TTATATATCG TATTAACAAT CGAAAGATGG   
       TGCTAGTTCC CACATCATGA TAAACATCA"T CGCCAGCAGG AATATATAGC ATAATTGTTA GCTTTCTACC "  
  
089111 TCGAAAGAAA AAATCTCTAT TTGACAGGGT TTCTTCCTAT ACCTATGAAT TTCATTGGAC CCAGAAATGA   
       "AGCTTTCTTT TTTAGAGATA AACTGTCCC"A AAGAAGGATA TGGATACTTA AAGTAACCTG GGTCTTTACT   
  
089181 TACATTGGAA GAATCTTTTG GCTCTTCCAA TATCAATAGG TTGATTGTTT TGCTCCTGTA TCTTCCAACT   
       ATGTAACCTT CTTAGAAAAC CGAGAAGGTT ATAGT"TATCC AACTAACAAA ACGAGGACAT AGAAGGTTGA "  
  
089251 AGTTTCGTTC AAGTAACGGA TTCTAGCCAA TTGAAAGGAT CTTCTGATCA ATCCAGAGAT CATTTCGATT   
       "TCAAAGCAAG TTCATTGCCT AAGATCGGTT AACTT"TCCTA GAAGACTAGT TAGGTCTCTA GTAAAGCTAA   
  
089321 CCATTAGTAA TGAGGATTCG GAATATCACA CATTGATCAA TCAAAAAGAG ATTCAACAAC CGCTTCCGGA   
       GGTAATCATT ACTCCTAAGC CTTATAGTGT GTAACTAGTT A"GTTTTTCTC TAAGTTGTTG GCGAAGGCCT "  
  
089391 AGAAATCGAA GAATTTCTCG GGAATCCTAC AAGATCCATT CGTTCTTTTT TCTCTGACAG ATGGTCAGAA   
       "TCTTTAGCTT CTTAAAGAGC CCTTAGGATG TTCTAGGTAA G"CAAGAAAAA AGAGACTGTC TACCAGTCTT   
  
089461 CTTCATATGG GTTCGAATCC TATTGAGAGG TCCACTAGAG ATCAGAAATT GTTGAAGAAA GAACAAGATG   
       GAAGTATACC CAAGCTTAGG ATAACT"CTCC AGGTGATCTC TAGTCTTTAA CAACTTCTTT CTTGTTCTAC "  
  
089531 TTTCTTTTGT CCCTTCCAGG CGATCGGAAA ATAAAGAAAT AGTTAATATA TTCAAGATAA TTACGTATTT   
       "AAAGAAAACA GGGAAGGTCC GCTAGC"CTTT TATTTCTTTA TCAATTATAT AAGTTCTATT AATGCATAAA   
  
089601 ACAAAATACC GTCTCAATTC ATCCTATTTC ATCAGATCCG GGATGTGATA TGGTTCCGAA GGATGAACTG   
       TGTTTTATGG CAGAGTTAAG TAGGATAAAG T"AGTCTAGGC CCTACACTAT ACCAAGGCTT CCTACTTGAC "  
  
089671 GATATGGACA GTTCCAATAA GATTTCATTC TTGAAGAAAA ATCCATTTAT TTATTTATTT CATCTATTCC   
       "CTATACCTGT CAAGGTTATT CTAAAGTAAG A"ACTTCTTTT TAGGTAAATA AATAAATAAA GTAGATAAGG   
  
089741 ATGACCGGAA CAGGAGGGGA TACACGTTAC ACCACGATTT TGAATCAGAA GAGAGATTTC AAGAAATGGC   
       TACTGGCCTT GTCCTCCCCT ATGTGCAATG TGGTGCTAA"A ACTTAGTCTT CTCTCTAAAG TTCTTTACCG "  
  
089811 AGATCTATTC ACTCTATCAA TAACCGAGCC GGATCTGGTG TATCATAAGG GATTTGCCTT TTCTATTGAT   
       "TCTAGATAAG TGAGATAGTT ATTGGCTCGG CCTAGACCA"C ATAGTATTCC CTAAACGGAA AAGATAACTA   
  
089881 TCCTACGGAT TGGATCAAAA ACAATTCTTG AATGAGGTAT TCAACTCCAG GGATGAATCG AAAAAGAAAT   
       AGGATGCCTA ACCTAGTTTT TGTTAAGAAC TTACTC"CATA AGTTGAGGTC CCTACTTAGC TTTTTCTTTA "  
  
089951 ATTTATTGGT TCTACCTCCT ATTTTTTATG AAGAGAATGA CTCTTTTTAT CGAAGGATCA GAAAAAAATG   
       "TAAATAACCA AGATGGAGGA TAAAAAATAC TTCTCT"TACT GAGAAAAATA GCTTCCTAGT CTTTTTTTAC   
  
090021 GGTCCGGACC TCCCCCGGGA ATGATTTGGA AGATCCAAAA CAAAAAATAG TGGTATTTGC TAGCAACAAC   
       CCAGGCCTGG AGGGGGCCCT TACTAAACCT TCTAGGTTTT GTTTTT"TATC ACCATAAACG ATCGTTGTTG "  
  
090091 ATAATGGAGG CAGTCAATCA ATATAGATTG ATCCGAAATC TGATTCAAAT CCAATATTAT AGGTACATAA   
       "TATTACCTCC GTCAGTTAGT TATATCTAAC TAGGCTTTAG ACTAAG"TTTA GGTTATAATA TCCATGTATT   
  
090161 GAAATGTATT GAATCGATTC TTTTTAATGA ATAGATCCGA TCGCGACTTG GAATATGGAA TTCAAAGGGA   
       CTTTACATAA CTTAGCTAAG AAAAATTACT TATC"TAGGCT AGCGCTGAAC CTTATACCTT AAGTTTCCCT "  
  
090231 TCAAATAGGA AATGATACTC TGAATCATAG AACTATAATG AAATATACGA TCAACCAACA TTTATCAAAT   
       "AGTTTATCCT TTACTATGAG ACTTAGTATC TTGA"TATTAC TTTATATGCT AGTTGGTTGT AAATAGTTTA   
  
090301 TTCAAAAAGG GTCAGAAGAA ATGGTTCGAT CCTCTTATTT TGATTTCTCG AACCGAGAGA TCCATGAATC   
       AAGTTTTTCC CAGTCTTCTT TACCAAGCTA GGAGAATAAA A"CTAAAGAGC TTGGCTCTCT AGGTACTTAG "  
  
090371 GGGATCCTAA TGCATATAGA TACAAATGGT CCAATGGGAG CAAGAATTTC CAGGAACATT TCATTTCTGA   
       "CCCTAGGATT ACGTATATCT ATGTTTACCA GGTTACCCTC G"TTCTTAAAG GTCCTTGTAA AGTAAAGACT   
  
090441 GCAGAAGAGC CATTTTCATT TTCAAGTAGT GTTCGATCGA TTACGTATTA ATCAATATTC GATTGATTGG   
       CGTCTTCTCG GTAAAAGTAA AAGTTCATCA C"AAGCTAGCT AATGCATAAT TAGTTATAAG CTAACTAACC "  
  
090511 TCTGAGGTTA TCGATAAAAA AGATTGGTCT AAGTCACTTC GTTTCTTTTT GTCCAAGTTA CTTGTTTTTT   
       "AGACTCCAAT AGCTATTTTT TCTAACCAGA T"TCAGTGAAG CAAAGAAAAA CAGGTTCAAT GAACAAAAAA   
  
090581 TGTCCAAGTT TCTTCTCTTT TTGTCTAACT CACTTCCTTT TTTCTTTGTG AGTTTCGGGA ATA"TCCCCAT "  
       ACAGGTTCAA AGAAGAGAAA AACAGATTGA GTGAAGGAAA A"AAGAAACAC TCAAAGCCCT TATAGGGGTA "  
  
090651 "TCATAGGTCC GAGATCCACA TCTATGAATT GAAAGGTCCG AATGATCGAC TCTGCAATCA GTT"GTTAGAA   
       "AGTATCCAGG CTCTAGGTGT AGATACTTAA CTTTCCAGGC T"TACTAGCTG AGACGTTAGT CAACAATCTT   
  
090721 TCAATAGGTC TTCAAATCGT TCATTTGAAA AAATTGAAAC CCTTCTTATT GGATGATCAT TATACTTCCC   
       AGTTATCCAG AAGTTTAGCA AGTAAACTTT T"TTAACTTTG GGAAGAATAA CCTACTAGTA ATATGAAGGG "  
  
090791 AAAAATCGAA ATTCTTGATC AATGGAGGAA CAATATCACC ATTTTTGTTC AATAAGATAC CAAAGTGGAT   
       "TTTTTAGCTT TAAGAACTAG TTACCTCCTT G"TTATAGTGG TAAAAACAAG TTATTCTATG GTTTCACCTA   
  
090861 GATTGACTCA TTCGATACTA GAAATAATCG CAGGAAATCC TTTGATAACA CGGATTCCTA TTTCTCAATG   
       CTAACTGAGT AAGCTATGAT CTTTATTAGC GTCCTT"TAGG AAACTATTGT GCCTAAGGAT AAAGAGTTAC "  
  
090931 ATATCCCACG ATCAAGACAA TTGGCTAAAT CCCGTGAAAC CATTTCATAG AAGTTCATTG ATATCTTCTT   
       "TATAGGGTGC TAGTTCTGTT AACCGATTTA GGGCAC"TTTG GTAAAGTATC TTCAAGTAAC TATAGAAGAA   
  
091001 TTTATAAAGC AACTCGACTT CGATTCTTGA ATAATCCACA TCACTTCTGC TTCTATTGTA ACAAAAGATT   
       AAATATTTCG TTGAGCTGAA GCTAAGAACT TATTAGGTG"T AGTGAAGACG AAGATAACAT TGTTTTCTAA "  
  
091071 CCCTTTTTAT GTGGATTATG TGGAAAAGGC CCGTATCAAT AATTATGATT TTACGTATGG ACAATTCCTC   
       "GGGAAAAATA CACCTAATAC ACCTTTTCCG GGCATAGTT"A TTAATACTAA AATGCATACC TGTTAAGGAG   
  
091141 AATATCTTGT TCATTCGCAA CAAAATATTT TCTTTGTGCG GCGGTAAAAA AAAACATGCT TTTTTGGAGA   
       TTATAGAACA AGTAAGCGTT GTTTTATAAA "AGAAACACGC CGCCATTTTT TTTTGTACGA AAAAACCTCT "  
  
091211 GAGATACTAT TTCACCAATC GAGTCACAGG TATCTAACAT ATTCATACCT AACGATTTTC CACAAAGTGG   
       "CTCTATGATA AAGTGGTTAG CTCAGTGTCC" ATAGATTGTA TAAGTATGGA TTGCTAAAAG GTGTTTCACC   
  
091281 TGACGAAAGG TATAACTTGT ACAAATCTTT CCATTTTCCA ATTCGATCCA ATCCATTCGT TCGTCGAGCT   
       ACTGCTTTCC ATATTGAACA TGTTTAGAAA GGTAAAAGG"T TAAGCTAGGT TAGGTAAGCA AGCAGCTCGA "  
  
091351 ATTTACTCGA TCGCAGACAT TTCTGTAACA CCTCTAACAG AGGGACAAAT AGTCAATTTT GAAAGAACTT   
       "TAAATGAGCT AGCGTCTGTA AAGACATTGT GGAGATTGT"C TCCCTGTTTA TCAGTTAAAA CTTTCTTGAA   
  
091421 ATTGTCAACC TCTTTCAGAT ATGAATCTAT CTGATTCAGA GGGGAAGAAC TTGCATCAGT ATCTCAATTT   
       TAACAGTTGG AGAAAGTCTA TACTTAGATA GAC"TAAGTCT CCCCTTCTTG AACGTAGTCA TAGAGTTAAA "  
  
091491 CAATTCAAAC ATGGGTTTGA TTCACACTCC ATGTTCTGAG AAATATTTAC CATCCGAAAA GAGGAAAAAA   
       "GTTAAGTTTG TACCCAAACT AAGTGTGAGG TAC"AAGACTC TTTATAAATG GTAGGCTTTT CTCCTTTTTT   
  
091561 CAGAGTCTTT ATCGAAAGAA ATGCCTTGAG AAAGGGCAGA TGTATAGAAC CTTTCAACGA GATAGTGCTT   
       GTCTCAGAAA TAGCTTTCTT TACGGAACT"C TTTCCCGTCT ACATATCTTG GAAAGTTGCT CTATCACGAA "  
  
091631 TTTCAACTCT CTCAAAATGG AATCTATTCC AAACATATAT GCCATGGTTC TTTACTTCGA CAGGGTACAA   
       "AAAGTTGAGA GAGTTTTACC TTAGATAAG"G TTTGTATATA CGGTACCAAG AAATGAAGCT GTCCCATGTT   
  
091701 ATATCTAAAT TTGCTATTTT TAGATACTTT TTCAGACCTA TTGCCGATAC TAAGTAGCAG CCCAAAATTT   
       TATAGATTTA AACGATAAAA ATCTATGAA"A AAGTCTGGAT AACGGCTATG ATTCATCGTC GGGTTTTAAA "  
  
091771 GTATCCATTT TTGATGATAT TATGCATCGA TCAGATAGAT CATGGCGAAT TCTTCGGAAA AAATTGTGTC   
       "CATAGGTAAA AACTACTATA ATACGTAGC"T AGTCTATCTA GTACCGCTTA AGAAGCCTTT TTTAACACAG   
  
091841 TTCCACAATG GAATCTGATA AGTGAGATTT CGATTAAGTG TTTACCTAAT CTTCTTCTGT CCGAAGAAAT   
       AAGGTGTTAC CTTAGACTAT TCACTCTAAA GCTAATTCAC AAAT"GGATTA GAAGAAGACA GGCTTCTTTA "  
  
091911 GATTCATCGA AATAATGAGT CACCATCGAT ATCGACACAT CTGAGATCGC CAAATGTTCG GGAGTTCCTC   
       "CTAAGTAGCT TTATTACTCA GTGGTAGCTA TAGCTGTGTA GACT"CTAGCG GTTTACAAGC CCTCAAGGAG   
  
091981 TATTCAATCC TTTTCCTTCT TCTTATTGCT GTATATCTCG TTCGTACACA TCTTCTCTTT GTTTCTCGAG   
       ATAAGTTAGG AAAAGGAAGA AGAATAACGA CATATA"GAGC AAGCATGTGT AGAAGAGAAA CAAAGAGCTC "  
  
092051 CCTATAGTGA GTTACAGACA GAGTTCGAAA AGGTCAAATC TTTGATGATT CCATCATACA TGATTGAGTT   
       "GGATATCACT CAATGTCTGT CTCAAGCTTT TCCAGT"TTAG AAACTACTAA GGTAGTATGT ACTAACTCAA   
  
092121 GCGAAAACTT CTGGATAGGT ATCCCACATA TGAACGGAAT TCTTTCTGGT TAAAGAATCT CTTTCTAGTT   
       CGCTTTTGAA GACCTATCCA TAGGGTGTAT "ACTTGCCTTA AGAAAGACCA ATTTCTTAGA GAAAGATCAA "  
  
092191 GCTCTGGAAC AATTAGGAGA TTCTCTAGAA GAAATATGGG GTTCTGCTTC TGGTGGCGGT CCCGCTTATG   
       "CGAGACCTTG TTAATCCTCT AAGAGATCTT" CTTTATACCC CAAGACGAAG ACCACCGCCA GGGCGAATAC   
  
092261 GGGTCAAATC AATACGTTCG AAGAAGAAAG ATTGGAATAT CAATCTCATC AATCTCATAA GTATCATACC   
       CCCAGTTTAG TTATGCAAGC TTCTTCTTTC TAACCTTATA "GTTAGAGTAG TTAGAGTATT CATAGTATGG "  
  
092331 AAATCCCATC AATCGAATCG CTTTTTCGAG AAATACGAGA CATCTAAGTC ATACAAGTAA AGAGATATAT   
       "TTTAGGGTAG TTAGCTTAGC GAAAAAGCTC TTTATGCTCT" GTAGATTCAG TATGTTCATT TCTCTATATA   
  
092401 TCATTGATAA GAAAAAATGT GAACGGTGAT TGGATTGATG ATAAAATCCA ATCCTGGGTC TGGAACAGTG   
       AGTAACTATT CTTTTTTACA CTTGCCACTA ACCTAACTAC TATTTT"AGGT TAGGACCCAG ACCTTGTCAC "  
  
092471 ATTCGATTGA TGATAAAGAA AGAGAATTCT TGGTTCAGTT CTCCACCTTA ACGACAGAAA AAAGGATTGA   
       "TAAGCTAACT ACTATTTCTT TCTCTTAAGA ACCAAGTCAA GAGGTG"GAAT TGCTGTCTTT TTTCCTAACT   
  
092541 TCAAATTCTA TTGAGTCTGA CTCATAGTGA TCATTTATCA AAGAATGACT CTGGTTATCA AATGATTGAA   
       AGTTTAAGAT AACTCAGACT GAGTAT"CACT AGTAAATAGT TTCTTACTGA GACCAATAGT TTACTAACTT "  
  
092611 CAACCGGGAG TAATTTACTT ACGATACTTA GTTGACATTC AGAAAAAGTA TCTAATGAAT TATAAGTTCA   
       "GTTGGCCCTC ATTAAATGAA TGCTAT"GAAT CAACTGTAAG TCTTTTTCAT AGATTACTTA ATATTCAAGT   
  
092681 ATACATCCT"G TTTAGCAGAA AGACGGACAT TCCTTGCTCA TTATCAGACA ATCACTCATT CACAAACCTC "  
       TATGTAGGAC AAATCGTCTT TCTGCCTGTA AGGAA"CGAGT AATAGTCTGT TAGTGAGTAA GTGTTTGGAG "  
  
092751 "GTGTGGGGC"T AATAGTTTTC ATTTCCCATC TCATGGAAAA CTCTTTTCGC TCCGCTTAGC CCTCTCCCCG   
       "CACACCCCGA TTATCAAAAG TAAAGGGTAG AGTAC"CTTTT GAGAAAAGCG AGGCGAATCG GGAGAGGGGC   
  
092821 GGTATTTTAG TGATAGGTTC TATAGGAACT GGACGATCCT ATTTGGTCAA ATACCTAGCG ACAAACTCCT   
       CCATAAAATC ACTATCCAAG ATATCCTTGA "CCTGCTAGGA TAAACCAGTT TATGGATCGC TGTTTGAGGA "  
  
092891 ATCTTCCTTT CATTACAGTA TTTCTGAACA AGTTCCGGGA TAACAAGCCT AAGTTTATTG ATGATAGTGA   
       "TAGAAGGAAA GTAATGTCAT AAAGACTTGT" TCAAGGCCCT ATTGTTCGGA TTCAAATAAC TACTATCACT   
  
092961 CGATGATAGT GACGATATTG ATGATAGTGG CGATATTGAT GATAGTGACG ATATCGACCG TGACCTTGAT   
       GCTACTATCA CTGCTATAAC TACTATCACC GCTATAACTA CTATCA"CTGC TATAGCTGGC ACTGGAACTA "  
  
093031 ATTGATACGG AGCTGGAGCT TCTAACTATG ATGAATGCGC TAACTATGGA GATGAAGCTG GAAATAGACC   
       "TAACTATGCC TCGACCTCGA AGATTGATAC TACTTACGCG ATTGAT"ACCT CTACTTCGAC CTTTATCTGG   
  
093101 AATTTTATAT CACCCTTCAA TTCGAATTAA TAAAAGCAAT GTCTCCTTGC ATAATATGGA TTCCAAACAT   
       TTAAAATATA GTGGGAAGTT AAGCTTAATT ATTTTCGTTA CAGA"GGAACG TATTATACCT AAGGTTTGTA "  
  
093171 TCATGATCTG TATGTGAATA AGTCGAGTCA CTTATACTTT GGTCTATTAG TGAACTATCT CTACAGGGAT   
       "AGTACTAGAC ATACACTTAT TCAGCTCAGT GAATATGAAA CCAG"ATAATC ACTTGATAGA GATGTCCCTA   
  
093241 TTTGAAAGAT GTTCCACTAC AAATATTCTT GTTATTGCTT CGACCCATAT TCCCCAAAAA GTGGATCCCG   
       AAACTTTCTA CAAGGTGATG TTTATAAG"AA CAATAACGAA GCTGGGTATA AGGGGTTTTT CACCTAGGGC "  
  
093311 CTCTAATAGC TCCGAATAAA TTGAATACAT GCATTAAAAT ACGAAGGCTT CTTATTCCAC AACAACGAAA   
       "GAGATTATCG AGGCTTATTT AACTTATG"TA CGTAATTTTA TGCTTCCGAA GAATAAGGTG TTGTTGCTTT   
  
093381 GCACTTTTTC ACTCTTTCAT ATACTAGGGG ATTTCACTTG GAAAAGAAAA TGTTCCATAC TAATGGATTC   
       CGTGAAAAAG TGAGAAAGTA TATGAT"CCCC TAAAGTGAAC CTTTTCTTTT ACAAGGTATG ATTACCTAAG "  
  
093451 GGTTCCATAA C"CATGGGTTC CAATGTACGA GATCTTGTAG CACTTACCAA TGAGGCCCTA TCGATTAGTA "  
       "CCAAGGTATT GGTACCCAAG GTTACA"TGCT CTAGAACATC GTGAATGGTT ACTCCGGGAT AGCTAATCAT   
  
093521 "TTACGCAGAG G"AAATCAATT ATAGACACTA ATATAATTAG ATCTGCTCTT CATAGACAAA CTTGGGATTT   
       AATGCGTCTC CTTTAGTTAA TATCTGTGAT TATATTAATC TAG"ACGAGAA GTATCTGTTT GAACCCTAAA "  
  
093591 GCGATCCCAG GTAAGATCAG TTCAGGATCA TGGGATCCTT TTCTATCAGA TAGGAAGGGC TCTTGCACAA   
       "CGCTAGGGTC CATTCTAGTC AAGTCCTAGT ACCCTAGGAA AAG"ATAGTCT ATCCTTCCCG AGAACGTGTT   
  
093661 AATGTACTTC TAAGTAATTG CTCCATAGAT CCTATATCTA TCTATATGAA GAAGAAATCA TGTAACGAAG   
       TTACATGAAG ATTCATTAAC GAGGTATCTA GGATATAGAT AGATA"TACTT CTTCTTTAGT ACATTGCTTC "  
  
093731 GGGGTTCTTA TTTGTACAAC TGGTACTTCG AACTTGAAAC GAGCATGAAG AAATTAACGA TACTTCTTTA   
       "CCCCAAGAAT AAACATGTTG ACCATGAAGC TTGAACTTTG CTCGT"ACTTC TTTAATTGCT ATGAAGAAAT   
  
093801 TCTTTTGAAT TGTTCTGCCG GATCGGTCGT TCAAGACCTT TGGTCTCTAT CCGGACCCGA TGAAAAAAAT   
       AGAAAACTTA ACAAGACGGC CTAGCCAGCA AGTTCTGGA"A ACCAGAGATA GGCCTGGGCT ACTTTTTTTA "  
  
093871 GGGATCACTT CTTATGTACT CGTTGAGAAT GATTCTCATC TAGTTCATGG CCTATTAGAA GTAGAAGGCG   
       "CCCTAGTGAA GAATACATGA GCAACTCTTA CTAAGAGTA"G ATCAAGTACC GGATAATCTT CATCTTCCGC   
  
093941 CTCTGTTTGG ATCCTCATGG ACAGAAAAAG ATTGCAGTCG ATTTGATAAT GATCGAGTGA CATTGCTTCT   
       GAGACAAACC TAGGAGTACC TGTCTTTTTC TAACGTC"AGC TAAACTATTA CTAGCTCACT GTAACGAAGA "  
  
094011 TCGGCCCGAA CCCAGGAATC CCTTAGATAT GATACAAAAT GGATCTTCTT CTATCGTTGA TCAGATATTT   
       "AGCCGGGCTT GGGTCCTTAG GGAATCTATA CTATGTT"TTA CCTAGAAGAA GATAGCAACT AGTCTATAAA   
  
094081 CTCTATCAAA AATACGAATC GAAGTTTGAA GAAGGGGAAG GAGTCCTCGA CCCGCAACAG ATAGAGGAGG   
       GAGATAGTTT TTATGCTTAG CTTCAAACTT CTTC"CCCTTC CTCAGGAGCT GGGCGTTGTC TATCTCCTCC "  
  
094151 ATTTATTCAA TCACATAGTT TGGGCTCCTA GAATATGGAG CCCTTGGGGC TTTCTATTTG ATTGTATCGA   
       "TAAATAAGTT AGTGTATCAA ACCCGAGGAT CTTA"TACCTC GGGAACCCCG AAAGATAAAC TAACATAGCT   
  
094221 AAGGCCCAAT GAATTGGGAG TTCCCTATTG GGCTAGGTCA TTTCGGGGCA AGCGGATCAT TTATGATGAA   
       TTCCGGGTTA CTTAACCCTC AAGGGATAAC CCGATC"CAGT AAAGCCCCGT TCGCCTAGTA AATACTACTT "  
  
094291 GAGGATGAGC TTCAAGAGAA TGATTCGGAG TTCTTGCAGA GTGGAACCGT GCAGTACCAG ACACGAGATA   
       "CTCCTACTCG AAGTTCTCTT ACTAAGCCTC AAGAAC"GTCT CACCTTGGCA CGTCATGGTC TGTGCTCTAT   
  
094361 GCTCTTCCAA AGAACAAGGC TTTTTTCTAA TAAACCAATT CATTTGGGAT CCTGCAGATC CACGGTCCTT   
       CGAGAAGGTT TCTTGTTCCG AAAAAAGATT ATTTGGTTAA GTAAAC"CCTA GGACGTCTAG GTGCCAGGAA "  
  
094431 TTTCCTATTC CAAGGATCAT CCTTTTGTCT CTGGTGTTTT CACATCCGAG AATTCTTTGC AGATGAAGAG   
       "AAAGGATAAG GTTCCTAGTA GGAAAACAGA GACCACAAAA GTGTAGGCTC TT"AAGAAACG TCTACTTCTC   
  
094501 ATGGCAAAGG GGCTGCTTAC TTCCCAACCA GCTTTTCCTA CATCTCTAGA AAAACCCTGG TTTATCAATA   
       TACCGTTTCC CCGACGAATG AAGGGTTGGT CGAAAAGGA"T GTAGAGATCT TTTTGGGACC AAATAGTTAT "  
  
094571 TCAAGAATAC GCAAGAAAAG TACGTCGAAT TGTTGATTCA TCACCAGAGA TGGCTTAAAA CTAGAACCAA   
       "AGTTCTTATG CGTTCTTTTC ATGCAGCTTA ACAACTAAG"T AGTGGTCTCT ACCGAATTTT GATCTTGGTT   
  
094641 TAGTTCATTA TCTAAATCTA AGGGATTTTT CCGTTCTAAT ACTCTATCCC AAAGTTATCA GTATTTATCA   
       ATCAAGTAAT AGATTTAGAT TCCCTAAAAA GGCAAGATTA TGAGATAGGG TT"TCAATAGT CATAAATAGT "  
  
094711 AATCTGTTCC TATCTAACGG AACACTATTG GATCAAATGA CAAAGACATT GTTGAGAAAA AGATGGCTTT   
       "TTAGACAAGG ATAGATTGCC TTGTGATAAC CTAGTTTACT GTTTCTG"TAA CAACTCTTTT TCTACCGAAA   
  
094781 TCCCGGATGA AATGAAATGA AAATTGGATT "CATGTAACAA GAGAAAGGTT TCCCATTCCT TAGCCGGAAA "  
       AGGGCCTACT TTACTTTACT TTTAACCTAA GTACATTGTT CTCTTT"CCAA AGGGTAAGGA ATCGGCCTTT "  
  
094851 "AATATGTGGC CATGAAATAG GGATTAAGTG" GAACAGAATT GACTGGGTGG TAGAGTCGTG AAAACACCAG   
       "TTATACACCG GTACTTTATC CCTAATTCAC CTTGTCTTAA CTGA"CCCACC ATCTCAGCAC TTTTGTGGTC   
  
094921 TTTCTTCCAT "ATTTTGGACC TTAGCTCCAT GGAACAATAT ACTACTGCTG AAACATGGAA GAATTGAAAT "  
       AAAGAAGGTA TAAAACCTGG AATCGAGGTA CCTTGTTAT"A TGATGACGAC TTTGTACCTT CTTAACTTTA "  
  
094991 "CTTAGATCCA" TACATAATGT TTTGATGGTA TGAACTGCCT AAACCAGAAT TCTTGAACAG CGAACAACCA   
       "GAATCTAGGT ATGTATTACA AAACTACCAT ACTTGACGG"A TTTGGTCTTA AGAACTTGTC GCTTGTTGGT   
  
095061 GAGTTATTAC TCACTACATC AAAAAA"TTTC CATTAATGAA AGATGTAAAT CCATTGGAAA ATCAAAAATA "  
       CTCAATAATG AGTGATGTAG TTTTTTAAAG GTAATTA"CTT TCTACATTTA GGTAACCTTT TAGTTTTTAT "  
  
095131 "CGCATGCCGA ATGAAATGGT TGTTGC"TATC TGTTACAATA ACGAATCATT GGTTTAACTG AATAACTAAA   
       "GCGTACGGCT TACTTTACCA ACAACGATAG ACAATGT"TAT TGCTTAGTAA CCAAATTGAC TTATTGATTT   
  
095201 TAAAATAGAT AGACCCCTCT CTTCGT"CTTA GGTCGACGGA TCTTCTCCAT TGAAAGATCC CCTATATGGA "  
       ATTTTATCTA TCTGGGGAGA GAAGCAGAAT CCA"GCTGCCT AGAAGAGGTA ACTTTCTAGG GGATATACCT "  
  
095271 "TAATACACAT TCCAGTTCAA CGAGCC"TAAT TCTAATTGTT TTGTTCCGAA GCAAACCACG GGGCGGTTCG   
       "ATTATGTGTA AGGTCAAGTT GCTCGGATTA AGA"TTAACAA AACAAGGCTT CGTTTGGTGC CCCGCCAAGC   
  
095341 TCCTATTCAG ATATTCACGA "CCAAGAAACA CTGGATTCTC TTTCGGGTAG GCCCTGAAAG GAGAAGGAAG "  
       AGGATAAGTC TATAAGTGCT GGTTCTTTGT GACCTAAGAG AAAGCCCATC "CGGGACTTTC CTCTTCCTTC "  
  
095411 "GCTGGAATGC CAACAGGCCT" CTATTATTGA ATTCACCCGA CCCGATAGTA CCCCATTTTT GGAACGTCCA   
       "CGACCTTACG GTTGTCCGGA GATAATAACT TAAGTGGGCT GGGCTATCAT" GGGGTAAAAA CCTTGCAGGT   
  
095481 GTGCCAAAGT CA"CTGAATGG GTAAGTCGCC AATCCCTAAA ACGGACTATG TAATGTACTT TATCTGCTGG "  
       CACGGTTTCA GTGACTTACC CATTCAGCGG TTAGG"GATTT TGCCTGATAC ATTACATGAA ATAGACGACC "  
  
095551 "GTTACGGGTG GG"CATTTTAC CAGAGGTTTC TATTGTATCA ATCTACCCTT GTTTAATTCC TGTTGAAGCA   
       "CAATGCCCAC CCGTAAAATG GTCTCCAAAG ATAAC"ATAGT TAGATGGGAA CAAATTAAGG ACAACTTCGT   
  
095621 TATACTCGGG GGGTG"GGTGC AGGGCGGACG ATTTCAAAGC GGACTCCCAT TCATTAGATA GAGAAGATCG "  
       ATATGAGCCC CCCACCCACG TCCCGCCTGC TAAAGTTT"CG CCTGAGGGTA AGTAATCTAT CTCTTCTAGC "  
  
095691 "CCAAGATTTC GTGAT"CCGCT GCCGAACTTA TTCCTTCCAA TTCAACGGGC ATTCTCAACA TTATGCCTTG   
       "GGTTCTAAAG CACTAGGCGA CGGCTTGAAT AAGGAAGG"TT AAGTTGCCCG TAAGAGTTGT AATACG"GAAC "  
  
095761 AAGAGGACTC GAACCTCC"AC GCTCTTTAGC ACGAGATTTT GAGTCTCGCG TGTCTACCAT TTCACCACCA "  
       "TTCTCCTGAG CTTGGAGGTG CGAGAAATCG TGCTCTAAAA CTCAGAGCGC ACAGATGGTA AAGTGG"TGGT   
  
095831 "AGGCATCTTG AAAGTGAA"TC ATATTCCATG AATATGATAT CTATCTAGTG TGATGTATGG AATATATGAC   
       TCCGTAGAAC TTTCACTTAG TATAAGGTAC TTATACTATA GATAGATCAC ACTACATACC TTATATACTG   
  
095901 AAAGGTGGAG TGTTAGAGTA TTTCTA"TTGC TCGGTCATGT CATATAGGTC CGAGTCGGAC ATCCAATTGC "  
       TTTCC"ACCTC ACAATCTCAT AAAGATAACG AGCCAGTACA GTATATCCAG GCTCAGCCTG TAGGTTAACG "  
  
095971 "TTCGATTTGA ATTATCCGGA GGATGC"CTTA CTATTATATC AAAAAGATGG ACAATCAAAC CTATTTCTCG   
       "AAGCT"AAACT TAATAGGCCT CCTACGGAAT GATAATATAG TTTTTCTACC TGTTAGTTTG GATAAAGAGC   
  
096041 ATTCAATAGA AGCCCAAA"GA GATGAATAGG GTCCCAAATA ACGAGAGATA TGTAAAAAAC AGGTCCGATT "  
       TAAGTTATCT TCGGGTTT"CT CTACTTATCC CAGGGTTTAT TGCTCTCTAT ACATTTTTTG TCCAGGCTAA "  
  
096111 "ACACCTATTC CTAATCCT"AA ATGGAATGTA AGGACGTAGG GATCCATATG TAAACATAGT ATCTATTTAA   
       "TGTGGATAAG GATTAGGA"TT TACCTTACAT TCCTGCATCC CTAGGTATAC ATTTGTATCA TAGATAAATT   
  
096181 ATACGCTCGG ATGACCCCTT CT"CATAATTA GAATGTATAT AACCTAGCCC TATTCCGGTC TGGCCCGGTA "  
       TAT"GCGAGCC TACTGGGGAA GAGTATTAAT CTTACATATA TTGGATCGGG ATAAGGCCAG ACCGGGCCAT "  
  
096251 "TGGAATGAAC TTATAATCAT GG"AATCGACT CGATCATCAG ATTATAGATT ATAAGTTCAT AACCCTAGCC   
       "ACC"TTACTTG AATATTAGTA CCTTAGCTGA GCTAGTAGTC TAATATCTAA TATTCAAGTA TTGGGATCGG   
  
096321 CATTCCCATT TTGG"GCGGAA CAGATCTACT AATTCTTTGA TTCCAGTTAG TAAGAGGGAT CTTGAACTAA "  
       GTAAGGGTAA AACC"CGCCTT GTCTAGATGA TTAAGAAACT AAGGTCAATC ATTCTCCCTA GAACTTGATT "  
  
096391 "GAAATAGATT CTAG"AAGCTA AACTAAAAAA GGGTATCCTG AGCAATTTCA ATAATCGGGT TCATTGATAT   
       "CTTTATCTAA GATC"TTCGAT TTGATTTTTT CCCATAGGAC TCGTTAAAGT TATTAGCCCA AGTAACTATA   
  
096461 TCCTGGTATA GTAGATGCTA TCACACAT"AC AATCATACTC AATTCGATGG AATTGTTTGA TCTTAAAGGA "  
       AGGACCATAT CATCTACGAT AGTGTGTA"TG TTAGTATGAG TTAAGCTACC TTAACAAACT AGAATTTCCT "  
  
096531 "GATCTTCTAT AATTTCGCAC GTGAGGGG"TT ATTTCTTGGT TTCGTCCAGT CATTAATAAC TTGATTATTT   
       "CTAGAAGATA TTAAAGCGTG CACTCCCC"AA TAAAGAACCA AAGCAGGTCA GTAATTATTG AACTAATAAA   
  
096601 TTAGATAATA GTAGATA"GAA ACAACGCTCG TAAGTAGTCC TATTGAAACC AAGAAATATA GGCCTGCCTG "  
       AATCTATTAT CATCTAT"CTT TGTTGCGAGC ATTCATCAGG ATAACTTTGG TTCTTTATAT CCGGACGGAC "  
  
096671 "CCATCCACAC CAGAATA"AAT GAAGTTTTCC GAAAAAACCT GCTAGTGGAG GAAGACCTCC TAAGGATAAG   
       "GGTAGGTGTG GTCTTAT"TTA CTTCAAAAGG CTTTTTTGGA CGATCACCTC CTTCTGGAGG ATTCCTATTC   
  
096741 AGACATAGGG CTAAAGAGA"G AGCCAAAAGA GGATCTTTTG TGTATAATCC TGCATAATCT CGAATGTTAT "  
       TCTGTATCCC GATTTCTCTC TCGGTTTTCT CCTAGAAAAC ACATATTAGG ACGTATTAGA GCTTACAATA   
  
096811 "CAGTTCCGGT ACGTAGACC"A AATGATACAA TGCGAGCAAA AGTTCCTAGA TTCATGGCGA TATAGAAGAG   
       GTCAAGGCCA TGCATCTGGT TTACTATGTT ACGCTCGTTT TCAAGGAT"CT AAGTACCGCT ATATCTTCTC "  
  
096881 CATATAAGTT AT"CATGCTTG CATATCCACC ATTTGAGTCT CCAACAATTA TTCCAATAAT TACATATCCG "  
       "GTATATTCAA TAGTACGAAC GTATAGGTGG TAAACTCAGA GGTTGTTA"AT AAGGTTATTA ATGTATAGGC   
  
096951 "ATTTGACCGA TG"GACGAATA CGCAAGCATA CGTTTCATGC TTGTTTGAGT AATAGCAATG AGATTCCCCA   
       TAAACTGGCT ACCTGCTTAT GCGTTCGTAT GCAAAGTACG AACAAACTCA TTATCG"TTAC TCTAAGGGGT "  
  
097021 ATATCATGCT AAGAATAGCT AGGA"TTTCCA GAAGAAGATG CCATTCGTTT GATGAGAAAT AAAAAGGAAT "  
       "TATAGTACGA TTCTTATCGA TCCTAAAGGT CTTCTTCTAC GGTAAGCAAA CTACTC"TTTA TTTTTCCTTA   
  
097091 "ATCGAAAATT CGAGTGGCTG AAGC"TGAAGC AGCTACTTTC GAAGTAACAG AAAGAAAAGC AACGACTGGA   
       TAGCTTTTAA GCTCACCGAC TTCGACTTCG TCGATGAAAG CTTCATTGTC TTTCTTTTCG TTGCTGACCT   
  
097161 GTGGGAGAGT "CAGAGTCGAA AAGAGGATTC CTCACTTCTT TCTCTCATTC AAAACCGTGC ATGAGACTTT "  
       CACCCTCTC"A GTCTCAGCTT TTCTCCTAAG GAGTGAAGAA AGAGAGTAAG TTTTGGCACG TACTCTGAAA "  
  
097231 "CATCTCGCAC" GGCTCCTAAG CTAAGTGATA AAAGAAAGAA GAACTCATCT TCTTTCCTTT TTTGATTACC   
       "GTAGAGCGT"G CCGAGGATTC GATTCACTAT TTTCTTTCTT CTTGAGTAGA AGAAAGGAAA AAACTAATGG   
  
097301 TTCCTCGCGT ATGTATAA"GA CCGAATCCAT TCGATTTCTA AAAAGGATTA CTAATCCTTA ACTTTTCGAG "  
       A"AGGAGCGCA TACATATTCT GGCTTAGGTA AGCTAAAGAT TTTTCCTAAT GATTAGGAAT TGAAAAGCTC "  
  
097371 "GAATCCTTCA TCAGTGGT"TG TGAATGACTG ATTTTTCTCA ATCTTTTCGA CCTTAGTTCC GTAGGAGCAA   
       "C"TTAGGAAGT AGTCACCAAC ACTTACTGAC TAAAAAGAGT TAGAAAAGCT GGAATCAAGG CATCCTCGTT   
  
097441 GTCAGAAAGA TTGAGAAATA GAA"CCATCTG ATTTGATTCG TTCTCAATAG CCATGTGATG ATCATCTTAG "  
       CAGTCTT"TCT AACTCTTTAT CTTGGTAGAC TAAACTAAGC AAGAGTTATC GGTACACTAC TAGTAGAATC "  
  
097511 "GGTGATCCTT TTGTCGACGG ATG"CTCCTAT TACACTCGTA GTCTCTGAAG GATGAGAACC AACTATGTAG   
       "CCACTAG"GAA AACAGCTGCC TACGAGGATA ATGTGAGCAT CAGAGACTTC CTACTCTTGG TTGATACATC   
  
097581 CATCTACATC "GAGAATTCAA GTATTGTATA CGTCATTAGT CCGATCCTTT GTAGGAACTA CCCGTAATAA "  
       GTA"GATGTAG CTCTTAAGTT CATAACATAT GCAGTAATCA GGCTAGGAAA CATCCTTGAT GGGCATTATT "  
  
097651 "CGAACTTGCA" AAATGAATCT GTTTATCATA AAGAGATTCG TTGTTCCTGA CCCTGCTTCA CCTTAATTGT   
       "GCT"TGAACGT TTTACTTAGA CAAATAGTAT TTCTCTAAGC AACAAGGACT GGGACGAAGT GGAATTAACA   
  
097721 TATTTGAACA AGTAAAAGTT ATGTCTT"GGT CCGAGTGGGG ATAGTATTTC TCTTCTGCAT GTCCATGAAG "  
       ATAAACTTGT TCATTTTCAA T"ACAGAACCA GGCTCACCCC TATCATAAAG AGAAGACGTA CAGGTACTTC "  
  
097791 "TTTTGAAAAA TCCAAACATC TCAGAGA"TAG ATAGAGAGGT AGGAATTTAT CGAACGAACC GCACTCCTTC   
       "AAAACTTTTT AGGTTTGTAG A"GTCTCTATC TATCTCTCCA TCCTTAAATA GCTTGCTTGG CGTGAGGAAG   
  
097861 GTATACGTCA "GGAGTCCATT GATGAGAAGG GGCTGGGGAA AGCTTGAACC CAATTCCTAC AGTGATGAAT "  
       CATATGCAGT CCTCAGGTAA CTACTCTTCC CCGACCCCTT TCGAACTTGG GTTAAGGATG TCACTACTTA   
  
097931 "ATAAGCGCAA" TTGAAATTCC TGGGGAGTTA TACATTTGTG TATTGAGAAG ACCATTTACT ATTTCTTGAA   
       TATTCGCGTT AACTTTAAGG ACCCCTCAAT ATGTAAACAC ATAA"CTCTTC TGGTAAATGA TAAAGAACTT "  
  
098001 GCTCGATCTC "TCCCCCGGAT GAACCATATA GCCAAGAGAA ACCATGAACC AGAATAGAAG AACTTGCCCC "  
       "CGAGCTAGAG AGGGGGCCTA CTTGGTATAT CGGTTCTCTT TGGT"ACTTGG TCTTATCTTC TT"GAACGGGG "  
  
098071 "ACCCATGAGT" AAATATTTCG TAGTAGCCTC ATTAGACCGT ACATCTTTCT TGGTATATCC AGATAATAGG   
       "TGGGTACTCA TTTATAAAGC ATCATCGGAG TAATCTGGCA TGTAGAAAGA ACCATATAGG TC"TATTATCC   
  
098141 TAGGAGCATA AA"CTGAAACA TTCTGGAGCT ACAAAGATAG TTATTAAATC ATTAGCACCG CATAAAAACA "  
       ATCCTCGTAT TTGACTTTGT AAGACCTCGA TGTTTCTATC AATAATTTAG TAATCGTGGC GTATTTTTGT   
  
098211 "TTCCTCCTAG AG"TAGCTGTT AATACGAATA ATAGAAACTC TGTTATAGCC ATTTCTGTAC ATTCAATGTA   
       AAGGAGGATC TCATCGACAA TTATGCTTAT TATCTTTGAG ACAATATCGG TAAAGACATG TAAGTTACAT   
  
098281 CTCTACGGAT AGAGGAATAC ATAGA"GTTGA ACATAGTAAA ATAAGAAATT GAAAGATTTC GTTGAAATTG "  
       GAGATGCCTA TCTCCTTATG TATCTCAACT TGTATCATTT TATTCTTTAA CTTT"CTAAAG CAACTTTAAC "  
  
098351 "TTCGTTTGGA AATTTCCCGA AAAGC"TAATC ATAGGTTCTT CTCTCCATCG GAACAATAGG GCCGTTATGC   
       "AAGCAAACCT TTAAAGGGCT TTTCGATTAG TATCCAAGAA GAGAGGTAGC CTTG"TTATCC CGGCAATACG   
  
098421 TCATTACTAA ACTTGTTGAA "GAGATGAAAT ATAACCAAGG TATATCTTTT TGATCAGAGG TTGAATCGAT "  
       AGTAATGATT TGAACAACTT CTCTACTTTA TATTGGTTCC ATAT"AGAAAA ACTAGTCTCC AACTTAGCTA "  
  
098491 "CATCAGAAGA AGAATTAGGC" CAAAAATTAG GATACATTCT GGGAAAATAA AACTTCCATC GAAGAGAAGC   
       "GTAGTCTTCT TCTTAATCCG GTTTTTAATC CTATGTAAGA CCCT"TTTATT TTGAAGGTAG CTTCTCTTCG   
  
098561 AAATGAAAGG CTTTCATAAA "AATTCTCGTA GAATCGAGAA TGAAGTTTTC ATTCTGTACA TGCCAGATCA "  
       TTTACTTTCC GAAAGTATTT TTAAGAGCAT CTTAGCTCTT ACTTCAAAAG TAAGACATGT ACGGTCTAGT   
  
098631 "TGAATTAGTA ACTGCATCCA" ATCTCCAAAA AAATCCCACT TGTTTCGAAC TTTCTCTTTT TGGAATGGAA   
       ACTTAATCAT TGACGTAGGT TAGAGGTTTT TTTAGGGTGA ACAAAGCTTG AAAGAGAAAA ACCTTACCTT   
  
098701 TATTTACGGA ATCCCCATGA ATAGGATCAA "ACCTTATTCC ATGGTATTTA CATGAGATTC CTCTTTCTTA "  
       ATAAATGCCT TAGGGGTACT TATCCTAGTT "TGGAATAAGG TACCATAAAT GTACTCTAAG GAGAAAGAAT "  
  
098771 "TTCTTAAGCA AGTCCCCGAG AGGGCTTGGC" TTAGTTGATC CATGATTTAT GTTTCGTCTT TCGTTTCGTT   
       "AAGAATTCGT TCAGGGGCTC TCCCGAACCG" AATCAACTAG GTACTAAATA CAAAGCAGAA AGCAAAGCAA   
  
098841 TTCGTTTGTT TCGAGAAATA TATC"GATCAA TTCCGATTCT TTCTTTTTCT ATTGATTCTT TTCCGATCGA "  
       AAGCAAACAA AGCTCTTTAT ATAGCTAGTT AAGGCTAAGA AAGAAAAA"GA TAACTAAGAA AAGGCTAGCT "  
  
098911 "GATGTATGGA TCCATGGGCC TATG"TGTCTA TATAGATCCT GTTCATGGAT TAACGAAAAT GTGCAAAAGC   
       "CTACATACCT AGGTACCCGG ATACACAGAT ATATCTAGGA CAAGTACC"TA ATTGCTTTTA CACGTTTTCG   
  
098981 TCTATTTGCC TCTGCCATTC "TATGAGTCTC TTCCTTTTTA CGTATGGCAT CGCCACTCCC TTTGGCAGCA "  
       AGATAAACGG AGACGGTAAG "ATACTCAGAG AAGGAAAAAT GCATACCGTA GCGGTGAGGG AAACCGTCGT "  
  
099051 "TCCACTAATT CGGAACTGAA" TTTGAAAGCC ATATTTCGAC CGGGACGTTT TCGGGATGCC CCTAATAACC   
       "AGGTGATTAA GCCTTGACTT" AAACTTTCGG TATA"AAGCTG GCCCTGCAAA AGCCCTACGG GGATTATTGG "  
  
099121 AACGAATGGC AAGTGCTTTT CCTTGTGTGG "ATCCTATTTC AATGGGAACT TGATGAGTTG ATCCGCCTAC "  
       "TTGCTTACCG TTCACGAAAA GGAACACACC TAGG"ATAAAG TTACCCTTGA ACTACTCAAC TAGGCGGATG   
  
099191 "ACGTCTTGCC TTTACTGCTA TATCGGGAGT" TACTCCACGT ATTGCTTGAC GTAAAACAGA TAGTGGATTT   
       TGCAGAACGG A"AATGACGAT ATAGCCCTCA ATGAGGTGCA TAACGAACTG CATTTTGTCT ATCACCTAAA "  
  
099261 GTTTCTGTCT TTTGTTGAAT CTTTTTCAT"G GCTCGATAGA TAATTTGATA AGCCAATGAT TTTTTTCCAT "  
       "CAAAGACAGA A"AACAACTTA GAAAAAGTAC CGAGCTATCT ATTAAACTAT TCGGTTACTA AAAAAA"GGTA "  
  
099331 "GTTTCAGAAT ACGGTTAACC AACATGTTA"A CTAATCGATT ACGATAAATT GGATCGGATT TTTCAATTTT   
       "CAAAGTCTTA TGCCAATTGG TTGTACAATT GATTAGCTAA TGCTATTTAA CCTAGCCTAA AAAGTT"AAAA   
  
099401 TTCTTCTGCA GTACCTC"GAC GTGACATGAG CGTGAAAGGG GTTCAAGAAT AAGTTTTCTT TTTATAAGGG "  
       AAGAAGACGT CATGGAGCTG CACTGTACTC GCACTTTCCC CAAGTTCTTA TTCAAAAGAA AAATATTCCC   
  
099471 "CTAAAATCAT TTATTTT"GGC TTTTTGACCC CATATTGTAG GGTGGATCTC GAAAGATATG AAAGATCTCC   
       GATTTTAGTA "AATAAAACCG AAAAACTGGG GTATAA"CATC CCACCTAGAG CTTTCTATAC TTTCTAGAGG   
  
099541 CTCCAAGCCG TACA"TACGAC TTTCATCGAA TACGGCTTTC CACAGAATTC TATATGTATC TATGAGATCG "  
       GAGGTTCG"GC ATGTATGCTG AAAGTAGCTT ATGCCGAAAG GTGTCTTAAG ATATACATAG ATACTCTAGC "  
  
099611 "AGTATGGAAT TCTG"TTTACT CACTTTAAAT TGAGTATCCG TTTCCCTCCC TTTCCTGCTA GGATTGGAAA   
       "TCATACCT"TA AGACAAATGA GTGAAATTTA ACTCATAGGC AAAGGGAGGG AAAGGACGAT CCTAACCTTT   
  
099681 TCCTGTATTT TACATATCCA TACGAT"TGAG TCCTTGGGTT TCCGAAATAG TGTAAAAAGA GGTGCTTCGA "  
       AGGACATA"AA ATGTATAGGT ATGCTAACTC AGGAACCCAA AGGCTTTATC ACATTTTTCT CCACGAAGCT "  
  
099751 "ATCATTACTA TTTGACTCGG ACCTGT"TCTA AAAAAGTCGA GGCATTTCGA ATTGTTTGTT GACACGGACA   
       "TAGTAATG"AT AAACTGAGCC TGGACAAGAT TTTTTCAGCT CCGTAAAGCT TAACAAACAA CTGTG"CCTGT "  
  
099821 AAGTCAAGGA AAACCTCTGA AATTAT"TTCA ATATTGGACC TTGGACATAT AATAGTTCCG AATCGAATCT "  
       "TTCAGTTCCT TTTGGAGACT TTAATAAAGT TATAACCTGG AACCTGTATA TTATCAAGGC TTAGC"TTAGA   
  
099891 "CTTTAGAAAG AAGATCTTTT GTTTGT"CTCA TGGTAGCCTG CTCCAGTTCC CTTACGAAAC TTTCGTTATT   
       GAAATCTTTC TTCTAGAAAA CAAACAGAGT ACCATCGGAC GAGGTCAAGG GAATGCTTTG AAAG"CAATAA "  
  
099961 GGGTTAGCCA TACACTTCAC ATGTTTC"TAG CGATTCACAT GGCATCATCA AATGATACAA GTCTTGGATA "  
       "CCCAATCGGT ATGTGAAGTG TACAAAGATC GCTAAGTGTA CCGTAGTAGT TTACTATGTT CAGA"ACCTAT   
  
100031 "AGAATCTACA ACGCACTAGA ACGCCCT"TGT TGACGATCCT TTACTCCGAC AGCATCTAGG GTTCCTCGAA   
       TCTTAGATGT TGCGTGATCT TGCGGGAACA ACTGCTAGGA AATGAGG"CTG TCGTAGATCC CAAGGAGCTT "  
  
100101 CAATGTGATA TCTCACACCG "GGTAAATCCT TAACCCTTCC CCCTCTTACT AAGACTACAG AATGTTCTTG "  
       "GTTACACTAT AGAGTGTGGC CCATTTAGGA ATTGGGAAGG GGGAGAA"TGA TTCTGATGTC TTACAAGAAC   
  
100171 "TAAATTATGG CCAATACCAG" GTATATAAGC AGTGATTTCA AATCCCGAGG TTAAGCGTAC TCTGGCAACT   
       ATTTAATACC GGTTATGGT"C CATATATTCG TCACTAAAGT TTAGGGCTCC AATTCGCATG AGACCGTTGA "  
  
100241 TTACGTAAGG CA"GAGTTTGG TTTTTTGGGG GTGATAGTGG AAAAGTTGAC AGATAAGTCA CCCTTACTGC "  
       "AATGCATTCC GTCTCAAAC"C AAAAAACCCC CACTATCACC TTTTCAACTG TCTATT"CAGT GGGAATGACG "  
  
100311 "CACTCGACAG AA"CCGTACGT GAGATTTTCA CCTCATACGG CTCCTCGTTC AATTCTTTCG AAGTCATTGG   
       "GTGAGCTGTC TTGGCATGCA CTCTAAAAGT GGAGTATGCC GAGGAGCAAG TTAAGA"AAGC TTCAGTAACC   
  
100381 ATCCTTTTCC TCG"TTCGAGA ATCTCCTCCC TTCTTCCATT CCGTCCCGAA GAGTGACCGG GACCAATTCA "  
       TAGGAAAAGG AGCAAGCTCT TAGAGGAGGG AAGAAGGTAA GGCAGG"GCTT CTCACTGGCC CTGGTTAAGT "  
  
100451 "GTCACGTTTT CAT"TCATTTG GAATCTGGGC TCTTCTACGT CATTTTTATT TACTTATTTT TCCCTCTCTT   
       "CAGTGCAAAA GTAAGTAAAC CTTAGACCCG AGAAGATGCA GTAAAA"ATAA ATGAATAAAA AGGGAGAGAA   
  
100521 TTTTTTTTTT TCTTTTTTTA T"TCCCTTCGA TCATTCCTTA AGTCCCATAG GTTTGATCCT GTAGAATCTG "  
       AAAAAAAAAA AGAAAAAAAT AAGGGAAGCT AGTAAGGAAT T"CAGGGTATC CAAACTAGGA CATCTTAGAC "  
  
100591 "ACCCATTTTT TCATTGAGCG A"AAGGTACGA AATAAATCAG ATTGATTTTT CGATCAAAAG TACTATGTGA   
       "TGGGTAAAAA AGTAACTCGC TTTCCATGCT TTATTTAGTC T"AACTAAAAA GCTAGTTTTC ATGATACACT   
  
100661 AATCTTCGTT TTTTTCCTCT "TTCTCTATCC CTATCCCGCG GGTACAGTGT TTGAATCAAT AGAGAACCTT "  
       TTAGAAGCAA AAAAAGGAGA AAGAGATAGG GATAGGGCGC CCATGTCACA AA"CTTAGTTA TCTCTTGGAA "  
  
100731 "TTCTTCTGTA TCTGTATGAA" TCGATATTAT TACATTCCAA TTCCTTCCCG ATACCTAAAA TTCCGAATTG   
       "AAGAAGACAT AGACATACTT AGCTATAATA ATGTAAGGTT AAGGAAGGGC TA"TGGATTTT AAGGCTTAAC   
  
100801 GATCCAAAAT TGACG"GGTTA GTGTGAGCTT ATCCATGCGG TTATGCACTC TTCGAATAGG AATCCATTTT "  
       CTAGGTTTTA ACTGCCCAAT CACACTCGAA TAGGTACGCC AATACGTGA"G AAGCTTATCC TTAGGTAAAA "  
  
100871 "CTGAAAGATT CTGGC"TTTCG TGCTTTGGTG GGTCTCCGAG ATCCTTTCGA TGACCTATGT TGTGTTGAAG   
       "GACTTTCTAA GACCGAAAGC ACGAAACCAC CCAGAGGCTC TAGGAAAGC"T ACTGGATACA ACACAACTTC   
  
100941 GGATATCTAT ATGAT"CCGAT TGATTGCGTA AAGCCCGCGG TAGCAACGGA ACCGGGGAAA GTATACAGAA "  
       CCTATAGATA TACTAGGCTA ACTAACGCAT TTCGGGCGCC AT"CGTTGCCT TGGCCCCTTT CATATGTCTT "  
  
101011 "AAGACAGTTC TTTTC"TATTC TATTAGTATT AGATTAGTAT TAGTTAGTGA TCTCGGCTCA GTGAGTCCTT   
       "TTCTGTCAAG AAAAGATAAG ATAATCATAA TCTAATCATA AT"CAATCACT AGAGCCGAGT CACTCAGGAA   
  
101081 TCTTCCGTGA T"GAACTGTTG GCACCAGTCC TCCATTTTTT CTCTGTGGAT CGAGGAGAAA GGGGGCTCGG "  
       AGAAGGCACT ACTTGACAAC CGTGGTCAGG AGGTAAAAAA GAGACACCTA GCTCCTCTT"T CCCCCGAGCC "  
  
101151 "CGGGAAGAGG A"TTGTAACAT GAGAGAAGCA AGGAGGTCAA CCTCTTTCAA ATATACAACA TGGATTCTGG   
       "GCCCTTCTCC TAACATTGTA CTCTCTTCGT TCCTCCAGTT GGAGAAAGTT TATATGTTG"T ACCTAAGACC   
  
101221 CAATGCAATG TAGTTGGACT CTCATGT"CGA TCCGAATGAA TCATCCTTTC CACGGAGGTA AATCTTTGCC "  
       GTTACGTTAC ATCAACCTGA GAGTACAGCT AGGCTTACTT AGTAGGAAAG "GTGCCTCCAT TTAGAAACGG "  
  
101291 "TGCTAGGCAA GAGGATAGCA AGTTACA"AAT TCTGTCGCGG TAGGACATGT ATTTCTATTA CTATGAAATT   
       "ACGATCCGTT CTCCTATCGT TCAATGTTTA AGACAGCGCC ATCCTGTACA" TAAAGATAAT GATACTTTAA   
  
101361 CATAAATGAA G"TAGTTAATG GTGGGGTTAC CATTATCCTT TTTGCAATGA CGAATCTTGT ATGTGTTCCG "  
       GTATTTACTT CATCAATTAC CACCCCAATG GTAATAGGAA AAACGTT"ACT GCTTAGAACA TACACAAGGC "  
  
101431 "AAGAAAAGTT G"TTCATTTTT CGGGGTCTCG AAGGGGCGTG GAAACACATA AAAACTCTGG AATGGAAATG   
       "TTCTTTTCAA CAAGTAAAAA GCCCCAGAGC TTCCCCGCAC CTTTGTG"TAT TTTTGAGACC TTACCTTTAC   
  
101501 GAAAAGAGAT GTAACTCCAG TTCC"TTCGGA AATGGTAAGA TCTTTGGCAC AAGAAGAAGG GGTTGATCCA "  
       CTTTTCTCTA CATTGAGGTC AAGGAAGCCT TTACCATTCT AGAAA"CCGTG TTCTTCTTCC CCAACTAGGT "  
  
101571 "TATCATCTTG ACTGGGTTCT GATT"TCTCTA TTTTTTTAAT AATACCGAGT TGGGTTCTTT TCCTACCCGT   
       "ATAGTAGAAC TGACCCAAGA CTAAAGAGAT AAAAAAATTA TTATG"GCTCA ACCCAAGAAA AGGATGGGCA   
  
101641 ATCGAATAGA ACATGCTGAG CCAAATCTTC "TTCATGTAAA ACCTGCTTTA TTTAGATCGG GAAAATCGTA "  
       TAGCTTATCT TGTACGACTC GGTTTAGAAG AAGTACATTT TGGACGAAAT AAATCTAGC"C CTTTTAGCAT "  
  
101711 "CGGTTTTATG AAACCGTGTG CTATGGCTCG" AATCCGTAGT CAATCCTATT TACGATAGGA GCTGTTGACA   
       "GCCAAAATAC TTTGGCACAC GATACCGAGC TTAGGCATCA GTTAGGATAA ATGCTATCC"T CGACAACTGT   
  
101781 ATTGAATACA ATTTTGCCCA "TTATTTTCAT TTTCGTATCC GTACGAAAAG AAGGAAGGCC CAATTCCAAG "  
       TAACTTATGT TAAAACGGGT AATAAAAGTA AAAGCATAGG CATGCTTTTC T"TCCTTCCGG GTTAAGGTTC "  
  
101851 "TTGTTCAAGA ATAGTGGCGT" TGAGTTTCTC GACCCTTTGC CTTAGGATTA GTCAGTTCTA TTTCTCGATG   
       "AACAAGTTCT TATCACCGCA ACTCAAAGAG CTGGGAAACG GAATCCTAAT C"AGTCAAGAT AAAGAGCTAC   
  
101921 GGGGCAAGGG A"AGGGATATA ACTCAGCGGT AGAGTGTCGC CTTGACGTGG CGGAAGTCAT CAGTTCGAGC "  
       CCCCGTTCCC TTCCCTATAT TGAGTCGCCA TCTCACAGCG GAACTGCACC G"CCTTCAGTA GTCAAGCTCG "  
  
101991 "CTGATTATCC C"TA"AACCCAA TGTGAGTTTT TCTATTTTGA CTTGCTCCCC CGCCGTGATC GAATGAGAAT "  
       "GACTAATAGG GATTTGGGTT ACACTCAAAA AGATAAAACT GAACGAGGGG G"CGGCACTAG CTTACTCTTA   
  
102061 "GGATAAGAGG CTC"GTGGGAT TGACGTGAGG GGGTGGGGAT GGCTATATTT CTGGGAGCGA ACTCCAGGCG   
       CCTATTCTCC GAGCACCCTA ACTGCACTCC CCCACCCCTA CC"GATATAAA GACCCTCGCT TGAGGTCCGC "  
  
102131 AATAT"GAAGC GCATGGATAC AAGTTATGCC TTGGAATGAA AGACAATTCC GAATTAGCTT TGTCTACGAA "  
       "TTATACTTCG CGTACCTATG TTCAATACGG AACCTTACTT TC"TGTTAAGG CTTAATCGAA ACAGATGCTT   
  
102201 "CAAGG"AAGCT ATAAGTAATG CAACTATGAA "TCTCATGGAG AGTTCGATCC TGGCTCAGGA TGAACGCTGG "  
       GTTCCTTCGA TATTCATTAC GTTGATACTT AGAGTACCTC TCAAGCT"AGG ACCGAGTCCT ACTTGCGACC "  
  
102271 "CGGCATGCTT AACACATGCA AGTCGGACGG" GAAGTGGTGT TTCCAGTGGC GGACGGGTGA GTAACGCGTA   
       "GCCGTACGAA TTGTGTACGT TCAGCCTGCC CTTCACCACA AAGGTCA"CCG CCTGCCCACT CATTGCGCAT   
  
102341 AGAACCTGCC CTTGGGAGGG GAACAACAGC TGGAAACGGC TGCTAATACC CCGTACGCTG AGGAGCAAAA   
       TCTTGGACGG GAACCCTCCC CTTGTTGTCG ACCTTTGCCG ACGATTATGG GGCATGCGAC "TCCTCGTTTT "  
  
102411 GGAGGAATCC GCCCGAGGAG GGGCTCGCGT CTGATTAGCT AGTTGGTGAG GCAATAGCTT ACCAAGGCGA   
       "CCTCCTTAGG CGGGCTCCTC CCCGAGCGCA GACTAATCGA TCAACCACTC CGTTATCGAA" TGGTTCCGCT   
  
102481 TGATCAGTAG CTGGTCCGAG AGGATGATCA GCCACACTGG GACTGAGACA CGGCCCAGAC TCCTACGGGA   
       ACTAGTCATC GACCAGGCTC TCCTACTAGT CGGTGTGACC CTGACTCTGT GC"CGGGTCTG AGGATGCCCT "  
  
102551 GGCAGCAGTG GGGAATTTTC CGCAATGGGC GAAAGCCTGA CGGAGCAATG CCGCGTGGAG GTAGA"AGGCC "  
       "CCGTCGTCAC CCCTTAAAAG GCGTTACCCG CTTTCGGACT GCCTCGTTAC GG"CGCACCTC CATCTTCCGG   
  
102621 "TACGGGTCGT GAACTTCTTT TCCCGGAGAA GAAGCAATGA CGGTATCTGG GGAATAAGCA TCGGC"TAACT   
       ATGCCCAGCA CTTGAAGAAA AGGGCCTCTT CTTCGTTACT GCCATAGACC CCT"TATTCGT AGCCGATTGA "  
  
102691 CTGTGCCAGC AGCCGCGGTA ATACAGAGGA TGCAAGCGTT ATCCGGAATG ATTGGGCGTA AAGCGTCTGT   
       "GACACGGTCG TCGGCGCCAT TATGTCTCCT ACGTTCGCAA TAGGCCTTAC TAA"CCCGCAT TTCGCAGACA   
  
102761 AGGTGGCTTT TTAAGTCCGC CGTCAAATCC CAGGGCTCAA CCCTGGACAG GCGGTGGAAA CTACCAAGCT   
       TCCACCGAAA AATTCAGGCG GCAGTTTAGG GTCCCGAGTT GGGACCTGTC CGCCAC"CTTT GATGGTTCGA "  
  
102831 GGAGTACGGT AGGGGCAGAG GGAATTTCCG GTGGAGCGGT GAAATGCGTA GAGATCGGAA AGAACACCAA   
       "CCTCATGCCA TCCCCGTCTC CCTTAAAGGC CACCTCGCCA CTTTACGCAT CTCTAG"CCTT TCTTGTGGTT   
  
102901 CGGCGAAAGC ACTCTGCTGG GCCGACACTG ACACTGAGAG ACGAAAGCTA GGGGAGCGAA TGGGATTAGA   
       GCCGCTTTCG TGAGACGACC CGGCTGTGAC TGTGACTCTC TGCTTTCGAT "CCCCTCGCTT ACCCTAATCT "  
  
102971 TACCCC"AGTA GTCCTAGCCG TAAACGATGG ATACTAGGCG CTGTGCGTAT CGACCCGTGC AGTGCTGTAG "  
       "ATGGGGTCAT CAGGATCGGC ATTTGCTACC TATGATCCGC GACACGCATA" GCTGGGCACG TCACGACATC   
  
103041 "CTAACG"CGTT AAGTATCCCG CCTGGGGAGT ACGTTCGCAA GAATGAAACT CAAAGGAATT GACGGGGGCC   
       GATTGCGCAA TTCATAGGGC GGACCCCTCA TGCAAGCGTT CTTACTTTGA GTTT"CCTTAA CTGCCCCCGG "  
  
103111 CGCACAAGCG GTGGAGCATG TGGTTTAATT CGATGCAAAG CGAAGAACCT TACCAGGGCT TGACATGCCG   
       "GCGTGTTCGC CACCTCGTAC ACCAAATTAA GCTACGTTTC GCTTCTTGGA ATGG"TCCCGA ACTGTACGGC   
  
103181 CGAATCCTCT TGAAAGAGAG GGGTGCCTTC GGGAACGCGG ACACAGGTGG TGCATGGCTG TCGTCAGCTC   
       GCTTAGGAGA ACTTTCTCTC CCCACGGAAG CCCTTGCGCC TGTGTCCACC ACGTACCGAC "AGCAGTCGAG "  
  
103251 GTGCCGTAAG GTGTTGGGTT AAGTCCCGCA ACGAGCGCAA CCCTCGTGTT TAGTTGCCAA CCGTTGAGGT   
       "CACGGCATTC CACAACCCAA TTCAGGGCGT TGCTCGCGTT GGGAGCACAA ATCAACGGTT" GGCAACTCCA   
  
103321 TGGAACCCTG AGCAGACTGC CGGTGATAAG CCGGAGGAAG G"TGAGGATGA CGTCAAGTCA TCATGCCCCT "  
       ACCTTGGGAC TCGTCTGACG GCCACTATTC GGCCTCCTTC C"ACTCCTACT GCAGTTCAGT AGTACGGGGA "  
  
103391 "TATGCCCTGG GCGACACACG TGCTACAATG GCCGGGACAA A"GGGTCGTGA TCCCGCGAGG GTGAGCTAAC   
       "ATACGGGACC CGCTGTGTGC ACGATGTTAC CGGCCCTGTT T"CCCAGCACT AGGGCGCTCC CACTCGATTG   
  
103461 TCCAAAAACC CGTCCTCAGT TCGGATTGTA GGCTGCAACT CGCCTGCATG AAGCCGGAAT CGCTAGTAAT   
       AGGTTTTTGG GCAGGAGTCA AGCCTAACAT CCGACGTTGA GCGGACGTAC "TTCGGCCTTA GCGATCATTA "  
  
103531 CGCCGGTCAG CCATACGGCG GTGAATTCGT TCCCGGGCCT TGTACACACC GCCCGTCACA CTATGGGAGC   
       "GCGGCCAGTC GGTATGCCGC CACTTAAGCA AGGGCCCGGA ACATGTGTGG" CGGGCAGTGT GATACCCTCG   
  
103601 TGGCCATGCC CGAAGTCGTT ACCTTAACCG CAAGGAGGGG GATGCCGAAG GCAGGGCTAG TGACTGGAGT   
       ACCGGTACGG GCTTCAGCAA TGGAATTGGC GTTCCTCCCC CTACGGCTTC C"GTCCCGATC ACTGACCTCA "  
  
103671 GAAGTCGTAA CAAGGTAGCC GTACTGGAAG GTGCGGCTGG ATCACCTCCT TTTCAGG"GAG AGCTAATGCT "  
       "CTTCAGCATT GTTCCATCGG CATGACCTTC CACGCCGACC TAGTGGAGGA A"AAGTCCCTC TCGATTACGA   
  
103741 "TGTTGGGTAT TTTGGTTTGA CACTGCTTCA CACCCAAAAA GAAGCGAGCT ACGTCTG"AGT TAAACTTGGA   
       ACAACCCATA AAACCAAACT GTGACGAAGT GTGGGTTTTT CT"TCGCTCGA TGCAGACTCA ATTTGAACCT "  
  
103811 GATGGAAGTC TTCTTTCGTT TCTCGACGGT GAAGTAAGAC TAAGCTCATG AGCTTATTA"T CCTAGGTCGG "  
       "CTACCTTCAG AAGAAAGCAA AGAGCTGCCA CTTCATTCTG AT"TCGAGTAC TCGAATAATA GGATCCAGCC   
  
103881 "AACAAGTTGA TAGGATCCCC TTTTTTACGT CCCCGTGTCC CTCCCGTGTG GCGACATGG"G GGCGAAAAAA   
       TTGTTCAACT ATCCTAGGGG AAAAAATGCA GGGGCACAGG G"AGGGCACAC CGCTGTACCC CCGCTTTTTT "  
  
103951 GGAAAGAGAG GGATGGGGTT TCTCTCGCTT TTGGCATAGC GGGCCCCCAG CGGGAGGCCC GCACGAC"GGG "  
       "CCTTTCTCTC CCTACCCCAA AGAGAGCGAA AACCGTATCG C"CCGGGGGTC GCCCTCCGGG CGTGCTGCCC   
  
104021 "CTATTAGCTC AGTGGTAGAG CGCGCCCCTG ATAA"TTGCGT CGTTGTGCCT "GGGCTGTGAG GGCTCTCAGC "  
       GATAATCGAG TCACCATCTC GCGCGGGGAC TATTAACGCA GCAACACGGA C"CCGACACTC CCGAGAGTCG "  
  
104091 "CACATGGATA GTTCAATGTG CTCATCAGCG CCTGACCCTG AGATGTGGAT" CATCCAAGGC ACATTAGCAT   
       "GTGTACCTAT CAAGTTACAC GAGTAGTCGC GGACTGGGAC TCTACACCTA G"TAGGTTCCG TGTAATCGTA   
  
104161 GGCGTACTTC TCCTGTTTGA ACCGGGGTTT GAAACCAAAC TTCTCCT"CAG GAGGATAGAT GGGGCGATTC "  
       CCGCATGAAG AGGACAAACT TGGCCCCAAA CTTTGGTTTG AAGAGGA"GTC CTCCTATCTA CCCCGCTAAG "  
  
104231 "AGGTGAGATC CAATGTAGAT CCAACTTTCT ATTCACTCGT GGGATCC"GGG CGGTCCGGGG GGGACCACCT   
       "TCCACTCTAG GTTACATCTA GGTTGAAAGA TAAGTGAGCA CCCTAGG"CCC GCCAGGCCCC CCCTGGTGGA   
  
104301 CGGCTCCTCT CTTCTCGAGA ATCCATACAT CCCTTATCAG TATATGGA"CA GCTATCTCTC GAGCACAGGT "  
       GCCGAGGAGA GAAGAGCTCT TAGGTATGTA GGGAATAGTC ATATACCT"GT CGATAGAGAG CTCGTGTCCA "  
  
104371 "TTAGGTTCGG CCTCAATGGG AAAATAAAAC GGAGCACCTA ACAACGTA"TC TTCACAGACC AAGAACTACG   
       "AATCCAAGCC GGAGTTACCC TTTTATTTTG CCTCGTGGAT TGTTGCAT"AG AAGTGTCTGG TTCTTGATGC   
  
104441 AGATCGCCCC TTTCATTCTG GGGTGACGGA "GGGATCGTAC CATTCGAGCC TTTTTTTTTC ATGCTTTTCC "  
       TCTAGCGGGG AAAGTAAGAC CCCACTGCCT CCCTAGCATG GTAA"GCTCGG AAAAAAAAAG TACGAAAAGG "  
  
104511 "CGGAGGTCTG GAGAAAGCTG CAATCAATAG" GATTTTCTTA ATCCTCCCTT CCCGAAAGCG AAAGGAAGAA   
       "GCCTCCAGAC CTCTTTCGAC GTTAGTTATC CTAAAAGAAT TAGG"AGGGAA GGGCTTTCGC TTTCCTTCTT   
  
104581 CGTGAAATTC TTTTTCCTTT CTTCCGCAGG GA"CCAGGAGA TTTGAAAAAG GATCTTAGAG TGTCTAGGGT "  
       GCACTTTAAG AAAAAGGAAA GAAGGCGTCC CTGGTCCTCT AAACTTTTTC "CTAGAATCTC ACAGATCCCA "  
  
104651 "TGGGCCAGGA GGGTTTCTTA ACGCCTTCTT TT"TTCTTCTC ATCGGAGTTA TTTCACAAAG ACTTGCCATG   
       "ACCCGGTCCT CCCAAAGAAT TGCGGAAGAA AAAAGAAGAG TAGCCTCAAT" AAAGTGTTTC TGAACGGTAC   
  
104721 GTAAGGAAGA AGGGGGGAAC AAGCACACTT GGAGAGCGCA GTA"CAACGGA GAGTTGTATG CTGCGTTCGG "  
       CATTCCTTCT TCCCCCCTTG TTCGTGTGAA CCTCTCGCGT CATG"TTGCCT CTCAACATAC GACGCAAGCC "  
  
104791 "GAAGGATGAA TCGCTCCCGA AAAGGAATCT ATTGATTCTC TCC"CAATTGG TTGGACCGTA GGTGCGATGA   
       "CTTCCTACTT AGCGAGGGCT TTTCCTTAGA TAACTAAGAG AGGG"TTAACC AACCTGGCAT CCACGCTACT   
  
104861 TTTACTTCAC "GGGCGAGGTC TCTGGTTCAA GTCCAGGATG GCCCA"GCT"AC GCCAAGGAAA AGAATAAAAG "  
       AAATGAAGTG CCCGCTCCAG AGACCAAGTT CAGGTCCTAC CGGGTCGAT"G CGGTTCCTTT TCTTATTTTC "  
  
104931 "AATAGAAGAA GCATCTGACT CCTTCATGCA GGCCCCACTT GGGCTCGG""GG GGATATAGCT CAGTTGGTAG "  
       "TTATCTTCTT CGTAGACTGA GGAAGTACGT CCGGGGTGAA CCCGAGCCCC" CCTATATCGA GTCAACCATC   
  
105001 "AGCTCCGCTC TTGCAA"TTGG GTCGTTGCGA TTACGGGTTG GATGTCTA"AT TGTCCAGGCG GTAATGATAG "  
       TCGAGGCGAG AACGTTAACC CAGCAACGCT AATGCCCAAC CTACAGATTA A"CAGGTCCGC CATTACTATC "  
  
105071 "TATCTTGTAC CTGAACCGGT GGCTCACTTT TTCTAAGTAA TGGGGAAG"AG GACCGAAACA TGCCACTGAA   
       "ATAGAACATG GACTTGGCCA CCGAGTGAAA AAGATTCATT ACCCCTTCTC C"TGGCTTTGT ACGGTGACTT   
  
105141 AGACTCTACT GAGACAAAGA TGGGCTGTCA AGAACGTAGA GG"AGGTAGGA TGGGCAGTTG GTCAGATCTA "  
       TCTGAGATGA CTCTGTTTCT ACCCGACAGT TCTTGCATCT CCTCCATCCT A"CCCGTCAAC CAGTCTAGAT "  
  
105211 "GTATGGATCG TACATGGACG GTAGTTGGAG TCGGCGGCTC TC"TTAGGGTT CCCTCATCTG GGATCCCTGG   
       "CATACCTAGC ATGTACCTGC CATCAACCTC AGCCGCCGAG AGAATCCCAA G"GGAGTAGAC CCTAGGGACC   
  
105281 GGAAGAGGAT CAAGTTGGCC CTTGCGAACA GCTTGATGCA CTATCT"CCCT TCAACCCTTT GAGCGAAATG "  
       CCTTCTCCTA GTTCAACCGG GAACGCTTGT CGAACTACGT GATAGAGGGA "AGTTGGGAAA CTCGCTTTAC "  
  
105351 "CGGCAAAAGG AAGGAAAATC CATGGACCGA CCCCATCGTC TCCACC"CCGT AGGAACTACG AGATCACCCC   
       "GCCGTTTTCC TTCCTTTTAG GTACCTGGCT GGGGTAGCAG AGGTGGGGCA" TCCTTGATGC TCTAGTGGGG   
  
105421 AAGGACGCCT TCGGTATCCA GGGGTCGCGG ACCGAC"CATA GAACCCTGTT CAATAAGTGG AACGCATTAG "  
       TTCCTGCGGA AGCCATAGGT CCCCAGCGCC TGGCTGGTAT CTTGGGACAA GTTA"TTCACC TTGCGTAATC "  
  
105491 "CTGTCCGCTC TCCGGTTGGG CAGTAAGGGT CGGAGA"AGGG CAATCACTCA TTCTTAAAAC CAGCATTCTT   
       "GACAGGCGAG AGGCCAACCC GTCATTCCCA GCCTCTTCCC GTTAGTGAGT AAGA"ATTTTG GTCGTAAGAA   
  
105561 AAGACCAAAG AGGCGGGCGG AAAAGGGGGG AAAGCTCT"CC GTTCCTGGTT CTCCTGTAGC TGGATTCTCC "  
       TTCTGGTTTC TCCGCCCGCC TTTTCCCCCC TTTCGAGAGG CAAGGA"CCAA GAGGACATCG ACCTAAGAGG "  
  
105631 "GGAACCACAA GAATCCTTAG TTAGAATGGG ATTCCAAC"TC AGTACCTTTT GAGATTTTGA GAAGAGTTGC   
       "CCTTGGTGTT CTTAGGAATC AATCTTACCC TAAGGTTGAG TCATGG"AAAA CTCTAAAACT CTTCTCAACG   
  
105701 TCTTTGGAGA GCACAGTACG ATGAAAGTTG TAAGCTGTGT TCGGGG"GGGA GTTATTGTCT ATCGTTGGCC "  
       AGAAACCTCT CGTGTCATGC TACTTTCAAC ATTCGACACA AGCCCCCCCT "CAATAACAGA TAGCAACCGG "  
  
105771 "TCTATGGTAG AATCAGTCGG GGGCCTGAGA GGCGGTGGTT TACCCT"GT"GG CGGATGTCAG CGGTTCGAGT "  
       "AGATACCATC TTAGTCAGCC CCCGGACTCT CCGCCACCAA ATGGGACACC" GCCTACAGTC GCCAAGCTCA   
  
105841 "CCGCTTATCT CCA"A"CTCGTG AACTTAGCCG ATACAAAGCT ATATGATAGC ACTCCATTTT TCCGATTCGG "  
       GGCGAATAGA GGTTGAGCAC TTGAATCGGC TATGTTTCGA TATACTATCG "TGAGGTAAAA AGGCTAAGCC "  
  
105911 "CAGTTCGATC TATG"ATTTAT CATTCATGGA CGTTGATAAG ATCCTTCCAT TTAGCAGCAC CTTAGGATGG   
       "GTCAAGCTAG ATACTAAATA GTAAGTACCT GCAACTATTC TAGGAAGGTA" AATCGTCGTG GAATCCTACC   
  
105981 CATAGCCTTA AAGTTAAGGG CGAGG"TTCAA ACGAAGAAAG GCTTACGGTG GATACCTAGG CACCCAGAGA "  
       GTATCGGAAT TTCAATTCCC GCTCCAAGTT TGCTTCTTTC CGAATGC"CAC CTATGGATCC GTGGGTCTCT "  
  
106051 "CGAGGAAGGG CGTAGTAAGC GACGA"AATGC TTCGGGGAGT TGCAAATAAG CGTAGATCCG GAGATTCCCG   
       "GCTCCTTCCC GCATCATTCG CTGCTTTACG AAGCCCCTCA ACGTTTA"TTC GCATCTAGGC CTCTAAGGGC   
  
106121 AATAGGTCAA CCTTTCAAAC TGCTGCTGAA TCCATGGGCA GGCAAGAGAC AACCTGGCGA ACTGAAACAT   
       TTATCCAGTT GGAAAGTTTG ACGACGACTT AGGTACCCGT CCGTTCTCTG "TTGGACCGCT TGACTTTGTA "  
  
106191 CTTAGTAGCC AGAGGAAAAG AAAGCAAAAG CGATTCCCGT AGTAGCGGCG AGCGAAATGG GAGCAGCCTA   
       "GAATCATCGG TCTCCTTTTC TTTCGTTTTC GCTAAGGGCA TCATCGCCGC" TCGCTTTACC CTCGTCGGAT   
  
106261 AACCGTGAAA ACGGGGTTGT GGGAGAGCAA TACAAGCGTC GTGCTGCTAG GCGAAGCAGT GGAGTGTTGC   
       TTGGCACTTT TGCCCCAACA CCCTCTCGTT ATGTTCGCAG CACGACGA"TC CGCTTCGTCA CCTCACAACG "  
  
106331 ACCCTAGATG GCGAGAGTCC AGTAGCCGAA AGCATCACTA GCTTACGCTC TGACCCGAGT AGCATGGGGC   
       "TGGGATCTAC CGCTCTCAGG TCATCGGCTT TCGTAGTGAT CGAATGCG"AG ACTGGGCTCA TCGTACCCCG   
  
106401 ACGTGGAATC CCGTGTGAAT CAGCAAGGAC CACCTTGCAA GGCTAAATAC TCCTGGGTGA CCGATAGTGA   
       TGCACCTTAG GGCACACTTA GTCGTTCCTG GTGGAACGT"T CCGATTTATG AGGACCCACT GGCTATCACT "  
  
106471 AGTAGTACCG TGAGGGAAGG GTGAAAAGAA CCCCCATCGG GGAGTGAAAT AGAACATGAA ACCGTAAGCT   
       "TCATCATGGC ACTCCCTTCC CACTTTTCTT GGGGGTAGC"C CCTCACTTTA TCTTGTACTT TGGCATTCGA   
  
106541 TCCAAGCAGT GGGAGGAGAC GAGGACTCTG ACCGCGTGCC TGTTGAAGAA TGAGCCGGCG ACTCATAGGC   
       AGGTTCGTCA CCCTCCTCTG CTCCTGAGAC TGGCGCACGG "ACAACTTCTT ACTCGGCCGC TGAGTATCCG "  
  
106611 AGTGGCTTGG TTAAGGGAAC CCACCGGAGC CGTAGCGAAA GCGAGTCTTC ATGGGGCAAT TGTCACTGCT   
       "TCACCGAACC AATTCCCTTG GGTGGCCTCG GCATCGCTTT" CGCTCAGAAG TACCCCGTTA ACAGTGACGA   
  
106681 TATGGACCCG AACCTGGGTG ATCTATCCAT GACCAG"GATG AAGCTTGGGT GAAACTAAGT GGAGGTCCGA "  
       ATACCTGGGC TTGGACCCAC TAGATAGGTA CTGGTCCTAC TTCGAACC"CA CTTTGATTCA CCTCCAGGCT "  
  
106751 "ACCGACTGAT GTTGAAGAAT CAGCGGATGA GTTGTG"GTTA GGGGTGAAAT GCCACTCGAA CCCAGAGCTA   
       "TGGCTGACTA CAACTTCTTA GTCGCCTACT CAACACCAAT CCCCACTT"TA CGGTGAGCTT GGGTCTCGAT   
  
106821 GCTGGTTCTC CCCGAAATGC GTTGAGGCGC AGCAGTTGAC TGGACATCTA GGGGTAAAGC ACTGTTTCGG   
       CGACCAAGAG GGGCTTTACG CAACTCCGCG TCGTCAACTG ACCTGT"AGAT CCCCATTTCG TGACAAAGCC "  
  
106891 TGCGGGCCGC GAGAGCGGTA CCAAATCGAG GCAAACTCTG AATACTAGAT ATGATCTCAA AATAACAGGG   
       "ACGCCCGGCG CTCTCGCCAT GGTTTAGCTC CGTTTGAGAC TTATGA"TCTA TACTAGAGTT TTATTGTCCC   
  
106961 GTCAAGGTCG GCCAGTGAGA CGATGGGGGA TAAGCTTCAT CGTCGAGAGG GAAACAGCCC GGATCACCAG   
       CAGTTCCAGC CGGTCACTCT GCTACCCCCT ATTCGAAGTA GCAGCTCTCC C"TTTGTCGGG CCTAGTGGTC "  
  
107031 CTAAGGCCCC TAAATGACCG CTCAGTGATA AAGGAGGTAG GGGTGCAGAG ACAGCCAGGA GGTTTGCCTA   
       "GATTCCGGGG ATTTACTGGC GAGTCACTAT TTCCTCCATC CCCACGTCTC T"GTCGGTCCT CCAAACGGAT   
  
107101 GAAGCAGCCA CCCTTGAAAG AGTGCGTAAT AGCTCACTGA TCGAGCGCTC TTGCGCCGAA GATGAACGGG   
       CTTCGTCGGT GGGAACTTTC TCACGCATTA TCGAGTGACT AGC"TCGCGAG AACGCGGCTT CTACTTGCCC "  
  
107171 GCTAAGCGAT CTGCCGAAGC TGTGGGATGT AAAAATGCAT CGGTAGGGGA GCGTTCCGCC TTAGAGGGAA   
       "CGATTCGCTA GACGGCTTCG ACACCCTACA TTTTTACGTA GCC"ATCCCCT CGCAAGGCGG AATCTCCCTT   
  
107241 GCACCTGCGC GAGCAGTTGT GGACGAAGCG GAAGCGAGAA TGTCGGCTTG AGTAACGCAA ACATTGGTGA   
       CGTGGACGCG CTCGTCAACA CCTGCTTCGC CTTCGCTCTT A"CAGCCGAAC TCATTGCGTT TGTAACCACT "  
  
107311 GAATCCAATG CCCCGAAAAC CTAAGGGTTC CTCCGCAAGG TTCGTCCACG GAGGGTGAGT CAGGGCCTAA   
       "CTTAGGTTAC GGGGCTTTTG GATTCCCAAG GAGGCGTTCC A"AGCAGGTGC CTCCCACTCA GTCCCGGATT   
  
107381 GATCAGGCCG AAAGGCGTAG TCGATGGACA A"CAGGTGAAT ATTCCTGTAC TACCCCTTGT TGGTCCCGAG "  
       CTAGTCCGGC TTTCCGCATC AGCTACCTGT TGTCCACTTA TAAGGACATG ATGGG"GAACA ACCAGGGCTC "  
  
107451 "GGACGGAGGA GGCTAGGTTA GCCGAAAGAT G"GTTATCGGT TCAAGGACGC AAGGTGCCCC TGTTTTTTTA   
       "CCTGCCTCCT CCGATCCAAT CGGCTTTCTA CCAATAGCCA AGTTCCTGCG TTCCA"CGGGG ACAAAAAAAT   
  
107521 GGGTAAGAAG AGGTAGAGAA AATACCTCGA GCCAATGTTC GAGTACCAGG CGCTACGGCG CTGAAGTAAC   
       CCCATTCTTC TCCATCTCTT TTATGGAGCT CGGTTACAAG "CTCATGGTCC GCGATGCCGC GACTTCATTG "  
  
107591 CCATGCCATA CTCCCAGGAA AAGCTCGAAC GACCTTCAAC AAAAGGGTAC CTGTACCCGA AACCGACACA   
       "GGTACGGTAT GAGGGTCCTT TTCGAGCTTG CTGGAAGTTG" TTTTCCCATG GACATGGGCT TTGGCTGTGT   
  
107661 GGTGGGTAGG TAGAGAATAC CTAGGGGCGC GAGACAACTC TCTCTAAGGA ACTCGGCAAA ATAGCCCCGT   
       CCACCCATCC ATCTCTTATG GATCCCCGCG CTCTGTT"GAG AGAGATTCCT TGAGCCGTTT TATCGGGGCA "  
  
107731 AACTTCGGGA GAAGGGGTGC CTCCTCACAA AGGGGGTCGC AGTGACCAGG CCCGGGCGAC TGTTTACCAA   
       "TTGAAGCCCT CTTCCCCACG GAGGAGTGTT TCCCCCA"GCG TCACTGGTCC GGGCCCGCTG ACAAATGGTT   
  
107801 AAACACAGGT CTCCGCAAAG TCGTAAGACC ATGTATGGGG GCTGACGCCT GCCCAGTGCC GGAAGGTCAA   
       TTTGTGTCCA GAGGCGTTTC AGCATTCTGG TACATACCCC CGACTGC"GGA CGGGTCACGG CCTTCCAGTT "  
  
107871 GGAAGTTGGT GACCTGATGA CAGGGGAGCC GGCGACCGAA GCCCCGGTGA ACGGCGGCCG TAACTATAAC   
       "CCTTCAACCA CTGGACTACT GTCCCCTCGG CCGCTGGCTT CGGGGCC"ACT TGCCGCCGGC ATTGATATTG   
  
107941 GGTCCTAAGG TAGCGAAATT CCTTGTCGGG TAAGTTCCGA CCCGCACGAA AGGCGTAACG ATCTGGGCAC   
       CCAGGATTCC ATCGCTTTAA GGAACAGCCC ATTCAAGGCT GGGCGTGCTT TCCGCAT"TGC TAGACCCGTG "  
  
108011 TGTCTCGGAG AGAGGCTCGG TGAAATAGAC ATGTCTGTGA AGATGCGGAC TACCTGCACC TGGACAGAAA   
       "ACAGAGCCTC TCTCCGAGCC ACTTTATCTG TACAGACACT TCTACGCCTG ATGGACG"TGG ACCTGTCTTT   
  
108081 GACCCTATGA AGCTTCACTG TTCCCTGGGA TTGGCTTTGG GCCTTTCCTG CGCAGCTTAG GTGGAAGGCG   
       CTGGGATACT TCGAAGTGAC AAGGGACCCT AACCGAAACC CGGAAAG"GAC GCGTCGAATC CACCTTCCGC "  
  
108151 AAGAAGGCCC CCT"TCGGGGG GGGCCCGAGC CATCAGTGAG ATACCACTCT GGAAGAGCTA GAATTCTAAC "  
       "TTCTTCCGGG GGAAGCCCCC CCCGGGCTCG GTAGTCACTC TATGGTG"AGA CCTTCTCGAT CTTAAGATTG   
  
108221 "CTTGTGTCAG GAC"CTACGGG CCAAGGGACA GTCTCAGGTA GACAGTTTCT ATGGGGCGTA GGCCTCCCAA   
       GAACACAGTC CTGGATGCCC GGTTCCCTGT CAGAGTCC"AT CTGTCAAAGA TACCCCGCAT CCGGAGGGTT "  
  
108291 AAGGTAACGG AGGCGTGCAA AGGTTTCCTC GGGCCAGACG GAGATTGGCC CTCGAGTGCA AAGGCAGAAG   
       "TTCCATTGCC TCCGCACGTT TCCAAAGGAG CCCGGTCT"GC CTCTAACCGG GAGCTCACGT TTCCGTCTTC   
  
108361 GGAGCTTGAC TGCAAGACCC ACCCGTCGAG CAGGGACGAA AGTCGGCCTT AGTGATCCGA CGGTGCCGAG   
       CCTCGAACTG ACGTTCTGGG TGGGCAGCTC GTCCCTGCTT TCAGC"CGGAA TCACTAGGCT GCCACGGCTC "  
  
108431 TGGAAGGGCC GTCGCTCAAC GGATAAAAGT TACTCTAGGG ATAACAGGCT GATCTTCCCC AAGAGCTCAC   
       "ACCTTCCCGG CAGCGAGTTG CCTATTTTCA ATGAGATCCC TATTG"TCCGA CTAGAAGGGG TTCTCGAGTG   
  
108501 ATCGACGGGA AGGTTTGGCA CCTCGATGTC GGCTCTTCGC CACCTGGGGC TGTAGTATGT TCCAAGGGTT   
       TAGCTGCCCT TCCAAACCGT GGAGCTACAG CCGAGAAGCG GTGGACC"CCG ACATCATACA AGGTTCCCAA "  
  
108571 GGGCTGTTCG CCCATTAAAG CGGTACGTGA GCTGGGTTCA GAACGTCGTG AGACAGTTCG GTCCATATCC   
       "CCCGACAAGC GGGTAATTTC GCCATGCACT CGACCCAAGT CTTGCAG"CAC TCTGTCAAGC CAGGTATAGG   
  
108641 GGTGTGGGCG TTAGAGCATT GAGAGGACCT TTCCCTAGTA CGAGAGGACC GGGAAGGACG CACCTCTGGT   
       CCACACCCGC AATCTCGTAA CTCTCCTGGA AAGGGATCAT GC"TCTCCTGG CCCTTCCTGC GTGGAGACCA "  
  
108711 GTACCAGTTA TCGTGCCCAC GGTAAACGCT GGGTAGCCAA GTGCGGAGCG GATAACTGCT GAAAGCATCT   
       "CATGGTCAAT AGCACGGGTG CCATTTGCGA CCCATCGGTT CA"CGCCTCGC CTATTGACGA CTTTCGTAGA   
  
108781 AAGTAGTAAG CCCACCCCAA GATGAGTGCT CTCCTATTCC GACTTCCCCA GAGCCTCCGG "TAGCACAGCC "  
       TTCATCATTC GGGTGGGGTT CTACTCACGA GAGGATAAGG CTGAAGGGGT CTCGGAG"GCC ATCGTGTCGG "  
  
108851 "GAGACAGCGA TGGGTTCTCT GCCCCTGCGG GGATGGAGCG ACAGAAGTTT TGAGAATTCA" AGAGA"AGGTC "  
       "CTCTGTCGCT ACCCAAGAGA CGGGGACGCC CCTACCTCGC TGTCTTCAAA ACTCTTA"AGT TCTCTTCCAG   
  
108921 "ACGGCGAGAT GAGCCGTTTA TCATCACGAT AGGTGTCAAG TGGAAGTGCA GTGATGTATG CAGCT"GAGGC   
       TGCCGCTCTA CTCGGCAAAT AGTAGTGCTA TCCACAGTTC ACCTTCA"CGT CACTACATAC GTCGACTCCG "  
  
108991 ATCCTAACAG ACCGGTAGAC TTGAAC"CTTG TTCCTACATG ACCCGATCAA TTCGATTAGG CACTCGCCAT "  
       "TAGGATTGTC TGGCCATCTG AACTTGGAAC AAGGATGTAC TGGGCTA"GTT AAGCTAATCC GTGAGCGGTA   
  
109061 "CTATTTTCAT TGTTCAACTC TTTGAC"AACA CGAAAAAACC ATTGTTCAAC TCTTTGACAA CATGAAAAAA   
       GATAAAAGTA ACAAGTTGAG AAACTGTTGT GCTTTTTTGG TAACAAGTT"G AGAAACTGTT GTACTTTTTT "  
  
109131 CCAAAAAAAG CTCTGCCCTC CCTCTC"TATC TATCCAAGGG ATGGAAGGGC AGAGGCCTTT GGTGTCCCCT "  
       "GGTTTTTTTC GAGACGGGAG GGAGAGATAG ATAGGTTCCC TACCTTCCC"G TCTCCGGAAA CCACAGGGGA   
  
109201 "CCAGTCAAGA ATTGGGGCCC CACAAT"CACT AGCCAATATG CTTTTCTCTC ATGCCTTTCT TCGTTCATGG   
       GGTCAGTTCT TAACCCCGGG GTGTTAGTGA TCGGTTATAC GAAAAGAGAG TA"CGGAAAGA AGCAAGTACC "  
  
109271 TTCGATATTC TGGTGTCC"TA GGCGTAGAGG AACCACACCA ATCCATCCCG AACTTGGTGG TTAAACTCTA "  
       "AAGCTATAAG ACCACAGGAT CCGCATCTCC TTGGTGTGGT TAGGTAGGGC TT"GAACCACC AATTTGAGAT   
  
109341 "CTGCGGTGAC GATACTGT"AG GGGAGGTCCT GCGGAAAAAT AGCTCGACGC CAGGATGATA AAAAGCTTAA   
       GACGCCACTG CTATGACATC CCCTCCAGGA CGCCTTTT"TA TCGAGCTGCG GTCCTACTAT TTTTCGAATT "  
  
109411 CACCTCTAAT TCTTATTA"CT TTTCAATATC AATATGGAAA AAAAATGAAA AATGAAAAGG TCGTCTTATT "  
       "GTGGAGATTA AGAATAATGA AAAGTTATAG TTATACCT"TT TTTTTACTTT TTACTTTTCC AGCAGAATAA   
  
109481 "CAAAACCCCT TCTCTCCC"AC TTTTCTATCT CACTTCACAC CTTGGAACGC ACCGTTCTTA TAGAGAGAGA   
       GTTTTGGGGA AGAGAGGGTG AAAAGATAGA GTGAAGTGTG GA"ACCTTGCG TGGCAAGAAT ATCTCTCTCT "  
  
109551 GGCGCTT"TCA CATCTTCTTA ACCCGAAATG GCAAATGGCT GGGGAGAGGA AAGGTTCCTT TTTTTAGGGT "  
       "CCGCGAAAGT GTAGAAGAAT TGGGCTTTAC CGTTTACCGA CC"CCTCTCCT TTCCAAGGAA AAAAATCCCA   
  
109621 "ACCCCCG"GAA CAGATCCAGT GGAGACGGGG TGGGGC"CTGT AGCTCAGAGG ATTAGAGCAC GTGGCTACGA "  
       TGGGGGCCTT GTCTAGGTCA CCTCTGCCCC ACCCCGGACA TCGAGTCTCC "TAATCTCGTG CACCGATGCT "  
  
109691 "ACCACGGTGT CGGGGGTTCG AATCCCTCCT CGCCCA"CAAC CGGCCAAAAA GGGAAGGACT TTT"CCCTCTG "  
       "TGGTGCCACA GCCCCCAAGC TTAGGGAGGA GCGGGTGTTG GCCGGTTTTT" CCCTTCCTGA AAAGGGAGAC   
  
109761 "GGGGTAGGAA AATCATGATC GGGATAGCGG ACCCAAAGCT ATGGAACTTG GGTGTGGGTC TTT"TGTCGAA   
       CCCCATCCTT TTAGTACTAG CCCTATCGCC TGGGTTTCGA TAC"CTTGAAC CCACACCCAG AAAACAGCTT "  
  
109831 ATGGAATGGC CTTATCTTTT TATTTTTTCT TTTTCGTTAA TGGGTTAAGG G"CGGGGGTTC CGTTATAAAT "  
       "TACCTTACCG GAATAGAAAA ATAAAAAAGA AAAAGCAATT ACC"CAATTCC CGCCCCCAAG GCAATATTTA   
  
109901 "TAAATATAGT ATACCCAACC CGAATCAGCA TATTTTTTTG TTTTACGCCC C"GTAATTCTT CCTCAGCCAG   
       ATTTATATCA TATGGGTTGG GCTTAGTCGT ATAAAAAAAC AAAATGCGGG GCATTAAG"AA GGAGTCGGTC "  
  
109971 GCTCGGGCAG AATAGCAGAG CAAGTACAAG TATTAGTAGA ATAGCAAAAA TGTGTTC"CTC GTCATTAATA "  
       "CGAGCCCGTC TTATCGTCTC GTTCATGTTC ATAATCATCT TATCGTTTTT ACACAAGG"AG CAGTAATTAT   
  
110041 "TGTTTGCTCG CGGTAATTGT GACCTCTCGG GAGAATCGAT GACTGCATCA AAGATGC"ACT TGCTAGTACT   
       ACAAACGAGC GCCATTAACA CTGGAGAGCC CTCTTAGCTA CT"GACGTAGT TTCTACGTGA ACGATCATGA "  
  
110111 AGTACATCTG AGAATTCTTA ATTGGCTAGT TGTAAATAGA CCCAGA"CTGT GGAACAAAGG ATTATCCCGG "  
       "TCATGTAGAC TCTTAAGAAT TAACCGATCA ACATTTATCT GG"GTCTGACA CCTTGTTTCC TAATAGGGCC   
  
110181 "ACCTACACCG AGGTATTGAC GGTGATTCTC AAATATCGCA GAACAG"AATT TGATACGATG AGATAGAATG   
       TGGATGTGGC TCCATAACTG CCACTAAGAG TTTATAGCGT CTTGTCTT"AA ACTATGCTAC TCTATCTTAC "  
  
110251 CAATAGAAAC AAAGACACAG GGAACGGGTT ACCTACTCTT AACGGTCAAA GCGAACCCTT TCAT"TCCGAA "  
       "GTTATCTTTG TTTCTGTGTC CCTTGCCCAA TGGATGAGAA TTGCCAGT"TT CGCTTGGGAA AGTAAGGCTT   
  
110321 "TTCTTTAATT CGGAATGAAT CAAATCTCCC CAAGTAGGAT TCGAACCTGC GACCAATCGG TTAA"CGGCCG   
       AAGAAATTAA GCCTTACTTA GTTTA"GAGGG GTTCATCCTA AGCTTGGACG CTGGTTAGCC AATTGCCGGC "  
  
110391 ACCGCTCTAC CACTGAGCTA CTGAGGAACA ACGGTAAATT AGGT"CTCAGA GAATTCAATT CCCGTTCTCA "  
       "TGGCGAGATG GTGACTCGAT GACTC"CTTGT TGCCATTTAA TCCAGAGTCT CTTAAGTTAA GGGCAAGAGT   
  
110461 "ACCCATGACC AATATGAGCT CGAGGTTTCC TTCGTAACTC CCGG"AACCTT CTTCGTAGTG GCTCCGTTCC   
       TGGGTACTGG T"TATACTCGA GCTCCAAAGG AAGCATTGAG GGCCTTGGAA GAAGCATCAC CGAGGCAAGG "  
  
110531 ATGCCTCATT TCATAGAGAA CCTCAAAGTG GCTCTATTTC ATTATATTCC ATCCATAT"CC CAATTCCATT "  
       "TACGGAGTAA AG"TATCTCTT GGAGTTTCAC CGAGATAAAG TAATATAAGG TAGGTATAGG GTTAA"GGTAA "  
  
110601 "CAGTTAATAT CCCTGTGGTG TCATTGAGAT AAGAGATGTC GTTTCTAGTC TATCTGTT"TC TCTAGTCTAT   
       "GTCAATTATA GGGACACCAC AGTAACTCTA TTCTCTACAG CAAAGATCAG ATAGACAAAG AGATC"AGATA   
  
110671 CTGTTTCTAT TTCTATATAT ATGGAAAGTT AAAAAATCAT CATATAATAA TATAATAATC TAGAAATTGC   
       GACAAAGATA AAGATATATA TACCTTTCAA TTTTTTAGTA GTATATTATT ATATTATTAG ATCTTTAACG   
  
110741 AATAGAAAAG AAAAAAAATT GCAATAGAAA AGAAAAAGGG AGGTTTGTGA TGATTTTAAA ATCTTTTATA   
       TTATCTTTTC TT"TTTTTTAA CGTTATCTTT TCTTTTTCCC TCCAAACACT ACTAAAATTT TAGAAAATAT "  
  
110811 CTAGGTAATC TAGTATCCCT ATGCATGAAG ATA"ATCAATT CGGTCGTTGT GGTCGGACTC TATTATGGAT "  
       "GATCCATTAG AT"CATAGGGA TACGTACTTC TATTAGTTAA GCCAGCAACA CCAGCCTGAG ATAATACCTA   
  
110881 "TTCTGACCAC ATTCTCCATA GGGCCCTCTT ATC"TCTTCCT TCTCCGAGCT TGGCTTATGG AAGAAGGAAC   
       AAGA"CTGGTG TAAGAGGTAT CCCGGGAGAA TAGAGAAGGA AGAGGCTCGA ACCGAATACC TTCTTCCTTG "  
  
110951 CGAGAAGAAA GTATCAGC"AA CAACAGGTTT TATTACGGGA CAGCTCATGA TGTTCATATC GATCTATTAT "  
       "GCTC"TTCTTT CATAGTCGTT GTTGTCCAAA ATAATGCCCT GTCGAGTACT ACAAGTATAG CTAGATAAT"A "  
  
111021 "GCGCCTCTGC ATCTAGCA"TT GGGTAGACCT TATACAATAA CTGTCCTAGC TCTACCGTAT CTTTTGTTTC   
       "CGCGGAGACG TAGATCGTAA CCCATCTGGA ATATGTTATT GACAGGATCG AGATGGCATA GAAAACAAA"G   
  
111091 ATTTCTTCTG GACCAATCCC AAACACTTTT TTTATTATGG ATCTATTACC AGAAACTCAA TGCGTAATCT   
       TAAAGAAGAC CTGGTTAGGG TTTGTGAAAA AAATAATACC TAGATAATGG TCTTTGAGTT ACGCATTAGA   
  
111161 TAGCATTCAA TGTGTATTCC TGAATAATCT CATTTTTCAA TTATTC"AACC ATTTCATTTT ACCAAGTTCA "  
       "ATCGTAAGTT ACACATAAGG ACTTATTAGA GTAAAAAGTT AATAAGTTGG TAAAGTAAAA TGGTTCAAGT"   
  
111231 "ATGTTAGTCA GATTAGTCAA CATTTCTATG TTTCGATGCA ACAACA"AGAT GTTATTTGTA ACAAGTAGTT   
       TACAATCAGT CTAATCAGTT GTAAAGATAC AAAGCTACGT TGTTGTTCTA CAATAAACAT TGTTCATCAA   
  
111301 TTGTTGGTTG GTTAATTGGT CACATTTTAT TCATGAAATC GGTTGGATTG ATATT"AGTCT GGATACAGCA "  
       AACAA"CCAAC CAATTAACCA GTGTAAAATA AGTACTTTAG CCAACCTAAC TATAATCAGA CCTATGTCGT "  
  
111371 "AAATAATTCT ATTATATCTA AGAAGTACAT TAGTTCTAAT AAGTACCTTG TGTCA"GAATT GAGAAATTCT   
       "TTTAT"TAAGA TAATATAGAT TCTTCATGTA ATCAAGATTA TTCATGGAAC ACAGTCTTAA CTCTTTAAGA   
  
111441 ATAGCCCCAA TCCTTTAGTA TTATCTTATT TATTAC"CTGG GTCTACTATT TAGGCAGAAT ACCGTCACCC "  
       TATCGGGGTT AGG"AAATCAT AATAGAATAA ATAATGGACC CAGATGATAA ATCCGTCTTA TGGCAGTGGG "  
  
111511 "ATTCCACTGA AAGAAACCTC AAAAACCTCA GAAACG"GCGG AAACGGAGGA AAGCGAGGAA GAAACAGATG   
       "TAAGGTGACT TTC"TTTGGAG TTTTTGGAGT CTTTGCCGCC TTTGCCTCCT TTCGCTCCTT CTTTGTCTAC   
  
111581 TAGAAATAGA AACCACTTCC GAAACGAAGG GGACTAAACA GGA"ACAAGAG GGATCCACCG AAGAAGATAC "  
       ATCTTTATCT TT"GGTGAAGG CTTTGCTTCC CCTGATTTGT CCTTGTTCTC CCTAGGTGGC TTCTTCTATG "  
  
111651 "TTCTCCTTCC CTTTTTTCGG AAGAAAAGGA GGATCCCGGA CAA"AATCGAT GAAACGGAAG AGATCCGAAT   
       "AAGAGGAAGG GA"AAAAAGCC TTCTTTTCCT CCTAGGGCCT GTTTTAGCTA CTTTGCCTTC TCTAGGCTTA   
  
111721 AGATAAAAAA GAAAAATATC TCTTCGGGTT TGAAAAACCT CTTGGGATTA TCCTTTTCGA CTATAAACGA   
       TCTATTTTTT C"TTTTTATAG AGAAGCCCAA ACTTTTTGGA GAACCCTAAT AGGAAAAGCT GATATTTGCT "  
  
111791 TGGAATCGTC CATTTCGATA TATAAAGAAT GATAAATTTG ACAATGCTGT TAGCAAAAAA CAAAGTAAAT   
       "ACCTTAGCAG G"TAAAGCTAT ATATTTCTTA CTATTTAAAC TGTTACGACA ATCGTTTTTT GTTTCATTTA   
  
111861 TACTAATATA AAAATAACTA AATAAACTAA ATACAACAAG ATA"TAAGACG AGATTCGACC ACCTCCTACA "  
       ATGATTATAT TTTTATTGAT TTATTTGATT TATGTTGTTC TATATTCTGC T"CTAAGCTGG TGGAGGATGT "  
  
111931 "TATTTAATAC TCTCTGCTAC AAAAAAATTA AGAATACCCA CCG"CATTGGT AATTCCATCA ATTATCCGTC   
       "ATAAATTATG AGAGACGATG TTTTTTTAAT TCTTATGGGT GGCGTAACCA T"TAAGGTAGT TAATAGGCAG   
  
112001 GATCAAAAAA ATAAGTTAAT TCAGCTAATA T"TCTTATACC CCCAATTAAG GATATTCGAT AAAAAGTATC "  
       CTAGTTTTTT TATTCAATTA AGTCGATTAT AAGAATATGG GGGTTAATTC CTATAAGCTA TTTTTCATAG   
  
112071 "TATGTAACCA CGATTATATG ACCAATTATA T"ATTATATTT ATAATTTTTT CAAAAAAAAA ATTCTTAGTA   
       ATACATTGGT GCTAATATAC TGGTTAATAT ATAATATAAA TATTAAAAAA GTTTTTTTTT TAAGAATCAT   
  
112141 AAACTTTTAA CAAATGAATT ACGAAAATTC AAATTTTGTA AAGATG"AATA AGTGGGCTTA TAGAAGAAAG "  
       TTTGAAAATT GTTTACTTAA TGCTTTTAAG TTTAAAACAT TTCTACTTAT TCACCCGAAT ATCTTCTTTC   
  
112211 "ACGCTATAAA TATTCCGAAA AAAGCTATAG TCACAGAAAA AGTTGC"ATTT TTAACAAATT CATACCAATT   
       TGCGATATTT ATAAGGCTTT TTTCGATATC AGTGTCTTTT TCAACGTAAA AATTGTTTAA GTATGGTTAA   
  
112281 TTCAGAATCT TTTGAACTTT CGTGTAACAA GTTT"ATAGAC GGAGTTAACC ATTTGTCTAA TATATTCAAA "  
       AAGTCTTAGA AAACTTGAAA GCACATTGTT CAAATATCTG CCTCAATTGG TAAACAGATT ATATAAGTTT   
  
112351 "TCAATTGCTT CTTGATTGAA TTGAGTGAAC GGGC"TTCCTA TGACTCCAAC AAACAAAGTA AATAGGGATA   
       AGTTAACGAA GAACTAACTT AACTCACTTG CCCGAAGGAT ACTGAGGTTG TTTGTTTCAT TTATCCCTAT   
  
112421 ACACAAACAT CGGAAATAGC ATAGTAT"TCT CTGATTCATA GGGATAGTAA AAAGTATTTT TAGTACTAAA "  
       TGTGTTTGTA GCCTTTATCG TATCATAAGA GACTAAGTAT CCCTATCATT TTTCATAAAA ATCATGATTT   
  
112491 "ATGAGGACTA CTAACAAAAG GGCGCAT"TTT TTTTGTTACA TTACTATTAA ATGGATATAT TGTTTTTTTC   
       TACTCCTGAT GATTGTTTTC CCGCGTAAAA AAAACAATGT AATGATAATT TACCTATATA ACAAAAAAAG   
  
112561 ATCCAAAAAG AAGACCTTTC TTTATTATTC ATTGTTAATA ATGA"GAAGAA AGGGAAGTTT TTTTTAATGA "  
       TAGGTTTTTC TTCTGGAAAG AAATAATAAG TAACAATTAT TACTCTTCTT TCCCTTCAAA AAAAATTACT   
  
112631 "TTTTTGATCC TTCTTTACCC CATAATGATA TTGAATAAAG GGAG"TTATTT GTTTTTCCAC TGTAGTTTTT   
       AAAAACTAGG AAGAAATGGG GTATTACTAT AACTTATTTC CCTCAATAAA CAAAAAGGTG ACATCAAAAA   
  
112701 ACAATAAAGG TTTAAATGTC CATCAAAAGT AAGTA"AGTAA ATGCGAAACA TATAAAATGC TGTTAATCCT "  
       TGTTATTTCC AAATTTACAG GTAGTTTTCA TTCATTCATT TACGCTTTGT ATATTTTACG ACAAT"TAGGA "  
  
112771 "GCTGTGGAAC AGGCGATTAT TGCGAAAATC GGTGA"ATACA ACCAACTATC ATTAAGAATT TCATCTTTGG   
       "CGACACCTTG TCCGCTAATA ACGCTTTTAG CCACTTATGT TGGTTGATAG TAATTCTTAA AGTAG"AAACC   
  
112841 ACCAAAAGCA GGCAAGGGGT GGAACA"CCAC AAAGAGAAAG GGTACCTAAT AAAAAAGCAT TTTTTGTAAT "  
       TGGTTTTCGT CCGTTCCCCA CCTTGTGGTG TTTCTCTTTC CCATGGATTA TTTTTTCGTA AAAAACATTA   
  
112911 "TGGCACATGC TTTGTTAAAC CACCCA"TAAG AACCATATTC TGACTTTTAT CTGGAGAATA TCCAACAACC   
       ACCGTGTACG AAACAATTTG GTGGGTATTC TTGGTATAAG ACTGAAAATA GACCTCTTAT AGGTTGTTGG   
  
112981 GATTCCATTG AGTGAATAAT GGACCCCGAT CCTAAAAACA A"CAATGCTTT TGAATAAGCA TGAGTAATCA "  
       CTAAGGTAAC TCACTTATTA CCTGGGGCTA GGATTTTTGT TGTTACGAAA ACTTATTCGT ACTCATTAGT   
  
113051 "AATGAAATAA AGCAGCCTGG TATGACCCCA TACCCAAAGC T"AACATCATA TAGCCCAATT GAGACATTGT   
       TTACTTTATT TCGTCGGACC ATACTGGGGT ATGGGTTTCG ATTGTAGTAT ATCGGGTTAA CTCTGTAACA   
  
113121 AGAATAGGCT AAACTTCTCT TAATATCTTT TTGAGCA"AGA GCTAAAGTAG CTCCAAATAG TACTGTTATT "  
       TCTTATCCGA TTTGAAGAGA ATTATAGAAA AACTCGTTCT CGATTTCATC GAGGTTTATC ATGACAATAA   
  
113191 "ATACCTATCA AAGCTATTAG ATTCATGATG TAAGGTA"TTG CTATAAAAAG CGGAAGAAGC CGGGCGACAA   
       TATGGATAGT TTCGATAATC TAAGTACTAC ATTCCATAAC GATATTTTTC GCCTTCTTCG GCCCGCTGTT   
  
113261 GAAAAATTCC CGCTGCTACC ATGGTAGCAG CATGTATAA"G AGCCGAAATG GGGGTAGGAC CCTCCATAGC "  
       CTTTTTAAGG GC"GACGATGG TACCATCGTC GTACATATTC TCGGCTTTAC CCCCATCCTG GGAGGTATCG "  
  
113331 "ATCGGGTAAC CATACATGAA GAGGAAATTG AGCAGATTT"A GCAACAGCAC CTGCAAATAA TAGGACGGCG   
       "TAGCCCATTG GT"ATGTACTT CTCCTTTAAC TCGTCTAAAT CGTTGTCGTG GACGTTTATT ATCCTGCCGC   
  
113401 CACAAAGTAA GAAATAAGAA ATTTACCTCA TTATTATCAA TCAA"GTTATT GAATATTTGA AATAAATCCC "  
       GTGTTTCATT CTTTATTCTT TAAATGGAGT AATAATAGTT AGTTCAATAA CTTATAAACT TTATTTAGGG   
  
113471 "GAAATTCAAA ACTCGCCGTT GTCCAATAAA GACCTAAAAT TCCT"AATAAT AAACCAAAAT CCCCTACGCG   
       CTTTAAGTTT TGAGCGGCAA CAGGTTATTT CTGGATTTTA AGGATTATTA TTTGGTTTTA GGGGATGCGC   
  
113541 ATTAGTTACA AATGCCTTTT GACCAGC"ATT CGCCGCACTA GGTCGAGTGA ACCAAAAACC TATTAATAGA "  
       TAATCAATGT TTACGGAAAA CTGGTCGTAA GCGGCGTGAT CCAGCTCACT TGGTTTTTGG ATAATTATCT   
  
113611 "TAAGAACACA TTCCACCCAA TTCCCAA"AAA ATATAAATTT GGATCAAATT CGAACTAGTA ACTAATCCCA   
       ATTCTTGTGT AAGGTGGGTT AAGGGTTTTT TATATTTAAA CCTAGTTTAA GCTTGATCAT TGATTAGGGT   
  
113681 ACATTGACGT ATTGAACAAA CTCATATACG CAAAAAATCT CAAATA"TCCT TGATCATGAG ACATATAATT "  
       TGTAACTGCA TAACTTGTTT GAGTATATGC GTTTTTTAGA GTTTATAGGA ACTAGTACTC TGTATATTAA   
  
113751 "GTCACTATAA ATAAGAACCA TAATTCCAAC AGTCGTGATT AATATT"GCCA TAATACAAGT AAGTGGATCG   
       CAGTGATATT TATTCTTGGT ATTAAGGTTG TCAGCACTAA TTATAACGGT ATTATGTTCA TTCACCTAGC   
  
113821 ATGAAGTAGC CCAACTCTAA AGAAAAA"TCA TTATTGATAG TCCAAGACCA TACATATTGA TAGATATAAC "  
       TACTTCATCG GGTTGAGATT TCTTTTTAGT AATAACTATC AGGTTCTGGT ATGTATAACT ATCTATATTG   
  
113891 "TAGTATTTAT TTGATCAATA GACAGAT"AAA CGGCAAAAAT CATAACTATA CTTAACAATA AAATACTCGG   
       ATCATAAATA AACTAGTTAT CTGTCTATTT GCCGTTTTTA GTATTGATAT GAATTGTTAT TTTATGAGCC   
  
113961 AAACGACCAC AGACGTCGAA GGTTTTT"GGT TGCCGTCGGA AAAAGTAGAA GGCCCACTCC TATTAAGATA "  
       TTTGC"TGGTG TCTGCAGCTT CCAAAAACCA ACGGCAGCCT TTTTCATCTT CCGGGTGAGG ATAATTCTAT "  
  
114031 "GGAATTGGAA GTGAGATGAA CGGTATG"ATC CATGAATATT GATATGTATA TTCCATAAAA AAGTATATTA   
       "CCTTA"ACCTT CACTCTACTT GCCATACTAG GTACTTATAA CTATACATAT AAGGTATTTT TTCATATAAT   
  
114101 AATTCCTAAT TAATTTTTCT GATTCA"CCAG CTCTTATCTC TTTTCAAAAG GATCAGTTAA TAACAAATTC "  
       TTAAGGATTA ATTAAAAAGA CTAAGTGGTC GAGAATAGAG AAAAGTTTTC CTAGTCAATT ATTGTTTAAG   
  
114171 "AAATATCCAA AACTTAAATA GAATTT"TATT CTTCCATTCT TAATTTTTTG CCAAATACTT CAAATATTTC   
       TTTATAGGTT TTGAATTTAT CTTAAAATAA GAAGGTAAG"A ATTAAAAAAC GGTTTATGAA GTTTATAAAG "  
  
114241 AAGTCACGAA GTTCCAACTG TTCAAATAAG AT"GATCCACT GAAACTATTA ATGAACACAC CCATTACTTT "  
       "TTCAGTGCTT CAAGGTTGAC AAGTTTATTC TACTAGGTG"A CTTTGATAAT TACTTGTGTG GGTAATGAAA   
  
114311 "TAAGTCAAAT TTTTTCACAA GATAGAAACT GC"AAATTTTC TTTATTATTT AGTATGCATT ACAAAAATTC   
       ATTCAGTTTA AAAAAGTGTT CTATCTTTGA CGTTT"AAAAG AAATAATAAA TCATACGTAA TGTTTTTAAG "  
  
114381 CCCGCTCTAG AGCAAGAAAG AATAATTAGA CCAAAAACAC TAT"TTTATTT TGGTATCAAT CATAAAATAA "  
       "GGGCGAGATC TCGTTCTTTC TTATTAATCT GGTTT"TTGTG ATAAAATAAA ACCATAGTTA GTATTTTATT   
  
114451 "AAGACAGTCT ACAAGTTGAT CCATTAAAAT AAGACTTCTT TGT"ATTAAAT TCTACGAATC ATTAAACAAT   
       TTCTGTCAGA TGTTCAACTA GGTAATTTTA TT"CTGAAGAA ACATAATTTA AGATGCTTAG TAATTTGTTA "  
  
114521 TTTTGTTTTT TGACAAAATA ACACTATATA TATATTGTTT TTTCT"TTGTT TCTAATCTAA TAATTTAGAA "  
       "AAAACAAAAA ACTGTTTTAT TGTGATATAT AT"ATAACAAA AAAGAAACAA AGATTAGATT ATTAAATCTT   
  
114591 "TTTTCGATAG CTATATTAGG AGTATACTCT AATTTCAAGA ATAAA"TGGAT AATAAATAGT AAAGAACAAT   
       AAAAGCTATC GATATAATCC TCATATGAGA TTAAAGTTCT TATTTACCTA TT"ATTTATCA TTTCTTGTTA "  
  
114661 TCATTTTGAA CAATAGATGT CTTTCACACT CAACTATAGT AATAAATAAT CAATTATAAT TTTGTAATGG   
       "AGTAAAACTT GTTATCTACA GAAAGTGTGA GTTGATATCA TTATTTATTA GT"TAATATTA AAACATTACC   
  
114731 CACTTCCAAA AAAGCGCACT TCTATAT"CAA AAAAACGGAT TCGGAAAAAT ATTTGGAAAA GCAAAGGGCG "  
       GTGAAGGTTT TTTCGCGTGA AGATATAGTT TTTTTGCCT"A AGCCTTTTTA TAAACCTTTT CGTTTCCCGC "  
  
114801 "TCGGGCAGCG CTGAAAGCTT TTTCCCC"AGC AAAATCTCTT TCAACGGGTA ATTCAAAAAG TTTTTGGGGG   
       "AGCCCGTCGC GACTTTCGAA AAAGGGGTCG TTTTAGAGA"A AGTTGCCCAT TAAGTTTTTC AAAAACCCCC   
  
114871 GACAAATCAA ATAAATACTG AAA"GATTGGA ATAAGCTGAA TTGGTTTGAC TCAAAAAACA CCTTAGTAAA "  
       CTGTTTAGTT TATTTATGAC TTTCTAACCT TAT"TCGACTT AACCAAACTG AGTTTTTTGT GGAATCATTT "  
  
114941 "AGTTCGTTTA TAATTTATAT ATA"TTAAATA AGATAAATAA TTAAATAAGA TAAATAAAGA TAAATAATTA   
       "TCAAGCAAAT ATTAAATATA TATAATTTAT TCT"ATTTATT AATTTATTCT ATTTATTTCT ATTTATTAAT   
  
115011 AATAAGATAA ATAA"GAATTT TTTCTTTTAT CAAAGCAATT AGTGGAATTT CCCTAATGTT TTGAGCAGAT "  
       TTATTCTATT TATTCTTAAA AAAGAAAATA GTTTCGTTAA "TCACCTTAAA GGGATTACAA AACTCGTCTA "  
  
115081 "AAATATGAAA TCCC"TTTTCC TTTTTGTAGG CTATGTAAAA AAAAAAAATA GACTAAATCT TTCGTTTACT   
       "TTTATACTTT AGGGAAAAGG AAAAACATCC GATACATTTT" TTTTTTTTAT CTGATTTAGA AAGCAAATGA   
  
115151 CAATTAAAAA ATAGAAAATG TTACTTTTCT GTT"CAAAGAA TAAAAAGGGT TGACCTTTCT TTTTCATATC "  
       GTTAATTTTT TATCTTTTAC AATGAAAAGA CAAGTTTCTT ATTTTTCCCA A"CTGGAAAGA AAAAGTATAG "  
  
115221 "TTTGTTTTAT CATTTTCGGG ATGGGGAAAA TAT"GAATCCC CATCGCCCCA CTAAACCAAA AAGTTTTTCT   
       "AAACAAAATA GTAAAAGCCC TACCCCTTTT ATACTTAGGG GTAGCGGGGT G"ATTTGGTTT TTCAAAAAGA   
  
115291 TCATTTTTGA TTTTATTATA TATTTTCGAT ATTA"TATATA ATATCGAAAT ATAGAAAGTA AACTGAAACT "  
       AGTAAAAACT AAAATAATAT ATAAAAGCTA TAATATATAT TA"TAGCTTTA TATCTTTCAT TTGACTTTGA "  
  
115361 "CTTTATTAAA AATTTAATAA GACATTGAAA CGAC"GACATT AGTTGATTAA GTTAAAACCT TCTTTTTTTA   
       "GAAATAATTT TTAAATTATT CTGTAACTTT GCTGCTGTAA TC"AACTAATT CAATTTTGGA AGAAAAAAAT   
  
115431 ATTCTATGAA CCCTT"ACCTT ATTTCTTTTG TAGAAATCAT TATTACTATT ATAGAATATA TCCCACCGAA "  
       TAAGATACTT GGGAATGGAA TAAAGAAAAC ATCTTTAGTA ATAATGATAA TA"TCTTATAT AGGGTGGCTT "  
  
115501 "TACATAATGA AATTG"GTTTG CAAAATTCTA TAAAAGAATT ATTAAATGAA GAAAATGGAC ACCTTAAATT   
       "ATGTATTACT TTAACCAAAC GTTTTAAGAT ATTTTCTTAA TAATTTACTT CT"TTTACCTG TGGAATTTAA   
  
115571 CTTATTGAAA TAAATGAAAT CGGATAAGAT TTT"TATTGGA AACGCTCAAA TCTCCTATTT TTTTCCCTTT "  
       GAATAACTTT ATTTACTTTA GCCTATTCTA AAA"ATAACCT TTGCGAGTTT AGAGGATAAA AAAAGGGAAA "  
  
115641 "ACTTTAATGA AATTATCGTT TAAAAATAAA GAG"TTTTTTA TCCACGATAA ATATTTTTTA CAAAAATATT   
       "TGAAATTACT TTAATAGCAA ATTTTTATTT CTC"AAAAAAT AGGTGCTATT TATAAAAAAT GTTTTTATAA   
  
115711 CCATTGAATT GAAAATTCAT TCGATTTTTT CAATCTCGAC GATTGAATAA TAATAGGTTA TTATGATTTC   
       GGTAACTTAA CTTTTAAGTA AGCTAAAAAA GTTAGAG"CTG CTAACTTATT ATTATCCAAT AATACTAAAG "  
  
115781 GAGAAA"GCCG CTATGGTGAA ATCGGTAGAC ACGCTGCTCT TAGGAAGCAG TGCTAGAGCA TCTCGGTTCG "  
       "CTCTTTCGGC GATACCACTT TAGCCATCTG TGCGACG"AGA ATCCTTCGTC ACGATCTCGT AGAGCCAAGC   
  
115851 "AGTCCG"AGTG GCGGCATGCC ATTTAAAATT ATAAAAAAAA GTT"CTATAAT ATAATAAATT CAAGTCCCAG "  
       TCAGGCTCAC CGCCGTACGG TAAATTTTAA TATTTTTTTT CAA"GATATTA TATTATTTAA GTTCAGGGTC "  
  
115921 "ATATTAATTC AAATAATTGA ATTGAGGGAC CTTTCAATTT TTT"TTTTTTT ATGATATTTT CAACTTTTGA   
       "TATAATTAAG TTTATTAACT TAACTCCCTG GAAAGTTAAA AAA"AAAAAAA TACTATAAAA GTTGAAAACT   
  
115991 GCATATATTA ACTCATATAT CTTTTTCGGT CATTTCAATT GTCATTACAA TTCAGTTAAT AACCTTATTA   
       CGTATATAAT TGAGTATATA GAAAAAGCCA GTAAAGTTAA CAGTAATGTT AA"GTCAATTA TTGGAATAAT "  
  
116061 ATCAATGAA"A CCGTAGGACT CTATGTTTCG TCAGAAAAGG GCATGATAGC TACTTTTTTC TGTATAACAG "  
       "TAGTTACTTT GGCATCCTGA GATACAAAGC AGTCTTTTCC CGTACTATCG AT"GAAAAAAG ACATATTGTC   
  
116131 "GATTATTAG"T TACTCGTTGG ATTTATTTGA GGCATTTACC ATTAAGTGAT TTATATGAAT CATTACTATT   
       CTAATAATCA ATGAGCAACC TAAATAAACT CCGTAAAT"GG TAATTCACTA AATATACTTA GTAATGATAA "  
  
116201 TCTTTCATGG GCTTTCTCCA TTATTCATCT ATTTACGTAT TTTAAAAATA TAAAAACCAT "TAAGTGTAAG "  
       "AGAAAGTACC CGAAAGAGGT AATAAGTAGA TAAATGCA"TA AAATTTTTAT ATTTTTGGTA ATTCACATTC   
  
116271 "CGCAATAACC GCGCCAGTAC TATTTTTACA CAAGGTTTTG CTACTTCAGG TTTTTTAACT" GAAATGCATC   
       GCGTTATTGG CGCGGTCATG ATAAAAATGT GTTCCAAA"AC GATGAAGTCC AAAAAATTGA CTTTACGTAG "  
  
116341 CATCCCCACT ATTAGTTCCC GCTCTCCAAT CTCATTGGTT AATGATGCAC GTAAGTATGA TGGTATTAGG   
       "GTAGGGGTGA TAATCAAGGG CGAGAGGTTA GAGTAACC"AA TTACTACGTG CATTCATACT ACCATAATCC   
  
116411 TTATGCCGCT CTTTTATGTG GATCATTATT TTCAGTAGCT CTTATAGTGA TTACCTTTCA AAAAGCTATA   
       AATACGGCGA GAAAATACAC CTAGTAATAA AAGTCAT"CGA GAATATCACT AATGGAAAGT TTTTCGATAT "  
  
116481 AGAATTTTTT GTAAAAACAA TAATTTATTA AATGCGTTAT TTTCCTTTAA TGAGATCCAA TCCATCAACG   
       "TCTTAAAAAA CATTTTTGTT ATTAAATAAT TTACGCA"ATA AAAGGAAATT ACTCTAGGTT AGGTAGTTGC   
  
116551 AA"GGGAACTA TTTTGTAAGA AACACTTCCT TTTTTTCTTC GAAGAATTAT TATAAGTCTC AACTGATTCA "  
       TTCCCTTGAT AAAACATTCT TTGTGAAGGA AAAAAAGAAG CT"TCTTAATA ATATTCAGAG TTGACTAAGT "  
  
116621 "AC"AATTAGAT CATTGGAGTT GTCGTATTAT TAGTCTAGGG TTTATCTTTT TAAGCATAGG TATTCTTTCA   
       "TGTTAATCTA GTAACCTCAA CAGCATAATA ATCAGATCCC AA"ATAGAAAA ATTCGTATCC ATAAGAAAGT   
  
116691 GGGGCAGTAT GGGCTAATGA GACC"TGGGGA TCGTATTGGA ATTGGGACCC AAAGGAAACT TGGGCATTTA "  
       CCCCGTCATA CCCGATTACT CTGGACCCCT AGCATAA"CCT TAACCCTGGG TTTCCTTTGA ACCCGTAAAT "  
  
116761 "TTACTTGGAC GATATTCGCA ATTT"ATTTAC ATACGCGAAC AAATAAAAAT TTTGAAGGTG TAAATTCCGC   
       "AATGAACCTG CTATAAGCGT TAAATAAATG TATGCGC"TTG TTTATTTTTA AAACTTCCAC ATTTAAGGCG   
  
116831 AATTGTGGCC TCTATGGGTT TTCTTATAAT TTGGATATGC TATTTTGGGG TCAATCTATT AGGGATAGGG   
       TTAACACCGG AGATACCCAA AAGAATATTA AA"CCTATACG ATAAAACCCC AGTTAGATAA TCCCTATCCC "  
  
116901 CTACATAGTT ATGGTCCGTT TACATTAACA TCTAATTGAA TTCATTC"AAG AAAGGGCCTG ACGACCACAA "  
       "GATGTATCAA TACCAGGCAA ATGTAATTGT AG"ATTAACTT AAGTAAGTTC TTTCCCGGAC TGCTGGTGTT   
  
116971 "ACATGAGAGG GTATAGACTA GAAAACTGTG ATTCAAATGG TTCTCAC"AAA AGCCAAATGT ATGAGGAAGT   
       TGTACTCTCC CATATCTGAT CTTTTGACAC TAAGTTT"ACC AAGAGTGTTT TCGGTTTACA TACTCCTTCA "  
  
117041 CCAATTCATT TTTTTATTTA ACTTAAGAAA AAAGACTTCT TTTTTTTAT"T GTGCAACGAA CGATTAAAAA "  
       "GGTTAAGTAA AAAAATAAAT TGAATTCTTT TTTCTGA"AGA AAAAAAATAA CACGTTGCTT GCTAATTTTT   
  
117111 "AAATTATTAT AGTATTGCTG TTTCATTTTT TTATGAAAAC TATCTAGCA"A AATAATTAGA TAAAATAGCT   
       TTTAATAATA TCATAACGAC AAAGTAAAAA AATACTTTTG ATAGATCGTT TTATTAATCT ATTTTATCGA   
  
117181 TCGACCTTGT CAACTGATAA TGAGAAAACA AAATCTGGAT AAATACCAAT "ACCTATGACA GGTATAAGGA "  
       AGCTGGAACA GTTGACTATT ACTCTTTTGT TTTAGACCTA TTTATGGTTA TGGATACTGT CCATATTCCT   
  
117251 "TAGAGATCGC AACAAACAAT TCTCGTGGTC CCGAATCAAA AAAATAAGAA" TTTTTAGCAT TAAAAAGCTT   
       ATCTCTAGCG TTGTTTGTTA AGAGCACCAG GGCTTAGTTT TTTTATTCTT AAAAATCGTA ATTTTTCGAA   
  
117321 ATATCCATAG AACATCTGGC GTAACATAGA TAATAAATAA ATGGGAGTT"A ATATAATTCC AATTGCCATT "  
       TATAGGTATC TTGTAGACCG CATTGTATCT ATTATTTATT TACCCTCAAT TATATTAAGG TTAACGGTAA   
  
117391 "ACCAAAGTAA TTACCATTTT TGTCATTAAA AGATATTTTT GGCTGGTAA"T TATTCCAAAA AAGACTATTA   
       TGGTTTCATT AATGGTAAAA ACAGTAATTT TCTATAAAAA CCGACCATT"A ATAAGGTTTT TTCTGATAAT "  
  
117461 ATTCTGCAAC AAAACCGCTC ATACCGGGCA ATGCAAGGGA AGCCATCGAT AAGAT"ATTGA AAGTCGTAAA "  
       "TAAGACGTTG TTTTGGCGAG TATGGCCCGT TACGTTCCCT TCGGTAGCT"A TTCTATAACT TTCAGCATTT   
  
117531 "TATTTTTGGA ATTGGGATAG CCAGTCCTCC CATTTCATCA AGATAAACCA GACGT"ATTCT ATCATAACTT   
       ATAAAAACCT TAACCCTATC GGTCAGGAGG GTAAAGTAGT TCTATTTGGT CTGCATAAGA TAGTATTGAA   
  
117601 GTTCCCGCTA AGAAAAAAAG CGCAGCGCCA ATAAATCCAT GAGAGATTAT TTGTAAAAT"A GCTCCATTCA "  
       CAAGGGCGAT TCTTTTTTTC GCGTCGCGGT TATTTAGGTA CTCTCTAATA AACATTTTAT CGAGGTAAGT   
  
117671 "GTCCCGTATC GCTTATAGAA CCAATTCCTA TAATTATGAA ACCCATATGA GAGACGGAG"G AATAAGCTAT   
       CAGGGCATAG CGAATATCTT GGTTAAGGAT ATTAATACTT TGGGTATACT CTCTGCCTCC TTATTCGATA   
  
117741 TCTTTTTTTT AAATTGCGTT GACCCGAAGA TGTTGAAGCT GCATAGATTA TTTGAATTAT GCCTA"CTATG "  
       AGAAAAAAAA TTTAACGCAA CTGGGCTTCT ACAACTTCGA CGTATCTAAT AAACTTAATA CGGAT"GATAC "  
  
117811 "ATCAACCAGG GTGAAAAGAT AGAATGGGCG TGGGGTAATA ATTCCATATT GATCCGAACC AATCC"ATATG   
       "TAGTTGGTCC CACTTTTCTA TCTTACCCGC ACCCCATTAT TAAGGTATAA CTAGGCTTGG TTAGG"TATAC   
  
117881 CTCCCATTTC TAATAAGATT CCGGATAGAA GCATACAAGT ACTGTAA"TGT GCCTCTCCGT GGGTATCTGG "  
       GAGGGTAAAG ATTATTCTAA GGCCTATCTT CGTATGTTCA TGACATTACA CGGAGAGGCA CCCATAGACC   
  
117951 "TAACCATGTA TGTAAGGGTA TAATCGGTGA TTTGACAGCA AAAGCAA"TAA GAAATCCGAT ATAGAATAGT   
       ATTGGTACAT ACATTCCCAT ATTAGCCACT AAACTGTCGT TTTCGTTATT CTTTAGGCTA TATCTTATCA   
  
118021 ATTTCCAGTG CTATAGGATA CGATTGATTA GCTGATGTTT CAAAATTTAA AGTT"GGTTCA TTAGAACCAT "  
       TAAAGGTCAC GATATCCTAT GCTAACTAAT CGACTACAAA GTTTTAAATT TCAACCAAGT AATCTTGGTA   
  
118091 "ATAAACCGAT ACCCAGAACT CCCATTAACA AAAAAATGGA ACCTCCCGCA GTGT"ACAAAA TAAACTTTGT   
       TATTTGGCTA TGGGTCTTGA GGGTAATTGT TTTTTTAC"CT TGGAGGGCGT CACATGTTTT ATTTGAAACA "  
  
118161 AGCTGAATAC AACCGTTTCT TTCCGCCCCA CATCGATAGA AGTAGATAAA CGGGAATTAA TT"CGAACTCC "  
       "TCGACTTATG TTGGCAAAGA AAGGCGGGGT GTAGCTAT"CT TCATCTATTT GCCCTTAATT AAGCTTGAGG   
  
118231 "CACATGATAA AAAATAGTAA GAGGTCTCGA GCAGAAAATG ATCCTATTTG ACCGCTATAC AT"TGCTAACA   
       GTGTACTATT TTTTATCATT CTCCAGAGCT CGTCTTTTAC TAGGATAAAC TGGCGATATG TAACGATTGT   
  
118301 TTAGAAAATA GAATAATCGG GAATCTCGAG TAACTGGCCA AGCCGCTAAA "GTAGCTAAAG TGGTGATAAA "  
       AATCTTTTAT CTTATTAGCC CTTAGAGCTC ATTGACCGGT TCGGCGATTT "CATCGATTTC ACCACTATTT "  
  
118371 "CCCCGTCAGT AAAATGGGTC CTATGGAAAG TCCATCGATT CCAATCTCCC" AGTAAAAATC CAAAAAAGGG   
       "GGGGCAGTCA TTTTACCCAG GATACCTTTC AGGTAGCTAA GGTTAGAGGG" TCATTTTTAG GTTTTTTCCC   
  
118441 ATCCATTTAT AATCCTCCAT CAATTGGATT AATGGATCGT CTAATTCGAA "ATGATAACAA AACGCATAGG "  
       TAGGTAAATA TTAGGAGGTA GTTAACCTAA TTACCTAGCA GATTAAGCTT TACTATTGTT TTGCGTATCC   
  
118511 "TTGTTAGAAG CAGCTCGAGT ACACAGATAC ATATAGTATA CCATCTCATT" ACTTTATTTC CTCTATGAGG   
       AACAATCTTC GTCGAGCTCA TGTGTCTATG TATATCATAT GGTAGAGTAA TGAAATAAAG GAGATACTCC   
  
118581 GAAAAAGAAA AGTAATAAAC CCGCAAATAT TGGAAAAACT ACAAC"TGTTG TTAACCAAGG AAAATAATTC "  
       CTTTTTCTTT TCATTATTTG GGCGTTTATA ACCTTTTTGA TGTTGACAAC AATTGGTTCC TTTTATTAAG   
  
118651 "GTAGTAAAAA CAAGATACAC TTGGACTAAA AAAACCCATG TTCGA"AAATG AAATAAAAAA AAATATATAT   
       CATCATTTTT GTTCTATGTG AACCTGATTT TTTTGGGTAC AAGCTTTTAC TTTATTTTTT TTTATATATA   
  
118721 ATGTATATTT ATTTTCGAGC ACGAGTTTTT GTCGGTAAAA AAGAAATCAA AT"GTATTCAA GGGGGCTTTT "  
       TACATATA"AA TAAAAGCTCG TGCTCAAAAA CAGCCATTTT TTCTTTAGTT TACATAAGTT CCCCCGAAAA "  
  
118791 "AGAGGACGTA TCAATAAGCT AGACCCATGC TTCGAGTTGT TTCATGCCAT AA"ATAAACGC GAACACTCAA   
       "TCTCCTGC"AT AGT"TATTCGA TCTGGGTACG AAGCTCAACA AAGTACGGTA TTTATTTGCG CTTGTGAGTT "  
  
118861 GAAATCCGTC GGACAGGCAG ATTCACATCT CTTACAACCA ACACAGTCTT CCGTTCGTGG AGC"CGAAGCT "  
       "CTTTAGGCAG CCT"GTCCGTC TAAGTGTAGA GAATGTTGGT TGTGTCAGAA GGCAAGCACC TCGGCTTCGA   
  
118931 "ATTTGCTTAG CTTTACATCC GCCCCAAGGT ATCATTTCTA ATACATCTGT GGGGCAAGCT CGG"ACACATT   
       TAAA"CGAATC GAAATGTAGG CGGGGTTCCA TAGTAAAGAT TATGTAGACA CCCCGTTCGA GCCTGTGTAA "  
  
119001 GAGTACACCC TATACATGTA TCATAAATCT TTACTGAATG TGACATTGGA TCTATAATTT TT"TTTAAGAC "  
       "CTCA"TGTGGG ATATGTACAT AGTATTTAGA AATGACTTAC ACTGTAACCT AGATATTAAA AA"AAATTCTG "  
  
119071 "CTAGAAATTT TCGGATCGAG TAAATTTGTA AATGAATTAT ATATTTAGAT ACCAGATGAG TC"AATAATTT   
       "GATCTTTAAA AGCCTAGCTC ATTTAAACAT TTACTTAATA TATAAATCTA TGGTCTACTC AG"TTATTAAA   
  
119141 ATCAGAATTT TGATAATAAA TCTACTTTCA GATTAACTCA TGCGAT"AGGA CCAAGATACT TTGAATTTTT "  
       TAGTCTTAAA ACTATTATTT AGATGAAAGT CTAATTGAGT ACGCTAT"CCT GGTTCTATGA AACTTAAAAA "  
  
119211 "ACGTTTTCGT AAACATTATA ATGGATTACG TATGATACAG ATAATT"TTAC TTGATGAATA TAATAATTTA   
       "TGCAAAAGCA TTTGTAATAT TACCTAATGC ATACTATGTC TATTAAA"ATG AACTACTTAT ATTATTAAAT   
  
119281 TCTCATAAAT AAATAAATAA ATACTACTTA TTCAACAAAT TCGATTGA"TT GATACGAGTT GATTTTCTGT "  
       AGAGTATTTA TTTATTTATT TATGATGAAT AAGTTGTTTA "AGCTAACTAA CTATGCTCAA CTAAAAGACA "  
  
119351 "TACGATAAGT TGACGAAAGA ATAGCTGGGC CAATAGCTGC TTCAGCGG"CT GCAATAGCTA TAACAAAAAT   
       "ATGCTATTCA ACTGCTTTCT TATCGACCCG GTTATCGACG" AAGTCGCCGA CGTTATCGAT ATTGTTTTTA   
  
119421 TGAGAAAATA TTCCCTTTTA ATTGGCGACT ATCAAAAAAA TCAGAAAATG TT"ACGAAATT CATATTAACT "  
       ACTCTTTTA"T AAGGGAAAAT TAACCGCTGA TAGTTTTTTT AGTCTTTTAC AATGCTTTAA GTATAATTGA "  
  
119491 "GCATTCAGTA TAAGCTCAAG ACACATAAGG GCCCTAACCA TATTTCGGCT CG"TGATCAAT CCATAGATAC   
       "CGTAAGTCA"T ATTCGAGTTC T"GTGTATTCC CGGGATTGGT ATAAAGCCGA GCACTAGTTA GGTATCTATG "  
  
119561 CGATAGAAAA TAAATAGGCA CTTATAATAA GTACATGTTC GAGCA"TGATT GATCAACTCC TTATCAATTC "  
       "GCTATCTTTT ATTTATCCGT G"AATATTATT CATGTACAAG CTCGTACTA"A CTAGTTGAGG AATAGTTAAG "  
  
119631 "CGATTCATTT CAATATGAAC AAAAATGTAA TAGATTCAAT TCAAT"TGACA AAAAAAGTAC AGAACAAGGG   
       "GCTAAGTAAA GTTATACTTG TTTTTACATT ATCTAAGTTA AGTTAACTG"T TTTTTTCATG TCTTGTTCCC   
  
119701 AATATATTGT TAATCGATCG AATAAAAGAA TTTTGAGTTT AGACCGAAAA "ATTCTTCAAT ATAGAATTGA "  
       TTATATAACA ATTAGCTAGC TTATTTTCTT AAAACTCAAA TCTGGCTTTT "TAAGAAGTTA TATCTTAACT "  
  
119771 "AGGGGAATGT GTGTGATAAC ATAGAACAAA GTTTAATTTA TGCTATTTCT" AACTAAAGAT TTATTACTGG   
       "TCCCCTTACA CACACTATTG TATCTTGTTT CAAATTAAAT ACGATAAAGA" TTGATTTCTA AATAAT"GACC "  
  
119841 CGAGCCACGG CAATTGCGCC GATCAAAGCA GCTAAAAGAA TGATCGAAAT GAGTTCAAAT GGAA"GAAAAA "  
       "GCTCGGTGCC GTTAACGCGG CTAGTTTCGT CGATTTTCTT ACTAGCTTTA CTCAAGTTTA CCTTCT"TTTT   
  
119911 "AATATGTTGA TAAATAAATT CCAATTTGTT GACTATTACT TATTAAATCT TGCTCTAGAA TCTG"ATTTGA   
       TTATACAACT ATTTATTTAA GGTTAAACAA CTGATAATGA ATAATTTAGA ACGAGATCTT AG"ACTAAACT "  
  
119981 TCGTGTAGTC CAAATAATAC CATACCATGA CGTATCCACA ATAGT"AGTCA TTAGTGAAAC AAAAATACTT "  
       "AGCACATCAG GTTTATTATG GTATGGTACT GCATAGGTGT TATCATCAGT AATCACTTTG TT"TTTATGAA   
  
120051 "GTACAAACCA GAAAAGTAAC TCCATTCCCA ACGGTCCAAA GATTA"AAATC TTTGGAATAT TCTGAACCCT   
       CATGTTTGGT CTTTTCATTG AGGTAAGGGT TGCCAGGTTT CTAATTTTAG AAACCTTATA AGACTTGGGA   
  
120121 TCATGAACAT CACAGCAAAT ATGATTAAAA CATTTATAGC TCCCACGTAA ATAA"GGAGTT GCGCAGCAGC "  
       AGTACTTGTA GTGTCGTTTA T"ACTAATTTT GTAAATATCG AGGGTGCATT TATTCCTCAA CGCGTCGTCG "  
  
120191 "TACAAACTGG GCGTTTGCTA GAATATAGAA TAAGGATATA GAAACAAGAA CCAG"TCCCAA CGAAAAAGCA   
       "ATGTTTGACC CGCAAACGAT C"TTATATCTT ATTCCTATAT CTTTGTTCTT GGTCAGGGTT GCTTTTTCGT   
  
120261 GAATAAATGG AGTTGTTAAA TAATAGTACT CCCATACTTC CTAATATA"AG ACCTGATCCC CACAAGACTA "  
       CTTATTTACC TCAACAATTT ATTA"TCATGA GGGTATGAAG GATTATATTC TGGACTAGGG GTGTTCTGAT "  
  
120331 "CAAGAAAATC ATGTATCGGT CCAGGCAAAT CCATTATATG TGGTAAAA"AA AGAGATAAAT AAATCTAAAT   
       "GTTCTTTTAG TACATAGCCA GGTC"CGTTTA GGTAATATAC ACCATTTTTT TCTCTATTTA TTTAGATTTA   
  
120401 GTTTTTCATG ACCTTATTGA TCTGACCAGG AAAAAAAATG TATAATCAAT TCCTAAT"TAA ATCTTAAGGG "  
       CAAAAAGTAC TGGAATAACT AGACTGGTCC TTTTTTTTAC ATATTAGTTA AGGATTAATT TAGAATTCCC   
  
120471 "GTGTGAATTA ATGTAGGTAC AGTTATTGTA TTAATCTTAG TCTTGATCTG AAAGAGT"AGA GTAGATTGAA   
       CACACTTAA"T TACATCCATG TCAATAACAT AATTAGAATC AGAACTAGAC TTTCTCATCT CATCTAACTT "  
  
120541 ACTATATATT TCGAAATATA TAGTTTCAAT CTAAATTAAG CTGCAA"TTCT CATGAATCAA TAGTTTGGAG "  
       "TGATATATA"A AGCTTTATAT ATCAAAGTTA GATTTAATTC GACGTTAAGA GTACTTAGTT ATCAAACCTC   
  
120611 "TTGTAGGTAG TGACCAAACA AACAAAACGA AAGAAACCTC ATTTCT"TTAG ATCAAAACGG AACCTTAGTA   
       AACATCCATC ACTGGTTTGT TTGTTTTGC"T TTCTTTGGAG TAAAGAAATC TAGTTTTGCC TTGGAATCAT "  
  
120681 ATTTCCAATT ACTCTTTAAT CAACAGATTT ACTTATTTTA AACTTAAATT TGA"GGCAAAT TCTAATTCAA "  
       "TAAAGGTTAA TGAGAAATTA GTTGTCTAA"A TGAATAAAAT TTGAATTTAA ACTCCGTTTA AGATTAAGTT   
  
120751 "AATTGTTCTA ATTGTATAAT CATCAACTAC TGACATCGGT AAACGACCCA AAG"AAATTTG ATTATAATTC   
       TTAACAAGAT TAACATATTA GTAGTTGATG ACTGTA"GCCA TTTGCTGGGT TTCTTTAAAC TAATATTAAG "  
  
120821 AATTCGTGAC GATCATAAGT AGAAAGTTCA TATTCTTCAG TCATTGATAA ACAATTTGTT GGACA"ATATT "  
       "TTAAGCACTG CTAGTATTCA TCTTTCAAGT ATAAGA"AGTC AGTAACTATT TGTTAAACAA CCTGTTATA"A "  
  
120891 "CAACGCAGTT ACCACAAAAT ATACAGATCC CGAAATCAAT ACTGTAATTA AGCAATCGTT TCTTT"CGAAT   
       "GTTGCGTCAA TGGTGTTTTA TATGTCTAGG GCTTTAGTTA TGACATTAAT TCGTTAGCAA AGAAAGCTT"A   
  
120961 ATCCGTTTCC AATTTCCAAT CAACAACGGG GAGATCTATA GGACATACAC GAACACATGC TTC"ACAAGCA "  
       TAGGCAAAGG TTAAAGGTTA GTTGTTGCCC CTCTAGATAT CCTGTATGTG CTTGTGTACG AAGTGTT"CGT "  
  
121031 "ATGCATTTAT CGAATTCAAA ATGGATTCGA CCGCGGAAAC GCTCCGATGC TATTAATTTT TCG"TAAGGAT   
       "TACGTAAATA GCTTAAGTTT TACCTAAGCT GGCGCCTTTG CGAGGCTACG ATAATTAAAA AGCATTC"CTA   
  
121101 ATTGAATAGT TACAGGCAAA CGATTTGCAT GGGATAAAGT AATCACGAAA CCCTGAC"CAA TGTACCTTGC "  
       TAACTTATCA ATGTCCGTTT GCTAAACGTA CCCTATTTCA TTAGTGCT"TT GGGACTGGTT ACATGGAACG "  
  
121171 "AGCTCGTACT GTTTGTTGAC CATAATTCAT GAACCCAGTT ACCATAGGGA ACATATT"GGA ATATCTCTAA   
       "TCGAGCATGA CAAACAACTG GTATTAAGTA CTTGGGTCAA TGGTATCC"CT TGTATAACCT TAT"AGAGATT "  
  
121241 AGAATTTTAT GTTTGTTTCT TTCTCTTGTT TGAGCAAGTC GTGAATATAG AATAT"GGTAT TCCTTTACAG "  
       "TCTTAAAATA CAAACAAAGA AAGAGAACAA ACTCGTTCAG CACTTATATC TTATACCATA AGG"AAATGTC   
  
121311 "TGAAAAAAGT TGAAAAGAAG TTGTTAATAA TAGATTACCG AGAGATATAG GTAAA"AGAAA TTTCCATCCG   
       ACTTTTTTCA ACTTTTCTTC AACAATTATT ATCTAATGGC TCTCTATATC "CATTTTCTTT AAAGGTAGGC "  
  
121381 AGATTTAATA GTTGATCTAT TCTTAGTCGG GGTAAAGTCC ATCTTGTTGC TATAGAAATG AACAA"GAACA "  
       "TCTAAATTAT CAACTAGATA AGAATCAGCC CCATTTCAGG TAGAACAACG" ATATCTTTAC TTGTTCTTGT   
  
121451 "AATAAGTTTT CGCTAATGTA ATAAAGATAC TAATTGTCAT TCCAAAGACT TCATACACTT TATTT"ATTTC   
       TTATTCAAAA GCGATTACAT TATTTCTATG ATTAACAGTA AGGTTTCTGA AGT"ATGTGAA ATAAATAAAG "  
  
121521 AAAAAGTTCA TAACCTAATA TGTATGGAAT AGAGATATCC CAACCACCCA AGTAAAGAAC AGTTA"CAAAT "  
       "TTTTTCAAGT ATTGGATTAT ACATACCTTA TCTCTATAGG GTTGGTGGGT TCA"TTTCTTG TCAATGTTTA   
  
121591 "AACGAAGAAA CTAATAGATT TAGATAGGAA GCAACATAAA ATAAACCAAA TTTTATACCC GAATA"CTCAG   
       TTGCTTCTTT GATTATCTAA ATCTATCCTT CG"TTGTATTT TATTTGGTTT AAAATATGGG CTTATGAGTC "  
  
121661 TTTGATAACC CGCTACTAAT TCTTCTTCTG CTTCTGGTAA ATCAA"AAGGT AATCTCTCGC ATTCTGCTAA "  
       "AAACTATTGG GCGATGATTA AGAAGAAGAC GA"AGACCATT TAGTTTTCCA TTAGAGAGCG TAAGACGATT   
  
121731 "GGAAGAAATT AGAAAAATAA CAAACCCTAT AGGTTGACGC CACAA"ATTCC AACCCCAAAA ACCATATTTT   
       CCTTCTTTAA TCTTTTT"ATT GTTTGGGATA TCCAACTGCG GTGTTTAAGG TTGGGGTTTT TGGTATAAAA "  
  
121801 GATTGAGCCT CAACTATATC AACGGTACTC GAACTGTTAG ATAAT"CATAG TCGGTAATAA CATCACTGTT "  
       "CTAACTCGGA GTTGATA"TAG TTGCCATGAG CTTGACAATC TATTAGTATC AGCCATTATT GTAGTGACAA   
  
121871 "CTCATCGCTA TTACAGAACC GTACATGAGA TTTTCACCTC ATACG"GCTCC TTGAGGGCCT TAAATAAATC   
       GAGTAGCGAT AATGTCTTGG CATGTACTCT AAAAG"TGGAG TATGCCGAGG AACTCCCGGA ATTTATTTAG "  
  
121941 TAAGGAGCGT ATCGGGATTG TTTATCTTGC TATGTCTTTT TTTGTAGATA CTATAAATAA AT"CTATCGTG "  
       "ATTCCTCGCA TAGCCCTAAC AAATAGAACG ATACA"GAAAA AAACATCTAT GATATTTATT TAGATAGCAC   
  
122011 "TTCCCCAATT AACCCAATAG AATTCTGTCT GTTATAATAA TAAAGAAAAA GTTACTTCTG AA"TGATCTCA   
       AAGGGGTTAA TTGGGTTATC TTAAGACAGA CAATATTATT ATTT"CTTTTT CAATGAAGAC TTACTAGAGT "  
  
122081 TCCTTAAAAA AAAATTTCTT TGTTGAGTAA TAACTTAATT CTTCACTAAA ATACTATTTA TTAT"AAATAT "  
       "AGGAATTTTT TTTTAAAGAA ACAACTCATT ATTGAATTAA GAAG"TGATTT TATGATAAAT AATATTTATA   
  
122151 "CAATATTATT ATAAATATCA ATAACCCATC GTTTTCAATT ACGAAAAAAA AAAAAAAATT GGCT"ATTCCA   
       GTTATAATAA TATTTATAGT TATTGGGTAG CAAAAGTTAA TGCTTTTTTT "TTTTTTTTAA CCGATAAGGT "  
  
122221 TTAGTTCATG AATTCGGACA TGAATTCTAT CTCTCTGAAT AGGGAGATAG CAAA"GAAATG CATATAGTAA "  
       "AATCAAGTAC TTAAGCCTGT ACTTAAGATA GAGAGACTTA TCCCTCTATC" GTTTCTTTAC GTATATCATT   
  
122291 "TGGAGATAAG AAACAATAAA AAATATCTCT TTTTTGTTGT AATTGGATTC TTTC"CATTTT TTTTCGTTCC   
       ACCTCTATTC TTTGTTATTT TTTATAGAGA AAAAACAACA T"TAACCTAAG AAAGGTAAAA AAAAGCAAGG "  
  
122361 TATTCTTCTT TCTGAGAAAA AGGGTACTTA CTAAATAAGG GATTATT"TCG TTTCGGATAG TCATTTATTT "  
       "ATAAGAAGAA AGACTCTTTT TCCCATGAAT GATTTATTCC C"TAATAAAGC AAAGCCTATC AGTAAATAAA   
  
122431 "AATGGGTGGA TAGGCGTATA CTCTGGATCG GAATAGTGGG GAGTACT"GCC TGATCATTTC TACAAACTTA   
       TTACCCACCT ATCCGCATAT GAGACCTAGC CTT"ATCACCC CTCATGACGG ACTAGTAAAG ATGTTTGAAT "  
  
122501 AAGCCCCAAT TCGTATTCGT TTTATATTAT GCATTTTTTC AAAAATGTCC T"TCTCTCTCT TTTGGATAAT "  
       "TTCGGGGTTA AGCATAAGCA AAATATAATA CGT"AAAAAAG TTTTTACAGG AAGAGAGAGA AAACCTATTA   
  
122571 "TTTGAAAATC TCCATTACTA ATCCTTTGTA TATCTTGGTG TTTCTAACCA T"CCACTCATT TTTGCTCAAT   
       AAACTTTTAG AGGTAATGAT TAGGAAACAT ATAGAACCAC AAA"GATTGGT AGGTGAGTAA AAACGAGTTA "  
  
122641 CGGCTGTGTT ATGGTAATAC ACATATGGTA GTACATAGAG ATAATAGTGA AAACT"CAAAC CGGTTGATCT "  
       "GCCGACACAA TACCATTATG TGTATACCAT CATGTATCTC TAT"TATCACT TTTGAGTTTG GCCAACTAGA   
  
122711 "TTTGAGTCCG CTTCAAGACA GGATAGACCC AATCTTGGGG CTCTCTTGCG CCTAT"ATTTT TTTCTGCTTA   
       AAACTCAGGC GAAGTTCTGT CCTATCTGGG "TTAGAACCCC GAGAGAACGC GGATATAAAA AAAGACGAAT "  
  
122781 ACTCTTATAC ATAGAAAATG AGACTCAATA TTTTGACTGC TAATTTTTAT TTATATA"AGC TGTTTTCTTT "  
       "TGAGAATATG TATCTTTTAC TCTGAGTTAT" AAAACTGACG ATTAAAAATA AATATATTCG ACAAAAGAAA   
  
122851 "CACTCATATA ACTATCTGAT TTAGTTCATT AATCCAAATA ATAAATATAA AAATGAG"GAT ATCTTTTCAA   
       GTGAGTATAT TGATAGACTA AATCAAGTAA TTAGGTTTAT TATT"TATATT TTTACTCCTA TAGAAAAGTT "  
  
122921 CTGCATTTCA ACCCTTTTCT CGAAAAAAAG TGGGAGAAGT TTATGTCTCA ACGAATCACA "CGTAGAGATA "  
       "GACGTAAAGT TGGGAAAAGA GCTTTTTTTC ACCCTCTTCA AATA"CAGAGT TGCTTAGTGT GCATCTCTAT   
  
122991 "TTGATAATAC ACATAGAGTT AATGGTATTT CATAACTAAT TGATTGAGCA GCAGCTCGTA" GACCACCTAA   
       AACTATTATG TGTATCTCAA TTACCATAAA GTATTGATTA A"CTAACTCGT CGTCGAGCAT CTGGTGGATT "  
  
123061 GAAGGAATAT TTATTATTGG ATCCATATCC TGACATAAGA AGTCC"GATGG GAGCAACACT TGAAATGGCA "  
       "CTTCCTTATA AATAATAACC TAGGTATAGG ACTGTATTCT T"CAGGCTACC CTCGTTGTGA ACTTTACCGT   
  
123131 "ATCCATAAAA AAACACCGAT AGGGAGATCG GCTAAAACAA GGCGA"TAACC AAAAGGAATT ACTGAATAGC   
       TAGGTATTTT TTTGTGGCTA TCCCTCTAGC CGATTTTGTT "CCGCTATTGG TTTTCCTTAA TGACTTATCG "  
  
123201 TTAGTAAAAT TGATATGACT GCTATGGATG GTCCGATACT GAATAAACGA GTATCACCTC TAGAT"GGAAG "  
       "AATCATTTTA ACTATACTGA CGATACCTAC CAGGCTATGA" CTTATTTGCT CATAGTGGAG ATCTACCTTC   
  
123271 "CAAATTTTCT TTAAAAAGTA GTTTTGTACC ATCTGCTAAA GCTTGAAGAA CTCCCAAAGG ACCAG"CATAT   
       GTTTAAAAGA AATTTTTCAT CAAAACATGG TAGAC"GATTT CGAACTTCTT GAGGGTTTCC TGGTCGTATA "  
  
123341 TCAGGTCCAA TCCGTTGTTG TATTCCTGCG GATATTTCTC TTTCTAACCA TACAATTACT "AGTACGCCTA "  
       "AGTCCAGGTT AGGCAACAAC ATAAGGACGC CTATA"AAGAG AAAGATTGGT ATGTTAATGA TCATGCGGA"T "  
  
123411 "TGGTGATTCC AATACAGTAG TCCACATAGG GACAGTACCA TAGATTCATA GAATCTTTAG" AATCCATCTC   
       "ACCACTAAGG TTATGTCATC AGGTGTATCC CTGTCATGGT ATCTAAGTAT CTTAGAAATC TTAGGTAGA"G   
  
123481 GAAAAGAATG GATATCTGGG ACTGTGATGT ATCAATTATC ATTTTAA"CGA TCAACTTCTC CCATAATGAT "  
       CTTTTCTTAC CTATAGACCC TGACACTACA TAGTTAATAG TAAAATTGCT AGTTGAAGAG GGTATTACTA   
  
123551 "ATCTATGCTA CCTAGTATTG TCATAATATC AGCCAATTTC ATTCTTT"TAA CTAACTGAGG AAGAATTTGC   
       TAGATACGAT GGATCATAAC AGTATTATAG TCGGTTAAAG TAAGAAAATT GATTGACTCC TTCTTAAACG   
  
123621 AAATTGATAA AACCCGGCGG GCGAATTTTC CATCTCCAAG GAAAA"CCACC CTGATCCCCT ATCAAAAAAA "  
       TTTAACTATT TTGGGCCGCC CGCTTAAAAG GTAGAGGTTC CTTTT"GGTGG GACTAGGGGA TAGTTTTTTT "  
  
123691 "TGCCCAATTC CCCTTTGGGG GCTTCGACTC TCACATAAAG TTCTT"GTTTC GCCAATTCAA AAGTTGGCGA   
       "ACGGGTTAAG GGGAAACCCC CGAAGCTGAG AGTGTATTTC AAGAA"CAAAG CGGTTAAGTT TTCAACCGCT   
  
123761 AGGTTTTTTA CTAATGAATC GATAGTCAAA GTCATTCCAT TCCGGAGACC TT"GCTCGATC AAAAGATCGT "  
       TCCAAAAAAT GATTACTTAG CTATCAGTTT CAGTAAGGTA AGGCCTCTGG AACGAGCTAG TTTTCTAGCA   
  
123831 "ATTTCTAAAT TTTCATAAGG TCCCCCTGGA ATTCCTTCCA AAGCTTGTTG AA"TAATTTTT ACGGATTCCG   
       TAAAGATTTA AAAGTATTCC AGGGGGACCT TAAGGAAGGT TTCGAACAAC TTATTAAAAA TGCCTAAGGC   
  
123901 TCATCTCAGC AAGTCTGACT AAATAACGAG CTAATGAATC GCCTTCTTTT T"GCCACTGAA CTTCCCAATC "  
       AGTAGAGTCG TTCAGACTGA TTTATTGCTC GATTACTTAG CGGAAGAAAA ACGGTGACTT GAAGGGTTAG   
  
123971 "AAATTCGTCA TAACACTCAT AACGATCAAC TTTACGAAGA TCCCATGGTA T"TCCGGAAGC TCGCAGCATT   
       TTTAAGCAGT ATTGTGAGTA T"TGCTAGTTG AAATGCTTCT AGGGTACCAT AAGGCCTTCG AGCGTCGTAA "  
  
124041 GGTCCTGATA ACCCCCAATT TATGACCTCT TCTCCAGAAA TAATACCTAC T"CCCTCAACT CGTTCTAAAA "  
       "CCAGGACTAT TGGGGGTTAA A"TACTGGAGA AGAGGTCTTT ATTATGGATG AGGGAGTTGA GCAAGATTTT   
  
124111 "AAATAGGATT TTGTGTAATA AGTTTTTGAT ATTCAGCAAC CGCTGTCAAA A"AATAATCGC AGAAATCCAA   
       TTTATCCTAA AACACATTAT TCAAAAACTA TAAGTCGTTG GCGACAGTTT TTTATTAGCG TCTTTAGGTT   
  
124181 ACATTTATCT ATCCATCCAT GAGGTAAATC AGCCGCGACT CCCCCGATAC GAAAAAAATT ATGCA"TCATT "  
       TGTAAATAGA TAGGTAGGTA CTCCATTTAG TCGGCGCTGA GGGGGCTATG CTTTTTTTAA TACGTAGTAA   
  
124251 "CTCATACCGG TAGCTGCTTC GAATAGATCA TATACTAATT CTCGTTCTCT GAAAATATAG AAGAA"GGGAG   
       GAGTATGGCC ATCGACGAAG CTTATCTAGT ATATGATTAA GAGCAAGAGA CTTTTATATC TTC"TTCCCTC "  
  
124321 TCTGTGCGCC AATATCCGCC ATAAAAGGGC CAAGCCATAA CAGATGAGAA GCTATACGAC TCA"ACTCCAA "  
       "AGACACGCGG TTATAGGCGG TATTTTCCCG GTTCGGTATT GTCTACTCTT CGATATGCTG AGT"TGAGGTT   
  
124391 "CATAATTACT CTGATATAGC TGGCTCTTTT AGGGACTTGA ATATTTCCCA ACTGTTCTGG ACC"ATTTACG   
       GTATTAATGA GACTATATCG ACCGAGAAAA TCCCTGAACT TATAAAGGGT TGACAAGACC TGGTAAATGC   
  
124461 GTTATTGCTT CTGTAAACAT AGTAGCTAAA TAATCCCAAC GTGTT"ACATA AGGTAGATAT TGTATAATTG "  
       CAATAACGAA GACATTTGTA TCATCGATTT ATTAGGGTTG CACAATGTAT TCCATCTATA ACATATTAAC   
  
124531 "TTCGGTTTTC TGCAATTTTT TCCATCCCCC TGTGTAAATA ACCCA"ATATT GGTTCACAGT CAATAACATC   
       AAGCCAAAAG ACGTTAAAAA AGGTAGGGGG ACACATTTAT TGGGTTATAA CCAAGTGTCA GTTATTGTAG   
  
124601 TTCACCATCC AAAGTAAGGA TGAGTCGAAG AACACCGTGC ATTGA"GGGGT GGTGAGGTCC CATATTGACT "  
       AAGTGGTAGG TTTCATTCCT ACTCAGCTTC TTGTG"GCACG TAACTCCCCA CCACTCCAGG GTATAACTGA "  
  
124671 "ATCATGAGGT CTTTTCTTGT AGCTGGTCCA TTCATAAGTT TTTCC"TCGAT TCATCTTTCC ATGAATTGCT   
       "TAGTACTCCA GAAAAGAACA TCGACCAGGT AAGTA"TTC"AA AAAGGAGCTA AGTAGAAAGG TACTTAACGA "  
  
124741 GAAAATGAAA ATAAGTTCAT AAAAATTCAA GATCTAATAA ATCAAATAAT TCAA"AATTAC TTTTCAAATT "  
       "CTTTTACTTT TATTCAAGTA TTTTTAAGTT CTAGATTA"TT TAGTTTATTA AGTTTTAATG AAAAGTTTAA   
  
124811 "ACTGAGTTTT TGACTCCCGA ATATCCAATT GATTAATTAA TTCTTTATAA CGCA"TTTTAT TTTTCTTTGA   
       T"GACTCAAAA ACTGAGGGCT TATAGGTTAA CTAATTAATT AAGAAATATT GCGTAAAATA AAAAGAAACT "  
  
124881 TAAATAAGAA AGTAATCGTT GGCGTTTTCC GAGAATTTTA CGTAGACCTC TTT"GAGATAA ATAGTCTTTT "  
       "A"TTTATTCTT TCATTAGCAA CCGCAAAAGG CTCTTAAAA"T GCATCTGGAG AAACTCTATT TATCAGAAAA "  
  
124951 "TTGTGCAATT CCAAATGTGA AGTAAGTCTT CGTATCTTAT TGGTGAAACT GAC"TACTTGA AATTCAACGG   
       "AACACGTTAA GGTTTACACT TCATTCAGAA GCATAGAAT"A ACCA"CTTTGA CTGATGAACT TTAAGTTGCC "  
  
125021 ATCCTCCATT TTCTTTTTTT TCTTCTTGCG AAATAACTGA GCTGAATGAA TTTT"TTACCA TAAAATGAAA "  
       "TAGGAGGTAA AAGAAAAAAA AGAAGAACGC TTTATTGACT CGAC"TTACTT AAAAAATGGT ATTTTACTTT   
  
125091 "TCTCCTTAGC CTCCTTTTTT TATTTTTTTT ATAAATTATT AGTGATCAGT AATA"ATAATG TTACTCATTT   
       AGAGGAATCG GAGGAAAAAA ATAAAAAAAA TATTTAATAA TCACTAGTCA TTATTATTAC AATGAGTAAA   
  
125161 TAATTTGATA TACACAATAA TCTTACTTTG ATTAAGAATT TCTTTTTTTT TTTTTTATAA AAT"TTAGCAA "  
       ATTAAACTAT ATGTGTTATT AGAATGAAAC TAATTCTTAA AGAAAAAAAA AAAAAATATT TTA"AATCGTT "  
  
125231 "TTCAATTTTT GAAAATTGTA TTTAGATAGG TATACAAAAG TCTGGTATAT TTCTGCATGC ACA"AAAAATA   
       "AAGTTAAAAA CTTTTAACAT AAATCTATCC ATATGTTTTC AGACCATATA AAGACGTACG TGT"TTTTTAT   
  
125301 TTTAGACTGC AAATTTGAAT TTAAAATGAA ATAAGAATAT TTTCT"GGATT CATTTCTAAA TCGAATAGAT "  
       AAATCTGACG TTTAAACTTA AATTTTACTT TATTCTTATA AAAGA"CCTAA GTAAAGATTT AGCTTATCTA "  
  
125371 "ACGTCATTGA ATTAATATCG TCTATCAGAA TCTAAGAGAG TGCCA"TATGT GTATTTCTTT GTCCTCATAT   
       "TGCAGTAACT TAATTATAGC AGATAGTCTT AGATTCTCTC ACGGT"ATACA CATAAAGAAA CAGGAGTATA   
  
125441 ACCATATATG GTACGGGCAA TATAGCAAAA ATGTAGCATT AACGAATT"TT CAATTGTGGA TACATATGGA "  
       TGGTATATAC CATGCCCGTT ATATCGTTTT TACATCGTAA TTGCTTAAAA GTTAACACCT ATGTATACCT   
  
125511 "TCCTTAGCAT ACTAAAACGA CTGCTATTAT TGGTATCAAA CCAATAAC"GA TTCATACAAG CTAGATTTTC   
       AGGAATCGTA TGATTTTGCT GACGATAATA ACCATAGTTT GGTTATTGCT AAGTATGTTC GATCTAAAAG   
  
125581 TAATCGATAA TTGGGCCAAA GAAATAACTT TAAATTTTTT AGTTGATTTT GATATCTAT"C AAGATGTTTG "  
       ATTAGCTATT AACCCGGTTT CTTTATTGAA ATTTAAAAAA TCAACTAAAA CTATAGATAG TTCTACAAAC   
  
125651 "CTTCTTTTAT CCAAAACTTG ATCATCTGTT TTCACCTTAT TTCCATCGTC AAATGCTGT"A TTTCTATGGA   
       GAAGAAAATA GGTTTTGAAC TAGTAGACAA AAGTGGAATA AAGGTAGCAG TTTACGACAT AAAGATACCT   
  
125721 TATCATTTTT ATTCCCAGAA TTGAAACAAA TTAGAATTCT AAACTCTCT"C CGACCTCTAG GGGATAAGAT "  
       ATAGTAAAAA TAAGGGTCTT AACTTTGTTT AATCTTAAGA TTTGAGAGAG GCTGGAGATC CCCTATTCTA   
  
125791 "ATTTTCCGGA ACAAGCAAAT CATAATGATT GCTGGTTCTA TTTTCAGTC"C TTTTTTGATG TATTGCAATG   
       TAAAAGGCCT TGTTCGTTTA GTATTACTAA CGACCAAGAT AAAAGTCAGG AAAAAACTAC ATAACGTTAC   
  
125861 GATTCAGCAA AACTCCTCTT ATAAGGATAG CCTTTTTCTT GATATCTTT"G ATTCAATTGG TACTTATTCT "  
       CTAAGTCGTT TTGAGGAGAA TATTCCTATC GGAAAAAGAA CTATAGAAAC TAAGTTAACC ATGAATAAGA   
  
125931 "TCTGAACTAA TGAAATACCT ATGGTTTGAG ACATAATAAA TTTTCCATC"A TTTTTTAGAG ACAGACGAAC   
       AGACTTGATT ACTTTATGGA TACCAAACTC TGTATTATTT AAAAGGTAGT AAAAAATCTC TGTCTGCTTG   
  
126001 GGGTTCGATA ATAAATATTC CCTTTTCAAG TAACTCGTGA AAAGTTA"TAT CGTCCGACGG AGGTAGCAGG "  
       CCCAAGCTAT TATTTATAAG GGAAAAGTTC ATTGAGCACT TTTCAATATA GCAGGCTGCC TCCATCGTCC   
  
126071 "AACTCCAGAC TGAATTCGTT ATTGCGAATA GCGGCTAGAC TAAGTTT"TTT GAGATCTAGC CGTCTAAGCA   
       TTGAGGTCTG ACTTAAGCAA TAACGCTTAT CGCCGATCTG ATTCAAAAAA CTCTAGATCG GCAGATTCGT   
  
126141 TGAAAGAAAA TCCGTTGAGG TTCATGAAAA GGCTTTTACT TATTT"CGGGA TTAGGCCAGT TCAATTGAAA "  
       ACTTTCTTTT AGGCAACTCC AAGTACTTTT CCGAAAATGA ATAAAGCCCT AATCCGGTCA AGTTAACTTT   
  
126211 "ACGAAAATGG CCTGTTCGGA GTACACCAAG CTTCCCTTCG ATCTT"TTTCT TGGATTGCTT TTTCTTTATA   
       TGCTTTTACC GGACAAGCCT CATGTGGTTC GAAGGGAAGC TAGAAAAAGA ACCTAACGAA AAAGAAATAT   
  
126281 CCTTTTTTCA CATCTGCTCC CGCAGAATCT TTTTGAAGAT TTTTTGT"GTG GTTTGAGAGA ACTGAGTCAA "  
       GGAAAAAAGT GTAGACGA"GG GCGTCTTAGA AAAACTTCTA AAAAACACAC CAAACTCTCT TGACTCAGTT "  
  
126351 "CACCCTCTTT GGCCACAGAT TCTTTTTTTG CTTTATTTCC ATTTTTC"TTT TCAATGAGTT TTTTTTTACT   
       "GTGGGAGAAA CCGGTGTC"TA AGAAAAAAAC GAAATAAAGG TAAAAAGAAA AGTTACTCAA AAAAAAATGA   
  
126421 AAAAATTTCA TTTTCATTCA AATCGAAAAG AAGTGATTTG ATTGGTATCA TCCATGGCTT AA"TCTTATAT "  
       TTTTTAAAGT AAAAGTAAGT TTAGCTTTTC TTCACTAAAC TAACCATAGT AGGTACCGAA TTAGAATATA   
  
126491 "GCATTATAAA GTATCAAAAA TTTTGGAAAG AACCACTTAG GGTTCGAATT CGATCTGTAA CG"ACTTATTT   
       CGTAATATTT CATAGTTTTT AAAACCTTTC TTGGTGAATC CCAAGCTTAA GCTAGACATT GCTGAATAAA   
  
126561 TTTTTTCACT CATTCTCATC CAATCAAAAA AGATTTGTTT TTTATTGTTG G"ATGGGTTGC TTTTTTTATC "  
       AAAAAAGTGA GTAAGAGTAG GTTAGTTTTT TCTAAACAAA AAATAACAAC CTACCCAACG AAAAAAATAG   
  
126631 "TTGATTAATT GTGAGATAAA ACAATTCTTT CTTTTTTATT TTTCTAATTA T"TTTCTTATT TATTTTTTGA   
       AACTAATTAA CACTCTATTT TGTTAAGAAA GAAAAAATAA AAAGATTAAT AAAAGAATAA ATAAAAAACT   
  
126701 ATTAGTTCAA AATTCTTAAC CCCATTTTGA GTATTTTTTT TACTGT"TCGT GTCAGACTCA TTATGTCTCC "  
       TAATCAAGTT TTAAGAATTG GGGTAAAACT CATAAAAAAA ATGACAAGCA CAGTCTGAGT AATACAGAGG   
  
126771 "AGACTCGTAT ATCGCCCTTA TTTTTAATAG AAAAATGCAG CATTCT"CCAA TCAAAATATT TTCTATCCAT   
       TCTGAGCATA TAGCGGGAAT AAAAATTATC TTTTTACGTC GTAAGAGGTT AGTTTTATAA AAGATAGGTA   
  
126841 AGTTTTTTCC AGACCTCTAA TATTAGCTTT CACTGGATAA TTATTTTTCA CTAGATATTT AT"CGATAGGG "  
       TCAAAAAAGG TCTGGAGATT ATAATCGAAA GTGACCTATT AATAAAAAGT GATCTATAAA TAGCTATCCC   
  
126911 "AGATCAGTCA GTATATCAAA AAATTTGCAT TTATCCTTGT TGTACTTATA ACAACTTTCT TG"ATTATTTT   
       TCTAGTCAGT CATATAGTTT TTTAAACGTA AATAGGAACA ACATGAATAT TGTTGAAAGA ACTAATAAAA   
  
126981 TTACTTGTAC TGGTAATTCA TAAATATATG AATCTTTATT ATCTTCATAA TTAATAGATT TATAT"GATAA "  
       AATGAACATG ACCATTAAGT ATTTATATAC TTAGAAATAA TAGAAGTATT AATTATCTAA ATATACTATT   
  
127051 "AGAATCATAT ATATATTTTT TTTTTTTTTT AGTCGGTAAT GAGTAGTGTG TTTCAAAAGG ATTTT"GTTTT   
       TCTTAGTATA TATATAAAAA AAAAAAAAAA TCAGCCATTA CTCATCACAC AAAGTTTTCC TAAAACAAAA   
  
127121 TTGTAATCAA TTAATCCTTT TTTTTCATAT GAATCACATT GGTTAAAATC TTTATTTTTA C"GATCTTGCT "  
       AACATTAGTT AATTAGGAAA AAAAAGTATA CTTAGTGTAA CCAATTTTAG AAATAAAAAT GCTAGAACGA   
  
127191 "TGACTCTATT TCGCCATTTT TGGGAGAGTA ATTTTGACCA TCTAATCGGA TATAAATCGA A"GTTATAATG   
       ACTGAGATAA A"GCGGTAAAA ACCCTCTCAT TAAAACTGGT AGATTAGCCT ATATTTAGCT TCAATATTAC "  
  
127261 ACCCCGTAAC CAGTTTTTCC ATGGATTTAT CATTCCAGAA TTTTCAAGAT TCTTTTGTTT "CTTTTGTTTT "  
       "TGGGGCATTG G"TCAAAAAGG TACCTAAATA GTAAGGTCTT AAAAGTTCTA AGAAAACAAA GAAAACAAAA   
  
127331 "AGATCGGAAT GTAATATTTC GTGTGCTCCA ACAACTTCAA CAAAATAATC CTGTGTTTCA" TTCTTAAGAA   
       TCTAGCCTTA CATTATAAAG CACACGAGGT TGTTGAAGTT GTTTTATTAG GACACAAAGT AAGAATTCTT   
  
127401 AACGAGATGT TCCGTGATAT TGAAAGACAG GTCTTAACTT AGATAAGTTA ATAATTTTTG TTTGT"GATAA "  
       TTGCTCTACA AGGCACTATA ACTTTCTGTC CAGAATTGAA TCTATTCAAT TATTAAAAAC AAACACTATT   
  
127471 "TTTGTAAAAT AAATATGCTT GCGACAAGTA TGATACGTCC CAAAACATCT TTAGATTCTG ATTCT"TTACA   
       AAACATTTTA TTTATACGAA CGCTGTTCAT ACTATGCAGG GTTTTGTAGA AATCTAAGAC TAAGAAATGT   
  
127541 TTCTTATTAG TAATATTCGA AAGTAACTTT TTGATAGTTG AAATAATTAT ACTTTGATT"T GGTTTATCAA "  
       AAGAATAATC ATTATAAGCT TTCATTGAAA AACTATCAAC TTTATTAATA TGAAACTAAA CCAAATAGTT   
  
127611 "TTCTTTCTTG ATTTGCTTCA TTATTGAAAA AGGATTTTGT AATTTTTGAA TCTTGGCCA"T TAATCCTCGA   
       AAGAAAGAAC TAAACGAAGT AATAACTTTT TCCTAAAACA TTAAAAACTT AGAACCGGTA ATTAGGAGCT   
  
127681 CATATTAATG ATACCTTGAA AGATATCTAT GTATATGTTT TGAACAAAAA ATTTTAGAAA CTTAT"ACGAT "  
       GTATAATTAC TATGGAACTT TCTATAGATA CATATACAAA ACTTGTTTTT TAAAATCTTT GAATATGCTA   
  
127751 "TTACGAATAA ATCGTACATT TCTTTTTTTT AATATCTTTA AAATATTTTT CTGAGATTCA AATAT"TTTAT   
       AATGCTTATT TAGCATGTAA AGAAAAAAAA TTATAGAAAT TTTATAAAAA GACTCTAAGT TTATAAAATA   
  
127821 CATAATAACT CATTTTGTTA GGACTAATAT TTATTTTTGT ATTTAGCAA"C TCTTTTTGCC TGTCTTTTTT "  
       GTATTATTGA GTAAAACAAT CCTGATTATA AATAAAAACA TAAATCGTTG AGAAAAACGG ACAGAAAAAA   
  
127891 "CATTTTTTCA ATTATTTTTA GCATGGTGTT TATTCTATCA GTCAACTCT"T TCATCTTTCG TTCTCTCAAT   
       GTAAAAAAGT TAATAAAAAT CGTACCACAA ATAAGATAGT CAGTTGAGAA AGTAGAAAGC AAGAGAGTTA   
  
127961 GACAAAATTA TCGAATCTAT AGACCCAGAC GAGCTTTGGA TCATTCCATT ACTTATTATT "GAATTGTTTT "  
       CTGTTTTAAT AGCTTAGATA TCTGGGTCTG CTCGAAACCT AGTAAGGTAA TGAATAATAA CTTAACAAAA   
  
128031 "CTTTTTTAGC TTCAGTCAAC TTGTATATTT CTTTCAATTG CAATAATAGA ATTGGGTTTC" TTTGCCAGAA   
       GAAAAAATCG AAGTCAGTTG AACATATAAA GAAAGTTAAC GTTATTATCT TAACCCAAAG AAACGGTCTT   
  
128101 TTCTCTTAGT CTTTTTTTTA TAAATAAAAT GGGTTTGATG ACCCATTTTT CTTT"TGCAAT ATTAATTTTT "  
       AAGAGAATCA GAAAAAAAAT ATTTATTTTA CCCAAACTAC TGGGTAAAAA GAAAACGTTA TAATTAAAAA   
  
128171 "CTAAAAACCT TTTTTCTTAA ACGTAAAAAT ATTCGAAAAT ACTTCGTTCT CCAT"TTTTTT AATTGTTGTT   
       GATTTTTGGA AAAAAGAATT TGCATTTTTA TAAGCTTTTA TGAAGCAAGA GGTAAAAAAA TTAACAACAA   
  
128241 TTTTTTTTTT TATGAACTCT CTTAACTCTA TTAAGTCGGA TTTAAAAAA"C AAGAATGGGT TTTCGGCAGA "  
       AAAAAAAAAA ATACTTGAGA GAATTGAGAT AATTCAGCCT AAATTTTTTG TTCTTACCCA AAAGCCGTCT   
  
128311 "CCCAAAAGGC TGTTCAGTTT CCGTTCCAAA AACTGTTAAA AAACAAAAA"T CCTTTTTTTC TATCTTTTTT   
       GGGTTTTCCG ACAAGTCAAA GGCAAGGTTT TTGACAATTT TTTGTTTTTA GGAAAAAAAG ATAGAAAAAA   
  
128381 GATCTGTGCC ACGATTGCAG ACGAAAAGGA AATAAGATCT TTATTTGAAT AC"CGTCTGTT AACCAATTTT "  
       CTAGACACGG TGCTAACGTC TGCTTTTCCT TTATTCTAGA AATAAACTTA TGGCAGACAA TTGGTTAAAA   
  
128451 "GGGGAAATTC GTTTTCTGCT AATGGAATAG CATTATATGT GCACTTTACA TA"TATTTCTT TTTTCCAATT   
       CCCCTTTAAG CAAAAGACGA TTACCTTATC GTAATATACA CGTGAAATGT ATATAAAGAA AAAAGGTTAA   
  
128521 TTTAAAATCC TCAAAAAAGT GAGGAGGTGA CAATAATAGT ATACGGGTAA "GGTTGGCAGC TATTATCACT "  
       AAATTTTAGG AGTTTTTTCA CTCCTCCACT GTTATTATCA TATGCCCATT CCAACCGTCG ATAATAGTGA   
  
128591 "GAGGGTAAGA TAATATATTT TCTCAAAAAT GCTTGAGTTA TTAACAGAGA" ACCTCTTACT AGTTGACCAA   
       CTCCCATTCT ATTATATAAA AGAGTTTTTA CGAACTCAAT AATTGTCTCT TGGAGAATGA TCAACTGGTT   
  
128661 ATCGTATGCT CTCCCAGAGT TCTGCTATTT GTATATGTTT TTCTT"CCTTC AGATTTATCG ATTCTTGATT "  
       TAGCATACGA GAGGGTCTCA AGACGATAAA CATATACAAA AAGAAGGAAG TCTAAATAGC TAAGAACTAA   
  
128731 "TTTTTCGTTT TTCAGATCTT CCCTTGTCTT TTCTTCTCCA TTATT"CGAAA TAGGCAATTC TGTGTTTGTC   
       AAAAAGCAAA AAGTCTAGAA GGGAACAGAA AAGAAGAGGT AATAAGCTTT ATCCGTTAAG ACACAAACAG   
  
128801 CGTATCCGAA GTCTATCAAG TTTTTCCATT AGTCTAGGAA TATAAATACA AAAATCAGAA AAAAA"AAAAT "  
       GCATAGGCTT CAGATAGTTC AAAAAGGTAA TCAGATCCTT ATATTTATGT TTTTAGTCTT TTTTTTTTTA   
  
128871 "TGTGTCTGCC CAAGAAAAGA TAAGAATTTG CACCTTGAAA CAGTTGATAC ATACCCATTT TACGT"CGTTG   
       ACACAGACGG GTTCTTTTCT ATTCTTAAAC GTGGAACTTT GTCAACTATG TATGGGTAAA ATGCAGCAAC   
  
128941 AGCACGCATA GAGCCTTTGA TTATCTCTCG ACGAAAATCC CATTGCTTGG "CATAGCGTAT AATAGCCTGT "  
       TCGTGCGTAT CTCGGAAACT AATAGAG"AGC TGCTTTTAGG GTAACGAACC GTATCGCATA TTATCGGACA "  
  
129011 "TCCATTGGTT CGTCCTTTGG TTCGTCCTTC GACTTAATCT CATTAGTCGA" AGTCGAAGTC GAAGCAGGAA   
       "AGGTAACCAA GCAGGAAACC AAGCAGG"AAG CTGAATTAGA GTAATCAGCT TCAGCTTCAG CTTCGTCCTT   
  
129081 TAGAAGTTGA AGTTGAAGTC GAAGTCGAAG TCGAAGCAGG AATAGAAGTT GAA"GTCGAAG CAGGAATAAA "  
       ATCTTCAACT TCAACTTCAG CTTCAGCTTC AGCTTCGTCC TTATCTTCAA CTTCAGCTTC GTCCTTATTT   
  
129151 "AGTTGAAGTC GAAGTCGAAG CAGGAATAGA AGTTGAAGTC GAAGTCGAAG TCG"AAGCAGG AATAGAAGTC   
       TCAACTTCAG CTTCAGCTTC GTCCTTATCT TCAACTTCAG CTTCAGCTTC AGCTTCGTCC TTATCTTCAG   
  
129221 GAAGTCGAAG CAGGAATAGA AGCAGGAGTA GAAGTAGAAG TAGAAGTAGG AGTAGAAGTA GGAG"TAGAAG "  
       CTTCAGCTTC GTCCTTATCT TCGTCCTCAT CTTCATCTTC ATCTTCATCC TCATCTTCAT CCTCATCTTC   
  
129291 "TAGGAGTAGA AGTAGGAGTA GAAGTAGAAG TAGGAGTAGA AGTAGGAGTA GAAGTAGGAG TAGA"AGTAGA   
       ATCCTCATCT TCATCCTCAT CTTCATCTTC ATCCTCATCT TCATCCTCAT CTTCATCCTC ATCTTCATCT   
  
129361 AATCTTCTTA ATTTTGTTAA GGTCACTTAA AATTACTACG CGTTTGCATT TTCTTGAACG AATT"TGATAA "  
       TTAGAAGAAT TAAAACAATT CCAGTGAATT TTAATGATGC GCAAACGTAA AAGAACTTGC TTAAACTATT   
  
129431 "TCTAACACGA GCTCTCCATC GGTTATGTCG TACACTTGTT CTATCTCCTT GATTAACTTG TATG"ACCAGC   
       AGATTGTGCT CGAGAGGTAG CCAATACAGC ATGTGAACAA GATAGAGGAA CTAATTGAAC ATACTGGTCG   
  
129501 GAGGAACTTT TTTACTGATT TCTGTTAGGT CAATCGAGTT TTGAT"CACTT GCGTTAGCCT TGTAAGGAAT "  
       CTCCTTGAAA AAATGACTAA AGACAATCCA GTTAGCTCAA AACTAGTGAA CGCAATCGGA ACATTCCTTA   
  
129571 "TGTAACTCCA TCGAAGAAAC CGTTGTTATT ATTACGAATG ATAGC"ATGAA TCTTATTTGT CCAAATGTTC   
       ACATTGAGGT AGCTTCTTTG GCAACAATAA TAATGCTTAC TATCGTACTT AGAATAAACA GGTTTACAAG   
  
129641 TCACTATCAT TTTTTCTAGA AGTTTCATTT AGCATTGAGA ATCCT"AAAAG TAAATTGATT CGTCTCCGGT "  
       AGTGATAGTA AAAAAGATCT TCAAAGTAAA TCGTAACTCT TAGGATTTTC ATTTAACTAA GCAGAGGCCA   
  
129711 "AAGGACCACT CAAGAACGGA TCATATATTT TTGGTAAATA TTCTT"TTTTA GTTTCATCAT TACATATACA   
       TTCCTGGTGA GTTCTTGCCT AGTATATAAA AACCATTTAT AAGAAAAAAT CAAAGTAGTA ATGTATATGT   
  
129781 TAATTGAGTC CTTTTTTGCA GTACATCTCC AACTAGAGTT GGGGATCCCT TATC"GAGAAG TAGAATTCTA "  
       ATTAACTCAG GAAAAAACGT CATGTAGAGG TTGATCTCAA CCCCTAGGGA ATAGCTCTTC ATCTTAAGAT   
  
129851 "GTTATAACTA TTTTGTTTAA AATTCTTTTT TTTTGTTCAT TCAAAGACTT CCAA"GGATTA TCGAATTCAT   
       CAATATTGAT AAAACAAATT TTAAGAAAAA AAAACAAGTA AGTTTCTGAA GGTTCCTAAT AGCTTAAGTA   
  
129921 CATAAAAAAG TTTTTCTGTT GAAAACAAAG ATATCTTTTT TTCTATCAG"T TCGAAAAAAG TTGCTACACT "  
       GTATTTTTTC AAAAAGACAA CTTTTGTTTC TATAGAAAAA AAGATAGTCA AGCTTTTTTC AACGATGTGA   
  
129991 "AGGTGGATAT GTAAAAACTA TTCGGTCTTT TCCGTCACTT TGACATGTA"T AAAAAAAATA TTGTGACATT   
       TCCACCTATA CATTTTTGAT AAGCCAGAAA AGGCAGTGAA ACTGTACATA TTTTTTTTAT AACACTGTAA   
  
130061 TCTTTTCTAA CAGCATTGTC AAATTTATCA TTCTTTATAT ATCGAAAT"GG ACGATTCCAT CGTTTATAGT "  
       AGAAAAGATT GTCGTAACAG TTTAAATAGT AAGAAATATA TAGCTTTACC TGCTAAGGTA GCAAATATCA   
  
130131 "CGAAAAGGAT AATCCCAAGA GGTTTTTCAA ACCCGAAGAG ATATTTTT"CT TTTTTATCTA TTCGGATCTC   
       GCTTTTCCTA TTAGGGTTCT CCAAAAAGTT TGGGCTTCTC TATAAAAAGA AAAAATAGAT AAGCCTAGAG   
  
130201 TTCCGTTTCA TCGATTTTGT CCGGATCCTC CTTTTCTTCC GAAAAA"AGGG AAGGAGAAGT ATCTTCTTCG "  
       AAGGCAAAGT AGCTAA"AACA GGCCTAGGAG GAAAAGAAGG CTTTTTTCCC TTCCTCTTCA TAGAAGAAGC "  
  
130271 "GTGGATCCCT CTTGTTCCTG TTTAGTCCCC TTCGTTTCGG AAGTGG"TTTC TATTTCTACA TCTGTTTCTT   
       "CACCTAGGGA GAACA"AGGAC AAATCAGGGG AAGCAAAGCC TTCACCAAAG ATAAAGATGT AGACAAAGAA   
  
130341 CCTCGCTTTC CTCCGTTTCC GCCGTTTCTG AGGTTTTTGA GGTTT"CTTTC AGTGGAATGG GTGACGGTAT "  
       GGAGCGAAAG GAGGCAAAGG CG"GCAAAGAC TCCAAAAACT CCAAAGAAAG TCACCTTACC CACTGCCATA "  
  
130411 "TCTGCCTAAA TAGTAGACCC AGGTAATAAA TAAGATAATA CTAAA"GATTG GAGCTATAGA ATTTCTCAAT   
       "AGACGGATTT ATCATCTGGG TC"CATTATTT ATTCTATTAT GATTTCTAAC CTCGATATCT TAAAGAGTTA   
  
130481 TCTGACACAA GGTACTTATT AGATCTAATG TACTTCTTAG ATATAATAGA AT"TATTTTGC TGTATCCAGA "  
       AG"ACTGTGTT CCATGAATAA TCTAGATTAC ATGAAGAATC TATATTATCT TAATAAAACG ACATAGGTCT "  
  
130551 "CTAATATCAA TCCAACCGAT TTCATGAATA AAATGTGACC AATTAACCAA CC"AACAAAAC TACTTGTTAC   
       "GA"TTATAGTT AGGTTGGCTA AAGTACTTAT TTTACACTGG TTAATTGGTT GGTTGTTTTG ATGAACAATG   
  
130621 AAATAACATC TTGTTGTTGC ATCGAAACAT AGAAATGTTG ACTAATCTGA CTAACAT"TGA ACTTGGTAAA "  
       TTTATTGTAG A"ACAACAACG TAGCTTTGTA TCTTTACAAC TGATTAGACT GATTGTAACT TGAACCATTT "  
  
130691 "ATGAAATGGT TGAATAATTG AAAAATGAGA TTATTCAGGA ATACACATTG AATGCTA"AGA TTACGCATTG   
       "TACTTTACCA A"CTTATTAAC TTTTTACTCT AATAAGTCCT TATGTGTAAC TTACGATTCT AATGCGTAAC   
  
130761 AGTTTCTGGT AGTAGATCCA TAATAAAAAA AGTGTTTGGG ATTGGTCCAG AAGAAATG"AA ACAAAAGATA "  
       TCAAAGACCA TCATCTAGGT ATTATTTTTT TCACAAACCC TAACCAGGTC TTCTTTACTT TGTTTTCTAT   
  
130831 "CGGTAGAGCT AGGACAGTTA TTGTATAAGG TCTACCCAAT GCTAGATGCA GAGGCGCA"TA ATAGATCGAT   
       GCCATCTCGA TCCTGTCAAT AACATATTCC AGATGGGTT"A CGATCTACGT CTCCGCGTAT TATCTAGCTA "  
  
130901 ATGAACATCA TGAGCTGTCC CGTAATAAAA CCTGTTGTTG CTGATACTTT CTT"CTCGGTT CCTTCTTCCA "  
       "TACTTGTAGT ACTCGACAGG GCATTATTTT GGACAACAA"C GACTATGAAA GAAGAGCCAA GGAAGAAGGT   
  
130971 "TAAGCCAAGC TCGGAGAAGG AAGAGATAAG AGGGCCCTAT GGAGAATGTG GTC"AGAAATC CATAATAGAG   
       ATTCGGTTCG AGCCTCTTCC TTCT"CTATTC TCCCGGGATA CCTCTTACAC CAGTCTTTAG GTATTATCTC "  
  
131041 TCCGACCACA ACGACCGAAT TGATTATCTT CATGCATAGG GATAC"TAGAT TACCTAGTAT AAAAGATTTT "  
       "AGGCTGGTGT TGCTGGCTTA ACTA"ATAGAA GTACGTATCC CTATGATCTA ATGGATCATA TTTTCTAAAA   
  
131111 "AAAATCATCA CAAACCTCCC TTTTTCTTTT CTATTGCAAT TTTTT"TTCTT TTCTATTGCA ATTTCTAGAT   
       TTTTAGTAGT GTTTGGAGGG AAAAAGAAAA GATAACGTTA AAAAAAAGAA AAGATAACGT TAAAGATCTA   
  
131181 TATTATATTA TTATATGATG ATTTTTTAAC TTTCCATATA TATAGAAATA GAAACAGATA GA"CTAGAGAA "  
       ATAATATAAT AATATACTAC TAAAAAATTG AAAGGTATAT ATATCTTTAT CTTTGTCTAT CTGATCTCT"T "  
  
131251 "ACAGATAGAC TAGAAACGAC ATCTCTTATC TCAATGACCC CACAGGGATA TTAACTGAAT GG"AATTGGGA   
       "TGTCTATCTG ATCTTTGCTG TAGAGAATAG AGTTACTGGG GTGTCCCTAT AATTGACTTA CCTTAACCC"T   
  
131321 TATGGATGGA ATATAATGAA ATAGAGCCAC TTTGAGGTTC TCTAT"GAAAT GAGGCATGGA ACGGAGCCAC "  
       ATACCTACCT TATATTACTT TATCTCGGTG AAACTCCAAG AGATACTTTA CTCCGTACCT TGCCTCGGTG   
  
131391 "TACGAAGAAG TTCCGGGAGT TACGAAGGAA ACCTCGAGCT CATAT"TGGTC ATGGGTTGAG AACGGGAATT   
       ATGCTTCTTC AA"GGCCCTCA ATGCTTCCTT TGGAGCTCGA GTATAACCAG TACCCAACTC TTGCCCTTAA "  
  
131461 GAATTCTCTG AGACCTAATT TACCGTTGTT C"CTCAGTAGC TCAGTGGTAG AGCGGTCGGC CGTTAACCGA "  
       "CTTAAGAGAC TC"TGGATTAA ATGGCAACAA GGAGTCATCG AGTCACCATC TCGCCAGCCG GC"AATTGGCT "  
  
131531 "TTGGTCGCAG GTTCGAATCC TACTTGGGGA G"ATTTGATTC ATTCCGAATT AAAGAATTCG GAATGAAAGG   
       "AACCAGCGTC CAAGCTTAGG ATGAACCCCT CTAAACTAAG TAAGGCTTAA TTTCTTAAGC CT"TACTTTCC   
  
131601 GTTCGCTT"TG ACCGTTAAGA GTAGGTAACC CGTTCCCTGT GTCTTTGTTT CTATTGCATT CTATCTCATC "  
       CAAGCGAAAC TGGCAATTCT CATCCATTGG GCAAGGGACA CAGAAACAAA GATAACGTAA GATAGAGTAG   
  
131671 "GTATCAAA"TT CTGTTCTGCG ATATTTGAGA ATCACCGTCA ATACCTCGGT GTAGGTCCGG GATAATCCTT   
       CATAGTTTAA "GACAAGACGC TATAAACTCT TAGTGGCAGT TATGGAGCCA CATCCAGGCC CTATTAGGAA "  
  
131741 TGTTCCACAG TCTG"GGTCTA TTTACAACTA GCCAATTAAG AATTCTCAGA TGTACTAGTA CTAGCAAGTG "  
       "ACAAGGTGTC" AGACCCAGAT AAATGTTGAT CGGTTAATTC TTAAGAGTCT ACATGATCAT GATCGTTCA"C "  
  
131811 "CATCTTTGAT GCAG"TCATCG ATTCTCCCGA GAGGTCACAA TTACCGCGAG CAAACATATT AATGACGA"GG "  
       "GTAGAAACTA CGTCAGTAGC TAAGAGGGCT CTCCAGTGTT AATGGCGCTC GTTTGTATAA TTACTGCTC"C   
  
131881 "AACACATTTT TGCTATTCTA CTAATACTTG TACTTGCTCT GCTATTCTGC CCGAGCCTGG CTGAGGAA"GA   
       TTGTGTAAAA ACGATAAGAT GATTATGAAC ATGAACGAGA CGATAAGACG GGCTCGGACC GACTCCTTCT   
  
131951 ATTACGGGGC GTAAAACAAA AAAATATGCT GATTCGGGTT GGGTATACTA TATTTAATTT ATAACGGAAC   
       TAATG"CCCCG CATTTTGTTT TTTTATACGA CTAAGCCCAA CCCATATGAT ATAAATTAAA TATTGCCTTG "  
  
132021 CCCCGCCCTT AAC"CCATTAA CGAAAAAGAA AAAATAAAAA GATAAGGCCA TTCCATTTCG ACAAAAGACC "  
       "GGGGC"GGGAA TTGGGTAATT GCTTTTTCTT TTTTATTTTT CTATTCCGGT AAGGTAAAGC TGT"TTTCTGG "  
  
132091 "CACACCCAAG TTC"CATAGCT TTGGGTCCGC TATCCCGATC ATGATTTTCC TACCCCCAGA GGGAAAAGTC   
       "GTGTGGGTTC AAGGTATCGA AACCCAGGCG ATAGGGCTAG TACTAAAAGG ATGGGGGTCT CCC"TTTTCAG   
  
132161 CTTCCC"TTTT TGGCCGGTTG TGGGCGAGGA GGGATTCGAA CCCCCGACAC CGTGGTTCGT AGCCACGTGC "  
       GAAGGGAAAA ACCGGCCAAC "ACCCGCTCCT CCCTAAGCTT GGGGGCTGTG GCACCAAGCA TCGGTGCACG "  
  
132231 "TCTAAT"CCTC TGAGCTACAG GCCCCACCCC GTCTCCACTG GATCTGTTCC GGGGGTACCC TAAAAAAAGG   
       "AGATTAGGAG ACTCGATGTC" CGGGGTGGGG CAGAGGTGAC CTAGACAAG"G CCCCCATGGG ATTTTTTTCC "  
  
132301 AACCTTTCCT CTCC"CCAGCC ATTTGCCATT TCGGGTTAAG AAGATGTGAA AGCGCCTCTC TCTCTATAAG "  
       "TTGGAAAGGA GAGGGGTCGG TAAACGGTAA AGCCCAATTC TTCTACACT"T TCGCGGAGAG AGAGATATTC   
  
132371 "AACGGTGCGT TCCA"AGGTGT GAAGTGAGAT AGAAAAGTGG AGAGAAGGGG TTTTGAATAA GACGACCTTT   
       TTGCCACGCA AGGTTCCACA CTTCACTCTA TCTTTTCA"CC TCTCTTCCCC AAAACTTATT CTGCTGGAAA "  
  
132441 TCATTTTTCA TTTTTTT"TCC ATATTGATAT TGAAAAGTAA TAAGAATTAG AGGTGTTAAG CTTTTTATCA "  
       "AGTAAAAAGT AAAAAAAAGG TATAACTATA ACTTTTC"ATT ATTCTTAATC TCCACAATTC GAAAAATAGT   
  
132511 "TCCTGGCGTC GAGCTAT"TTT TCCGCAGGAC CTCCCCTACA GTATCGTCAC CGCAGTAGAG TTTAACCACC   
       AGGACCGCAG CTCGATAAAA AGGCGTCCTG GAGGGGA"TGT CATAGCAGTG GCGTCATCTC AAATTGGTGG "  
  
132581 AAG"TTCGGGA TGGATTGGTG TGGTTCCTCT ACGCCTAGGA CACCAGAATA TCGAACCATG AACGAAGAAA "  
       "TTCAAGCCCT ACCTAACCAC ACCAAGGAGA TGCGGAT"CCT GTGGTCTTAT AGCTTGGTAC TTGCTTCTTT   
  
132651 "GGC"ATGAGAG AAAAGCATAT TGGCTAGTGA TTGTGGGGCC CCAATTCTTG ACTGGAGGGG ACACCAAAGG   
       CCGTACTCTC TTTTCGTATA ACCGATCAC"T AACACCCCGG GGTTAAGAAC TGACCTCCCC TGTGGTTTCC "  
  
132721 CCTCTG"CCCT TCCATCCCTT GGATAGATAG AGAGGGAGGG CAGAGCTTTT TTTGGTTTTT TCATGTTGTC "  
       "GGAGACGGGA AGGTAGGGAA CCTATCTAT"C TCTCCCTCCC GTCTCGAAAA AAACCAAAAA AGTACAACAG   
  
132791 "AAAGAG"TTGA ACAATGGTTT TTTCGTGTTG TCAAAGAGTT GAACAATGAA AATAGATGGC GAGTGCCTAA   
       TTTCTCAACT TGTTACCAAA AAAGCACAA"C AGTTTCTCAA CTTGTTACTT TTATCTACCG CTCACGGATT "  
  
132861 TCGAATTG"AT CGGGTCATGT AGGAACAAGG TTCAAGTCTA CCGGTCTGTT AGGATGCCTC AGCTGCATAC "  
       "AGCTTAACTA GCCCAGTACA TCCTTGTTC"C AAGTTCAGAT GGCCAGACAA TCCTACGGAG "TCGACGTATG "  
  
132931 "ATCACTGC"AC TTCCACTTGA CACCTATCGT GATGATAAAC GGCTCATCTC GCCGTGACCT TCTCTTGA"AT "  
       "TAGTGACGTG AAGGTGAACT GTGGATAGCA CTACTATTTG CCGAGTAGAG CGGCACTGGA" AGAGA"ACTTA "  
  
133001 "TCTCAAAACT TCTGTCGCTC CATCCCCGCA GGGGCAGAGA ACCCATCGCT GTCTCGGCTG TGCTACCG"GA   
       "AGAGTTTTGA AGACAGCGAG GTAGGGGCGT CCCCGTCTCT TGGGTAGCGA CAGAGCCGAC ACGAT"GGCCT   
  
133071 GGCTCTGGGG AAGTCGGAAT AGGAGAGCAC TCATCTTGGG GTGGGCTTAC TACTTAGATG CTTTCAGCAG   
       CCGAGACCCC TTCAGCCTTA TCCTCTCGTG AGTAGAACCC CACCCGAATG ATGAATCTAC GAAAGTCGTC   
  
133141 TTATCCGCTC CGC"ACTTGGC TACCCAGCGT TTACCGTGGG CACGATAACT GGTACACCAG AGGTGCGTCC "  
       AATAGGCGAG GCGTGAACCG ATGGGTCGCA AATGGCACCC GTGCTATTGA CCATGTGGTC TCCACGCAGG   
  
133211 "TTCCCGGTCC TCT"CGTACTA GGGAAAGGTC CTCTCAATGC TCTAACGCCC ACACCGGATA TGGACCGAAC   
       AAGGGCCAGG AGAGCATGAT CCCTTTCCAG GAGAGTTACG AGATTGCGGG TGTGGCCTAT ACCTGGCTTG   
  
133281 TGTCTCAC"GA CGTTCTGAAC CCAGCTCACG TACCGCTTTA ATGGGCGAAC AGCCCAACCC TTGGAACATA "  
       ACAGAGTGCT GCAAGACTTG GGTCGAGTGC ATGGCGAAAT TACCCGCTTG TCGGGTTGGG AACCTTGTAT   
  
133351 "CTACAGCC"CC AGGTGGCGAA GAGCCGACAT CGAGGTGCCA AACCTTCCCG TCGATGTGAG CTCTTGGGGA   
       GATGTCGGGG TCCACCGCTT CTCGGCTGTA GCTCCACGGT TTGGAAGGGC AGCTACACTC GAGAACCCCT   
  
133421 AGATCAGCCT "GTTATCCCTA GAGTAACTTT TATCCGTTGA GCGACGGCCC TTCCACTCGG CACCGTCGGA "  
       TCTAGTCGGA CAATAGGGAT CTCATTGAAA ATAGGCAACT CGCTGCCGGG AAGGTGAGCC GTGGCAGCCT   
  
133491 "TCACTAAGGC" CGACTTTCGT CCCTGCTCGA CGGGTGGGTC TTGCAGTCAA GCTCCCTTCT GCCTTTGCAC   
       AGTGATTCCG GCTGAAAGCA GGGACGAGCT GCCCACCCAG AACGTCAGTT CGAGGGAAGA CGGAAACGTG   
  
133561 TCGAGGGCCA ATCTCCG"TCT GGCCCGAGGA AACCTTTGCA CGCCTCCGTT ACCTTTTGGG AGGCCTACGC "  
       AGCTCCCGGT TAGAGGCAGA CCGGGCTCCT TTGGAAACGT GCGGAGGCAA TGGAAAACCC TCCGGATGCG   
  
133631 "CCCATAGAAA CTGTCTA"CCT GAGACTGTCC CTTGGCCCGT AGGTCCTGAC ACAAGGTTAG AATTCTAGCT   
       GGGTATCTTT GACAGATGGA CTCTGACAGG GAACCGGGCA TC"CAGGACTG TGTTCCAATC TTAAGATCGA "  
  
133701 CTTCCAGA"GT GGTATCTCAC TGATGGCTCG GGCCCCCCCC GAAGGGGGCC TTCTTCGCCT TCCACCTAAG "  
       "GAAGGTCTCA CCATAGAGTG ACTACCGAGC CCGGGGGGGG CT"TCCCCCGG AAGAAGCGGA AGGTGGATTC   
  
133771 "CTGCGCAG"GA AAGGCCCAAA GCCAATCCCA GGGAACAGTG AAGCTTCATA GGGTCTTTCT GTCCAGGT"GC "  
       GACGCGTCCT TTCCGGGTTT CGGTTAGGGT CCCTTGTCAC TTCGAAGTAT CCCAGAAAGA CAGGTCCACG   
  
133841 "AGGTAGTCCG CATCTTCACA GACATGTCTA TTTCACCGAG CCTCTCTCCG AGACAGTGCC CAGATCGT"TA   
       TCCATCAGGC GTAGAAGTGT CTGTACAGAT AAAGTGGCTC GGAGAGAGGC TCTGTCACGG GTCTAGCAAT   
  
133911 CGCCTTTCGT GCGGGTCGGA ACTTACCCGA CAAGGAATTT CGCTACCTTA GGACCGTTAT AGTTACGGCC   
       GCGGAAAGCA CGCCCAGCCT TGAATGGGCT GTTCCTTAAA GCGATGGAAT CCTGGCAATA TCAATGCCGG   
  
133981 GCCGTTCA"CC GGGGCTTCGG TCGCCGGCTC CCCTGTCATC AGGTCACCAA CTTCCTTGAC CTTCCGGCAC "  
       CGGCAAGTGG CCCCGAAGCC AGCGGCCGAG GGGACAGTAG TCCAGTGGTT GAAGGAACTG GAAGGCCGTG   
  
134051 "TGGGCAGG"CG TCAGCCCCCA TACATGGTCT TACGACTTTG CGGAGACCTG TGTTTTTGGT AAACAGTCGC   
       ACCCGTCCGC AGTCGGGGGT ATGTACCAGA ATGCTGAAAC GCCTCTGGAC ACAAAAACCA TTTGTCAGCG   
  
134121 CCGGGCCTGG TCACTGCG"AC CCCCTTTGTG AGGAGGCACC CCTTCTCCCG AAGTTACGGG GCTATTTTGC "  
       GGCCCGGACC AGTGACGCTG GGGGAAACAC TCCTCCGTGG GGAAGAGGGC TTCAATGCCC CGATAAAACG   
  
134191 "CGAGTTCCTT AGAGAGAG"TT GTCTCGCGCC CCTAGGTATT CTCTACCTAC CCACCTGTGT CGGTTTCGGG   
       GCTCAAGGAA TCTCTCTCAA CAGAGCGCGG GGATCCATAA GAGATGGATG GGTGGACACA GCCAAAGCCC   
  
134261 TACAGGTACC CTTTT"GTTGA AGGTCGTTCG AGCTTTTCCT GGGAGTATGG CATGGGTTAC TTCAGCGCCG "  
       ATGTCCATGG GAAAACAACT TCCAGCAAGC TCGAAAAGGA CCCTCATACC GTACCCAATG AAGTCGCGGC   
  
134331 "TAGCGCCTGG TACTC"GAACA TTGGCTCGAG GTATTTTCTC TACCTCTTCT TACCCTAAAA AAACAGGGGC   
       ATCGCGGACC ATGAGCTTGT AACCGAGCTC CATAAAAGAG ATGGAGAAGA ATGGGATTTT TTTGTCCCCG   
  
134401 "ACCTTGCGTC CTTGAACCGA TAACCATCTT TCGGCTAACC TAGCCTCCTC CGTCCCTCGG GACCAACAAG"   
       TGGAACGCAG GAACTTGGCT ATTG"GTAGAA AGCCGATTGG ATCGGAGGAG GCAGGGAGCC CTGGTTGTTC "  
  
134471 GGGTAGTACA GGAATATTCA CCTGTTGTCC ATCGACTACG CCTTTCGGCC TGATCTTAGG CCCTGACTCA   
       "CCCATCATGT CCTTATAAGT GGAC"AACAGG TAGCTGATGC GGAAAGCCGG ACTAGAATCC GGGACTGAGT   
  
134541 CCCTCCGTGG ACGA"ACCTTG CGGAGGAACC CTTAGGTTTT CGGGGCATTG GATTCTCACC AATGTTTGCG "  
       GGGAGGCACC TGCTTGGAAC GCCTCCTTGG GAATCCAAAA GCCCCGTAAC CTAAGAGTGG TTACAAACGC   
  
134611 "TTACTCAAGC CGAC"ATTCTC GCTTCCGCTT CGTCCACAAC TGCTCGCGCA GGTGCTTCCC TCTAAGGCGG   
       AATGAGTTCG GCTGTAAGAG CGAAGGCGAA GCAGGTGTTG ACGAGCGCGT CCACGAAGGG AGATTCCGCC   
  
134681 AACGCTCCCC TA"CCGATGCA TTTTTACATC CCACAGCTTC GGCAGATCGC TTAGCCCCGT TCATCTTCGG "  
       TTGCGAGGGG ATGGCTACGT AAAAATGTAG GGTGTCGAAG CCGTCTAGCG AATCGGGGCA AGTAGAAGCC   
  
134751 "CGCAAGAGCG CT"CGATCAGT GAGCTATTAC GCACTCTTTC AAGGGTGGCT GCTTCTAGGC AAACCTCCTG   
       GCGTTCTCGC GAGCTAGTCA CTCGATAATG CGTGAGAAAG TTCCCACCGA CGAAGATCCG TTTGGAGGAC   
  
134821 GCTG"TCTCTG CACCCCTACC TCCTTTATCA CTGAGCGGTC ATTTAGGGGC CTTAGCTGGT GATCCGGGCT "  
       CGACAGAGAC GTGGGGATGG AGGAAATAGT GACTCGCCAG TAAATCCCCG GAATCGACCA CTAGGCCCGA   
  
134891 "GTTT"CCCTCT CGACGATGAA GCTTATCCCC CATCGTCTCA CTGGCCGACC TTGACCCCTG TTATTTTGAG   
       CAAAGGGAGA GCTGCTACTT CGAATAGGGG GTAGCAGAGT GACCGGCTGG AACTGGGGAC AATAAAACTC   
  
134961 ATCATATCT"A GTATTCAGAG TTTGCCTCGA TTTGGTACCG CTCTCGCGGC CCGCACCGAA ACAGTGCTTT "  
       TAGTATAGAT CATAAGTCTC AAACGGAGCT AAACCATGGC GAGAGCGCCG GGCGTGGCTT TGTCACGAAA   
  
135031 "ACCCCTAGA"T GTCCAGTCAA CTGCTGCGCC TCAACGCATT TCGGGGAGAA CCAGCTAGCT CTGGGTTCGA   
       TGGGGATCTA CAGGTCAGTT GACGACGCGG AGTTGCGTAA AGCCCCTCTT GGTCGATCGA GACCCAAGCT   
  
135101 GTGGCAT"TTC ACCCCTAACC ACAACTCATC CGCTGATTCT TCAACATCAG TCGGTTCGGA CCTCCACTTA "  
       CACCGTAAAG TGGGGATTG"G TGTTGAGTAG GCGACTAAGA AGTTGTAGTC AGCCAAGCCT GGAGGTGAAT "  
  
135171 "GTTTCAC"CCA AGCTTCATCC TGGTCATGGA TAGATCACCC AGGTTCGGGT CCATAAGCAG TGACAATTGC   
       "CAAAGTGGGT TCGAAGTAG"G ACCAGTACCT ATCTAGTGGG TCCAAGCCCA GGTATTCGTC ACTGTTAACG   
  
135241 CCCATGAAGA CTCGC"TTTCG CTACGGCTCC GGTGGGTTCC CTTAACCAAG CCACTGCCTA TGAGTCGCCG "  
       GGGTACTTCT GAGCGAAAGC GATGCCGAGG CCACCCAAGG GAATTGGTTC GGTGACGGAT ACTCAGCGGC   
  
135311 "GCTCATTCTT CAACA"GGCAC GCGGTCAGAG TCCTCGTCTC CTCCCACTGC TTGGAAGCTT ACGGTTTCAT   
       CGAGTAAGAA GTTGTCCGTG CGCCAGTCTC AGGAGCAGAG GAGGGTGACG AACCTTCGAA TGCCAAAGTA   
  
135381 GTTCTATTTC ACTCCC"CGAT GGGGGTTCTT TTCACCCTTC CCTCACGGTA CTACTTCACT ATCGGTCACC "  
       CAAGATAAAG TGAGGGGCTA CCCCCAAGAA AAGTGGGAAG GGAGTGCCAT GATGAAGTGA TAGCCAGTGG   
  
135451 "CAGGAGTATT TAGCCT"TGCA AGGTGGTCCT TGGCTGATTC ACACGGGATT CCACGTGCCC CATGCTACTC   
       GTCCTCATAA ATCGGAACGT TCCACCAGGA ACCGACTAAG TGTGCCCTAA GGTGCACGGG GTACGATGAG   
  
135521 GGGTCAGGG"G CGTAAGCTAG TGATGCTTTC GGCTACTGGA CTCTCGCCAT CTAGGGTGCA ACACTCCACT "  
       CCCAGTCCCC GCATTCGATC ACTACGAAAG CCGATGACCT GAGAGCGGTA GATCCCACGT TGTGAGGTGA   
  
135591 "GCTTCGCCT"A GCAGCACGAC GCTTGTATTT GCTCTCCCAC AACCCCGTTT TCACGGTTTA GGCTGCTCCC   
       CGAAGCGGAT CGTCGTGCTG CGAACATAAA CGAGAGGGTG TTGGGGCAAA AGTGCCAAAT CCGACGAGGG   
  
135661 ATTTCGCT"CG CCGCTACTAC GGGAATCGCT TTTGCTTTCT TTTCCTCTGG CTACTAAGAT GTTTCAGTTC "  
       TAAAGCGAGC GGCGATGATG CCCTTAGCGA AAACGAAAGA AAAGGAGACC GATGATTCTA CAAAGTCAAG   
  
135731 "GCCAGGTT"GT CTCTTGCCTG CCCATGGATT CAGCAGCAGT TTGAAAGGTT GACCTATTCG GGAATCTCCG   
       CGGTCCAACA GAGAACGGAC GGGTACCTAA GTCGTCGTCA AACTTTCCAA CTGGATAAGC CCTTAGAGGC   
  
135801 GATCTACGCT T"ATTTGCAAC TCCCCGAAGC ATTTCGTCGC TTACTACGCC CTTCCTCGTC TCTGGGTGCC "  
       CTAGATGCGA ATAAACGTTG AGGGGCTTCG TAA"AGCAGCG AATGATGCGG GAAGGAGCAG AGACCCACGG "  
  
135871 "TAGGTATCCA C"CGTAAGCCT TTCTTCGTTT GAACCTCGCC CTTAACTTTA AGGCTATGCC ATCCTAAGGT   
       "ATCCATAGGT GGCATTCGGA AAGAAGCAAA CTT"GGAGCGG GAATTGAAAT TCCGATACGG TAGGATTCCA   
  
135941 GCTGCTAA"AT GGAAGGATCT TATCAACGTC CATGAATGAT AAATCATAGA TCGAACTGCC GAATCGGAAA "  
       CGACGATTTA CCTTCCTAGA ATAGTTGCAG GTACTTACTA TTTA"GTATCT AGCTTGACGG CTTAGCCTTT "  
  
136011 "AATGGAGT"GC TATCATATAG CTTTGTATCG GCTAAGTTCA CGAGTTGGAG ATAAGCGGAC TCGAACCGCT   
       "TTACCTCACG ATAGTATATC GAAACATAGC CGATTCAAGT GCTC"A"ACCTC TATTCGCCTG AGCTTGGCGA "  
  
136081 GACATCCG"CC ACAGGGTAAA CCACCGCCTC TCAGGCCCCC GACTGATTCT ACCATAGAGG CCAACGATAG "  
       "CTGTAGGCGG" TG"TCCCATTT GGTGGCGGAG AGTCCGGGGG CTGACTAAGA TGGTATCTCC GGTTGCTATC "  
  
136151 "ACAATAAC"TC CCCCCCGAAC ACAGCTTACA ACTTTCATCG TACTGTGCTC TCCAAAGAGC AACTCTTCTC   
       "TGTTATTGAG GG"GGGGCTTG TGTCGAATGT TGAAAGTAGC ATGACACGAG AGGTTTCTCG TTGAGAAGAG   
  
136221 AAAATCTCAA AA"GGTACTGA GTTGGAATCC CATTCTAACT AAGGATTCTT GTGGTTCCGG AGAATCCAGC "  
       TTTTAGAGTT TTCCATGACT "CAACCTTAGG GTAAGATTGA TTCCTAAGAA CACCAAGGCC TCTTAGGTCG "  
  
136291 "TACAGGAGAA CC"AGGAACGG AGAGCTTTCC CCCCTTTTCC GCCCGCCTCT TTGGTCTTAA GAATGCTGGT   
       "ATGTCCTCTT GGTCCTTGCC" TCTCGAAAGG GGGGAAAAGG CGGGCGGAGA AACCAGAATT CTTACGACCA   
  
136361 TTTA"AGAATG AGTGATTGCC CTTCTCCGAC CCTTACTGCC CAACCGGAGA GCGGACAGCT AATGCGTTCC "  
       AAATTCTTAC TCACTAACGG GA"AGAGGCTG GGAATGACGG GTTGGCCTCT CGCCTGTCGA TTACGCAAGG "  
  
136431 "ACTT"ATTGAA CAGGGTTCTA TGGTCGGTCC GCGACCCCTG GATACCGAAG GCGTCCTTGG GGTGATCTCG   
       "TGAATAACTT GTCCCAAGAT AC"CAGCCAGG CGCTGGGGAC CTATGGCTTC CGCAGGAACC CCACTAGAGC   
  
136501 TAGTTCCT"AC GGGGTGGAGA CGATGGGGTC GGTCCATGGA TTTTCCTTCC TTTTGCCGCA TTTCGCTCAA "  
       ATCAAGGATG CC"CCACCTCT GCTACCCCAG CCAGGTACCT AAAAGGAAGG AAAACGGCGT AAAGCGAGTT "  
  
136571 "AGGGTTGA"AG GGAGATAGTG CATCAAGCTG TTCGCAAGGG CCAACTTGAT CCTCTTCCCC AGGGGATCCC   
       "TCCCAACTTC CC"TCTATCAC GTAGTTCGAC AAGCGTTCCC GGTTGAACTA GGAGAAGGGG TCCCCTAGGG   
  
136641 AGATGAGG"GA ACCCTAAGAG AGCCGCCGAC TCCAACTACC GTCCATGTAC GATCCATACT AGATCTGACC "  
       TCTACTCCCT TGGGATT"CTC TCGGCGGCTG AGGTTGATGG CAGGTACATG CTAGGTATGA TCTAGACTGG "  
  
136711 "AACTGCCC"AT CCTACCTCCT CTACGTTCTT GACAGCCCAT CTTTGTCTCA GTAGAGTCTT TCAGTGGCAT   
       "TTGACGGGTA GGATGGA"GGA GATGCAAGAA CTGTCGGGTA GAAACAGAGT CATCTCAGAA AGTCACCGTA   
  
136781 GTTTCGGT"CC TCTTCCCCAT TACTTAGAAA AAGTGAGCCA CCGGTTCAGG TACAAGATAC TATCATTACC "  
       CAAAGCCAGG A"GAAGGGGTA ATGAATCTTT TTCACTCGGT GGCCAAGTCC ATGTTCTATG ATAGTAATGG "  
  
136851 "GCCTGGAC"AA TTAGACATCC AACCCGTAAT CGCAACGACC CAATTGCAAG AGCGGAGCTC TACCAACTGA   
       "CGGACCTGTT A"ATCTGTAGG TTGGGCATTA GCGTTGCTGG GTT"AACGTTC TCGCCTCGAG ATGGTTGACT "  
  
136921 GCTATATCC"C CCCGAGCCAA GTGGGGCCTG CATGAAGGAG TCAGATGCTT CTTCTATTCT TTTATTCTTT "  
       "CGATATAGGG G""GGCTCGGTT CACCCCGGAC GTACTTCCTC AGTCTACGAA GAAGATAAGA AAATAAGAAA "  
  
136991 "TCCTTGGCG"T AGCTGGGCCA TCCTGGACTT GAACCAGAGA CCTCGCCCGT GAAGTAAATC ATCGCACCTA   
       "AGGAACCGCA" TCG"ACCCGGT AGGACCTGAA CTTGGTCTCT GGAGCGGG"CA CTTCATTTAG TAGCGTGGAT   
  
137061 CGGTCCAACC AATT"GGGAGA GAATCAATAG ATTCCTTTTC GGGAGCGATT CATCCTTCCC GAACGCAGCA "  
       GCCAGGTTGG TTAAC"CCTCT CTTAGTTATC TAAGGAAAAG CCCTCGCTAA GTAGGAAGGG CTTGCGTCGT "  
  
137131 "TACAACTCTC CGTT"GTACTG CGCTCTCCAA GTGTGCTTGT TCCCCCCTTC TTCCTTACCA TGGCAAGTCT   
       "ATGTTGAGAG GCAAC"ATGAC GCGAGAGGTT CACACGAACA AGGGGGGAAG AAGGAATGGT ACCGTTCAGA   
  
137201 TTGTGAAA"TA ACTCCGATGA GAAGAAAAAA GAAGGCGTTA AGAAACCCTC CTGGCCCAAC CCTAGACACT "  
       AACACTTTAT TGAGGCTACT CTTCTT"TTTT CTTCCGCAAT TCTTTGGGAG GACCGGGTTG GGATCTGTGA "  
  
137271 "CTAAGATC"CT TTTTCAAATC TCCTGGTCCC TGCGGAAGAA AGGAAAAAGA ATTTCACGTT CTTCCTTTCG   
       "GATTCTAGGA AAAAGTTTAG AGGACC"AGGG ACGCCTTCTT TCCTTTTTCT TAAAGTGCAA GAAGGAAAGC   
  
137341 CTTTCGGGAA GGGA"GGATTA AGAAAATCCT ATTGATTGCA GCTTTCTCCA GACCTCCGGG AAAAGCATGA "  
       GAAAGCCCTT CCCTCCTAAT TCTTTTAG"GA TAACTAACGT CGAAAGAGGT CTGGAGGCCC TTTTCGTACT "  
  
137411 "AAAAAAAAGG CTCG"AATGGT ACGATCCCTC CGTCACCCCA GAATGAAAGG GGCGATCTCG TAGTTCTTGG   
       "TTTTTTTTCC GAGCTTACCA TGCTAGGG"AG GCAGTGGGGT CTTACTTTCC CCGCTAGAGC ATCAAGAACC   
  
137481 TCTGTGGAGA "TACGTTGTTA GGTGCTCCGT TTTATTTTCC CATTGAGGCC GAACCTAAAC CTGTGCTCGA "  
       AGACACCTCT "ATGCAACAAT CCACGAGGCA AAATAAAAGG GTAACTCCGG CTTGGATTTG GACACGAGCT "  
  
137551 "GAGATAGCTG" TCCATATACT GATAAGGGAT GTATGGATTC TCGAGAAGAG AGGAGCCGAG GTGGTCCCCC   
       "CTCTATCGAC" AGGTATATGA CTATTCCCTA CATACCTAAG AGCTCTTCTC TCCTCGGCTC CACCAGGGGG   
  
137621 CCGGACCGCC C"GGATCCCAC GAGTGAATAG AAAGTTGGAT CTACATTGGA TCTCACCTGA ATCGCCCCAT "  
       GGCCTGGCGG G"CCTAGGGTG CTCACTTATC TTTCAACCTA GATGTAACCT AGAGTGGACT TAGCGGGGTA "  
  
137691 "CTATCCTCCT G"AGGAGAAGT TTGGTTTCAA ACCCCGGTTC AAACAGGAGA AGTACGCCAT GCTAATGTGC   
       "GATAGGAGGA C"TCCTCTTCA AACCAAAGTT TGGGGCCAAG TTTGTCCTCT TCATGCGGTA CGATTACACG   
  
137761 CTTGGAT"GAT CCACATCTCA GGGTCAGGCG CTGATGAGCA CATTGAACTA TCCATGTGGC TGAGAGCCCT "  
       GAACCTAC"TA GGTGTAGAGT CCCAGTCCGC GACTACTCGT GTAACTTGAT AGGTACACCG ACTCTCGGGA "  
  
137831 "CACAGCC"CAG GCACAACGAC GCAATTATCA GGGGCGCGCT CTACCACTGA GCTAATAGCC CGTCGTGCGG   
       "GTGTCGGG"TC CGTGTTGCTG CGTT"AATAGT CCCCGCGCGA GATGGTGACT CGATTATCGG G"CAGCACGCC   
  
137901 GCCTCCCGCT GGGGGCC"CGC TATGCCAAAA GCGAGAGAAA CCCCATCCCT CTCTTTCCTT TTTTCGCCCC "  
       CGGAGGGCGA CCCCCGGGCG ATACGGTTTT CGCTCTCTTT GGGGTAGGGA GAGAAAGGAA AAAAGCGGG"G "  
  
137971 "CATGTCGCCA CACGGGA"GGG ACACGGGGAC GTAAAAAAGG GGATCCTATC AACTTGTTCC GACCTAGGAT   
       "GTACAGCGGT GTGCCCTCCC TGTGCCCCTG CATTTTTTCC CCTAGGATAG TTGAACAAGG CTGGATCCT"A   
  
138041 AATAAGCTCA TGAGCT"TAGT CTTACTTCAC CGTCGAGAAA CGAAAGAAGA CTTCCATCTC CAAGTTTAAC "  
       TTATTCGAGT ACTCGAATCA GAATGAAGTG GCAGCTCTTT GCTTTCTTCT GAAGGTAGAG GTTCAAATTG   
  
138111 "TCAGACGTAG CTCGCT"TCTT TTTGGGTGTG AAGCAGTGTC AAACCAAAAT ACCCAACAAG CATTAGCTCT   
       A"GTCTGCATC GAGCGAAGAA AAACCCACAC TTCGTCACAG TTTGGTTTTA TGGGTTGTTC GTAATCGAGA "  
  
138181 CCCTGAA"AAG GAGGTGATCC AGCCGCACCT TCCAGTACGG CTACCTTGTT ACGACTTCAC TCCAGTCACT "  
       "G"GGACTTTTC CTCCACTAGG TCGGCGTGGA AGGTCATGCC GATGGAACAA TGCTGAAGTG AGGTCAGTGA   
  
138251 "AGCCCTG"CCT TCGGCATCCC CCTCCTTGCG GTTAAGGTAA CGACTTCGGG CATGGCCAGC TCCCATAGTG   
       TCGGGACGGA AGCCGTAGGG GGAGGAACGC CAATTCCATT GCTGAAGCCC GTACCGGTCG AGGGTATCAC   
  
138321 TGACGGGC"GG TGTGTACAAG GCCCGGGAAC GAATTCACCG CCGTATGGCT GACCGGCGAT TACTAGCGAT "  
       ACTGCCCGCC ACACATGTTC CGGGCCCTTG CTTAAGTGGC GGCATACCGA CTGGCCGCTA ATGATCGCTA   
  
138391 "TCCGGCTT"CA TGCAGGCGAG TTGCAGCCTA CAATCCGAAC TGAGGACGGG TTTTTGGAGT TAGCTCACCC   
       AGGCCGAAGT ACGTCCGCTC AACGTCGGAT GTTAGGCTTG ACTCCTGCCC AAAAACCTCA ATCGAGTGGG   
  
138461 TCGCGGGATC ACGACCC"TTT GTCCCGGCCA TTGTAGCACG TGTGTCGCCC AGGGCATAAG GGGCATGATG "  
       AGCGCCCTAG TGCTGGG"AAA CAGGGCCGGT AACATCGTGC ACACAGCGGG TCCCGTATTC CCCGTACTAC "  
  
138531 "ACTTGACGTC ATCCTCA"CCT TCCTCCGGCT TATCACCGGC AGTCTGCTCA GGGTTCCAAC CTCAACGG"TT "  
       "TGAACTGCAG TAGGAGT"GGA AGGAGGCCGA ATAGTGGCCG TCAGACGAGT CCCAAGGTTG GAGTTGCCAA   
  
138601 "GGCAACTAAA CACGAGGGTT GCGCTCGTTG CGGGACTTAA CCCAACACCT TACGGCACGA GCTGACGA"CA   
       CCGTTGATTT GTGCTCCCAA CGCGAGCAAC GCCCTGAATT GGGTTGTGGA ATGCCGTGCT CGACTGCTGT   
  
138671 GCCATGCACC ACCTGTGTCC GCGTTCCCGA AGGCACCCCT CTCTTTCAAG AGGATTCGCG GCATGTCAAG   
       CGGTACGTGG TGGACACAGG CGCAAGGGCT TCCGTGGGGA GAGAAAGTTC TCCTAAGCGC CGTACAGTTC   
  
138741 CCCT"GGTAAG GTTCTTCGCT TTGCATCGAA TTAAACCACA TGCTCCACCT CTTGTGCGGG CCCCCGTCAA "  
       GGGACCATTC CAAGAAGCGA AACGTAGCTT AATTTGGTGT ACGAGGTGGA GAACACGCCC GGGGGCAGTT   
  
138811 "TTCC"TTTGAG TTTCATTCTT GCGAACGTAC TCCCCAGGCG GGATACTTAA CGCGTTAGCT ACAGCACTGC   
       AAGGAAACTC AAAGTAAGAA CGCTTGCATG AGGGGTCCGC CCTATGAATT GC"GCAATCGA TGTCGTGACG "  
  
138881 ACGGGTCG"AT ACGCACAGCG CCTAGTATCC ATCGTTTACG GCTAGGACTA CTGGGGTATC TAATCCCATT "  
       "TGCCCAGCTA TGCGTGTCGC GGATCATAGG TAGCAAATGC CGATCCTGAT GA"CCCCATAG ATTAGGGTAA   
  
138951 "CGCTCCCC"TA GCTTTCGTCT CTCAGTGTCA GTGTCGGCCC AGCAGAGTGC TTTCGCCGTT GGTGTTCTTT   
       GCGAGGGGAT CGAAAGCAGA GAGTCACAGT CACAGCCGGG TCGTCTCACG AAAGCGGCAA CCACAAGAAA   
  
139021 CC"GATCTCTA CGCATTTCAC CGCTCCACCG GAAATTCCCT CTGCCCCTAC CGTACTCCAG CTTGGTAGTT "  
       GGCTAGAGAT GCGTAAAGTG GCGAGGTGGC CTTTAAGGGA GACGGGGATG GCATGAGGTC GAACCATCAA   
  
139091 "TC"CACCGCCT GTCCAGGGTT GAGCCCTGGG ATTTGACGGC GGACTTAAAA AGCCACCTAC AGACGCTTTA   
       AGGTGGCGGA CAGGTCCCAA CTCGGGACCC TAAACTGCCG CCTGAATTTT TCGGTGGATG TCTGCGAAAT   
  
139161 CGCCC"AATCA TTCCGGATAA CGCTTGCATC CTCTGTATTA CCGCGGCTGC TGGCACAGAG TTAGCCGATG "  
       GCGGGTTAGT AAGGCCTATT GCGAACGTAG GAGACATAAT GGCGCCGACG ACCGTGTCTC AAT"CGGCTAC "  
  
139231 "CTTAT"TCCCC AGATACCGTC ATTGCTTCTT CTCCGGGAAA AGAAGTTCAC GACCCGTAGG CCTTCTACCT   
       "GAATAAGGGG TCTATGGCAG TAACGAAGAA GAGGCCCTTT TCTTCAAGTG CTGGGCATCC GGA"AGATGGA   
  
139301 CCACGC"GGCA TTGCTCCGTC AGGCTTTCGC CCATTGCGGA AAATTCCCCA CTGCTGCCTC CCGTAGGAGT "  
       GGTGCGCCGT AACGAGGCAG TCCGAAAGCG GGTAACGCCT TTTAAGGGGT GACGACGGAG GGCATCCTCA   
  
139371 "CTGGGC"CGTG TCTCAGTCCC AGTGTGGCTG ATCATCCTCT CGGACCAGCT ACTGATCATC GCCTTGGT"AA "  
       GACCCGGCAC AGAGTCAGGG TCACACCGAC TAGTAGGAGA GCCTGGTCGA TGACTAGTAG CGGAACCATT   
  
139441 "GCTATTGCCT CACCAACTAG CTAATCAGAC GCGAGCCCCT CCTCGGGCGG ATTCCTCCTT TTGCTCCT"CA   
       CGATAACGGA GTGGTTGATC GATTAGTCTG CGCTCGGGGA GGAGCCCGCC TAAGGAGGAA AACGAGGAGT   
  
139511 GCGTACGGGG TATTAGCAGC CGTTTCCAGC TGTTGTTCCC CTCCCAAGGG CAGGTTCTTA CGCGTTACTC   
       CGCATGCCCC ATAATCGTCG GCAAAGGTCG ACAACAAGGG GAGGGTTCCC GTCCAAGAAT GCGCAATGAG   
  
139581 ACCCGTCCGC C"ACTGGAAAC ACCACTTCCC GTCCGACTTG CATGTGTTAA GCATGCCGCC AGCGTTCATC "  
       TGGGCAGGCG GTGACCTTTG TGGTGAAG"GG CAGGCTGAAC GTACACAATT CGTACGGCGG TCGCAAGTAG "  
  
139651 "CTGAGCCAGG A"TCGAACTCT CCATGAGATT CATAGTTGCA TTACTTATAG CTTCCTTGTT CGTAGACAAA   
       "GACTCGGTCC TAGCTTGAGA GGTACTCT"AA GTATCAACGT AATGAATATC GAA"GGAACAA GCATCTGTTT "  
  
139721 GCTAATTCGG AATTGT"CTTT CATTCCAAGG CATAACTTGT ATCCATGCGC TTCATATTCG CCTGGAGTTC "  
       "CGATTAAGCC TTAACAGAAA GTAAGGTTCC GTATTGAACA TAGGTACGCG AAG"TATAAGC GGACCTCAAG   
  
139791 "GCTCCCAGAA ATATAG"CCAT CCCCACCCCC TCACGTCAAT CCCACGAGCC TCTTATCCAT TCTCATTCGA   
       CGAGGGTCTT TATATCGGTA GGGGTGGGGG AGTGCAGTTA GGGTG"CTCGG AGAATAGGTA AGAGTAAGCT "  
  
139861 TCACGGC"GGG GGAGCAAGTC AAAATAGAAA AACTCACATT GGGTTTAGGG ATAATCAGGC TCGAACTGAT "  
       "AGTGCCGCCC CCTCGTTCAG TTTTATCTTT TTGAGTGTAA CCCAA"AT"CCC TATTAGTCCG AGCTTGACTA "  
  
139931 "GACTTCC"GCC ACGTCAAGGC GACACTCTAC CGCTGAGTTA TATCCCTTCC CTTGCCCCCA TCGAGAAATA   
       "CTGAAGGCGG TGCAGTTCCG CTGTGAGATG GCGACTCAAT ATAGGGA"AGG GAACGGGGGT AGCTCTTTAT   
  
140001 GAACTGA"CTA ATCCTAAGGC AAAGGGTCGA GAAACTCAAC GCCACTATTC TTGAACAACT TGGAATTGGG "  
       CTTGACTGAT TAGGATTCCG TTTCCCAGCT CTTTGAGT"TG CGGTGATAAG AACTTGTTGA ACCTTAACCC "  
  
140071 "CCTTCCT"TCT TTTCGTACGG ATACGAAAAT GAAAATAATG GGCAAAATTG TATTCAATTG TCAACAGCT"C "  
       "GGAAGGAAGA AAAGCATGCC TATGCTTTTA CTTTTATT"AC CCGTTTTAAC ATAAGTTAAC AGTTGTCGAG   
  
140141 "CTATCGTAAA TAGGATTGAC TACGGATTCG AGCCATAGCA CACGGTTTCA TAAAACCGTA CGATTTTCC"C   
       GATAGCATTT ATCCTAACTG ATGCCTAA"GC TCGGTATCGT GTGCCAAAGT ATTTTGGCAT GCTAAAAGGG "  
  
140211 GATCTAAATA AAGCAGGTTT TACATGAAGA AGATTTGGCT CAGCATGTTC TATTCGATAC GGGTAGGAAA   
       "CTAGATTTAT TTCGTCCAAA ATGTACTT"CT TCTAAACCGA GTCGTACAAG ATAAGCTATG CCCATCCTTT   
  
140281 AGAACCCAAC TCG"GTATTAT TAAAAAAATA GAGAAATCAG AACCCAGTCA AGATGATATG GATCAACCCC "  
       TCTTGGGTTG AGCCATAATA ATTTTTTTAT CTCT"TTAGTC TTGGGTCAGT TCTACTATAC CTAGTTGGGG "  
  
140351 "TTCTTCTTGT GCC"AAAGATC TTACCATTTC CGAAGGAAGC TGGAGTTACA TCTCTTTTCC ATTTCCATTC   
       "AAGAAGAACA CGGTTTCTAG AATGGTAAAG GCTT"CCTTCG ACCTCAATGT AGAGAAAAGG TAAAGGTAAG   
  
140421 CAGAGTTTTT AT"GTGTTTCC ACGCCCCTTC GAGACCCCGA AAAATGAACA ACTTTTCTTC GGAACACATA "  
       GTCTCAAAAA TACACAAAGG TGCGGGGAAG CTCTGGGGCT TTTTACTT"GT TGAAAAGAAG CCTTGTGTAT "  
  
140491 "CAAGATTCGT CA"TTGCAAAA AGGATAATGG TAACCCCACC ATTAACTACT TCATTTATGA ATTTCATAGT   
       "GTTCTAAGCA GTAACGTTTT TCCTATTACC ATTGGGGTGG TAATTGAT"GA AGTAAATACT TAAAGTATCA   
  
140561 AATAGAAAT"A CATGTCCTAC CGCGACAGAA TTTGTAACTT GCTATCCTCT TGCCTAGCAG GCAAAGATTT "  
       TTATCTTTAT GTACAGGATG GCGCTGTCTT AA"ACATTGAA CGATAGGAGA ACGGATCGTC CGTTTCTAAA "  
  
140631 "ACCTCCGTG"G AAAGGATGAT TCATTCGGAT CGACATGAGA GTCCAACTAC ATTGCATTGC CAGAATCCAT   
       "TGGAGGCACC TTTCCTACTA AGTAAGCCTA GC"TGTACTCT CAGGTTGATG TAACGTAACG GTCTTAGGTA   
  
140701 "GTTGTATATT TGAAAGAGGT TGACCTCCTT GCTTCTCTCA TGTTACAATC CTCTTCCCGC CGAGCCCCCT"   
       CAACATATAA ACTTTCTCCA ACTGGAGGAA CGAAGAGAGT ACAATGTT"AG GAGAAGGGCG GCTCGGGGGA "  
  
140771 TTCTCCTCGA TCCACAGAGA AAAAATGGAG GACTGGTGCC AACAGTTCAT CACGGAAGAA AGGACTCACT   
       "AAGAGGAGCT AGGTGTCTCT TTTTTACCTC CTGACCACGG TTGTCAAG"TA GTGCCTTCTT TCCTGAGTGA   
  
140841 GAGCCGAGAT CACTAAC"TAA TACTAATCTA ATACTAATAG AATAGAAAAG AACTGTCTTT TCTGTATACT "  
       CTCGGCTCTA GTGATTGATT ATGATTAGAT TATGATTATC TTAT"CTTTTC TTGACAGAAA AGACATATGA "  
  
140911 "TTCCCCGGTT CCGTTGC"TAC CGCGGGCTTT ACGCAATCAA TCGGATCATA TAGATATCCC TTCAACACAA   
       "AAGGGGCCAA GGCAACGATG GCGCCCGAAA TGCGTTAGTT AGCC"TAGTAT ATCTATAGGG AAGTTGTGTT   
  
140981 CATAGGTCAT "CGAAAGGATC TCGGAGACCC ACCAAAGCAC GAAAGCCAGA ATCTTTCAGA AAATGGATTC "  
       GTATCCAGTA GCTTTCCTAG AGCCTCTGGG TGGTTTCGTG CTTT"CGGTCT TAGAAAGTCT TTTACCTAAG "  
  
141051 "CTATTCGAAG" AGTGCATAAC CGCATGGATA AGCTCACACT AACCCGTCAA TTTTGGATCC AATTCGGAAT   
       "GATAAGCTTC TCACGTATTG GCGTACCTAT TCGAGTGTGA TTGG"GCAGTT AAAACCTAGG TTAAGCCTTA   
  
141121 TTTAGGT"ATC GGGAAGGAAT TGGAATGTAA TAATATCGAT TCATACAGAT ACAGAAGAAA AGGTTCTCTA "  
       AAATCCATAG CCCTTCCTTA ACCTTACATT ATTATAGCT"A AGTATGTCTA TGTCTTCTTT TCCAAGAGAT "  
  
141191 "TTGATTC"AAA CACTGTACCC GCGGGATAGG GATAGAGAAA GAGGAAAAAA ACGAAGATTT CACATAGTAC   
       "AACTAAGTTT GTGACATGGG CGCCCTATCC CTATCTCTT"T CTCCTTTTTT TGCTTCTAAA GTGTATCATG   
  
141261 TTTTGATCGA AAAATCAA"TC TGATTTATTT CGTACCTTTC GCTCAATGAA AAAATGGGTC AGATTCTACA "  
       AAAACTAGCT TTTTAGTTAG ACTAAATAAA GCATGGAA"AG CGAGTTACTT TTTTACCCAG TCTAAGATGT "  
  
141331 "GGATCAAACC TATGGGAC"TT AAGGAATGAT CGAAGGGAAT AAAAAAAGAA AAAAAAAAAA AGAGAGGGAA   
       "CCTAGTTTGG ATACCCTGAA TTCCTTACTA GCTTCCCT"TA TTTTTTTCTT TTTTTTTTTT TCTCTCCCTT   
  
141401 AAATAAGTAA ATA"AAAATGA CGTAGAAGAG CCCAGATTCC AAATGAATGA AAACGTGACT GAATTGGTCC "  
       TTTATTCATT TATTTTTACT GCATCTTCTC GGGTCTAAGG TTTACT"TACT TTTGCACTGA CTTAACCAGG "  
  
141471 "CGGTCACTCT TCG"GGACGGA ATGGAAGAAG GGAGGAGATT CTCGAACGAG GAAAAGGATC CAATGACTTC   
       "GCCAGTGAGA AGCCCTGCCT TACCTTCTTC CCTCCTCTAA GAGCTT"GCTC CTTTTCCTAG GTTACTGAAG   
  
141541 GAA"AGAATTG AACGAGGAGC CGTATGAGGT GAAAATCTCA CGTACGGTTC TGTCGAGTGG CAGTAAGGGT "  
       CTTTCTTAAC TTGCTCCTCG GCATACTCCA CTTTTAGAGT GCATGCC"AAG ACAGCTCACC GTCATTCCCA "  
  
141611 "GAC"TTATCTG TCAACTTTTC CACTATCACC CCCAAAAAAC "CAAACTCTGC CTTACGTAAA GTTGCCAGAG "  
       "CTGAATAGAC AGTTGAAAAG GTGATAGTGG GGGTTTTTTG GTTTGAG"ACG GAATGCATTT CAACGGTCTC   
  
141681 "TACGCTTAAC CTCGGGATTT GAAATCACTG CTTATATACC" TGGTATTGGC CATAATTTAC AAGAACATTC   
       ATGCGAATTG GAGCCCTAAA CTTTAGTGAC GAATATATG"G ACCATAACCG GTATTAAATG TTCTTGTAAG "  
  
141751 TGTAGTCTTA GT"AAGAGGGG GAAGGGTTAA GGATTTACCC GGTGTGAGAT ATCACATTGT TCGAGGAACC "  
       "ACATCAGAAT CATTCTCCCC CTTCCCAATT CCTAAATGG"G CCACACTCTA TAGTGTAACA AGCTCCTTGG   
  
141821 "CTAGATGCTG TC"GGAGTAAA GGATCGTCAA CAAGGGCGTT CTAGTGCGTT GTAGATTCTT ATCCA"AGACT "  
       GATCTACGAC AGCCTCATTT CCTAGCAGTT GT"TCCCGCAA GATCACGCAA CATCTAAGAA TAGGTTCTGA "  
  
141891 "TGTATCATTT GATGATGCCA TGTGAATCGC TAGAAACATG TGAAGTGTAT GGCTAACCCA ATAAC"GAAAG   
       "ACATAGTAAA CTACTACGGT ACACTTAGCG AT"CTTTGTAC ACTTCACATA CCGATTGGGT TATTGCTTTC   
  
141961 TTTCGTAAGG GAACTGGAGC AGGCTACCAT GAGACAAACA AAAGATCTTC TTTCTAAAGA GATT"CGATTC "  
       AAAGCATTCC CTTGACCTCG TCCGATGGTA CTC"TGTTTGT TTTCTAGAAG AAAGATTTCT CTAAGCTAAG "  
  
142031 "GGAACTATTA TATGTCCAAG GTCCAATATT GAAATAATTT CAGAGGTTTT CCTTGACTTT GTCC"GTGTCA   
       "CCTTGATAAT ATACAGGTTC CAGGTTATAA CTT"TATTAAA GTCTCCAAAA GGAACTGAAA CAGGCACAGT   
  
142101 ACAAACAATT CGAAATGCCT CGACTTTTTT AGAACAGGTC CGAGTCAAAT A"GTAATGATT CGAAGCACCT "  
       TGTTTGTTAA GCTTTACGGA GCTGAAAAAA TCT"TGTCCAG GCTCAGTTTA TCATTACTAA GCTTCGTGGA "  
  
142171 "CTTTTTACAC TATTTCGGAA ACCCAAGGAC TCAATCGTAT GGATATGTAA A"ATACAGGAT TTCCAATCCT   
       "GAAAAATGTG ATAAAGCCTT TGGGTTCCTG AGT"TAGCATA CCTATACATT TTATGTCCTA AAGGTTAGGA   
  
142241 AGCAGGAAAG GGAGGGAAAC GGATACTCAA TTTAAAGTGA GTAAACAGAA T"TCCATACTC GATCTCATAG "  
       TCGTCCTTTC CCTCCCTTTG CCTATGAGTT AAATTTCACT CATTT"GTCTT AAGGTATGAG CTAGAGTATC "  
  
142311 "ATACATATAG AATTCTGTGG AAAGCCGTAT TCGATGAAAG TCGTATGTAC G"GCTTGGAGG GAGATCTTTC   
       "TATGTATATC TTAAGACACC TTTCGGCATA AGCTACTTTC AGCAT"ACATG CCGAACCTCC CTCTAGAAAG   
  
142381 ATATCTTTCG AGATCCACCC TAC"AATATGG GGTCAAAAAG CCAAAATAA"A TGATTTTAGC CCTTATAAAA   
       TATAGAAAGC TCTAGGTGGG ATGTTATACC CCAGTTTTTC GG"TTTTATTT ACTAAAATCG GGAATATTTT "  
  
142451 AGAAAACTTA TTCTTGAACC CCTTTCACGC TCATGTCACG TCGAGGTACT GCAGAAGAAA AAA"TTGAAAA "  
       "TCTTTTGAAT AAGAACTTGG GGAAAGTGCG AGTACAGTGC AG"CTCCATGA CGTCTTCTTT TTTAACTTTT   
  
142521 "ATCCGATCCA ATTTATCGTA ATCGATTAGT TAACATGTTG GTTAACCGTA TTCTGAAACA TGG"AAAAAAA   
       TAGGCTAGGT TAAATAGCAT TAGCTAATCA "ATTGTACAAC CAATTGGCAT AAGACTTTGT ACCTTTTTTT "  
  
142591 TCATTGGCTT ATCAAATTAT CTATCGAGCC ATGAAAAAGA TTCAACAA"AA GACAGAAACA AATCCACTAT "  
       "AGTAACCGAA TAGTTTAATA GATAGCTCGG" TACTTTTTCT AAGTTGTTTT CTGTCTTTGT TTAGGTGATA   
  
142661 "CTGTTTTACG TCAAGCAATA CGTGGAGTAA CTCCCGATAT AGCAGTAA"AG GCAAGACGTG TAGGCGGATC   
       GACAAAATGC AGTTCGTTAT GCACCTCAT"T GAGGGCTATA TCGTCATTTC CGTTCTGCAC ATCCGCCTAG "  
  
142731 AACTCATCAA GTTCCCATTG AAATA"GGATC CACACAAGGA AAAGCACTTG CCATTCGTTG GTTATTAGGG "  
       "TTGAGTAGTT CAAGGGTAAC TTTATCCTA"G GTGTGTTCCT TTTCGTGAAC GGTAAGCAAC CAATAATCCC   
  
142801 "GCATCCCGAA AACGTCCCGG TCGAA"ATATG GCTTTCAAA"T TCAGTTCCGA ATTAGTGGAT GCTGCCAAAG "  
       CGTAGGGCTT TTGCAGGGCC AGCTTTATAC CGAAAGTTT"A AGTCAAGGCT TAATCACCTA CGACGGTTTC "  
  
142871 "GGAGTGGCGA TGCCATACGT AAAAAGGAAG AGACTCATA"G AATGGCAGAG GCAAATAGAG CTTTTGCACA   
       "CCTCACCGCT ACGGTATGCA TTTTTCCTTC TCTGAGTAT"C TTACCGTCTC CGTTTATCTC GAAAACGTGT   
  
142941 TTTTCGTTAA T"CCATGAACA GGATCTATAT AGACACATAG GCCCATGGAT CCATACATCT CGATCGGAAA "  
       AAAAGCAATT AGGTACTTGT CCTAGATATA TCTGT"GTATC CGGGTACCTA GGTATGTAGA GCTAGCCTTT "  
  
143011 "AGAATCAATA G"AAAAAGAAA GAATCGGAAT TGATCGATAT ATTTCTCGAA ACAAACGAAA ACGAAACGAA   
       "TCTTAGTTAT CTTTTTCTTT CTTAGCCTTA ACTAG"CTATA TAAAGAGCTT TGTTTGCTTT TGCTTTGCTT   
  
143081 AGACGAAACA TAAATCATGG ATCAACTAA"G CCAAGCCCTC TCGGGGACTT GCTTAAGAAT AAGAAAGAGG "  
       TCTGCTTTGT ATTTAGTACC TAGTTGATT"C GGTTCGGGAG AGCCCCTGAA CGAATTCTTA TTCTTTCTCC "  
  
143151 "AATCTCATGT AAATACCATG GAATAAGGT"T TGATCCTATT CATGGGGATT CCGTAAATAT TCCATTCCAA   
       "TTAGAGTACA TTTATGGTAC CTTATTCCA"A ACTAGGATAA GTACCCCTAA GGCATTTATA AGGTAAGGTT   
  
143221 AAAGAGAAAG TTCGAAACAA GTGGGATTTT TTTGGAGATT GGATGCAGTT ACTAATTCAT GATCTGGCAT   
       TTTCTCTTTC AAGCTTTGTT CACCCTAAAA AAACCTCTA"A CCTACGTCAA TGATTAAGTA CTAGACCGTA "  
  
143291 GTACAGAATG AAAACTTCAT TCTCGATTCT ACGAGAATTT TTATGAAAGC CTTTCATTTG CTTCTCTTCG   
       "CATGTCTTAC TTTTGAAGTA AGAGCTAAGA TGCTCTTAA"A AATACTTTCG GAAAGTAAAC GAAGAGAAGC   
  
143361 ATGGAAGTTT TATTT"TCCCA GAATGTATCC TAATTTTTGG CCTAATTCTT CTTCTGATGA TCGATTCAAC "  
       TACCTTCAAA ATAAAAGGGT CTTACATAGG ATTAAAAAC"C GGATTAAGAA GAAGACTACT AGCTAAGTTG "  
  
143431 "CTCTGATCAA AAAGA"TATAC CTTGGTTATA TTTCATCTCT TCAACAAGTT TAGTAATGAG CATAACGGCC   
       "GAGACTAGTT TTTCTATATG GAACCAATAT AAAGTAGAG"A AGTTGTTCAA ATCATTACTC GTATTGCCGG   
  
143501 CTATT"GTTCC GATGGAGAGA AGAACCTATG ATTAGCTTTT CGGGAAATTT CCAAACGAAC AATTTCAACG "  
       GATAACAAGG CTACCTCTCT TCTTGGATAC TAAT"CGAAAA GCCCTTTAAA GGTTTGCTTG TTAAAGTTGC "  
  
143571 "AAATC"TTTCA ATTTCTTATT TTACTATGTT CAACTCTATG TATTCCTCTA TCCGTAGAGT ACATTGAATG   
       "TTTAGAAAGT TAAAGAATAA AATGATACAA GTTG"AGATAC ATAAGGAGAT AGGCATCTCA TGTAACTTAC   
  
143641 TACAGAAATG GCTATAACAG AGTTTCTATT ATTCGTATTA ACAGCTACTC TAGGAGGAAT GTTTTTATGC   
       ATGTCTTTAC CGATATTGTC TCAAAGATAA TAAGCATAAT TGTCGAT"GAG ATCCTCCTTA CAAAAATACG "  
  
143711 GGTGCTAATG ATTTAATAAC TATCTTTGTA GCTCCAGAAT GTTTCAGTTT ATGCTCCTAC CTATTAT"CTG "  
       "CCACGATTAC TAAATTATTG ATAGAAACAT CGAGGTCTTA CAAAGTC"AAA TACGAGGATG GATAATAGAC   
  
143781 "GATATACCAA GAAAGATGTA CGGTCTAATG AGGCTACTAC GAAATATTTA CTCATGGGTG GGGCAAG"TTC   
       CTATATGGTT CTTTCTACAT GCCAGATTAC TCCGATGATG CTTTATAAA"T GAGTACCCAC CCCGTTCAAG "  
  
143851 TTCTATTCTG GTTCA"TGGTT TCTCTTGGCT ATATGGTTCA TCCGGGGGAG AGATCGAGCT TCAAGAAATA "  
       "AAGATAAGAC CAAGTACCAA AGAGAACCGA TATACCAAGT AGGCCCCCT"C TCTAGCTCGA AGTTCTTTAT   
  
143921 "GTAAATGGTC TTCTC"AATAC ACAAATGTAT AACTCCCCAG GAATTTCAAT TGCGCTTATA TTCATCACTG   
       CATTTACCAG AAGAGTTATG TGTTTACATA TTGAGGGGTC CTTAAAGTT"A ACGCGAATAT AAGTAGTGAC "  
  
143991 TAGGAATTGG GTTCAAGCTT TCCCCAGCCC CTTCTCATCA ATGGACTCCT GACGTATACG AAGGAGTGCG   
       "ATCCTTAACC CAAGTTCGAA AGGGGTCGGG GAAGAGTAGT TACCTGAGG"A CTGCATATGC TTCCTCACGC   
  
144061 GTTCGTTCGA TAAATTCCTA CCTCTCTATC TATCTCTG"AG ATGTTTGGAT TTTTCAAAAC TTCATGGACA "  
       CAAGCAAGCT ATTTAAGGAT GGAGAGATAG AT"AGAGACTC TACAAACCTA AAAAGTTTTG AAGTACCTGT "  
  
144131 "TGCAGAAGAG AAATACTATC CCCACTCGGA CCAAGACA"TA ACTTTTACTT GTTCAAATAA CAATTAAGGT   
       "ACGTCTTCTC TTTATGATAG GGGTGAGCCT GG"TTCTGTAT TGAAAATGAA CAAGTTTATT GTTAATTCCA   
  
144201 GAAGCAGGGT CAGGAACAAC GAATCTCTTT ATGATAAACA GATTCATTTT GCAAGT"TCGT TATTACGGGT "  
       CTTCGTCCCA GTCCTTGTTG CTTAGAGAAA TACTATTTGT CTAAGTAAA"A CGTTCAAGCA ATAATGCCCA "  
  
144271 "AGTTCCTACA AAGGATCGGA CTAATGACGT ATACAATACT TGAATTCTCG ATGTAG"ATGC TACATAGTTG   
       "TCAAGGATGT TTCCTAGCCT GATTACTGCA TATGTTATGA ACTTAAGAG"C TACATCTACG ATGTATCAAC   
  
144341 GTTCTCATCC TTCAGAGACT ACGAGTGTAA TAGGAGCATC CGTCGACAAA AG"GATCACCC TAAGATGATC "  
       CAAGAGTAGG AAGTCTCTGA TGCTCACATT ATCCTC"GTAG GCAGCTGTTT TCCTAGTGGG ATTCTACTAG "  
  
144411 "ATCACATGGC TATTGAGAAC GAATCAAATC AGATGGTTCT ATTTCTCAAT CT"TTCTGACT TGCTCCTACG   
       "TAGTGTACCG ATAACTCTTG CTTAGTTTAG TCTACC"AAGA TAAAGAGTTA GAAAGACTGA ACGAGGATGC   
  
144481 GAACTAAGGT CGAAAAGATT GAGAAAAATC AGTCATTCAC AACCACTGAT GAAGGATT"CC TCGAAAAGTT "  
       CTTGATTCCA GCTTTTCTAA CTCTTTTTAG TCAGTAAGTG T"TGGTGACTA CTTCCTAAGG AGCTTTTCAA "  
  
144551 "AAGGATTAGT AATCCTTTTT AGAAATCGAA TGGATTCGGT CTTATACATA CGCGAGGA"AG GTAATCAAAA   
       "TTCCTAATCA TTAGGAAAAA TCTTTAGCTT ACCTAAGCCA G"AATATGTAT GCGCTCCTTC CATTAGTTTT   
  
144621 AAGGAAAGAA GATGAGTTCT TCTTTCTTTT ATCACTTAGC TTAGGAGCCG "TGCGAGATGA AAGTCTCATG "  
       TTCCTTTCTT CTACTCAAGA AGAAAGAAAA TAGTGAATCG AATCCTCGG"C ACGCTCTACT TTCAGAGTAC "  
  
144691 "CACGGTTTTG AATGAGAGAA AGAAGTGAGG AATCCTCTTT TCGACTCTGA" CTCTCCCACT CCAGTCGTTG   
       "GTGCCAAAAC TTACTCTCTT TCTTCACTCC TTAGGAGAAA AGCTGAGAC"T GAGAGGGTGA GGTCAGCAAC   
  
144761 CTTTTCTTTC TGTTACTTCG AAAGTAGCTG CTTCAGCTTC AGCCACTCGA ATTTTCGATA TTCCTTTTTA   
       GAAAAGAAAG ACAATGAAGC TTTCATCGAC GAAGT"CGAAG TCGGTGAGCT TAAAAGCTAT AAGGAAAAAT "  
  
144831 TTT"CTCATCA AACGAATGGC ATCTTCTTCT GGAAATCCTA GCTATTCTTA GCATGATATT GGGGAATCTC "  
       "AAAGAGTAGT TTGCTTACCG TAGAAGAAGA CCTTT"AGGAT CGATAAGAAT CGTACTATAA CCCCTTAGAG   
  
144901 "ATT"GCTATTA CTCAAACAAG CATGAAACGT ATGCTTGCGT ATTCGTCCAT CGGTCAAATC GGATATGTAA   
       TAACGATAAT GAGTTTGTTC GTACTTTGCA TACGAACGCA TAAGCAG"GTA GCCAGTTTAG CCTATACATT "  
  
144971 TTATTGGAAT A"ATTGTTGGA GACTCAAATG GTGGATATGC AAGCATGATA ACTTATATGC TCTTCTATAT "  
       "AATAACCTTA TTAACAACCT CTGAGTTTAC CACCTATACG TTCGTAC"TAT TGAATATACG AGAAGATATA   
  
145041 "CGCCATGAAT C"TAGGAACTT TTGCTCGCAT TGTATCATTT GGTCTACGTA CCGGAACTGA TAACATTCGA   
       GCGGTACTTA GATCCTTGAA AACGAGCGTA ACATAGTAAA "CCAGATGCAT GGCCTTGACT ATTGTAAGCT "  
  
145111 GATTATGCAG GATTATACAC AAAAGATCCT CTTTTGGCTC TCTCTTTAGC CCTATGTCTC TTATCCTTAG   
       "CTAATACGTC CTAATATGTG TTTTCTAGGA GAAAACCGAG" AGAGAAATCG GGATACAGAG AATAGGAATC   
  
145181 GAGGTCTTCC TCCACTAGCA GGTTTTTTCG GAAAACTTCA TT"TATTCTGG TGTGGATGGC AGGCAGGCCT "  
       CTCCAGAAGG AGGTGATCGT CCAAAAAAGC CTTTTGAAGT AA"ATAAGACC ACACCTACCG TCCGTCCGGA "  
  
145251 "ATATTTCTTG GTTTCAATAG GACTACTTAC GAGCGTTGTT TC"TATCTACT ATTATCTAAA AATAATCAAG   
       "TATAAAGAAC CAAAGTTATC CTGATGAATG CTCGCAACAA AG"ATAGATGA TAATAGATTT TTATTAGTTC   
  
145321 TTATTAATGA CTGGACGAAA CCAAGAAATA A"CCCCTCACG TGCGAAATTA TAGAAGATCT CCTTTAAGAT "  
       AATAATTACT GACCTGCTTT GGTTCTTTAT T"GGGGAGTGC ACGCTTTAAT ATCTTCTAGA GGAAATTCTA "  
  
145391 "CAAACAATTC CATCGAATTG AGTATGATTG T"ATGTGTGAT AGCATCTACT ATACCAGGAA TATCAATGAA   
       "GTTTGTTAAG GTAGCTTAAC TCATACTAAC A"TACACACTA TCGTAGATGA TATGGTCCTT ATAGTTACTT   
  
145461 CCCGATTATT GAAATTGCTC AGGATACCCT TTTTTAGTTT AGCTT"CTAGA ATCTATTTCT TAGTTCAAGA "  
       GGGCTAATAA CTTTAACGAG TCCTATGGGA AAAAATCAAA TCGAA"GATCT TAGATAAAGA ATCAAGTTCT "  
  
145531 "TCCCTCTTAC TAACTGGAAT CAAAGAATTA GTAGATCTGT TCCGC"CCAAA ATGGGAATGG GCTAGGGTTA   
       "AGGGAGAATG ATTGACCTTA GTTTCTTAAT CATCTAGACA AGGCG"GGTTT TACCCTTACC CGATCCCAAT   
  
145601 TGAACTTATA ATCTATAATC TGATGATCGA GTCGATTCCA TGATTATAAG TTCATT"CCAT ACCGGGCCAG "  
       ACTTGAATAT TAGATATTAG ACTACTAGCT CAGCTAA"GGT ACTAATATTC AAGTAAGGTA TGGCCCGGTC "  
  
145671 "ACCGGAATAG GGCTAGGTTA TATACATTCT AATTATGAGA AGGGGTCATC CGAGCG"TATT TAAATAGATA   
       "TGGCCTTATC CCGATCCAAT ATATGTAAGA TTAATAC"TCT TCCCCAGTAG GCTCGCATAA ATTTATCTAT   
  
145741 CTATGTTTAC ATATGGATCC CTACGTCCTT ACATTCCATT T"AGGATTAGG AATAGGTGTA ATCGGACCTG "  
       GATACAAATG TATACCTAGG GATGCAGGAA TGTAAGGTAA A"TCCTAATCC TTATCCACAT TAGCCTGGAC "  
  
145811 "TTTTTTACAT ATCTCTCGTT ATTTGGGACC CTATTCATCT C"TTTGGGCTT CTATTGAATC GAGAAATAGG   
       "AAAAAATGTA TAGAGAGCAA TAAACCCTGG GATAAGTAGA G"AAACCCGAA GATAACTTAG CTCTTTATCC   
  
145881 TTTGATTGTC CATCTTTTTG ATATAATAGT AAGGCATCCT CCGGATAATT CAAA"TCGAAG CAATTGGATG "  
       AAACTAACAG GTAGAAAAAC TATATTATCA TTC"CGTAGGA GGCCTATTAA GTTTAGCTTC GTTAACCTAC "  
  
145951 "TCCGACTCGG ACCTATATGA CATGACCGAG CAATAGAAAT ACTCTAACAC TCCA"CCTTTG TCATATATTC   
       "AGGCTGAGCC TGGATATACT GTACTGGCTC GTT"ATCTTTA TGAGATTGTG AGGTGGAAAC AGTATATAAG   
  
146021 CATACATCAC ACTAGATAGA TATCATATTC ATGGAATATG ATTCACTTTC AAGATGCCTT GGT"GGTGAAA "  
       GTATGTAGTG TGATCTATCT ATAGTATAAG TACCTTATAC T"AAGTGAAAG TTCTACGGAA CCACCACTTT "  
  
146091 "TGGTAGACAC GCGAGACTCA AAATCTCGTG CTAAAGAGCG TGGAGGTTCG AGTCCTCTTC AAG"GCATAAT   
       "ACCATCTGTG CGCTCTGAGT TTTAGAGCAC GATTTCTCGC A"CCTCCAAGC TCAGGAGAAG TTCCGTATTA   
  
146161 GTTGAGAATG CCCGTTGAAT T"GGAAGGAAT AAGTTCGGCA GCGGATCACG AAATCTTGGC GATCTTCTCT "  
       CAACTCTTAC GGGCAACTTA ACCTTCCTTA TTCAAGCCGT CGCC"TAGTGC TTTAGAACCG CTAGAAGAGA "  
  
146231 "ATCTAATGAA TGGGAGTCCG C"TTTGAAATC GTCCGCCCTG CACCCACCCC CCGAGTATAT GCTTCAACAG   
       "TAGATTACTT ACCCTCAGGC GAAACTTTAG CAGGCGGGAC GTGG"GTGGGG GGCTCATATA CGAAGTTGTC   
  
146301 GAATTAAACA AGGGTAGATT GATA"CAATAG AAACCTCTGG TAAAATGCCC ACCCGTAACC CAGCAGATAA "  
       CTTAATTTGT TCCCATCTAA CTATGTTATC TTTGGAGACC ATTTTAC"GGG TGGGCATTGG GTCGTCTATT "  
  
146371 "AGTACATTAC ATAGTCCGTT TTAG"GGATTG GCGACTTACC CATTCAGTGA CTTTGGCACT GGACGTTCCA   
       "TCATGTAATG TATCAGGCAA AATCCCTAAC CGCTGAATGG GTAAGTC"ACT GAAACCGTGA CCTGCAAGGT   
  
146441 AAAATGGGG"T ACTATCGGGT CGGGTGAATT CAATAATAGA GGCCTGTTGG CATTCCAGCC TTCCTTCTCC "  
       TTTTACCCCA TGATAGCCCA GCCCACTTAA GTTATTATC"T CCGGACAACC GTAAGGTCGG AAGGAAGAGG "  
  
146511 "TTTCAGGGC"C TACCCGAAAG AGAATCCAGT GTTTCTTGGT CGTGAATATC TGAATAGGAC GAACCGCCCC   
       "AAAGTCCCGG ATGGGCTTTC TCTTAGGTCA CAAAGAACC"A GCACTTATAG ACTTATCCTG CTTGGCGGGG   
  
146581 GTGGTTTGCT TCGGAACAAA ACAATT"AGAA TTAGGCTCGT TGAACTGGAA TGTGTATTAT CCATATAGGG "  
       CACCAAACGA AGCCTTGTTT TGTTAATCTT AAT"CCGAGCA ACTTGACCTT ACACATAATA GGTATATCCC "  
  
146651 "GATCTTTCAA TGGAGAAGAT CCGTCG"ACCT AAGACGAAGA GAGGGGTCTA TCTATTTTAT TTAGTTATTC   
       "CTAGAAAGTT ACCTCTTCTA GGCAGCTGGA TTC"TGCTTCT CTCCCCAGAT AGATAAAATA AATCAATAAG   
  
146721 AGTTAAACCA ATGATTCGTT AT"TGTAACAG ATAGCAACAA CCATTTCATT CGGCATGCGT ATTTTTGATT "  
       TCAATTTGGT TACTAAGCAA TAACATTGTC TAT"CGTTGTT GGTAAAGTAA GCCGTACGCA TAAAAACTAA "  
  
146791 "TTCCGATGGA TTTACATCTT TC"ATTAATGG AAATTTTTTG ATGTAGTGAG TAATAACTCT GGTTGTTCGC   
       "AAGGCTACCT AAATGTAGAA AGTAATTACC TTT"AAAAAAC TACATCACTC ATTATTGAGA CCAACAAGCG   
  
146861 TGTTCAAGAA TTCTGGTTTA "GGCAGTTCAT ACCATCAAAA CATTATGTAT GGATCTAAGA TTTCAATTCT "  
       ACAAGTTCTT AAGACCAAAT CCGTCAAGTA TGGTAGTTTT GTAATACAT"A CCTAGATTCT AAAGTTAAGA "  
  
146931 "TCCATGTTTC AGCAGTAGTA" TATTGTTCCA TGGAGCTAAG GTCCAAAATA TGGAAGAAAC TGGTGTTTTC   
       "AGGTACAAAG TCGTCATCAT ATAACAAGGT ACCTCGATTC CAGGTTTTA"T ACCTTCTTTG ACCACAAAAG   
  
147001 ACGACTCTAC CACCC"AGTCA ATTCTGTTCC ACTTAATCCC TATTTCCATG GCCACATATT TTTCCGGCTA "  
       TGCTGAGATG GTGGGTCAGT TAAGACAAG"G TGAATTAGGG ATAAAGGTAC CGGTGTATAA AAAGGCCGAT "  
  
147071 "AAGGAATGGG AAACC"TTTCT CTTGTTACAT GAATCCAATT TTCATTTCAT TTCATCCGGG AAAAGCCATC   
       "TTCCTTACCC TTTGGAAAGA GAACAATGTA C"TTAGGTTAA AAGTAAAGTA AAGTAGGCCC TTTTCGGTAG   
  
147141 TTTTTCTCAA CAAT"GTCTTT GTCATTTGAT CCAATAGTGT TCCGTTAGAT AGGAACAGAT TTGATAAATA "  
       AAAAAGAGTT GTTACAGAAA CAGTAAACTA GGTTATCACA AGGCAATCTA TCCTTGTCTA AACTATTTAT   
  
147211 "CTGATAACTC TCGG"ATAGAG TATTAGAACG GAAAAATCCA TTAGATTTAG ATAATGAACT ATTGGTTCTA   
       GACTATTGAG AGCCTATCTC ATAATCTTGC CTTTTTAGGT AATCTAAATC TATTACTTGA TAACCAAGAT   
  
147281 GTTCTAAGCC ATCTCTGGCG AT"GAATCAAC AATTCGACGT ACTTTTCTTG CGTATTCTTG ATATTGATAA "  
       CAAGATTCGG TAGAGACCGC TACTTAGTTG TTAAGCTGCA TGAAAAGAAC GCATAAGAAC TATAACTATT   
  
147351 "ACCAGCGTTT TTCTAGAGAT GT"AGGAAAAG CTGGTTGGGA AGTAAGCAGC CCCTTTGCCA TCTCTTCATC   
       TGGTCGCAAA AAGATCTCTA CATCCTTTTC GACCAACCCT TCATTCGTCG GGGAAACGGT AGAGAAGTAG   
  
147421 TGCAAAGAA"T TCTCGATGTG AAAACACAGA GACAAAAGGA TGATCTTGGA ATAGGAAAAA GAGTGGATCT "  
       ACGTTTCTTA AGAGCTACAC TTTTGTGTCT CTGTTTTCCT ACTAGAACCT TATCCTTTTT CTCACCTAGA   
  
147491 "GCAGGATCC"C AAATGAATTG GTTTATTAGA AAAAAGCCTT GTTCTTTGGA AGAGCTATCT CGTGTCTGGT   
       CGTCCTAGGG TTTACTTAAC CAAATAATCT TTTTTCGGAA CAAGAAACCT TCTCGATAGA GCACAGACCA   
  
147561 ACTGCACGGT TCCACTCTG"C AAGAACTCCG AATCATTCTC TTGAAGCTCA TCCTCTTCAT CATAAATGAT "  
       TGACGTGCCA AGGTGAGACG TTCTTGAGGC TTAGTAAGAG AACTTCGAGT AGGAGAAGTA GTATTTACTA   
  
147631 "CCGCTTGCCC CGAAATGAC"C TAGCCCAATA GGGAACTCCC AATTCATTGG GCCTTTCGAT ACAATCAAAT   
       GGCGAACGGG GCTTTACTGG ATCGGGTTAT CCCTTGAGGG TTAAGTAACC CGGAAAGCTA TGTTAGTTTA   
  
147701 AGAAAGCCCC AAGGGCTCCA T"ATTCTAGGA GCCCAAACTA TGTGATTGAA TAAATCCTCC TCTATCTGTT "  
       TCTTTCGGGG TTCCCGAGGT ATAAGATCCT CGGGTTTGAT ACACTAACTT ATTTAGGAGG AGATAGACAA   
  
147771 "GCGGGTCGAG GACTCCTTCC C"CTTCTTCAA ACTTCGATTC GTATTTTTGA TAGAGAAATA TCTGATCAAC   
       CGCCCAGCTC CTGAGGAAGG GGAAGAAGTT TGAAGCTAAG CATAAAAACT ATCTCTTTAT AGACTAGTTG   
  
147841 GATAGAAGAA GATCCATT"TT GTATCATATC TAAGGGATTC CTGGGTTCGG GCCGAAGAAG CAATGTCACT "  
       CTATCTTCTT CTAGGTAAAA CATAGTATAG ATTCCCTAAG GACCCAAGCC CGGCTTCTTC GTTACAGTGA   
  
147911 "CGATCATTAT CAAATCGA"CT GCAATCTTTT TCTGTCCATG AGGATCCAAA CAGAGCGCCT TCTACTTCTA   
       GCTAGTAATA GTTTAGCTGA CGTTAGAAAA AGACAGGTAC TCCTAGGTTT GTCTCGCGGA AGATGAAGAT   
  
147981 ATAGGCCATG AACTAG"ATGA GAATCATTCT CAACGAGTAC ATAAGAAGTG ATCCCATTTT TTTCATCGGG "  
       TATCCGGTAC TTGATCTACT CTTAGTAAGA GTTGCTCATG TATTCTTCAC TAGGGTAAAA AAAGTAGCCC   
  
148051 "TCCGGATAGA GACCAA"AGGT CTTGAACGAC CGATCCGGCA GAACAATTCA AAAGATAAAG AAGTATCGTT   
       AGGCCTATCT CTGGTTTCCA GAACTTGCTG GCTAGGCCGT CTTGTTAAGT TTTCTATTTC TTCATAGCAA   
  
148121 AATTTCTTCA "TGCTCGTTTC AAGTTCGAAG TACCAGTTGT ACAAATAAGA ACCCCCTTCG TTACATGATT "  
       TTAAAGAAGT ACGAGCAAAG TTCAAGCTTC ATGGTCAACA TGTTTATTCT TGGGGGAAGC AATGTACTAA   
  
148191 "TCTTCTTCAT" ATAGATAGAT ATAGGATCTA TGGAGCAATT ACTTAGAAGT ACATTTTGTG CAAGAGCCCT   
       AGAAGAAGTA TATCTATCTA TATCCTAGAT ACCTCGTTAA TGAATCTTCA TGTAAAACAC GTTCTCGGGA   
  
148261 TCCTATCTGA TA"GAAAAGGA TCCCATGATC CTGAACTGAT CTTACCTGGG ATCGCAAATC CCAAGTTTGT "  
       AGGATAGACT ATCTTTTCCT AGGGTACTAG GACTTGACTA GAATGGACCC TAGCGTTTAG GGTTCAAACA   
  
148331 "CTATGAAGAG CA"GATCTAAT TATATTAGTG TCTATAATTG ATTTCCTCTG CGTAATACTA ATCGATAGGG   
       GATACTTCTC GTCTAGATTA ATATAATCAC AGATATTAAC TAAA"GGAGAC GCATTATGAT TAGCTATCCC "  
  
148401 CCTCATTGGT AAGTGCTACA AGATCTCGT"A CATTGGAACC CATGGTTATG GAACCGAATC CATTAGTATG "  
       "GGAGTAACCA TTCACGATGT TCTAGAGCAT GTAACCTTGG GTAC"CAATAC CTTGGCTTAG GTAATCATAC   
  
148471 "GAACATTTTC TTTTCCAAGT GAAATCCCC"T AGTATATGAA AGAGTGAAAA AGTGCTTTCG TTGTTGTGGA   
       CTTGTAAAAG AAAAGGTTCA CTTTAGGGGA TCATATACTT TCTCACTTTT TCACGAAAGC AACAACACCT   
  
148541 ATAAGAAGCC TTCGTATTTT AATGCAT"GTA TTCAATTTAT TCGGAGCTAT TAGAGCGGGA TCCACTTTTT "  
       TATTCTTCGG AAGCATAAAA TTACGTACAT AAGTTAAATA AGCCTCGATA ATCTCGCCCT AGGTGAAAAA   
  
148611 "GGGGAATATG GGTCGAAGCA ATAACAA"GAA TATTTGTAGT GGAACATCTT TCAAAATCCC TGTAGAGATA   
       CCCCTTATAC CCAGCTTCGT TATTGTTCTT ATAAACATCA CCTTGTAGAA AGTTTTAGGG ACATCTCTAT   
  
148681 GTTCACTAAT A"GACCAAAGT ATAAGTGACT CGACTTATTC ACATACAGAT CATGAATGTT TGGAATCCAT "  
       CAAGTGATTA TCTGGTTTCA TATTCACTGA GCTGAATAAG TGTATGTCTA GTACTTACAA ACCTTAGGTA   
  
148751 "ATTATGCAAG G"AGACATTGC TTTTATTAAT TCGAATTGAA GGGTGATATA AAATTGGTCT ATTTCCAGCT   
       TAATACGTTC CTCTGTAACG AAAATAATTA AGCTTAACTT CCCACTATAT TTTAACCAGA TAAAGGTCGA   
  
148821 TCATCTCCA"T AGTTAGCGCA TTCATCATAG TTAGAAGCTC CAGCTCCGTA TCAATATCAA GGTCACGGTC "  
       AGTAGAGGTA TCAATCGCGT AAGTAGTATC AATCTTCGAG GTCGAGGCAT AGTTATAGTT CCAGTGCCAG   
  
148891 "GATATCGTC"A CTATCATCAA TATCGCCACT ATCATCAATA TCGTCACTAT CATCGTCACT ATCATCAATA   
       CTATAGCAGT GATAGTAGTT ATAGCGGTGA TAGTAGTTAT AGCAGTGATA GTAGCAGTGA TAGTAGTTAT   
  
148961 AACTTAGGCT TGTTATCCCG GAACT"TGTTC AGAAATACTG TAATGAAAGG AAGATAGGAG TTTGTCGCTA "  
       TTGAATCCGA ACAATAGGGC CTTGAACAAG TCTTTATGAC ATTACTTTCC TTCTATCCTC AAACAGCGAT   
  
149031 "GGTATTTGAC CAAATAGGAT CGTCC"AGTTC CTATAGAACC TATCACTAAA ATACCCGGGG AGAGGGCTAA   
       CCATAAACTG GTTTATCCTA GCAGGTCAAG GATATCTTGG ATAGTGATTT TATGGGCCCC TCTCCCGATT   
  
149101 GCGGAGCGAA AAGAGTTTTC "CATGAGATGG GAAATGAAAA CTATTAGCCC CACACGAGGT TTGTGAATGA "  
       CGCCTCGCTT TTCTCAAAAG GTACTCTACC CTTTACTTTT GATAAT"CGGG GTGTGCTCCA AACACTTACT "  
  
149171 "GTGATTGTCT GATAATGAGC" AAGGAATGTC CGTCTTTCTG CTAAACAGGA TGTATTGAAC TTATAATTCA   
       "CACTAACAGA CTATTACTCG TTCCTTACAG GCAGAAAGAC GATTTG"TCCT ACATAACTTG AATATTAAGT   
  
149241 TTAGATACTT TTTCTGAATG TCAACTAAG"T ATCGTAAGTA AATTACTCCC GGTTGTTCAA TCATTTGATA "  
       AATCTATGAA AAAGACTTAC AGTTGATTCA TAGCATTCAT TTAATGAGGG CCAACAAGTT AGTAAACTAT   
  
149311 "ACCAGAGTCA TTCTTTGATA AATGATCAC"T ATGAGTCAGA CTCAATAGAA TTTGATCAAT CCTTTTTTCT   
       TGGTCTCAGT AAGAAACTAT TTACTAGTGA TACTCAGTCT GAGTTATCTT AAACTAGTTA GGAAAAAAGA   
  
149381 GTCGTTAAG"G TGGAGAACTG AACCAAGAAT TCTCTTTCTT TATCATCAAT CGAATCACTG TTCCAGACCC "  
       CAGCAATTCC ACCTCTTGAC TTGGTTCTTA AGAGAAAGAA ATAGTAGTTA GCTTAGTGAC AAGGTCTGGG   
  
149451 "AGGATTGGA"T TTTATCATCA ATCCAATCAC CGTTCACATT TTTTCTTATC AATGAATATA TCTCTTTACT   
       TCCTAACCTA AAATAGTAGT TAGGTTAGTG GCAAGTGTAA AAAAGAATAG TTACTTATAT AGAGAAATGA   
  
149521 TGTATGACTT AGATG"TCTCG TATTTCTCGA AAAAGCGATT CGATTGATGG GATTTGGTAT GATACTTATG "  
       ACATACTGAA TCTACAGAGC ATAAAGAGCT TTTTCGCTAA GCTAACTACC CTAAACCATA CTATGAATAC   
  
149591 "AGATTGATGA GATTG"ATATT CCAATCTTTC TTCTTCGAAC GTATTGATTT GACCCCATAA GCGGGACCGC   
       TCTAACTACT CTAACTATAA GGTTAGAAAG AAGAAGCTTG CATAACTAAA CTGGGGTATT CGCCCTGGCG   
  
149661 CACCAGAAGC AGAACCCCAT ATTTC"TTCTA GAGAATCTCC TAATTGTTCC AGAGCAACTA GAAAGAGATT "  
       GTGGTCTTCG TCTTGGGGTA TAAAGAAGAT CTCTTAGAGG ATTAACAAGG TCTCGTTGAT CTTTCTCTAA   
  
149731 "CTTTAACCAG AAAGAATTCC GTTCA"TATGT GGGATACCTA TCCAGAAGTT TTCGCAACTC AATCATGTAT   
       GAAATTGGTC TTTCTTAAGG CAAGTATACA CCCTATGGAT AGGTCTTCAA AAGCGTTGAG TTAGTACATA   
  
149801 GATGGAATCA TCAAAGATT"T GACCTTTTCG AACTCTGTCT GTAACTCACT ATAGGCTCGA GAAACAAAGA "  
       CTACCTTAGT AGTTTCTAAA CTGGAAAAGC TTGAGACAGA CATTGAGTGA TATCCGAGCT CTTTGTTTCT   
  
149871 "GAAGATGTGT ACGAACGAG"A TATACAGCAA TAAGAAGAAG GAAAAGGATT GAATAGAGGA ACTCCCGAAC   
       CTTCTACACA TGCTTGCTCT ATATGTCGTT ATTCTTCTTC CTTTTCCTAA CTTATCTCCT TGAGGGCTTG   
  
149941 ATTTGGCGAT C"TCAGATGTG TCGATATCGA TGGTGACTCA TTATTTCGAT GAATCATTTC TTCGGACAGA "  
       TAAACCGCTA GAGTCTACAC AGCTATAGCT ACCACTGAGT AATAAAGCTA CTTAGTAAAG AAGCCTGTCT   
  
150011 "AGAAGATTAG G"TAAACACTT AATCGAAATC TCACTTATCA GATTCCATTG TGGAAGACAC AATTTTTTCC   
       TCTTCTAATC CATTTGTGAA TTAGCTTTAG AGTGAATAGT CTAAGGTAAC ACCTTCTGTG TTAAAAAAGG   
  
150081 GAAGAATTCG CCATGATCTA TCTGAT"CGAT GCATAATATC ATCAAAAATG GATACAAATT TTGGGCTGCT "  
       CTTCTTAAGC GGTACTAGAT AGACTAGCTA CGTATTATAG TAGTTTTTAC CTATGTTTAA AACCCGACGA   
  
150151 "ACTTAGTATC GGCAATAGGT CTGAAA"AAGT ATCTAAAAAT AGCAAATTTA GATATTTGTA CCCTGTCGAA   
       TGAATCATAG CCGTTATCCA GACTTTTTCA TAGATTTTTA TCGTTTAAAT CTATAAACAT GGGACAGCTT   
  
150221 GTAAAGAACC ATGGCATATA TGTTTG"GAAT AGATTCCATT TTGAGAGAGT TGAAAAAGCA CTATCTCGTT "  
       CATTTCTTGG TACCGTATAT ACAAACCTTA TCTAAGGTAA AACTCTCTCA ACTTTTTCGT GATAGAGCAA   
  
150291 "GAAAGGTTCT ATACATCTGC CCTTTC"TCAA GGCATTTCTT TCGATAAAGA CTCTGTTTTT TCCTCTTTTC   
       CTTTCCAAGA TATGTAGACG GGAAAGAGTT CCGTAAAGAA AGCTATTTCT GAGACAAAAA AGGAGAAAAG   
  
150361 GGATGGTAAA TATTTCTCAG AA"CATGGAGT GTGAATCAAA CCCATGTTTG AATTGAAATT GAGATACTGA "  
       CCTACCATTT ATAAAGAGTC TTGTACCTCA CACTTAGTTT GGGTACAAAC TTAACTTTAA CTCTATGACT   
  
150431 "TGCAAGTTCT TCCCCTCTGA AT"CAGATAGA TTCATATCTG AAAGAGGTTG ACAATAAGTT CTTTCAAAAT   
       ACGTTCAAGA AGGGGAGACT TAGTCTATCT AAGTATAGAC TTTCTCCAAC TGTTATTCAA GAAAGTTTTA   
  
150501 TGACTATTTG TCCCTC"TGTT AGAGGTGTTA CAGAAATGTC TGCGATCGAG TAAATAGCTC GACGAACGAA "  
       ACTGATAAAC AGGGAGACAA TCTCCACAAT GTCTTTACAG ACGCTAGCTC ATTTATCGAG CTGCTTGCTT   
  
150571 "TGGATTGGAT CGAATT"GGAA AATGGAAAGA TTTGTACAAG TTATACCTTT CGTCACCACT TTGTGGAAAA   
       ACCTAACCTA GCTTAACCTT TTACCTTTCT AAACATGTTC AATATGGAAA GCAGTGGTGA AACACCTTTT   
  
150641 TCGTTAGGTA TGAATATGTT AGATA"CCTGT GACTCGATTG GTGAAATAGT ATCTCTCTCC AAAAAAGCAT "  
       AGCAATCCAT ACTTATACAA TCTATGGACA CTGAGCTAAC CACTTTATCA TAGAGAGAGG TTTTTTCGTA   
  
150711 "GTTTTTTTTT ACCGCCGCAC AAAGA"AAATA TTTTGTTGCG AATGAACAAG ATATTGAGGA ATTGTCCATA   
       CAAAAAAAAA TGGCGGCGTG TTTCTTTTAT AAAACAACGC TTACTTGTTC TATAACTCCT TAACAGGTAT   
  
150781 CGTAAAATCA TAATTA"TTGA TACGGGCCTT TTCCACATAA TCCACATAAA AAGGGAATCT TTTGTTACAA "  
       GCATTTTAGT ATTAATAACT ATGCCCGGAA AAGGTGTATT AGGTGTATTT TTCCCTTAGA AAACAATGTT   
  
150851 "TAGAAGCAGA AGTGAT"GTGG ATTATTCAAG AATCGAAGTC GAGTTGCTTT ATAAAAAGAA GATATCAATG   
       ATCTTCGTCT TCACTACACC TAATAAGTTC TTAGCTTCAG CTCAACGAAA TATTTTTCTT CTATAGTTAC   
  
150921 AACTTCTATG AAATGGTTT"C ACGGGATTTA GCCAATTGTC TTGATCGTGG GATATCATTG AGAAATAGGA "  
       TTGAAGATAC TTTACCAAAG TGCCCTAAAT CGGTTAACAG AACTAGCACC CTATAGTAAC TCTTTATCCT   
  
150991 "ATCCGTGTTA TCAAAGGAT"T TCCTGCGATT ATTTCTAGTA TCGAATGAGT CAATCATCCA CTTTGGTATC   
       TAGGCACAAT AGTTTCCTAA AGGACGCTAA TAAAGATCAT AGCTTACTCA GTTAGTAGGT GAAACCATAG   
  
151061 TTATTGAACA AAAATGGTGA TATT"GTTCCT CCATTGATCA AGAATTTCGA TTTTTGGGAA GTATAATGAT "  
       AATAACTTGT TTTTACCACT ATAACAAGGA GGTAACTAGT TCTTAAAGCT AAAAACCCTT CATATTACTA   
  
151131 "CATCCAATAA GAAGGGTTTC AATT"TTTTCA AATGAACGAT TTGAAGACCT ATTGATTCTA ACAACTGATT   
       GTAGGTTATT CTTCCCAAAG TTAAAAAAGT TTACTTGCTA AACTTCTGGA TAACTAAGAT TG"TTGACTAA "  
  
151201 GCAGAGTCGA TCAT"TCGGAC CTTTCAATTC ATAGATGTGG ATCTCGGACC TATGAATGGG GATATTCCCG "  
       "CGTCTCAGCT AGTAAGCCTG GAAAGTTAAG TATCTACACC TAGAGCCTGG ATACTTACCC CT"ATAAGGGC   
  
151271 "AAACTCACAA AGAA"AAAAGG AAGTGAGTTA GACAAAAAGA GAAGAAACTT GGACAAAAAA ACAAGTAACT   
       TTTGAGTGTT TCTTTTTTCC TTCACTCAAT CTGTTTTTCT CTTCTTTGAA CCTGTTTTTT TGTTCATTGA   
  
151341 TGGACAAAAA GAAACGAAGT GACT"TAGACC AATCTTTTTT ATCGATAACC TCAGACCAAT CAATCGAATA "  
       ACCTGTTTTT CTTTGCTTCA CTGAATCTGG TTAGAAAAAA TAGCTATTGG AGTCTGGTTA GTTAGCTTAT   
  
151411 "TTGATTAATA CGTAATCGAT CGAA"CACTAC TTGAAAATGA AAATGGCTCT TCTGCTCAGA AATGAAATGT   
       AACTAATTAT GCATTAGCTA GCTTGTGATG AACTTTTACT TTTACCGAGA AGACGAGTCT TTACTTTACA   
  
151481 TCCTGGAAAT TCTT"GCTCCC ATTGGACCAT TTGTATCTAT ATGCATTAGG ATCCCGATTC ATGGATCTCT "  
       AGGACCTTTA AGAACGAGGG TAACCTGGTA AACATAGATA TACGTAATCC TAGGGCTAAG TACCTAGAGA   
  
151551 "CGGTTCGAGA AATC"AAAATA AGAGGATCGA ACCATTTCTT CTGACCCTTT TTGAAATTTG ATAAATGTTG   
       GCCAAGCTCT TTAGTTTTAT TCTCCTAGCT TGGTAAAGAA GACTGGGAAA AACTTTAAAC TATTTACAAC   
  
151621 GTTGATCGTA TATTTCATTA T"AGTTCTATG ATTCAGAGTA TCATTTCCTA TTTGATCCCT TTGAATTCCA "  
       CAACTAGCAT ATAAAGTAAT ATCAAGATAC TAAGTCTCAT AGTAAAGGAT AAACTAGGGA AACTTAAGGT   
  
151691 "TATTCCAAGT CGCGATCGGA T"CTATTCATT AAAAAGAATC GATTCAATAC ATTTCTTATG TACCTATAAT   
       ATAAGGTTCA GCGCTAGCCT AGATAAGTAA TTTTTCTTAG CTAAGTTATG TAAAGAATAC ATGGATATTA   
  
151761 ATTGGATTT"G AATCAGATTT CGGATCAATC TATATTGATT GACTGCCTCC ATTATGTTGT TGCTAGCAAA "  
       TAACCTAAAC TTAGTCTAAA GCCTAGTTAG ATATAACTAA CTGACGGAGG TAATACAACA ACGATCGTTT   
  
151831 "TACCACTAT"T TTTTGTTTTG GATCTTCCAA ATCATTCCCG GGGGAGGTCC GGACCCATTT TTTTCTGATC   
       ATGGTGATAA AAAACAAAAC CTAGAAGGTT TAGTAAGGGC CCCCTCCAGG CCTGGGTAAA AAAAGACTAG   
  
151901 CTTCGATAAA AAGAGTCAT"T CTCTTCATAA AAAATAGGAG GTAGAACCAA TAAATATTTC TTTTTCGATT "  
       GAAGCTATTT TTCTCAGTAA GAGAAGTATT TTTTATCCTC CATCTTGGTT ATTTATAAAG AAAAAGCTAA   
  
151971 "CATCCCTGGA GTTGAATAC"C TCATTCAAGA ATTGTTTTTG ATCCAATCCG TAGGAATCAA TAGAAAAGGC   
       GTAGGGACCT CAACTTATGG AGTAAGTTCT TAACAAAAAC TAGGTTAGGC ATCCTTAGTT ATCTTTTCCG   
  
152041 AAATCCCTTA TGATAC"ACCA GATCCGGCTC GGTTATTGAT AGAGTGAATA GATCTGCCAT TTCTTGAAAT "  
       TTTAGGGAAT ACTATGTGGT CTAGGCCGAG CCAATAACTA TCTCACTTAT CTAGACGGTA AAGAACTTTA   
  
152111 "CTCTCTTCTG ATTCAA"AATC GTGGTGTAAC GTGTATCCCC TCCTGTTCCG GTCATGGAAT AGATGAAATA   
       GAGAGAAGAC TAAGTTTTAG CACCACATTG CACATAGGGG AGGACAAGGC CAGTACCTTA TCTACTTTAT   
  
152181 AATAAATAAA TGGATTTTTC TTCA"AGAATG AAATCTTATT GGAACTGTCC ATATCCAGTT CATCCTTCGG "  
       TTATTTATTT ACCTAAAAAG AAGTTCTTAC TTTAGAATAA CCTTGACAGG TATAGGTCAA GTAGGAAGCC   
  
152251 "AACCATATCA CATCCCGGAT CTGA"TGAAAT AGGATGAATT GAGACGGTAT TTTGTAAATA CGTAATTATC   
       TTGGTATAGT GTAGGGCCTA GACTACTTTA TCCTACTTAA CTCTGCCATA AAACATTTAT GCATTAATAG   
  
152321 TTGAATATAT TAACTATTTC TTTATTTTC"C GATCGCCTGG AAGGGACAAA AGAAACATCT TGTTCTTTCT "  
       AACTTATATA ATTGATAAAG AAATAAAAGG CTAGCGGACC TTCCCTGTTT TCTTTGTAGA ACAAGAAAGA   
  
152391 "TCAACAATTT CTGATCTCTA GTGGACCTC"T CAATAGGATT CGAACCCATA TGAAGTTCTG ACCATCTGTC   
       AGTTGTTAAA GACTAGAGAT CACCTGGAGA GTTATCCTAA GCTTGGGTAT ACTTCAAGAC TGGTAGACAG   
  
152461 AGAGAAAAAA GAAC"GAATGG ATCTTGTAGG ATTCCCGAGA AATTCTTCGA TTTCTTCCGG AAGCGGTTGT "  
       TCTCTTTTTT CTTGCTTACC TAGAACATCC TAAGGGCTCT TTAAGAAGCT AAAGAAGGCC TTCGCCAACA   
  
152531 "TGAATCTCTT TTTG"ATTGAT CAATGTGTGA TATTCCGAAT CCTCATTACT AATGGAATCG AAATGATCTC   
       ACTTAGAGAA AAACTAACTA GTTACACACT ATAAGGCTTA GGAGTAATGA TTACCTTAGC TTTACTAGAG   
  
152601 TGGATTGATC AGAAGATCCT "TTCAATTGGC TAGAATCCGT TACTTGAACG AAACTAGTTG GAAGATACAG "  
       ACCTAACTAG TCTTCTAGGA AAGTTAACCG ATCTTAGGCA ATGAACTTGC TTTGATCAAC CTTCTATGTC   
  
152671 "GAGCAAAACA ATCAACCTAT" TGATATTGGA AGAGCCAAAA GATTCTTCCA ATGTATCATT TCTGGGTCCA   
       CTCGTTTTGT TAGTTGGATA ACTATAACCT TCTCGGTTTT CTAAGAAGGT TACATAGTAA AGACCCAGGT   
  
152741 ATGAAATTCA TAGGTATAGG AAGAAA"CCCT GTCAAATAGA GATTTTTTCT TTCGACCATC TTTCGATTGT "  
       TACTTTAAGT ATCCATATCC TTCTTTGGGA CAGTTTATCT CTAAAAAAGA AAGCTGGTAG AAAGCTAACA   
  
152811 "TAATACGATA TATAAGGACG ACCGCT"ACTA CAAATAGTAC TACACCCTTG ATCGTGAAAT ATCGATTGCT   
       ATTATGCTAT ATATTCCTGC TGGCGATGAT GTTTATCATG ATGTGGGAAC TAGCAC"TTTA TAGCTAACGA "  
  
152881 TGTTGAACCC "TGTGAATTGC GTGAAAGTAG GATACTCCAA ATTCGGGAGT CTAAGAGTTT TATAAAACGT "  
       "ACAACTTGGG ACACTTAACG CACTTTCATC CTATGAGGTT TAAGCCCTCA GATTCT"CAAA ATATTTTGCA   
  
152951 "TCTTGATGGA" AAAAAATGTG AATGAAAGAT CCCAATGAAT TGAATTGGGT CCATGAATCT AAGAAATAAT   
       AGAACTACCT TTTTTTACAC TTACTTTCTA GGGTTACTTA ACTTAACCCA GGTACTTAGA TTCTTTATTA   
  
153021 GAGAATTCTT GATCTCTCTG AA"TTCTAAAA TCCATAATTT GAATTGCTGC CCTTTCACTG AATTGAGTCC "  
       CTCTTAAGAA CTAGAGAGAC TTAAGATTTT AGGTATTAAA CTTAACGACG GGAAAGTGAC TTAACTCAGG   
  
153091 "TCCTAAATTG CATTGATTTA CC"CCAAAGAT TTCATTTCAA TTGGAATTTG GTTATTCACC ATGTACGAGG   
       AGGATTTAAC GTAACTA"AAT GGGGTTTCTA AAGTAAAGTT AACCTTAAAC CAATAAGTGG TACATGCTCC "  
  
153161 ATCCCCGCTA AGC"ATCCATG GCTGAATGGT TAAAGCGCCC AACTCATAAT TGGCGAATTC GTAGGTTCAA "  
       "TAGGGGCGAT TCGTAGG"TAC CGACTTACCA ATTTCGCGGG TTGAGTATTA ACCGCTTAAG CATCCAAGTT   
  
153231 "TTCCTACTGG ATG"CA"CGCCA ATGGGACCCT CCAATAAGTC TATTGGAATT GGCTCTGTAT CGATGGAATC "  
       AAGGATGACC TACGT"GCGGT TACCCTGGGA GGTTATTCAG ATAACCTTAA CCGAGACATA GCTACCTTAG "  
  
153301 "TCATCATCCA TACAT"AACGA ATTGGTGTGG TATATTCATA TCATAACATA TGAACAGTAA GAACTCGCAT   
       "AGTAGTAGGT ATGTA"TTGCT TAACCACACC ATATAAGTAT AGTATTGTAT ACTTGTCATT CTTGAGCGTA   
  
153371 TCTTATTGAG ACTCGAACTC ATAGGGAAGA AAATTTATGG ATGGAATCAA ATAT"GCAGTA TTTACAGACA "  
       AGAATAACTC TGAGCTTGAG T"ATCCCTTCT TTTAAATACC TACCTTAGTT TATACGTCAT AAATGTCTGT "  
  
153441 "AAAGTATTCG GTTATTGGGG AAAAATCAAT ATACTTCTAA TGTCGAATCA GGAT"CAACTA GGACAGAAAT   
       "TTTCATAAGC CAATAACCCC T"TTTTAGTTA TATGAAGATT ACAGCTTAGT CCTAGTTGAT CCTGTCTTTA   
  
153511 AAAGCA"TTGG GTCGAACTCT TCTTTGGTGT CAAGGTAATA GCTATGAATA GTCATCGACT CCCGGGAAAG "  
       TTTCGTAACC CAGCTTGAGA AGAAACCACA GTT"CCATTAT CGATACTTAT CAGTAGCTGA GGGCCCTTTC "  
  
153581 "GGTAGA"AGAA TGGGACCTAT TAT"GGGACAT ACAATGCATT ACAGACGTAT GATCATTACG CTTCAACCGG "  
       "CCATCTTCTT ACCCTGGATA ATACCCTGTA TGT"TACGTAA TGTCTGCATA CTAGTAATGC GAAGTTGGCC   
  
153651 "GTTATTCTAT TCCACCTCTT AGA"AAGAAAA GAACTTAAAT CAAAATACTT AATAGCATG"G CGATACATTT "  
       CAATAAGATA AGGTGGAGAA TCTTTCTTTT CTT"GAATTTA GTTTTATGAA TTATCGTACC GCTATGTAAA "  
  
153721 "ATACAAAACT TCTACCCCGA GCACACGCAA TGGAGCCGTA GACAGCCAAG TAAAATCCA"A TCCACGAAAT   
       "TATGTTTTGA AGATGGGGCT CGTGTGCGTT ACC"TCGGCAT CTGTCGGTTC ATTTTAGGTT AGGTGCTTTA   
  
153791 AATTTGATCT ATG"GACAGCA TCGTTGTGGT AAAGGTCGTA ATGCCAGAGG AATCATTACC GCAGGGCATA "  
       TTAAACTAGA TACCTGTCGT AGCA"ACACCA TTTCCAGCAT TACGGTCTCC TTAGTAATGG CGTCCCGTAT "  
  
153861 "GAGGGGGAGG TCA"TAAGCGT CTATACCGTA AAATCGATTT TCGACGGAAT GAAAAAGACA TAT"ATGGTAG "  
       "CTCCCCCTCC AGTATTCGCA GATA"TGGCAT TTTAGCTAAA AGCTGCCTTA CTTTTTCTGT ATATACCATC   
  
153931 "AATCGTAACC ATAGAATACG ACCCTAATCG AAATGCATAC ATTTGTCTCA TACACTATGG GGA"TGGTGAG   
       TTAGCATTGG TATCTTATGC TGGGATTAGC TTT"ACGTATG TAAACAGAGT ATGTGATACC CCTACCACTC "  
  
154001 AAAAGATATA TTTTACATCC CAGAGG"GGCT ATAATTGGAG ATACCATTGT TTCTGGTACA GAAGTTCCTA "  
       "TTTTCTATAT AAAATGTAGG GTCTCCCCGA TAT"TAACCTC TATGGTAACA AAGACCATGT CTTCAAGGAT   
  
154071 "TAAAAATGGG AAATGCCCTA CCTTTG"AGTG CGGTTTGAAC TATTGATTTA CGTAATT"GGA AGTAACCAAT "  
       ATTTTTACCC TTTACGGGAT G"GAAACTCAC GCCAAACTTG ATAACTAAAT GCATTAACCT TCATTGGTTA "  
  
154141 "TAGGTTTACG ACGAAACCTA GAAATCGATC ACTGATCCAA TTTGAGTACC TCCACAG"GAT AGACCTCAAC   
       "ATCCAAATGC TGCTTTGGAT C"TTTAGCTAG TGACTAGGTT AAACTCATGG AGGTGTCCTA TCTGGAGTTG   
  
154211 AGAAAACTGA AGAGTAACGG CAGCAAGTGA TTGAGTTCAG TA"GTTCCTCA TATAAAATTA TTGACTCTAG "  
       TCTTTTGACT TCTCAT"TGCC GTCGTTCACT AACTCAAGTC ATCAAGGAGT ATATTTTAAT AACTGAGATC "  
  
154281 "AGATATAGTA ATATGGAGAA GACTAAATTG TTTCAAGCAC CG"ACAGAACC AGAAGCGTCC CTTGTTTCAA   
       "TCTATATCAT TATACC"TCTT CTGATTTAAC AAAGTTCGTG GCTGTCTTGG TCTTCGCAGG GAACAAAGTT   
  
154351 AGAGAGGAGG ACGGGTTATT CACATTTCAT TTGATGGTCG GAGGCAAAT"T GAAAGCTAAG CAGTGGTAAT "  
       TCTCTCCTCC TGCCCAATAA GTGTAAAGTA A"ACTACCAGC CTCCGTTTAA CTTTCGATTC GTCACCATTA "  
  
154421 "TCTAAGGATT CCCGAGGGGA AAAATAGAGA TGTCTCCTAC GTTACCCGT"A ATATGTGGAA GTATCGACGT   
       "AGATTCCTAA GGGCTCCCCT TTTTATCTCT A"CAGAGGATG CAATGGGCAT TATACACCTT CATAGCTGCA   
  
154491 AATTTCATAG AGTCATTCGG TCTGAATGCT ACATGAAGAA CATAAGCCAG ATGAA"GGAAC GGGAAGACCT "  
       TTAAAGTATC TCAGTA"AGCC AGACTTACGA TGTACTTCTT GTATTCGGTC TACTTCCTTG CCCTTCTGGA "  
  
154561 "AGGATGTAGA AGATCATAAC ATGAGTGATT CGGCAGATTT GGATTCCTAT ATATC"CACTC ATATGGTACT   
       "TCCTACATCT TCTAGT"ATTG TACTCACTAA GCCGTCTAAA CCTAAGGATA TATAGGTGAG TATACCATGA   
  
154631 TCATTGTACC ATATATATAA GATCCATATG TATAGATATC ATCATCTACA TCCAGAAAGC CG"TATGCTTT "  
       AGTAACATGG TATATATATT CT"AGGTATAC ATATCTATAG TAGTAGATGT AGGTCTTTCG GCATACGAAA "  
  
154701 "GGAAGAAGCT TGTACAGTTT GGGAAGGGGT TTTGATTGAT CAAAAAGAAG AATCTACTTC AA""CCGATATG "  
       "CCTTCTTCGA ACATGTCAAA CC"CTTCCCCA AAACTAACTA GTTTTTCTTC TTAGATGAAG TTGGCTATAC   
  
154771 "CCCTTAGGCA CGGCCATACA TAACATAGAA ATCACACTTG GGAAGGGGGG ACAATTAGCT AG"AGCAGCAG   
       GGGAATCCGT GCCGGTAT"GT ATTGTATCTT TAGTGTGAAC CCTTCCCCCC TGTTAATCGA TCTCGTCGTC "  
  
154841 GTGCTGTAGC GAAACTGATT GCAAAAGAGG GGAAATCGGC CACATTAAAA TTACC"TTCTG GGGAGGTCCG "  
       "CACGACATCG CTTTGACT"AA CGTTTTCTCC CCTTTAGCCG GTGTAATTTT AATGGAAGAC CCCTCCAGGC   
  
154911 "TTTGATATCC AAAAATTGCT CAGCAACAGT CGGACAAGTG GGGAATGCTG GGGTA"AACCA GAAAAGTTTG   
       AAACTATAGG TTTTTAACGA GTCGTTGTCA G"CCTGTTCAC CCCTTACGAC CCCATTTGGT CTTTTCAAAC "  
  
154981 GGTAGA"GCCG GATCTAAATG TTGGCTAGGT AAGCGTCCTG TAGTAAGAGG AGTAGTTATG AACCCTGTAG "  
       "CCATCTCGGC CTAGATTTAC AACCGATCCA T"TCGCAGGAC ATCATTCTCC TCATCAATAC TTGGGACATC
[truncated: 638 more chars]
