# Supplementary material for: A tiling microarray for global analysis of chloroplast genome expression in cucumber and other plants
Source: Plant Methods. 2011 Sep 28;7:29. doi: 10.1186/1746-4811-7-29 (PMC3195753; doi:10.1186/1746-4811-7-29)
Supplement: Additional file 3 — 1.6 k plastid microarray probe set. This file provides a brief description of microarray experiments A-E and their design. [file 1746-4811-7-29-S3.DOC]

| Experiment symbol | Experiment description | Design | Replicates | Total number of microarrays |
| --- | --- | --- | --- | --- |
| A | Evaluation of the effect of chemical inhibitors of photosynthesis (20 µM DCMU, 40 µM DBMIB) on leaf chloroplast transcriptome | common reference | 3 biological plus 1 technical dye-swap | 8 |
| B | Time-course evaluation of the effect of osmotic stress (0.4 M NaCl) on leaf chloroplast transcriptome | common reference | 3 biological | 18 |
| C | Evaluation of the effect of night cold stress (2 °C) on leaf chloroplast transcriptome | common reference plus direct comparison of some time points | 2-3 biological | 13 |
| D | Comparative analysis of leaf chloroplast transcriptome in wild type and mutant cucumber lines (*tch03* and *msc16)* grown at various temperatures | direct comparison | 2-3 technical | 16 |
| E | Comparative analysis of chloroplast transcriptome in various organs of cucumber (leaves, female flowers, roots, stems) as well as in etiolated seedlings | common reference | 3 technical | 15 |
